# Physical interventions to interrupt or reduce the spread of respiratory viruses

**DOI:** 10.1002/14651858.CD006207.pub6

**Published:** 2023-01-30

**Authors:** Tom Jefferson, Liz Dooley, Eliana Ferroni, Lubna A Al-Ansary, Mieke L Driel, Ghada A Bawazeer, Mark A Jones, Tammy C Hoffmann, Justin Clark, Elaine M Beller, Paul P Glasziou, John M Conly

**Affiliations:** Department for Continuing EducationUniversity of OxfordOxford OX1 2JAUK; Institute for Evidence-Based HealthcareBond UniversityGold CoastAustralia; Epidemiological System of the Veneto RegionRegional Center for Epidemiology, Veneto RegionPadovaItaly; Department of Family and Community MedicineKing Saud UniversityRiyadhSaudi Arabia; General Practice Clinical Unit, Faculty of MedicineThe University of QueenslandBrisbaneAustralia; Department of Public Health and Primary CareGhent UniversityGhentBelgium; Department of Clinical Pharmacy, College of PharmacyKing Saud UniversityRiyadhSaudi Arabia; Cumming School of Medicine, University of CalgaryRoom AGW5, SSB, Foothills Medical CentreCalgaryCanada; O’Brien Institute for Public Health and Synder Institute for Chronic DiseasesCumming School of Medicine, University of CalgaryCalgaryCanada; Calgary ZoneAlberta Health ServicesCalgaryCanada

**Keywords:** Aged, Child, Preschool, Humans, Communicable Disease Control, Communicable Disease Control/methods, COVID-19, COVID-19/epidemiology, COVID-19/prevention & control, Global Health, Global Health/statistics & numerical data, Influenza A Virus, H1N1 Subtype, Influenza, Human, Influenza, Human/epidemiology, Influenza, Human/prevention & control, Randomized Controlled Trials as Topic, Respiratory Tract Infections, Respiratory Tract Infections/epidemiology, Respiratory Tract Infections/prevention & control, SARS-CoV-2

## Abstract

**Background:**

Viral epidemics or pandemics of acute respiratory infections (ARIs) pose a global threat. Examples are influenza (H1N1) caused by the H1N1pdm09 virus in 2009, severe acute respiratory syndrome (SARS) in 2003, and coronavirus disease 2019 (COVID‐19) caused by SARS‐CoV‐2 in 2019. Antiviral drugs and vaccines may be insufficient to prevent their spread. This is an update of a Cochrane Review last published in 2020. We include results from studies from the current COVID‐19 pandemic.

**Objectives:**

To assess the effectiveness of physical interventions to interrupt or reduce the spread of acute respiratory viruses.

**Search methods:**

We searched CENTRAL, PubMed, Embase, CINAHL, and two trials registers in October 2022, with backwards and forwards citation analysis on the new studies.

**Selection criteria:**

We included randomised controlled trials (RCTs) and cluster‐RCTs investigating physical interventions (screening at entry ports, isolation, quarantine, physical distancing, personal protection, hand hygiene, face masks, glasses, and gargling) to prevent respiratory virus transmission.

**Data collection and analysis:**

We used standard Cochrane methodological procedures.

**Main results:**

We included 11 new RCTs and cluster‐RCTs (610,872 participants) in this update, bringing the total number of RCTs to 78. Six of the new trials were conducted during the COVID‐19 pandemic; two from Mexico, and one each from Denmark, Bangladesh, England, and Norway. We identified four ongoing studies, of which one is completed, but unreported, evaluating masks concurrent with the COVID‐19 pandemic.

Many studies were conducted during non‐epidemic influenza periods. Several were conducted during the 2009 H1N1 influenza pandemic, and others in epidemic influenza seasons up to 2016. Therefore, many studies were conducted in the context of lower respiratory viral circulation and transmission compared to COVID‐19. The included studies were conducted in heterogeneous settings, ranging from suburban schools to hospital wards in high‐income countries; crowded inner city settings in low‐income countries; and an immigrant neighbourhood in a high‐income country. Adherence with interventions was low in many studies.

The risk of bias for the RCTs and cluster‐RCTs was mostly high or unclear.

**Medical/surgical masks compared to no masks**

We included 12 trials (10 cluster‐RCTs) comparing medical/surgical masks versus no masks to prevent the spread of viral respiratory illness (two trials with healthcare workers and 10 in the community). Wearing masks in the community probably makes little or no difference to the outcome of influenza‐like illness (ILI)/COVID‐19 like illness compared to not wearing masks (risk ratio (RR) 0.95, 95% confidence interval (CI) 0.84 to 1.09; 9 trials, 276,917 participants; moderate‐certainty evidence. Wearing masks in the community probably makes little or no difference to the outcome of laboratory‐confirmed influenza/SARS‐CoV‐2 compared to not wearing masks (RR 1.01, 95% CI 0.72 to 1.42; 6 trials, 13,919 participants; moderate‐certainty evidence). Harms were rarely measured and poorly reported (very low‐certainty evidence).

**N95/P2 respirators compared to medical/surgical masks**

We pooled trials comparing N95/P2 respirators with medical/surgical masks (four in healthcare settings and one in a household setting). We are very uncertain on the effects of N95/P2 respirators compared with medical/surgical masks on the outcome of clinical respiratory illness (RR 0.70, 95% CI 0.45 to 1.10; 3 trials, 7779 participants; very low‐certainty evidence). N95/P2 respirators compared with medical/surgical masks may be effective for ILI (RR 0.82, 95% CI 0.66 to 1.03; 5 trials, 8407 participants; low‐certainty evidence). Evidence is limited by imprecision and heterogeneity for these subjective outcomes. The use of a N95/P2 respirators compared to medical/surgical masks probably makes little or no difference for the objective and more precise outcome of laboratory‐confirmed influenza infection (RR 1.10, 95% CI 0.90 to 1.34; 5 trials, 8407 participants; moderate‐certainty evidence). Restricting pooling to healthcare workers made no difference to the overall findings. Harms were poorly measured and reported, but discomfort wearing medical/surgical masks or N95/P2 respirators was mentioned in several studies (very low‐certainty evidence).

One previously reported ongoing RCT has now been published and observed that medical/surgical masks were non‐inferior to N95 respirators in a large study of 1009 healthcare workers in four countries providing direct care to COVID‐19 patients.

**Hand hygiene compared to control**

Nineteen trials compared hand hygiene interventions with controls with sufficient data to include in meta‐analyses. Settings included schools, childcare centres and homes. Comparing hand hygiene interventions with controls (i.e. no intervention), there was a 14% relative reduction in the number of people with ARIs in the hand hygiene group (RR 0.86, 95% CI 0.81 to 0.90; 9 trials, 52,105 participants; moderate‐certainty evidence), suggesting a probable benefit. In absolute terms this benefit would result in a reduction from 380 events per 1000 people to 327 per 1000 people (95% CI 308 to 342). When considering the more strictly defined outcomes of ILI and laboratory‐confirmed influenza, the estimates of effect for ILI (RR 0.94, 95% CI 0.81 to 1.09; 11 trials, 34,503 participants; low‐certainty evidence), and laboratory‐confirmed influenza (RR 0.91, 95% CI 0.63 to 1.30; 8 trials, 8332 participants; low‐certainty evidence), suggest the intervention made little or no difference. We pooled 19 trials (71, 210 participants) for the composite outcome of ARI or ILI or influenza, with each study only contributing once and the most comprehensive outcome reported. Pooled data showed that hand hygiene may be beneficial with an 11% relative reduction of respiratory illness (RR 0.89, 95% CI 0.83 to 0.94; low‐certainty evidence), but with high heterogeneity. In absolute terms this benefit would result in a reduction from 200 events per 1000 people to 178 per 1000 people (95% CI 166 to 188). Few trials measured and reported harms (very low‐certainty evidence).

We found no RCTs on gowns and gloves, face shields, or screening at entry ports.

**Authors' conclusions:**

The high risk of bias in the trials, variation in outcome measurement, and relatively low adherence with the interventions during the studies hampers drawing firm conclusions. There were additional RCTs during the pandemic related to physical interventions but a relative paucity given the importance of the question of masking and its relative effectiveness and the concomitant measures of mask adherence which would be highly relevant to the measurement of effectiveness, especially in the elderly and in young children.

There is uncertainty about the effects of face masks. The low to moderate certainty of evidence means our confidence in the effect estimate is limited, and that the true effect may be different from the observed estimate of the effect. The pooled results of RCTs did not show a clear reduction in respiratory viral infection with the use of medical/surgical masks. There were no clear differences between the use of medical/surgical masks compared with N95/P2 respirators in healthcare workers when used in routine care to reduce respiratory viral infection. Hand hygiene is likely to modestly reduce the burden of respiratory illness, and although this effect was also present when ILI and laboratory‐confirmed influenza were analysed separately, it was not found to be a significant difference for the latter two outcomes. Harms associated with physical interventions were under‐investigated.

There is a need for large, well‐designed RCTs addressing the effectiveness of many of these interventions in multiple settings and populations, as well as the impact of adherence on effectiveness, especially in those most at risk of ARIs.

## Plain language summary

**Do physical measures such as hand‐washing or wearing masks stop or slow down the spread of respiratory viruses?**

**Key messages** We are uncertain whether wearing masks or N95/P2 respirators helps to slow the spread of respiratory viruses based on the studies we assessed.

Hand hygiene programmes may help to slow the spread of respiratory viruses.

**How do respiratory viruses spread?** Respiratory viruses are viruses that infect the cells in your airways: nose, throat, and lungs. These infections can cause serious problems and affect normal breathing. They can cause flu (influenza), severe acute respiratory syndrome (SARS), and COVID‐19.

People infected with a respiratory virus spread virus particles into the air when they cough or sneeze. Other people become infected if they come into contact with these virus particles in the air or on surfaces on which they land. Respiratory viruses can spread quickly through a community, through populations and countries (causing epidemics), and around the world (causing pandemics).

Physical measures to try to prevent respiratory viruses spreading between people include:

· washing hands often;

· not touching your eyes, nose, or mouth;

· sneezing or coughing into your elbow;

· wiping surfaces with disinfectant;

· wearing masks, eye protection, gloves, and protective gowns;

· avoiding contact with other people (isolation or quarantine);

· keeping a certain distance away from other people (distancing); and

· examining people entering a country for signs of infection (screening).

**What did we want to find out?** We wanted to find out whether physical measures stop or slow the spread of respiratory viruses from well‐controlled studies in which one intervention is compared to another, known as randomised controlled trials.

**What did we do?** We searched for randomised controlled studies that looked at physical measures to stop people acquiring a respiratory virus infection.

We were interested in how many people in the studies caught a respiratory virus infection, and whether the physical measures had any unwanted effects.

**What did we find?** We identified 78 relevant studies. They took place in low‐, middle‐, and high‐income countries worldwide: in hospitals, schools, homes, offices, childcare centres, and communities during non‐epidemic influenza periods, the global H1N1 influenza pandemic in 2009, epidemic influenza seasons up to 2016, and during the COVID‐19 pandemic. We identified five ongoing, unpublished studies; two of them evaluate masks in COVID‐19. Five trials were funded by government and pharmaceutical companies, and nine trials were funded by pharmaceutical companies.

No studies looked at face shields, gowns and gloves, or screening people when they entered a country.

We assessed the effects of:

· medical or surgical masks;

· N95/P2 respirators (close‐fitting masks that filter the air breathed in, more commonly used by healthcare workers than the general public); and

· hand hygiene (hand‐washing and using hand sanitiser).

We obtained the following results:

*Medical or surgical masks*

Ten studies took place in the community, and two studies in healthcare workers. Compared with wearing no mask in the community studies only, wearing a mask may make little to no difference in how many people caught a flu‐like illness/COVID‐like illness (9 studies; 276,917 people); and probably makes little or no difference in how many people have flu/COVID confirmed by a laboratory test (6 studies; 13,919 people). Unwanted effects were rarely reported; discomfort was mentioned.

*N95/P2 respirators*

Four studies were in healthcare workers, and one small study was in the community. Compared with wearing medical or surgical masks, wearing N95/P2 respirators probably makes little to no difference in how many people have confirmed flu (5 studies; 8407 people); and may make little to no difference in how many people catch a flu‐like illness (5 studies; 8407 people), or respiratory illness (3 studies; 7799 people). Unwanted effects were not well‐reported; discomfort was mentioned.

*Hand hygiene*

Following a hand hygiene programme may reduce the number of people who catch a respiratory or flu‐like illness, or have confirmed flu, compared with people not following such a programme (19 studies; 71,210 people), although this effect was not confirmed as statistically significant reduction when ILI and laboratory‐confirmed ILI were analysed separately. Few studies measured unwanted effects; skin irritation in people using hand sanitiser was mentioned.

**What are the limitations of the evidence?** Our confidence in these results is generally low to moderate for the subjective outcomes related to respiratory illness, but moderate for the more precisely defined laboratory‐confirmed respiratory virus infection, related to masks and N95/P2 respirators. The results might change when further evidence becomes available. Relatively low numbers of people followed the guidance about wearing masks or about hand hygiene, which may have affected the results of the studies.

**How up to date is this evidence?** We included evidence published up to October 2022.

## Summary of findings

**Summary of findings 1:** Medical/surgical masks compared to no masks for preventing the spread of viral respiratory illness

**Randomised studies: medical/surgical masks compared to no masks for preventing the spread of viral respiratory illness**						
**Patient or population:** general population **Setting:** community and hospitals **Intervention:** medical/surgical masks **Comparison:** no masks						
**Outcomes**	Anticipated absolute effects^*^ (95% CI)		**Relative effect (95% CI)**	**№ of participants (studies)**	**Certainty of the evidence (GRADE)**	**Comments**
	**Risk with no masks**	**Risk with randomised studies: masks**				
Viral respiratory illness ‐ influenza/COVID‐like illness	Study population		RR 0.95 (0.84 to 1.09)	276,917 (9 RCTs)	⊕⊕⊕⊝ Moderate^a^	
	160 per 1000	152 per 1000 (134 to 174)				
Viral respiratory illness ‐ laboratory‐confirmed influenza/SARS‐CoV‐2	Study population		RR 1.01 (0.72 to 1.42)	13,919 (6 RCTs)	⊕⊕⊕⊝ Moderate^b^	
	40 per 1000	40 per 1000 (29 to 57)				
Adverse events	‐		‐	(3 RCTs)	⊕⊝⊝⊝ Very low^a,c^	Adverse events were not reported consistently and could not be meta‐analysed. Adverse events reported for masks included warmth, discomfort, respiratory difficulties, humidity, pain, and shortness of breath, in up to 45% of participants.
*The risk in the intervention group (and its 95% confidence interval) is based on the median observed risk in the comparison group of included studies and the **relative effect** of the intervention (and its 95% CI). **CI:** confidence interval; **RCT:** randomised controlled trial; **RR:** risk ratio						
**GRADE Working Group grades of evidence** **High certainty:** we are very confident that the true effect lies close to that of the estimate of the effect. **Moderate certainty:** we are moderately confident in the effect estimate: the true effect is likely to be close to the estimate of the effect, but there is a possibility that it is substantially different. **Low certainty:** our confidence in the effect estimate is limited: the true effect may be substantially different from the estimate of the effect. **Very low certainty:** we have very little confidence in the effect estimate: the true effect is likely to be substantially different from the estimate of effect.						

^a^Downgraded one level for study limitations (lack of blinding). ^b^Downgraded one level for imprecision (wide confidence intervals). ^c^Downgraded two levels for imprecision (only three studies enumerated adverse events; another study mentioned no adverse events).

**Summary of findings 2:** N95 respirators compared to medical/surgical masks for preventing the spread of viral respiratory illness

**Randomised studies: N95 respirators compared to medical/surgical masks for preventing the spread of viral respiratory illness**						
**Patient or population:** general population and healthcare workers **Setting:** hospitals and households **Intervention:** N95 masks **Comparison:** medical/surgical masks						
**Outcomes**	Anticipated absolute effects^*^ (95% CI)		**Relative effect (95% CI)**	**№ of participants (studies)**	**Certainty of the evidence (GRADE)**	**Comments**
	**Risk with medical masks**	**Risk with randomised studies: N95**				
Viral respiratory illness ‐ clinical respiratory illness	Study population		RR 0.70 (0.45 to 1.10)	7799 (3 RCTs)	⊕⊝⊝⊝ Very Low^a,b,c^	All studies were conducted in hospital settings with healthcare workers.
	120 per 1000	84 per 1000 (54 to 132)				
Viral respiratory illness ‐ influenza‐like illness	Study population		RR 0.82 (0.66 to 1.03)	8407 (5 RCTs)	⊕⊕⊝⊝ Low^a,b^	1 study was conducted in households (MacIntyre 2009).
	50 per 1000	41 per 1000 (33 to 52)				
Viral respiratory illness ‐ laboratory‐confirmed influenza	Study population		RR 1.10 (0.90 to 1.34)	8407 (5 RCTs)	⊕⊕⊕⊝ Moderate^b^	1 study was conducted in households (MacIntyre 2009).
	70 per 1000	77 per 1000 (63 to 94)				
Adverse events	‐		‐	(5 RCTs)	⊕⊝⊝⊝ Very Low^a,b,c^	There was insufficient consistent reporting of adverse events to enable meta‐analysis. Only 1 study reported detailed adverse events: discomfort was reported in 41.9% of N95 wearers versus 9.8% of medical mask wearers (P < 0.001); headaches were more common with N95 (13.4% versus 3.9%; P < 0.001); difficulty breathing was reported more often in the N95 group (19.4% versus 12.5%; P = 0.01); and N95 caused more problems with pressure on the nose (52.2% versus 11.0%; P < 0.001). 4 RCTs either reported no adverse events or only reported on comfort wearing masks.
*The risk in the intervention group (and its 95% confidence interval) is based on the median risk in the comparison group and the observed **relative effect** of the intervention (and its 95% CI). **CI:** confidence interval; **RCT:** randomised controlled trial; **RR:** risk ratio						
**GRADE Working Group grades of evidence** **High certainty:** we are very confident that the true effect lies close to that of the estimate of the effect. **Moderate certainty:** we are moderately confident in the effect estimate: the true effect is likely to be close to the estimate of the effect, but there is a possibility that it is substantially different. **Low certainty:** our confidence in the effect estimate is limited: the true effect may be substantially different from the estimate of the effect. **Very low certainty:** we have very little confidence in the effect estimate: the true effect is likely to be substantially different from the estimate of effect.						

^a^Downgraded one level for study limitations (lack of blinding). ^b^Downgraded one level for imprecision (wide confidence interval or no meta‐analysis conducted). ^c^Downgraded one level for inconsistency of results (heterogeneity).

**Summary of findings 3:** Hand hygiene compared to control for preventing the spread of viral respiratory illness

**Hand hygiene compared to control for preventing the spread of viral respiratory illness**						
**Patient or population:** general population and healthcare workers **Setting:** schools, childcare centres, homes, offices, nursing homes **Intervention:** hand hygiene **Comparison:** control						
**Outcomes**	Anticipated absolute effects^*^ (95% CI)		**Relative effect (95% CI)**	**№ of participants (studies)**	**Certainty of the evidence (GRADE)**	**Comments**
	**Risk with control**	**Risk with hand hygiene**				
Acute respiratory illness	Study population		RR 0.86 (0.81 to 0.90)	52,105 (9 RCTs)	⊕⊕⊕⊝ Moderate^a^	
	380 per 1000	327 per 1000 (308 to 342)				
Influenza‐like illness	Study population		RR 0.94 (0.81 to 1.09)	34,503 (11 RCTs)	⊕⊕⊝⊝ Low^a,b^	
	90 per 1000	85 per 1000 (73 to 98)				
Laboratory‐confirmed influenza	Study population		RR 0.91 (0.63 to 1.30)	8332 (8 RCTs)	⊕⊕⊝⊝ Low^b,c^	
	80 per 1000	73 per 1000 (50 to 104)				
Composite of acute respiratory illness, influenza‐like illness, laboratory‐confirmed influenza	Study population		RR 0.89 (0.83 to 0.94)	71,210 (19 RCTs)	⊕⊕⊝⊝ Low^a,b^	
	200 per 1000	178 per 1000 (166 to 188)				
Adverse events	‐		‐	(2 RCTs)	⊕⊝⊝⊝ Very low^a,b,c^	Data were insufficient to conduct meta‐analysis. 1 study reported that no adverse events were observed, and another study reported that skin reaction was recorded for 10.4% of participants in the hand sanitiser group versus 10.3% in the control group.
*The risk in the intervention group (and its 95% confidence interval) is based on the median observed risk in the comparison groups of included studies and the **relative effect** of the intervention (and its 95% CI). **CI:** confidence interval; **RCT:** randomised controlled trial; **RR:** risk ratio						
**GRADE Working Group grades of evidence** **High certainty:** we are very confident that the true effect lies close to that of the estimate of the effect. **Moderate certainty:** we are moderately confident in the effect estimate: the true effect is likely to be close to the estimate of the effect, but there is a possibility that it is substantially different. **Low certainty:** our confidence in the effect estimate is limited: the true effect may be substantially different from the estimate of the effect. **Very low certainty:** we have very little confidence in the effect estimate: the true effect is likely to be substantially different from the estimate of effect.						

^a^Downgraded one level for study limitation (majority of studies were unblinded, with participant‐assessed outcome). ^b^Downgraded one level for inconsistent results across studies. ^c^Downgraded one level for imprecision (wide confidence interval or no meta‐analysis conducted).

## Background

### Description of the condition

Epidemic and pandemic viral infections pose a serious threat to people worldwide. Epidemics of note include severe acute respiratory syndrome (SARS) in 2003 and the Middle East respiratory syndrome (MERS), which began in 2012, and the current SARS‐CoV‐2 pandemic. Major pandemics include the H1N1 influenza caused by the H1N1pdm09 virus in 2009 and the coronavirus disease 2019 (COVID‐19) caused by SARS‐CoV‐2.

Even non‐epidemic acute respiratory infections (ARIs) place a huge burden on healthcare systems around the world, and are a prominent cause of morbidity (WHO 2017). Furthermore, ARIs are often antecedents to lower respiratory tract infections (RTIs) caused by bacterial pathogens (i.e. pneumonia), which cause millions of deaths worldwide, mostly in low‐income countries (Schwartz 2018).

High viral load, high levels of transmissibility, susceptible populations, and symptomatic patients are considered to be the drivers of such epidemics and pandemics (Jefferson 2006a). Preventing the spread of respiratory viruses from person to person may be effective at reducing the spread of outbreaks. 

Physical interventions, such as the use of masks and physical distancing measures, might prevent the spread of respiratory viruses which are considered to be transmitted by multiple modes of transmission including by respiratory particles of varying sizes spreading from infected to susceptible people and through direct and indirect contact (Kutter 2018; Leung 2021). It is recognised that there is a continuum of respiratory particle sizes varying between large droplet to fine aerosols, which is an important concept. Particles of a variety of sizes may be expelled from the human airway during coughing, sneezing, singing, talking, and during certain medical procedures (WHO 2021). In addition, transmission of respiratory viruses is likely highly complex, dependent on multiple host, virus and environmental factors, plus the myriad of interactions between these factors, which may influence the predominant modes of transmission in any given setting (Broderick 2008; Hendley 1988; Kutter 2018; Leung 2021). Current evidence suggests that the virus responsible for the current COVID‐19 pandemic spreads mainly between people who are in close contact with each other (Onakpoya 2022a).

It is also unknown if all respiratory viruses or different strains of a specific respiratory virus transmit in a similar manner, further adding to the complexity of respiratory virus transmission. 

### Description of the intervention

Single measures of intervention such as the use of vaccines or antivirals, may be insufficient to contain the spread of influenza, but combinations of interventions may reduce the reproduction number to below 1 (Demicheli 2018a; Demicheli 2018b; Jefferson 2014; Jefferson 2018; Thomas 2010). When the reproduction number (or R0) is below 1, each infection causes less than one new secondary infection and the disease will eventually die out. For some respiratory viruses there are no licensed interventions, and a combination of social and physical interventions may be the only option to reduce the spread of outbreaks, particularly those that may be capable of becoming epidemic or pandemic in nature (Luby 2005). Such interventions were emphasised in the World Health Organization's latest Global Influenza Strategy 2019 to 2030, and have several possible advantages over other methods of suppressing ARI outbreaks since they may be instituted rapidly and may be independent of any specific type of infective agent, including novel viruses. In addition, the possible effectiveness of public health measures during the Spanish flu pandemic of 1918 to 1919 in US cities supports the impetus to investigate the existing evidence on the effectiveness of such interventions (Bootsma 2007), including quarantine (such as isolation, physical distancing) and the use of disinfectants. We also considered the major societal implications for any community adopting these measures (CDC 2005a; CDC 2005b; WHO 2006b; WHO 2020a; WHO 2020b).

### How the intervention might work

Epidemics and pandemics are more likely during antigenic change (changes in the viral composition) in the virus or transmission from animals (domestic or wild) when there is no natural human immunity (Bonn 1997). High viral load, high levels of transmissibility, and symptomatic patients are considered to be the drivers of such epidemics and pandemics (Jefferson 2006b).

Physical interventions, such as the use of masks (Greenhalgh 2020; Howard 2020), physical distancing measures, school closures, and limitations of mass gatherings, might prevent the spread of the virus transmitted by infectious respiratory particles from infected to susceptible individuals. The use of hand hygiene, gloves, and protective gowns can also prevent the spread by limiting the transfer of viral particles onto and from fomites (inanimate objects such as flat surfaces, tabletops, utensils, porous surfaces, or nowadays cell phones, which can transmit the agent if contaminated) (Onakpoya 2022b). Such public health measures were widely adopted during the Spanish flu pandemic and have been the source of considerable debate (Bootsma 2007).

### Why it is important to do this review

Although the benefits of physical interventions seem self‐evident, given the global importance of interrupting respiratory virus transmission, having up‐to‐date estimates of their effectiveness is necessary to inform planning, decision‐making, and policy. The continuance of outbreaks of COVID‐19 and the reporting of several new trials assessing different barrier interventions in preventing the spread of SARS‐COV‐2 virus, have prompted this update (WHO 2022). Physical methods have several possible advantages over other methods of suppressing ARI outbreaks, including their rapid deployment and ability to be independent of the infective agent, including novel viruses.

The hallmark of the 2020 update was shifting from including all types of studies to a focus on randomised controlled trials (RCTs) only, which had substantially increased in number. This change enabled more robust evidence summaries from high‐quality studies, which are much less prone to the risk of the multiple biases associated with observational studies, to help policy and decision makers in making national and global recommendations. The 2020 update identified 67 relevant studies, but none were carried out during the COVID‐19 pandemic (Jefferson 2020). The three key messages of that update were: (1) hand hygiene programmes may help to slow the spread of respiratory viruses; (2) uncertainty whether wearing masks or N95/P2 respirators would help in slowing the spread of respiratory viruses; and (3) few studies were identified for other interventions. One study looked at quarantine, and none looked at eye protection, gowns and gloves, or screening people when they entered a country*.* However, during the last search of the 2020 update, six ongoing, unpublished studies were identified; three of them evaluate masks in COVID‐19. The review authors are aware that several trials have now been published since the publication of the 2020 update, warranting this new update. 

This is the fifth update (Jefferson 2009; Jefferson 2010; Jefferson 2011; Jefferson 2020) of a Cochrane Review first published in 2007 (Jefferson 2007).

## Objectives

To assess the effectiveness of physical interventions to interrupt or reduce the spread of acute respiratory viruses.

## Methods

### Criteria for considering studies for this review

#### Types of studies

For this 2022 update we only considered individual‐level randomised controlled trials (RCTs), or cluster‐RCTs, or quasi‐RCTs for inclusion.

In versions of this review prior to 2020 we also included observational studies (cohorts, case‐controls, before‐after, and time series studies). However, for this update there were sufficient randomised studies to address our study aims, so we excluded observational studies because randomisation is the optimal method to prevent systematic differences between participants in different intervention groups and, further, deciding who receives an intervention and who does not is influenced by many factors, including prognostic factors (Higgins 2011). This point is particularly relevant here because individuals who chose to implement physical interventions are likely to use multiple interventions, thus making it difficult to separate out the effect of single interventions. Further, they are likely to be different from individuals who do not implement physical interventions in ways that are difficult to measure.

#### Types of participants

People of all ages.

#### Types of interventions

We included RCTs and cluster‐RCTs of trials investigating physical interventions or combinations of interventions to prevent respiratory virus transmission compared with doing nothing or with other interventions. The interventions of interest included: screening at entry ports, isolation, quarantine, physical distancing, personal protection (clothing, gloves, devices), hand hygiene, face masks, gargling, nasal washes, eye protective devices, face shields, disinfecting, and school closure. 

#### Types of outcome measures

For the outcomes listed below we had no predetermined key time points of interest or adverse events of special interest, however, methods of assessment of cases of viral respiratory illness based on laboratory‐confirmation needed to be based on an accurate test in combination with critical additional information. For example, a polymerase chain reaction (PCR) test in combination with symptoms of disease, or a serological test at baseline as well as at the end of follow‐up were acceptable methods. Further, we stratified analyses by study‐specific definitions for cases of viral respiratory illness which included a broad definition of acute respiratory infection (ARI), a more specific definition of influenza‐like‐illness (ILI), and the most precise definition of a laboratory‐confirmed respiratory infection that identified the actual viral pathogen. For the studies conducted during the COVID‐19 pandemic, we assumed that COVID‐like illness was interchangeable with ILI. In the case of laboratory‐confirmed respiratory infection we separated out SARS‐CoV‐2/influenza and other viral pathogens. We did not pool these outcomes as it cannot be assumed that the effects of physical interventions will be the same for the different viral pathogens. The one exception was for the comparison of hand‐hygiene versus control where the estimated effects for ARI, ILI and laboratory‐confirmed infection were highly consistent. 

##### Primary outcomes

1. Numbers of cases of viral respiratory illness (including acute respiratory infections (ARI), influenza‐like illness (ILI), COVID‐like illness and laboratory‐confirmed influenza, SARS‐CoV‐2 or other viral pathogens).
2. Adverse events related to the intervention.

##### Secondary outcomes

1. Deaths.
2. Severity of viral respiratory illness as reported in the studies.
3. Absenteeism.
4. Hospital admissions.
5. Complications related to the illness, e.g. pneumonia.

### Search methods for identification of studies

#### Electronic searches

For this 2022 update, we refined the original search strategy using a combination of previously included studies and automation tools (Clark 2020). We converted this search using the Polyglot Search Translator (Clark 2020), and ran the searches in the following databases:

1. the Cochrane Central Register of Controlled Trials (CENTRAL) (2022, Issue 09), which includes the Acute Respiratory Infections Group's Specialised Register (searched 04 October 2022) (Appendix 1);
2. PubMed (01 January 2020 to 04 October 2022) (Appendix 2);
3. Embase (01 January 2020 to 04 October 2022) (Appendix 3);
4. CINAHL (Cumulative Index to Nursing and Allied Health Literature) (01 January 2020 to 04 October) (Appendix 4);
5. US National Institutes of Health Ongoing Trials Register ClinicalTrials.gov (January 2010 to 04 October 2022); and
6. World Health Organization International Clinical Trials Registry Platform (January 2010 to 04 October 2022).

We combined the database searches with the Cochrane Highly Sensitive Search Strategy for identifying randomised trials in MEDLINE: sensitivity‐ and precision‐maximising version (2008 revision) (Lefebvre 2011). Details of previous searches are available in Appendix 5.

#### Searching other resources

We conducted a backwards‐and‐forwards citation analysis in Scopus on all newly included studies to identify other potentially relevant studies.

### Data collection and analysis

#### Selection of studies

The search and citation analysis results were initially screened via the RobotSearch tool (Marshall 2018) to exclude all studies that were obviously not RCTs. We scanned the titles and abstracts of studies identified by the searches. We obtained the full‐text articles of studies that either appeared to meet our eligibility criteria or for which there was insufficient information to exclude it. We then used a standardised form to assess the eligibility of each study based on the full article.

#### Data extraction and management

Five review authors (LA/GB/EF/EB/TOJ) independently applied the inclusion criteria to all identified and retrieved articles, and extracted data using a standard template that had been developed for and applied to previous versions of the review, but was revised to reflect our focus on RCTs and cluster‐RCTs for this update. We resolved any disagreements through discussion with either PG or JMC acting as arbiter. We extracted and reported descriptions of interventions using the Template for Intervention Description and Replication (TIDieR) template (Table 4). 

**1:** Description of interventions in included studies, using the items from the Template for Intervention Description and Replication (TIDieR) checklist

**Author, year**	**Brief name**	**Recipient**	**Why**	**What (materials)**	**What (procedures)**	**Who provided**	**How**	**Where**	**When and how much**	**Tailoring**	**Modification of intervention throughout trial**	**Strategies to improve or maintain intervention fidelity**	**Extent of intervention fidelity**
**Masks compared to either no masks or different mask types**													
Abaluck 2022 (additional sources: Abaluck 2021a, Abaluck 2021b, Kwong 2021)	Community‐level mask promotion and distribution of free masks. A. Cloth masks or B. Surgical masks with possible additional village level elements: i) incentive ii) signage iii) text message reminder and household elements: i) altruism or self‐protection messages ii) amount of households receiving texts iii) commitment to mask‐wearing	Leaders and adult householders of rural and peri‐urban villages	Increase large‐scale adoption and proper wearing of face masks to slow the spread of COVID‐19 and save lives informed by research in public health, psychology, economics, marketing, and other social sciences on product promotion and dissemination strategies	Masks colour‐coded by households, either: A. cloth masks: an exterior layer of 100% non‐woven polypropylene (70 grams/m^2^ [gsm]), 2 interior layers of 60% cotton/40% polyester interlocking knit (190 gsm), an elastic loop that goes around the head above and below the ears, and a nose bridge; filtration efficiency: 37%^[1]^ B. 3 layers of 100% non woven polypropylene^[2]^, elastic ear loops, and a nose bridge; filtration efficiency: 95%. Sticker that had a logo of a mask with an outline of the Bangladeshi flag and a phrase in Bengali that noted the mask could be washed and reused^[3]^; filtration efficiency of 76% Initial 3 masks per household Video of notable public figures^[4]^ discussing why, how, and when to wear a mask Brochure based on WHO materials depicting proper mask‐wearing Scripted speeches for use by role models and local leaders at Friday prayers Scripted text messages Monetary rewards (USD 190) or non‐monetary reward (certificate) for villages Signage for household doors declaring they are a mask‐wearing household Smart phone for delivery and receipt of text message reminders Loudspeaker for announcements in markets by research staff Masks woven by and procured from local Bangladeshi garment factories within 6 weeks after ordering: $0.50 per cloth mask and $0.13 per surgical mask Masks and hand sanitiser for staff delivering intervention Costs: Cloth masks: $275.10/village Surgical masks: $88.90/village PPE for staff: $70/village Media costs: $100/village Transport and other costs: $30/village Handouts and written and some audio scripts for role models, leaders, surveillance officers and texts etc provided by the research team and in online protocol supplement via osf.io/23mws/	All villages: 1. household distribution of surgical or cloth masks and showing of mask‐wearing video; 2. distribution and promotion of masks at village markets; 3. mask distribution at mosques; 4. mask promotion in public spaces; 5. role modelling and advocacy by local leaders, including Imams during Friday prayers using a scripted speech. Periodic monitoring of passers‐by and reminding people to put on masks Some villages: village police accompanying mask promoters, providing monetary rewards or certificates to villages if mask‐wearing rate improves. Some villages: public signalling of mask‐wearing via signage, text message reminders, messaging emphasizing either altruistic or self‐protection motives for mask‐wearing, and extracting verbal commitments from households. Modelling of safe mask wearing by study staff Detailed procedures outlined in online protocol supplement osf.io/23mws/	Local NGO staff and volunteers (Bangladeshi NGO GreenVoice)^[5]^ and Innovations for Poverty Action (IPA) Village Imams and police officers No “specialized skills” needed as intervention designed to be easily adopted by other NGOs or agencies Training of staff provided by researchers for mask promotion	Masks and promotion delivered face to face in households, markets, mosques and streets of villages both as groups and individually Text messages delivered by phone and individually	Households, markets, mosques and streets of 572 villages (in rural Bangladesh)	8 weeks per village rolled out over a 6 week period (November 2020 to January 2021) 1 day of training per village Once off mask distribution and promotion at households (4 days / village) Mask distribution 3 to 6 days / week at markets and on 3 Fridays at mosques during the first 4 weeks Weekly or biweekly mask promotion Role‐modelling and leader advocacy at Friday prayers Periodic monitoring: 1/week on weeks 1, 2, 4, 6, 8, and 10; daily schedule provided in Protocol – 1 hour per site for 9 sites 8am to 5pm Each village observed on 2 alternating days of the week. Observations occurred 7 days of the week (9 am to 7 pm) Detailed schedules provided in online protocol supplement via osf.io/23mws/	Periodic monitoring and then additional training of staff provided as needed Different locations and timing of observation across different days	In the first 5 weeks of the study staff found low engagement in some villages with local mask use, so mask promotion staff were retrained by researcher part‐way through the intervention “to work more closely with local leaders and set specific milestones for that partnership” After 5 weeks, monitoring of mask‐wearing was limited to those who appeared to be 18 years or older.	Numbers of masks distributed was noted Promoters periodically monitored passers‐by and reminded people to put on masks Direct surveillance of mask wearing, correct mask‐wearing (wearing either a project mask or an alternative face‐covering over the mouth and nose) and physical distancing (if s/he was at least one arm’s length away from the nearest person)^[6]^ Monetary rewards or certificates to villages if mask‐wearing rate improved Additional training for mask promotion staff Recording of activities undertaken by intervention staff including the degree to which leaders or imams understood the script, sites observed etc (see p.9 of Protocol osf.io/23mws/) “consistent with the WHO guideline that defines physical distancing as one meter of separation.” www.who.int/westernpacific/emergencies/covid-19/information/physical-distancing (accessed 13 June 2022).	Numbers of masks distributed: A. 370,643 B. 924,849 Mask‐wearing: IGs: 42.3% CG: 13.3% Increase was largest in mosques (37% points) and 25% to 29% points in other locations Proper mask‐wearing increased by 29.0% Physical distancing increased from 24.1% in CG villages to 29.2% in IG villages No difference between IGs and CGs in number of people observed in public areas, as an indication of social distancing.
Alfelali 2020	Face masks	Hajj pilgrims aged ≥ 18 years	Prevent and control viral respiratory infections at mass gatherings	50 surgical face masks per participant (3M™ Standard Tie‐On surgical mask, Cat No: 1816) Written instructions for mask use (See S1 Appendix)	Provide masks and verbal and printed instructions, rules for mask use and demonstration of appropriate mask usage provided (See S1 Appendix) Rules for mask use: • ”Try to avoid touching the front of the mask. • Change your mask if it is damp, wet or dirty. • Always clean your hands before and after changing the masks. • Put used masks in a plastic bag and throw it into a rubbish bin. You will find bins somewhere close to your tent in Mina.”	464 volunteer trained research team members approached pilgrims in their tents Training included how to approach pilgrims and explanation and demonstration of mask use	Individually and face to face to groups of pilgrims in tents	Tents of pilgrims for Hajj in Makkah (Saudi Arabia) 50 to 150 pilgrims per large tent, sleeping head‐to‐head and sharing meals and rites	Mask wearing for 24 hours if possible, over days of Hajj season inside and outside assigned tents 3 consecutive Hajj seasons (5 to 6 days, October 2013 to 2015)	Written information provided in preferred language (Arabic or English) Pilgrims who used at least 1 mask each day were considered to have used the mask during that day (i.e. could be < 24 hours)	None described	4 day diaries of mask use: number of masks used and hours worn each day (see S1 Appendix)	Mask use: IG: Daily: 24.7% Intermittently: 47.7% None: 20.9% CG: Daily: 14.3% Intermittently: 34.9% None: 43.7% Mask use of at least 4 hours consistently greater in IG than CG
Barasheed 2014	Supervised mask use	Religious pilgrims ≥ 15 years	Prevent respiratory virus infections at mass gatherings through mask use	Plain surgical face masks (3M Standard Tie‐On Surgical Mask, Cat No: 1816) manufactured by 3M company, USA; 5 masks per day Written instructions on face mask use Special polythene bags for disposal	Masks provided to index case and their contacts with advice on mask use (before prayers, in seminars, and after meals). Written instructions provided on face mask use, need to change them, and disposal.	Not described, presumably the medical researchers	Face‐to‐face provision of masks, instructions, and reminders	Tents of pilgrimage site (Mina Valley, Saudi Arabia)	Advice on mask use given throughout pilgrimage stay (5 days)	None reported.	None reported.	The medical researchers followed pilgrims each day to remind participants about recording their mask usage in health diary.	Face mask use: mask group: 56/75 (76%), control group: 11/89 (12%) (P < 0.001) 76% of intervention tents wore masks. 10 of 75 (13%) pilgrims in ‘mask’ tents wore face masks during sleep.
Bundgaard 2021 (additional source Bundgaard 2020)	Face masks (surgical)	Community‐dwelling adults aged 18 years or older with internet access	Reduce wearers' risk for SARS‐CoV‐2 infection outside the home through protection of the nose and mouth from droplets or aerosols or contaminated fingers and hands	Per participant: 50 x 3‐layer, disposable, surgical face masks with ear loops (TYPE II EN 14683 (Abena, Denmark); filtration rate, 98%; made in China) 1 badge (saying: “I am testing face masks – for you and me”) Written instructions and instructional videos for proper use of masks (See supplement 8) of published paper including link to video for proper face mask use [in Danish] vimeo.com/406952695	Supply of masks sent to home address by courier Provision of written instructions sent by courier about how and when to wear masks including links to instructional video for face mask use Instruction to follow advice of local health authorities (in Denmark) Provision of follow‐up support by email and a phone help‐line for questions	Researchers provided the masks (funded by Salling Group), instructions and follow‐up support Background and training of researcher not described Hotline provided medical expertise and guidance, (qualification and training needed for this support not specified)	Individually by mail, email, online and telephone	Mask wearing: when outside the home ‐ and in the home when they had guests (in Denmark) Instructions and support at home and online	Mask wearing: whenever outside the home or when guests in the home, up to 8 hours for 1 mask, for 1 month (April to May 2020) 1 off instructions for mask use and again as needed Weekly follow‐up emails Hotline available at all times during study period	Changing of mask if worn for more than 8 hours If guests in the home, wear mask Individualised support as needed via email or telephone	None described	Face mask adherence: Self‐report (Yes / Partial / No) (Suppl 4) Average mask use per day Self‐assessed adherence with health authority guideline on social distancing and hygiene (Suppl)	Face mask adherence: % Adhere: 46% Partial: 47% No: 7% Mean face masks used: Weekdays: 1.7 Weekends: 1.3 Health authority guidance adherence not reported
Canini 2010	Surgical face masks	Householders (over 5 years)	Limit transmission of influenza transmission by large droplets produced during coughing in households	Initial supply of 30 masks: for adults and children > 10: surgery masks with ear loops, 3 plys, anti fog (AEROKYN, LCH medical products, Paris, France) Children 5 to 10: face mask KC47127, (Kimberly‐Clark, Dallas, TX, USA) Closed plastic bags for disposal	Masks given immediately on home visit by attending general practitioner with demonstration of proper use and instruction to be worn for 5 days in presence of another household member or in confined space (e.g. car) and to change every 3 hours or if damaged.	General practitioners	Face‐to‐face individually	Households in France	One‐off provision of masks worn for 5 days	None described.	None described.	Not described, but reported mask usage was measured	34/51 (66%) wore masks > 80% of the duration. Reported mask‐wearing: 11 ± 7.2 masks during 4.0 ± 1.6 days with an average use of 2.5 ± 1.3 masks per day and duration of use of 3.7 ± 2.7 hours/day
Jacobs 2009	Face masks	Hospital healthcare providers (nurses, doctors, and co‐medical personnel)	Decrease risk of infection through limiting droplet spread through masks	Hospital‐standard disposable surgical Mask MA‐3 (Ozu Sangyo, Tokyo, Japan); quantity not specified	Provision of masks and instructions for use	Not described, presumably research team	Face‐to‐face	Tertiary care hospital in Tokyo, Japan Face masks worn whilst on hospital property.	77 days	None described.	None described.	Self‐reported adherence	Self‐reported adherence for both groups reported as good, with full adherence by 84.3% and remainder complying 79.2% to 98.7%.
Loeb 2009	2 active interventions A. surgical masks B. N95 respirators	Healthcare workers (nurses)	Reduce transmission of influenza in healthcare settings through coughing or sneezing with protective masks	A. Surgical masks B. N95 respirators	Provision of masks or N95 respirators Instruction in use and proper placement of devices Fit‐testing and demonstration of positioning of N95 using standard protocol and procedure (details provided) Qualitative fit‐testing using saccharin or Bitrex protocol^[7]^	Provided by research team (not further described) Fit‐testing by technician for N95	In‐person face‐to‐face	Tertiary hospitals in Ontario, Canada	1 influenza season (12 weeks) Use of mask as required^[8]^ when providing care to or within 1 m of patient with febrile respiratory illness, ≥ 38 °C, and new or worsening cough or shortness of breath Nurses to wear N95 when caring for patients with “febrile respiratory illness”	Fit‐testing of nurses not already fit‐tested	Ceased before end of season	Adherence audits during peak of season by trained auditor who stood short distance from patient isolation room	18 episodes: N95: 6/7 participants (85.7%) wearing assigned device versus 100% for masks
MacIntyre 2009	2 active interventions in addition to infection control guidelines A. Surgical masks (SM) B. P2 masks (P2)	Householders with a child with fever and respiratory symptoms	Prevent or reduce respiratory virus transmission in the community through non‐pharmaceutical interventions	A. 3M surgical mask, catalogue no. 1820; St Paul, MN, USA for adults B. P2 masks (3M flat‐fold P2 mask, catalogue no. 9320; Bracknell, Berkshire, UK) A and B: health guidelines and pamphlets about infection control	Provision of masks and pamphlets and education about infection prevention and mask use Telephone calls and exit interviews to record adherence to mask use All groups: health guidelines, pamphlets about infection control were provided	Not described, presumably research team	Face‐to‐face and by telephone	Households in Sydney, Australia	2 winter seasons (3 months and 6 months) 2 weeks of follow‐up Masks to be worn at all times when in same room as index child, regardless of distance from child	None described.	None described.	Daily telephone calls to record mask use throughout day Exit interviews about adherence	Reported mask use: Day 1 SM: 36/94 (38%) P2: 42/92 (46%) stated wearing “most or all” of the time. Other participants were wearing face masks rarely or never. Day 5: SM: 29/94 (31%) P2: 23/92 (25%)
MacIntyre 2011	3 active interventions A. Medical masks B. N95 respirators fit‐tested C. N95 respirators non‐fit‐tested	Healthcare workers	Protect HCWs by preventing transmission of influenza and other respiratory viruses from patients through mask wearing	Daily supply of A. 3 medical masks (3M medical mask, catalogue number 1820, St Paul, MN, USA) 2 respirators: B. N95 fit‐tested mask (3M flat‐fold N95 respirator, catalogue number 9132) fit‐tested with 3M FT‐30 Bitrex Fit Test kit according to manufacturer's instructions (3M, St Paul, MN, USA) C. N95 non‐fit‐tested mask (3M flat‐fold N95 respirator, catalogue number 9132) Diary cards for usage recording	Supply of masks or respirators. Instruction in when to wear it, correct fitting, and storage (in paper bag in personal locker) Instruction in importance of hand hygiene before and after removal For fit‐tested group: fit‐testing procedure	Masks provided to hospitals. Training of staff provided by 1 member of research team.	Masks and training provided face‐to‐face, not described if training was individually or in groups.	Emergency departments and respiratory wards in hospitals in Beijing, China	Entire work shift for 4 weeks	Taken off for toilet and meal breaks and at end of shift	None described.	Mask ⁄ respirator use monitored by: (i) observed adherence by head ward nurse recorded daily; (ii) self‐report diary cards carried during day recording; (i) no. hours; (ii) usage. Exit interviews	Adherence for usage was high for all and not significantly different amongst arms. Medical mask: 76%, 5 hours N95 fit‐tested: 74%, 5.2 hours N95 non‐fit‐tested: 68%, 4.9 hours
MacIntyre 2013	3 active interventions A. N95 respirators at all times B. N95 respirators targeted use C. Medical masks	Healthcare workers (nurses and doctors)	Protect HCWs from respiratory infections from patients through mask use	Daily supply of: A. and B. 2 respirators (3M Health Care N95 Particulate Respirator; catalogue number 1860) 3M FT‐30 Bitrex Fit Test Kit C. 3 masks 3 masks (3M Standard Tie‐On Surgical Mask catalogue number mask 1817; 3M, St Paul, MN, USA) Pocket‐sized diary card with tick boxes for mask use	Supply of respirators Instructions in use including times and fit Fit‐testing procedure according to the manufacturer’s instructions (3M) For targeted N95: checklist of defined high‐risk procedures, including common aerosol‐generating procedures	3M supplied respirators and masks. Provider of instructions not specified.	Masks and training provided face‐to‐face, not described if training was individually or in groups.	Emergency departments and respiratory wards of tertiary hospitals in Beijing, China	For 4 weeks, A and B worn at all times on shift; B. targeted (intermittent) use of N95 respirators only whilst performing high‐risk procedures or barrier.	None described.	None described.	Self‐reported daily record of number of hours worked, mask or respirator use, number of high‐risk procedures undertaken collected by study staff.	Adherence highest for targeted N95 (82%; 422/516) versus N95 (57%; 333/581) versus medical mask (66%; 380/572).
MacIntyre 2015	2 active interventions A. Cloth masks B. Medical masks	Hospital healthcare workers	Prevent respiratory infections in HCWs from patients through mask‐wearing	A. 5 cloth masks for study duration (2‐ layer, cotton) B. 2 medical masks daily for each 8‐hour shift for study duration (3 layers, non‐woven material) All masks locally manufactured. Written instructions on cleaning cloth masks	Cloth or medical masks to be worn at all times on shift. Cloth masks to be washed with soap and water daily after shifts, and the process of cleaning to be documented. Provision of written instructions for cloth mask cleaning	Researchers arranged supply of masks and instructions and any training of staff assisting the delivery.	Masks and written instructions provided face‐to‐face.	Hospital wards in Vietnam	4 weeks (25 days) of face mask use	Masks not worn while in the toilet or during tea or lunch breaks.	None described.	Monitored adherence with mask use by self‐report diary card and exit survey and interviews with a sub‐sample (ACTRN12610000887077)	Mask‐wearing adherence: cloth mask: 56.8% medical mask: 56.6% Reported cloth mask washing: 23/25 days (92%)
MacIntyre 2016	Medical mask use	Sick householders with ILI (index cases) and their well contacts of the same household	Protect well people in the community from transmission of respiratory pathogens by contacts with ILI through mask use	21 medical masks (3M 1817 surgical mask) Diary cards for mask use	Supply of masks Instructions for mask wearing and hand‐washing protocol Provision of diary cards	Study staff member provided masks and instructions in use.	Masks and instructions provided face‐to‐face and individually.	Fever clinics of major hospitals in Beijing, China	3 masks/day for 21 days Mask wearing: whenever in the same room as a household member or a visitor to the household Hand‐washing: before putting on and after taking off	Allowed to remove their masks during mealtimes and whilst asleep and to cease wearing once symptoms resolved	None reported.	Self‐reported daily record of mask use using diary card	Mask use: mask group: 4.4 hours; control group: 1.4 hours
Radonovich 2019	2 active interventions A. N95 respirators (N95) B. Medical masks (MM)	Healthcare personnel of outpatient sites within medical centres	Prevent HCP from acquiring workplace viral respiratory infections and transmitting them to others by effective respiratory protection by N95 respirators which reduce aerosol exposure and inhalation of small airborne particles, meet filtration requirements, and fit tightly	A. N95 respirators: 3M Corporation 1860, 1860S, and 1870 (St Paul, MN, USA) or Kimberly Clark Technol Fluidshield PFR95‐270, PFR95‐274 (Dallas, TX, USA) B. Medical mask Precept 15320 (Arden, NC, USA) or Kimberly Clark Technol Fluidshield 47107 (Dallas, TX, USA). Reminder signs posted at each site A portable computer equipped with data recording software (HandyAudit; Toronto, Canada) to document adherence (Radonovich 2016)	Participants instructed to wear assigned protective devices whenever they were positioned within 6 feet (1.83 m) of patients with suspected or confirmed respiratory illness and to don a new N95/MM with each patient interaction. Hand hygiene recommended to all participants in accordance with Centers for Disease Control and Prevention guidelines. Infection prevention policies were followed at each study site. Reminder signs posted at sites and emails sent. Annual fit‐testing conducted for all participants. Filtration testing performed on the device models in the study. Further details in protocol (Radonovich 2016).	Centres provided device supplied by study to HCP. Study personnel posted reminder signs and emails and conducted adherence observations.	Face‐to‐face individual provision of devices and adherence observations Onsite posting of signs Other reminders by email	Outpatient sites within medical centres in USA	As instructed, for each new patient interaction during 12‐week period of peak viral respiratory illness each year for 4 years (total of 48 weeks)	Fitting of N95 masks	None described.	Reminder signage posted at study sites, and emails sent by study personnel. Self‐reported daily device wearing of “always”, “sometimes”, “never”, or “did not recall" Observation of device‐wearing behaviours as participants entered and exited care rooms conducted during unannounced, inconspicuous visits to randomly selected sites documented on portable computer	Device wearing: N95: 89.4% reported “always” or “sometimes” versus MM: 90.2% “Never” N95: 10.2% MM: 9.5%
**Hand hygiene**													
Alzaher 2018	Hand hygiene workshop	Primary school girls	Targeted school children to improve hand hygiene to reduce school absences due to upper respiratory infection and spread of infection in schools and to families	6‐minute video‐clip of 2 siblings that attended school‐based health education about hand hygiene Short interactive lecture about: common infections in schools, methods of transmission, hand‐washing procedure using soap and water including when to wash hands Puzzle games related to hand hygiene Posters with cartoon princesses’ picture promoting hand‐washing	Delivery of workshop and distribution of supporting materials (games and posters) to school and students	Study investigator delivered workshop.	Delivered face‐to‐face in group format for the workshop	2 primary girls’ schools in Saudi Arabia	1‐hour once‐off workshop; posters and games provided to school	Not described	Not described	Posters in restrooms as reminders of hand‐washing hygiene during 5‐week follow‐up period after workshop	Not reported
Arbogast 2016	Multimodal hand hygiene intervention programme in addition to control of brief video	Office buildings and the employees of health insurance company	Reduce hand‐to‐mouth germ transmission from shared workspaces and workplace facilities and thereby healthcare claims and absenteeism through improved workplace hand hygiene	Alcohol‐based hand sanitiser (PURELL Advanced, GOJO Industries Inc, Akron, OH, USA) installed as wall‐mounted dispensers, stands, or free‐standing bottles One 8‐ounce bottle of hand sanitiser (PURELL Advanced) per cubicle One 100‐count canister of hand wipes (PURELL Wipes) per cubicle Replenishment products stored in supply room (in addition to existing foam hand wash (GOJO Green Certified Foam Handwash) and an alcohol‐based hand sanitiser foam wall‐mounted dispenser (PURELL, GOJO Industries) already provided near the restroom exits prior to intervention) Identical soap in all restrooms Intervention and control group: brief (< 1‐minute educational video) about proper hand hygiene technique, for both washing and sanitising hands ‘‘Wash Your Hands’’, signage promoting hand hygiene adherence, was already posted next to restroom exits at both the control and intervention sites.	Hand hygiene supplies installed in offices. Replenishment product was made easily available to individual employees upon request via a simple process. Monitoring of product shipments into sites Physical collection and full replacement of soap, sanitiser, and wipes Intervention and control group: educational video embedded at end of baseline online knowledge survey	Not described, presumably study investigators arranged installations	Hand hygiene supplies provided in office environments and individually at staff cubicles/offices. Video provided individually via email.	High‐traffic common areas of 2 US health insurance company offices (e.g. near elevators, at entrances) and appropriate public spaces (e.g. coffee area, break rooms, conference rooms, training rooms, lobbies, reception areas); individual staff cubicles of mostly open plan offices (average 309 square feet). Office restrooms	13.5 months overall One‐off email video 11 days before study hand hygiene supplies installed. 13 months of provision of supplies 2 times evening collection and full replacement of products	Sanitiser installed in high‐use areas of the offices.	Not described	Employee survey at 4 months included questions about hand hygiene practice adherence. Monitoring of product shipments into the sites and physical collection of the soap, sanitiser, and wipes products 2 times in the study; collected samples were measured and usage rates were estimated	Intervention group employees: reported 40% more cleaning of work area regularly; significantly more likely to keep the hand sanitiser with them and use it throughout the day; significant increases in hand sanitiser use for at‐risk activities^[9]^ Estimated use by average employee from sample collection: sanitiser 1.8 to 3.0 times/day, soap 2.1 to 4.4 times/day, wipes at their desk 1.4 to 1.5 times/week
Azor‐Martinez 2016	Hand‐washing programme	Primary school children and their parents and teachers	Prevent transmission of upper respiratory infections in schools and to families through non‐pharmaceutical intervention of hand‐washing programme in schools	Brochure about hand‐washing awareness and habits Workshop content materials Stories, songs, and classroom posters about hand hygiene and infection transmission Hand sanitiser (ALCO ALOE GEL hand sanitiser by Americo Govantes Burguete, S.L. Madrid, Spain containing 0.2% chlorhexidine digluconate, 1% phenoxyethanol, 0.1% benzalkonium chloride, 5% aloe barbadensis, 70% denat ethyl alcohol, excipients quantity sufficient for 100 mL alcohol 70%, pH 7.0 to 7.5) Informational poster about when and how to wash hands Written and verbal guidance to teachers, parents, and students on properties, possible side effects, and precautionary measures for gel use and storage	Brochure sent to parents by mail with study information sheet. Workshop provided for pupils and teachers: frequent infections in schools, transmission and prevention, instructions on correct hand‐washing (water and soap, soaping > 20 s, drying hands), use of hand sanitisers and possible side effects Classroom activities linked to hand hygiene and infection transmission Reinforcement of hand hygiene by teachers Hand sanitiser dispensers fixed to walls with an informational poster about hand‐washing Supervision of younger children when using hand sanitiser and administration of sanitiser if needed Instruction of children in hand‐washing procedures after toilet and when dirty and correct hand sanitiser use^[10]^	Brochure sent by school administration. Workshop and verbal and written information presumably provided by the study research assistant. Classroom activities provided by research assistant and teachers. Supervision and administration of hand sanitiser for younger children by teachers	Brochure sent by mail to individual parents. Workshops and classroom activities delivered in groups face‐to‐face. Teacher reinforcement of hand hygiene provided to class face‐to‐face. Hand sanitiser use supervision was provided individually and face‐to‐face.	Primary school classes in Spain (details not provided)	8 months overall One‐off brochure and installation of hand sanitiser dispensers 2‐hour workshop held 1 month before study commencement Fortnightly classroom activities As required, teacher supervision and administration of hand sanitiser Daily reinforcement of hand hygiene by teachers	Supervision and administration of hand sanitiser as needed by teachers, especially for younger children	Not described	Daily reinforcement by teachers of hand hygiene Fortnightly support by research assistant promoting hand‐washing Self‐reported correct hand‐washing procedure (water and soap, soaping > than 20 s, drying hands)	Self‐reported correct hand‐washing included in analysis but not separately reported.
Azor‐Martinez 2018	Educational and hand hygiene programme 2 active interventions: A. soap and water B. hand sanitiser	Day care centres and their attending children, their parents, and DCC staff	Prevent transmission of respiratory infections by improved hand hygiene of children, parents, and staff through hand‐washing practices and use of hand sanitiser due to its bactericide and virucide properties	A. Liquid soap (no specific antibacterial components (pH = 5.5)) OR B. Hand sanitiser (70% ethyl alcohol (pH = 7.0 to 7.5)) for home use and in dispensers for school classroom Workshop content handout Stories, songs, and posters about hand hygiene and infection transmission	Installation of liquid soap or hand sanitiser dispensers in classrooms Supervision and administration of hand sanitiser if required 3 hand hygiene workshops for parents and DCC staff: 1. Hand‐washing practices, hand sanitiser use, possible side effects and precautionary measures (HSG only) 2. RIs and their treatments 3. Fever Instructions to children, parents, and DCC staff on usual hand‐washing practices and protocol^[11]^ Classroom activities (stories and songs) about hand hygiene and infection transmission	Workshop delivered by researchers. Research assistant provided hand hygiene materials to DCCs and parents. Parents and staff supervised and administered sanitiser where indicated.	Workshops delivered face‐to‐face in groups to parents and staff. Workshop content emailed to attendees individually. Individual face‐to‐face supervision of hand sanitiser use, as indicated	Classroom of DCCs (in Spain) for child interventions Workshops provided at DCCs.	8 months overall Initial 1‐hour workshop 1 month before study commencement 3 further identical sessions/DCC provided again 1 month apart Fortnightly classrooms and DCC activities One‐off installation of dispensers As‐needed supervision of hand sanitiser use Dose of sanitiser: 1 to 2 mL/disinfection	Administration of hand sanitiser in the case of young children DCC staff could attend training at other DCC if unable to attend at own DCC.	Not described	Not described Reported that no monitoring of adherence through continuous observation of hand hygiene behaviours was done, but amount of hand sanitiser was measured	Families or DCC staff, or both, used 1660 L of hand sanitiser, estimated use by each child of dose 6 to 8 times/day.
Biswas 2019	Hand sanitiser and respiratory hygiene education	Primary schools and their students and staff	Reduce community‐wide influenza virus transmission by improving hand‐washing and respiratory hygiene and use of sanitiser in schoolchildren as contributors to community‐wide virus transmission	Hand sanitiser (63% ethyl alcohol) in colourless, transparent 1.5‐litre local plastic bottles (manufactured by a local pharmaceutical company and was available commercially in Bangladesh (price: USD 5.75/L)) Video clip on respiratory hygiene practices Behavioural change materials – 3 colour posters (see Appendix of paper) Curriculum materials for hygiene classes	Installation of hand sanitiser in wall dispensers in all classrooms and outside all toilets, refilled by field staff as needed Encouragement of use of sanitiser at 5 key times during the day^[12]^ Hand and respiratory hygiene education provided.^[13]^ Integration of hygiene messages into school’s hygiene curriculum Delivery of video clip on respiratory hygiene practice Behaviour change materials distributed and placed around schools. Use of sanitiser by classroom teachers after training Training of selected teachers in consultation with head of school and management committee in key messages Communication of key messages by the selected teachers to other teachers	Selected teachers responsible for dissemination of intervention messages throughout were trained over 2 days in these messages, behaviour change communication, sanitiser use, and practices for preventing spread of respiratory secretions. Classroom teachers conveyed intervention messages during regular hygiene classes. Field staff replaced supplies as needed.	Hand sanitiser and education materials provided to schools. Education provided in classrooms in groups and face‐to‐face.	Primary schools (in Bangladesh) Sanitiser in each classroom and outside toilets Education in classroom	10 weeks Intervention messages conveyed in classrooms 3 times/week.	Refills provided as needed.	Not described	Structured field observation by 2 field staff of 5 hours/school observing hand‐washing and respiratory hygiene behaviours of children at 2 different locations in a classroom or outside Every other day, field staff measured the level of hand sanitiser in the morning and in the afternoon to calculate amount of hand sanitiser used/day/school and enrolled children.	Hand‐washing observed opportunities: IG 604/921 (66%) versus CG 171/802 (21%) Hand sanitiser used in 91% of observed hand‐washing events in intervention schools. Average consumption of hand sanitiser/child/day: 4.3 mL Observation of proper cough or sneeze etiquette: IG: 33% versus CG: 2%
Correa 2012	Alcohol‐based hand rubs	Childcare centres and their staff and children	Reduce incidence and transmission of infection in children by improved hand hygiene where water is scarce including provision of ABH and training in hand hygiene teaching techniques	Dispensers of alcohol‐based hand rubs with ethanol 62.0% (PURELL, GOJO Industries, Akron, OH, USA) Workshop materials^[14]^ Visual reminders on ABH techniques in bathrooms and next to dispensers	ABH and training on proper use to staff and children Pre‐trial ABH use workshop to teachers that followed recommended HH teaching techniques and instructed teachers to add ABH to routine HH and give preference to hand‐washing with soap and water if hands visibly soiled Continuous refilling of ABH ABH technique refresher workshops (8/centre) Monitoring of safety, proper use of ABH, amount of ABH used	Local representative of GOJO Industries Inc. provided dispensers and dispenser installations free of charge. Fieldwork team delivered other components.	Face‐to‐face training and provision of materials; group training	Childcare centres in Colombia (centres or community homes) ABH in centres, classrooms, and common areas depending on size Visual reminders in bathrooms and next to dispensers Workshops and training presumably provided in centres.	8 months overall 1 ABH dispenser per centre with < 14 children; 1 per classroom in larger centres; 1 per classroom + 1 for common areas in centres with > 28 children 1 workshop pre‐trial to staff Monthly 30‐minute ABH technique refresher training (8 per centre) Biweekly monitoring	Refilled ABH as needed	Not described	Visual reminders and monthly refresher training Monitoring of safety, proper use of ABH, amount of ABH used Semi‐structured survey on completion of teachers' perceptions about changes in HH practices and use of HSW and ABH. Measurement of consumption of resources and costs related to ABH use and HSW	Teachers at 7 intervention centres reported almost complete substitution of HSW with ABH, and HSW decreased from 3 times per day to 1 per day, and ABH rose to 6 per day. Teachers at remaining 14 centres reported partial substitution of HSW with ABH. Controls reported HSW 3 times per day. Median number of ABH applications per child rose from 3.5 to 4.5 in preschools and 3.5 to 5.5 in community centres.
DiVita 2011	Household hand‐washing promotion	Householders with index patient with ILI	Prevent influenza transmission in households in resource‐poor settings through provision of hand‐washing facilities and use of them at critical times for pathogen transmission	Hand‐washing stations with soap	Provision of hand‐washing stations Hand‐washing motivation to wash at critical times for pathogen transmission (e.g. after coughing or sneezing)	Not specifically described, presumably the researchers	Face‐to‐face provision of facilities in households "Motivation" not described	Household in Bangladesh	Over 2 influenza seasons One‐off provision of hand‐washing facilities Frequency of “motivation” not described	Not described	Not described	Not described	Not described
Feldman 2016	2 active interventions A. Hand disinfection with chlorhexidine gluconate + hygiene education B. Hygiene education	Naval ships and their sailors	Reduced infection transmission and improved hand hygiene in sailors who are at increased risk due to closed environments, contact with shared surfaces, and poor HH culture	Septadine solution (Floris, Misgav, Israel) 70% alcohol and 0.5% CHG; inactive materials: purified water, glycerin, propylene glycol, and methylene blue	Installation of CHG disinfection devices on ships alongside regular soap and water Supply and replenishment of CHG (sent to ships regardless of replenishment demands) Hygiene instruction by a naval physician (to both intervention groups and study control group)	Provision of CHG presumably by study team and funds Hygiene instruction by naval physician	CHG sent to ships directly. Mode of hygiene instruction not described.	Navy fast missile boats and patrol boats of naval base in Israel Dispensers installed in key locations onboard (adjacent to heads (toilets), mess decks (dining rooms), common areas).	4 months Unlimited supply of CHG replenished on demand for 4 to 5 months. Automatic amount dispensed: 3 mL	CHG replenished on demand.	Not described	Total amount of CHG dispensed was tallied.	Mean volume CHG: 8.2 mL per sailor per day (projected yearly cost USD 45 per sailor)
Gwaltney 1980	A. Virucidal hand preparation B. Placebo (no control)	Healthy young adults	Reduce infection rates by interrupting viral spread by hand or self‐inoculation route	A. Virucidal hand preparation: aqueous iodine (2% iodine and 4% potassium iodide) B. Placebo: aqueous solution of food colours (Kroger; Kroger Co., Cincinnati, OH, USA) mixed to resemble the colour of iodine with 0.01% iodine and 0.02% potassium iodide to give an odour of iodine Masks	Immersion of each finger and thumb of both hands to proximal interphalangeal joint (interphalangeal joint of thumb) into designated preparation for 5 seconds then air‐dried for 5 to 6 min Exposure of recipients to donors either immediately after treatment or after 2‐hour delay by hand contact with donor stroking fingers for 10 s Masks worn by donors and recipients during procedure. Recipients placed in single isolation rooms after second exposure till end of experiment.	Researchers	Face‐to‐face and individually	US university	Exposure to donors on 3 consecutive days (days 2, 3, and 4) after initial exposure	Not described	Not described	Reported knowledge of hand preparation use as active, placebo, or don't know	Active (n = 24): 6 active 2 placebo 16 don't know Placebo (n = 22): 6 active 7 placebo 9 don't know
Hubner 2010	Alcoholic hand disinfection	Employees (administrative officers)	Reduce absenteeism and spread of infection in administration employees with frequent customer contact and work with paper documents through improved hand hygiene	2 alcohol‐based hand rubs (500 mL bottles) for desktop use to ensure minimal effort for use: 1. Amphisept E (Bode Chemie, Hamburg, Germany) ethanol (80% w/w) based formula with antibacterial, antifungal, and limited virus inactivating activity. 2. For participants with skin problems: Sterillium (Bode Chemie, Hamburg, Germany) 2‐propanol (45% w/w), 1‐propanol (30% w/w), and mecetronium etilsulfate (0.2% w/w), with a refatting effect and has activity against bacteria, fungi and enveloped viruses. Hand cream: Baktolan balm, water‐in‐oil emulsion with no non‐antibacterial properties (Bode Chemie, Hamburg, Germany)	Provision of hand rub and instruction on use as needed at work only and in accordance with prevailing standard^[15]^: at least 5 times per day, especially after toileting, blowing nose, before eating, and after contact with ill colleagues, customers, and archive material	Presumably provided or arranged by study team	In person to staff	Administration offices in Germany Hand rubs used at desk or work (not outside of work).	12 months overall Hand rub used as much needed for complete wetting of the hands (at least 3 mL or a palmful)^[16]^ at least 5 times per day.	Hand rub use especially after toileting, blowing nose, before eating, and after contact with ill colleagues, customers, and archive material	Not described	Self‐reported adherence with hand hygiene measures	Reported mean hand disinfection frequency times per day: > 5: 19% 3 to 5: 59.8% 1 to 2: 20.5% < 1: 0.7%
Ladegaard 1999 (translated from Danish)	Hand hygiene programme	Daycare centres and their staff, children, and parents of children	Reduce risk of infection in child care through increased hygienic education of daycare professionals, motivation of daycare facilities for regular hand hygiene, and informing parents about hand hygiene	Personnel guide on recommendations for: hygiene, ventilation, out‐of‐stay care, stricter hygienic regulations in cases with selected diseases Fairy tale and poster “The Princess Who Won't Wash Hands” Colouring in drawings “Wash hands” song and rhymes T‐shirt for children with the inscription “Clean hands ‐ yes thank you” Diploma for children and book “The Princess Who Won't Wash Hands” to also be used by parents with their child Informational leaflet for parents in envelope	Staff meeting in each DCC and training in microbiological cause of infection spread guided by National Board of Health and Hygiene Education of children in hand‐washing (about bacteria and why and when to wash hands) Practical hand‐washing classes with 4 to 5 children at a time Provision of t‐shirt, book, and diploma to children Provision of leaflet for parents	Research team presumably provided training.	Face‐to‐face with training and activities by group with staff and children Information sent home to parents via children.	Onsite in DCCs	2‐month intervention period 1‐hour training of children	None described.	None described.	None described.	None reported.
Little 2015	Web‐based hand‐washing intervention	Householders (over 18) who were general practice patients	Prevent transmission of respiratory tract infections through improved hand hygiene to reduce spread via close contact (via droplets) and hand‐to‐face contact	Website‐based programme: provided information about the importance of influenza and role of hand‐washing; developed a plan to maximise intention formation for hand‐washing; reinforced helpful attitudes and norms; addressed negative beliefs (URL provided for demonstration version no longer active; see www.lifeguideonline.org)	Provision of link to website for direct log in Automated emails prompted participants to use sessions and complete monthly questionnaires and maintain hand‐washing.	Researchers delivered web‐based programme and emails.	Online individually	Households in England	4 months overall 4 weekly web‐based sessions Monthly email questions to maintain hand‐washing over 4 months	Tailored feedback provided within web programme	None described.	Emailed questions monthly to maintain hand‐washing	None reported.
Luby 2005	Hand‐washing promotion at neighbourhood level with 2 interventions at household level A. Antibacterial soap B. Plain soap	Neighbourhoods and their households	Improve hand‐washing and bathing with soap in settings where communicable diseases are leading causes of childhood morbidity and mortality	Slide shows, videotapes, and pamphlets illustrating health problems from contaminated hands and specific hand‐washing instructions Soaps: 90‐gram white bars without brand names or symbols, same smell with identical generic white wrappers with serial numbers matched to households A. Households: 2 to 4 white bars of 90‐gram antibacterial soap containing 1.2% triclocarban (Safeguard Bar Soap: Procter & Gamble Company (Cincinnati, OH, USA) B. Households: plain soap (no triclocarban) Soap packets	Hand‐washing promotion to neighbourhoods: Neighbourhood meetings of 10 to 15 householders (mothers) from nearby homes and monthly meetings for men Soap to households Fieldworker home visits: discussed importance of and correct hand‐washing (wet hands, lather them completely with soap, rub them together for 45 seconds, and rinse off completely) technique and promote regular hand‐washing habits^[17]^ Encouragement of daily bathing with soap and water	Research team in collaboration with Health Oriented Preventive Education (HOPE)^[18]^ Fieldworkers were trained in interviewing and hand‐washing promotion.	Face‐to‐face in small groups and individually	Neighbourhoods and homes in Karachi, Pakistan	1‐year weekly household visits 30‐ to 45‐minute neighbourhood meetings 2 to 3 times/week first 2 months then weekly for months 2 to 9, then monthly Monthly men’s meetings first 3 months Weekly household visits	Soap replaced regularly.	None described.	None described, though soap use measured.	Households' mean use of study soap per week: 3.3 bars Average use per resident per day: 4.4 g
Millar 2016 additional details from Ellis 2010	Skin and soft‐tissue infection prevention intervention in addition to SSTI brief on entry also provided to control A. Enhanced standard B. Chlorhexidine	Military trainees	Improve personal hygiene practices to prevent infection, especially acute respiratory infection in military trainees who are at increased risk	A. Enhanced standard: supplemental materials (a pocket card and posters in the barracks) B. CHG: CHG‐based body wash (Hibiclens, Mölnlycke Heath Care, Norcross, GA, USA)	Provision of education and hygiene‐based measures in addition to standard SSTI prevention brief upon entry: Enhanced standard: supplemental materials CHG: as for enhanced standard group, plus a CHG‐based body wash and instructions for use	Not described, presumably the researchers	Face‐to‐face and individually for body wash and pocket card Mode of education not described.	US military training base	One‐off education on entry to training CHG: use of wash 1 per week for entire training period (14 weeks)	None described.	None described.	None described.	None described.
Morton 2004	Healthy hands (alcohol gel as hand‐washing adjunct)	Elementary schools and their children and staff	Prevent infections in elementary school‐age children who are particularly vulnerable through adjunct use of alcohol gel and education based on Health Belief Model (HBM) (Kirscht 1974)	Alcohol gel and dispensers: AlcoSCRUB (60% ethyl alcohol) supplied by Erie Scientific Company, Portsmouth, NH, USA ‘‘Healthy Hands Rules’’ protocol^[19]^ (Figure 3 in paper) Healthy Hand Resource Manual for school nurse, available for parents Monthly newsletters to parents ‘‘Healthy Hands’’ refrigerator magnet for families (see Figure 2 in paper) Informational letter to local primary care providers, paediatricians, family practitioners, and advanced practice nurses “Germ Unit” curriculum and materials including Germ models and Glo Germ	Healthy hands protocol introduced after "Germ unit" education in classes Daily reminders to children on public address system (in first week) then weekly reminders Review of protocol in each classroom after vacation by school nurse 2 classroom visits from school nurse “Healthy Hands” magnet provided to parents and guardians. “Hand Checks on Wednesdays” to identify adverse effects of gel	Gel provided by suppliers. Research team provided educational aspects. Classroom teachers responsible for encouraging use of gel and reinforcing protocol School nurse assisted in monitoring and hand checks for adverse effects.	Face‐to‐face training in classes and individual information giving and monitoring	Elementary schools in USA Wall‐mounted near door entrance of each classroom at age‐appropriate height	46 days 0.5 mL dispensed per application. Use of “special soap” according to “Healthy Hands Protocol” (Figure 3 in paper)	Reinforcement teaching provided if gel usage indicated that it was needed. Germ unit education tailored for each grade level.	1 student was concerned gel was making her sick, so school nurse provided additional classroom visit to allay concerns.	Usage of gel calculated.	5 gel applications per day 1 dispenser lasted 1 month.
Nicholson 2014	Hand‐washing with soap	Households with 5‐year‐olds and their mothers	Targeted 5‐year‐old children and their mothers as change agents to reduce incidence of respiratory infections (and diarrhoeal disease) through hand‐washing using behaviour change principles (Claessen 2008), including social norms for child and mother (Perkins 2003), using fear of contamination and disgust (Curtis 2001), peer pressure (Sidibe 2003), morale boosting, and networking support	Initial supply of 5 bars of free soap (90‐gram Lifebuoy bars) replenished on submission of empty wrappers. Environmental cue reminders (wall hangers, danglers) Rewards (e.g. stickers, coins, toy animals)	Provision of soap and social marketing programme (Sidibe 2009) (Lifebuoy branding) to educate, motivate, and reward children for HWWS at key times Weeks 1 to 17: hand‐washing occasions, germ education, soap’s importance in germ removal Week 18 onward: encouragement of HWWS on 5 key occasions supported by environmental cues "Classrooms" for children Home visits for mothers Parents’ evenings to boost morale, build networks, and run competition for adherence, assignment completion, and folder decoration Establishment of a "Good Mums" club for sharing HWWS tips Rewards provided by mothers. Children encouraged to advocate HWWS within families before meals. Establishment of social norms for child and mother with pledges in front of peers	Dedicated team of "promoters" delivered education and home visits. Mothers provided supplied rewards.	Face‐to‐face in groups Individually by mother to child	"Classrooms" held in community buildings Home visits of households in Mumbai, India	41 weeks Weekly "classrooms" after school and home visits HWWS encouraged 5 key occasions: after defecation, before each of 3 meals, and during bathing. Week 18 onward: hand‐washing on 5 occasions for 10 consecutive days 6 weekly parents’ meetings	Mothers were asked to provide and share hand‐washing tips with other mothers, competitions held for mothers.	Technical difficulties with "soap acceleration sensors" to measure HWWS behaviours prevented successful use.	Registers for "classrooms" and home visits where 3‐week gaps in attendance triggered supervisors to ask participants to resume or be withdrawn Monitoring of soap resale on open market by use of unique identifiers on soap wrappers and twice weekly checks in local shops Collection of used soap wrappers as soap consumption measure	Soap consumption: IG versus CG: 235 g versus 45 g
Pandejpong 2012	3 active interventions (no control) different time‐interval applications of alcohol hand gel A. Every 60 min B. Every 120 min C. Once before lunch	Preschool classes (students and teachers) and their parents	Targeted preschool children who can have high infection rates in ILI; have close interaction so at risk of airborne, droplet, and contact transmission; and are of increasingly younger ages through hand gel as a single strategy of convenient and effective disinfection	1 container of alcohol hand gel per classroom (active ingredients: ethyl alcohol, 70%; chlorhexidine gluconate,1%; Irgasan (triclosan), 0.3%) Cost of hand gel every 60 minutes was USD 6.39 per child per 12‐week period Leaflet describing risk factors for ILI for each family	Teachers instructed to: assist each child with dispensing hand gel at required time interval, store hand gel properly, and refill gel as needed. Monitoring of hand gel use at specified times	Teachers supervised, stored, and refilled hand gel. Instructions to teachers presumably provided by researchers. Leaflets distributed through school. Monitoring of use by 2 research assistants	Face‐to‐face to schools, teachers and children Individual assistance to children with hand gel Leaflets given to each family.	Kindergarten school in Bangkok, Thailand	12 weeks overall 1 pump of gel per child per disinfection round at 1 of 3 time intervals of school day: A. every 60 min B. every 120 min C. once only before lunch, the school standard for hand hygiene	None described.	Students whose families declined to participate were not asked to use alcohol hand gel. These students remained in their classrooms and continued to follow the school standard for hand hygiene.	2 research assistants monitored hand gel use every 60 or 120 minutes for the duration of study. Classroom teachers were required to co‐sign after each disinfection round.	Reported that adherence was ensured for each intervention group Cost of hand gel every 60 minutes was USD 6.39 per child per 12‐week period.
Priest 2014	Hand sanitiser provision (in addition to hand hygiene education session also provided to control group)	Primary schools and their students, teachers, and administrative staff	Reduce person‐to‐person community transmission of infectious disease by targeting improved and additional hand hygiene of school children through supervised hand sanitiser provision as an alternative to improving and maintaining bathroom facilities	‘‘No touch’’ dispensers (> 60% ethanol) for each classroom that dispensed dose when hands were placed under an infrared sensor Supply of top‐up sanitiser as needed	Dispensers installed into each classroom. Teachers asked to ensure that the children used sanitiser at particular times and to oversee general use (McKenzie 2010). Weekly classroom visits to top‐up of sanitiser and measure quantity used 30‐minute in‐class hand hygiene education session provided (also to control group) plus instruction in hand sanitiser use.	School liaison research assistants topped‐up sanitiser. Teachers	Installation of dispensers to classrooms Supervision of children by teachers delivered face‐to‐face individually and as a class.	City schools in New Zealand	20 weeks (2 school terms) Sanitiser to be used by students at least after coughing/sneezing, blowing their nose, and as they leave for morning break and for lunch break. Approximately 0.45 mL of sanitiser dispensed per wash. Weekly top‐up of sanitiser	Children were able to use the sanitiser at any time they wished as well as at key times (McKenzie 2010).	Change of sanitiser after week 10 to flavourless type of the same % ethanol in 41 of 396 classrooms (10%) (in 9 of 34 schools) due to children tasting it when eating, affecting use.	Weekly classroom visits by school liaison research assistants who recorded quantity of sanitiser used Total amount of sanitiser per classroom was measured. adherence defined as dispensing a volume equivalent to at least 45 mL per child of hand sanitiser solution over the trial period.	100% dispensing 45 mL per child Average hand sanitiser dispensed/child for 34 schools: 94 mL Median classroom difference in sanitiser usage between first 10 weeks and second 10 weeks amongst classes that switched products was 220 mL.
Ram 2015	Soap and intensive hand‐washing promotion	Household compounds and its householders (adults and children) that had a householder with ILI	Reduce household transmission of ILI and influenza by promoting hand‐washing in households with householder with ILI as other householders who are well are at highest risk of exposure due to crowded and poorly ventilated homes. Followed constructs of Social Cognitive Theory and the Health Belief Model (Glanz 2008) and behaviour change communication using social marketing concepts	Hand‐washing station in central location of each compound using: large water container with a tap; plastic case for soap; bar of soap. Cue cards depicting critical times for hand‐washing: after coughing or sneezing; after cleaning one’s nose or child’s nose, after defecation; after clearing a child who has defecated; before food preparation or serving; before eating.	Hand‐washing station in each compound Didactic and interactive group‐level education and skills training describing influenza symptoms, transmission, and prevention, promoting health and non‐health benefits of hand‐washing with soap and identification of barriers and proposed solutions to hand‐washing with soap Daily surveillance including weighing of soap and replacing if ≥ 20 g and resupply of water in container if needed Posting of cue cards Asking householders to demonstrate hand‐washing with soap technique	Intervention staff arranged provision of hand‐washing station and presumably provided education. Intervention staff conducted daily surveillance and reinforcement visits.	All elements delivered face‐to‐face but at compound (facilities), group (education), and individual levels (reinforcement).	Household compounds in a rural area of Bangladesh consisting of several households with common courtyard, shared latrine, water source, and cooking facilities	Initiation of intervention within 18 hours of study enrolment, then daily visits until 10 days following resolution of index case patient’s symptoms Day 1 set up of hand‐washing station	Daily surveillance included observation of individual hand‐washing reinforcement and modelling as needed.	None described.	Daily surveillance of facilities and reinforcement and modelling of hand‐washing behaviours including observed hand‐washing Cue cards in common areas of courtyard Presence or absence of soap during each of first 10 days of surveillance from 180 household compounds Patterns and amount of soap use measured.^[20]^	Soap present for at least 7 days in all compounds and on all 10 days in 133 compounds (74%). Soap and water together were present 7 or more of first 10 days in 99% of compounds, with water and soap observed together on all 10 days in 99 compounds (55%) Soap consumption per capita: median: 2.3 g maximal: 5 g (on Day 7)
Roberts 2000	Education about infection control measures, hand‐washing, and aseptic nose wiping	Childcare centres and their staff and children	Reduce transmission of respiratory infections in childcare centres through improved infection control procedures	GloGerm (GloGerm, Moab, UT, USA) Newsletters to staff Songs and rhymes on hand‐washing Plastic bags (sandwich bags available at supermarkets) to cover hand for nose wiping	Staff training in good health (developed by Kendrick 1994) and practical exercise of hand‐washing with GloGerm Fortnightly visits and newsletter to reinforce training and to communicate techniques Recommended hand‐washing technique as per guidelines of the time^[21]^ and after toileting, before eating, after changing diaper (staff and child), and after wiping nose unless barrier used Teaching of technique to children and wash hands for infants	Training and reinforcement activities provided by 1 of the researchers. Teachers delivered training to children based on their training.	Face‐to‐face in groups for training and classes and individually as needed to children or staff	Childcare centres in Canberra, Australia	8 months overall 3‐hour training in evening or 1‐hour during lunch for new staff after study start Duration of hand‐washing: “count to 10” to wash and “count to 10” to rinse	Training for new staff provided as needed.	None described.	6‐weekly adherence measured by recorded observation of recommended practice for 3 hours in the morning in each centre, graded by quantiles of frequency of recommended hand‐washing by children.	Adherence was reported only in relation to analysis of outcomes. High adherence reported for nose wiping and child hand‐washing.
Sandora 2005	Healthy Hands Healthy Families	Families with an index child in out‐of‐home childcare	Reduce illness transmission in the home through multifactorial campaign centred on hand hygiene education and hand sanitiser	Alcohol‐based hand sanitiser: active ingredient: 62% ethyl alcohol (PURELL Instant Hand Sanitiser; GOJO Industries, Inc, Akron, OH, USA) Hand hygiene educational materials at home (fact sheets, toys, games)	Supply of hand sanitiser and hand hygiene materials Biweekly telephone calls Biweekly educational materials	Study investigator	Not stated whether materials mailed or delivered in person	Homes in USA Sanitiser use in home	5 months overall Biweekly educational materials Sanitiser dispensed 1 mL each pump.	None described.	None described.	Recorded amount of hand sanitiser used (as reported by the primary caregiver)	Median frequency of reported times of hand sanitiser use: 5.2 per day 38% used > 2 ounces of hand sanitiser per fortnight = 4 to 5 uses per day
Savolainen‐Kopra 2012 further details from Savolainen‐Kopra 2010	STOPFLU Enhanced hygiene 2 active interventions IR1. Soap and water wash IR2. Alcohol‐based hand rub	Office workers of office work units	Prevent transmission of respiratory infections in workplaces through enhanced hand hygiene with behavioural recommendations to reduce transmission by droplets during coughing or sneezing	IR1: Liquid hand soap (“Erisan Nonsid” by Farmos Inc., Turku, Finland) IR2: in addition: Alcohol‐based hand rub, 80% ethanol (“LV” by Berner Inc., Helsinki, Finland) Bottles of hand hygiene product (free of charge) to be used at home and in the office (IR2). Written instructions on hygiene for further reference	Toilets equipped with liquid hand soap (all groups) or alcohol‐based hand rub (IR2). Guidance on other ways to limit transmission of infections, e.g. frequent hand‐washing in office and at home, coughing, sneezing into disposable handkerchief or sleeve, avoiding hand‐shaking Visits to work clusters and monitoring of materials availability Monthly electronic “information spot” about viral diseases for motivation to maintain hygiene habits Adherence activities	In collaboration with occupational health clinics servicing the corporation Specially trained research nurse provided guidance and visited worker clusters throughout intervention period.	In‐person provision of soap or hand rub Guidance and written instructions given personally. Face‐to‐face visits by study nurse	Office work units in corporations in Helsinki, Finland	15 to 16 months overall Monthly visits by nurse throughout	Nurses assisted with any practical problems with intervention as they arose. New employees received guidance on hand hygiene and habits.	None described.	Adherence assessed by an electronic self‐report survey of transmission‐limiting habits 3 times (more details in protocol). Use of soap (IR1) and alcohol‐based disinfectant (IR2) for personal use was recorded. Study nurse checked availability of soap and alcohol rub.	Avoiding hand‐shaking became more common and remained high in both groups. Recorded use for personal use smaller than predicted use based on hand hygiene instructions. Soap or disinfectant usage per participant: IR1: 6.1 IR2: 6.9
Stebbins 2011	“WHACK the Flu” (hand sanitiser and training in hand and respiratory hygiene)	Elementary schools and their students and homeroom teachers	Targeted school‐aged children as important sources of influenza transmission through improved cough etiquette and hand hygiene in schools including sanitiser as potential inexpensive non‐pharmaceutical interventions	Hand sanitiser dispensers with 62% alcohol‐based hand sanitiser from PURELL (GOJO Industries, Inc, Akron, OH, USA) automatically dispensing 1 dose	Delivery of grade‐specific presentations on “WHACK the Flu” concepts and proper hand‐washing technique and sanitiser use: (W)ash or sanitise your hands often; (H)ome is where you stay when you are sick; (A)void touching your eyes, nose and mouth; (C)over your coughs and sneezes; and (Keep your distance from sick people (provided URL no longer active) Desired frequency of hand wash use taught to student (see When and how much) Installation of hand sanitiser dispensers Refresher training at each school Reinforcement of message and monitoring of sanitiser	Project staff provided education. Home room teachers reinforced message and monitored proper use of sanitiser.	Face‐to‐face at schools, presumably as a group in classes	Elementary schools (Pittsburgh, USA) Dispensers installed in each classroom and all major common areas.	Whole intervention over 1 influenza season One‐off installation of hand sanitiser dispensers One‐off 45‐minute education presentation and one‐off refresher training at onset of influenza season Goal of use of 1 dose (0.6 mL) of sanitiser 4 times per day^[22]^	Encouraged to wash hands or use additional doses of hand sanitiser, or both, as needed	None reported.	Monthly teacher surveys of observed NPI‐related behaviour in their students before, during, and after influenza season Measurement of hand sanitiser use at 2‐week intervals throughout the intervention period	Teacher surveys of observed classroom NPI behaviour indicated successful adoption and maintenance of behaviours throughout influenza season. Average sanitiser use: 2.4 times per day
Talaat 2011	Intensive hand hygiene campaign	Schools and their students, teachers, and parents	Reduce or prevent transmission of influenza viruses amongst children through intensive hand hygiene intervention campaign	Soap supplied as needed. Grade‐specific student booklets each including a set of 12 games and fun activities that promoted hand‐washing Hand hygiene activities materials including: games (e.g. how to escape from the germs); puzzles; soap activities (e.g. soap drawing); song specially developed to promote hand hygiene Teachers’ guidebook including detailed description of the students’ activities and methods to encourage students to practice these activities. Posters with messages to wash hands with soap and water upon arriving at school, before and after meals, after using the bathroom, and after coughing or sneezing. Informational flyers for parents reinforcing the messages delivered at the schools.	Establishment of a hand hygiene team in each school Provision of hand hygiene activities: weekly exercises (e.g. games, aerobics, songs, experiments); school activities, (e.g. obligatory hand‐washing under supervision, morning broadcast, parent meetings, students‐parents information transfer); specific school initiatives: (e.g. competitions and awards, hand‐washing committee, school trips to soap factory and water purification plant) More details in Table 1 of paper Song played regularly. Social worker weekly visits Distribution of flyers to parents	Hand hygiene team (3 teachers from social studies, arts, and sports and the school nurse) ensured that all pre‐designed activities for the hand hygiene campaign were implemented. 6 independent social workers visited the schools.	Delivered face‐to‐face in groups and individually	Elementary schools (grades 1 to 3) in Cairo, Egypt In school environment and classrooms Poster near sinks in classrooms and on playground	12 weeks overall Weekly hand hygiene campaign activities Weekly visits by social workers Twice‐daily obligatory supervised hand‐washing required by students for about 45 seconds, followed by proper rinsing and drying with a clean cloth towel.	Soap and hand‐drying material provided by school administration if children did not bring their own as was the custom or families could not afford it. Schools could create own motivating activities such as selecting a weekly hand hygiene champion, developing theatre plays, and launching school contests for drawings and songs.	None described.	Observation by social workers of hand hygiene activities, availability of soap and drying material, and students’ hand‐washing during the day Schools created own activities to improve adherence.	About 93% of the students had soap and drying material available. All but 2 intervention schools “had a rigorous system of ensuring that schoolchildren were washing their hands at least twice daily”.
Teesing 2021 (additional sources: Teesing 2020a and Teesing 2020b)	HANDSOME multimodal nursing home HH adherence intervention	NH management staff and nurses and nursing students (with or of 3 or 4‐year nursing degree) and residents	Change hygiene policy and individual HH behaviour of nurses through multimodal intervention designed specifically for nursing homes based on literature, interviews at nursing homes and intervention mapping principles, the principle of repetition and informal discussions with members of over 20 nursing home organisations in an iterative process See protocol for more details of intervention mapping process using determinants and methods to develop strategies for intervention components	Materials for lessons about WHO‐defined 5 moments for HH^[23]^ using HANDSOME novel method: ‘Room In’ (moment 1), ‘Room Out’ (moments 4 and 5 combined), ‘Before Clean’ (moment 2), and ‘After Dirty’ (moment 3)^[24]^ Nurse’s watches and certificates earned on completion of e‐learning Paint for washing hands exercise 28 stickers representing barriers to HH in 4 themes (facilities, forgetting, choosing not to do HH, and the telephone) E‐learning materials including videos modelling knowledge, guided practice and promotion of active learning 10 posters (multiple copies, new one each month) Prize for photo competition NH certificate of good HH Small bottle of hand sanitiser for lesson participants See website (www.zorgvoorbeter.nl/hygiene/handhygiene-verbeteren-verpleeghuis) for materials (in Dutch) used for intervention:^[25]^ ‐ Manual (84p) ‐ E‐learning module ‐ PowerPoint presentation and script ‐ Assignments ‐ Awareness activities ‐ Audit materials ‐ Policy materials ‐ Posters Adherence recording application and computer table Adherence observer training materials using method adapted from a study in Dutch hospital^[26]^: videos and case studies and examination using videos from Hand Hygiene Australia^[27]^ [1] World Health Organization. (‎2012)‎. Hand hygiene in outpatient and home‐based care and long‐term care facilities: a guide to the application of the WHO multimodal hand hygiene improvement strategy and the “My Five Moments For Hand Hygiene” approach. World Health Organization. apps.who.int/iris/handle/10665/78060 (accessed 15 June 2022) [2] Moment 1 (before touching a resident) = Room In; Moment 4 (after touching a resident) and Moment 5 (after touching a resident’s surroundings) = Room Out; Moment 2 (before a clean/antiseptic procedure) = Before Clean; Moment 3 (after body fluid exposure risk) – After Dirty [3] Handsome: handhygiëne in verpleeghuizen.: Zorg voor beter; 2019 May 03. URL: www.zorgvoorbeter.nl/handsome (accessed 7 June 2022) [4] Veiligheid en Kwaliteit: Project Handen uit de Mouwen.: Stichting Samenwerkende Rijnmond Ziekenhuizen [5] Auditor training.: Hand Hygiene Australia URL: www.hha.org.au/audits/auditor-training (accessed 7 June 2022)	See Table 1 of Teesing 2020a and Teesing 2020b for more details 1. Policy change: ‐ management meeting (with senior nursing home manager, infection prevention specialist, and facilities manager), ‐ personal hygiene rules ‐ HH materials audit 2. Nursing staff interventions (The New Way of Working) i) 3 live lessons: a. introduction of HANDSOME/WHO HH moments; teaching and discussion re HH when handling medication, food, laundry; when to use hand sanitiser/soap/gloves. Team HH goal‐setting; b. make inventory and solutions for barriers to HH adherence; and c. exercise washing hands with paint to see where missed; teaching how to disinfect hands ii) e‐learning: introduction and 7 lessons showing: ‐ correct/incorrect HH behaviour ‐ common HH actions ‐ when to use gloves ‐ food and medication preparation Quizzes: iii) reminder posters hung throughout NH showing large picture of hands and text: “Did you remember to wash your hands?” (in Dutch’) iv) photo competition: prize for best photo of hands 3. Arts and craft project for residents involving hands that NH displays Adherence recording procedures Provision of hand sanitiser to lesson participants Provision of good HH certificate to NH if higher than average adherence Provision of nurse’s watch on completion of e‐learning Provision of adherence observers training	Meeting and materials provided by researcher Study team member delivered 3 live lessons with involvement of senior NH manager Senior NH managers involved in delivery of aspects, including a lesson on NH personal hygiene policy between lessons 1 and 2 Nurses and doctors in training provided adherence observation and assessment	Face to face in groups (management and nursing staff) Lessons in groups of maximums of 18/session Online individual e‐learning	In residents’ rooms or other areas of 2 units each of 33 Dutch nursing homes with ≥ 3 nurses providing intense psychogeriatric and/ or somatic care to geriatric residents Meetings on‐site Lessons on‐site and online Posters throughout NH	4 months (Jan to Apr 2017) Management meeting (45 to 60 min) Personal hygiene policy presentation (10 min) Live lessons: 1 (20 min) 2 (30 min) 3 (40 min) given multiple times on 1 day E‐learning: 5 to 10 min each Adherence observer training: 2 to 3 days Adherence observation: during observation hours (8 am to 1.30 pm, weekdays)	Persuasive communication used to encourage continuing when NH wanted to stop When < 3 nurses working at the unit, either the observers continued observations at an additional ward (who also received the intervention) or they stopped observing HH needed to happen in the same room as action occurred, except if a nurse brought a resident to another room, they carried something soiled or no door needed to be opened before leaving the room; for these instances, HH should take place at the end of action	None described, except that the process was iterative in response to feedback from individual nursing homes	Unobtrusive HH direct observation disguised as registering of frequency of health care activities recorded on computer tablet (see Figure 2 in Teesing 2020a and Table 3 of Teesing 2020b) Compliance registered if HH occurred immediately before (moments 1 and 2) or after (moments 3, 4 and 5) a HH opportunity without touching another object (e.g. door handle) and only if hand sanitiser or soap, water and paper towel used Hand‐related personal hygiene^[28]^ for each nurse according to Dutch guidelines^[29]^1 / every nurse / day Attendance at live lessons and e‐learning was recorded Participants asked if HH policy information received and if posters seen	HH compliance (12 m f/u) IG: 36% CG: 21% (OR 2.28, CI 1.67 to 3.11) HH compliance increased more for IG than CG for each WHO‐defined moment, except for moment 2 Estimated attendance at lessons: varied per unit: 23% had < 50% attending at least 1 lesson, 18% had 50% to 74% attendance at at least 1 lesson and 59% had > 75% attendance at least 1 lesson (n = 22).
Temime 2018	Multifaceted hand hygiene programme (including alcohol‐based hand rub)	Nursing home staff, residents, visitors, and outside care providers	Nursing homes and their residents, staff, and visitors and external providers have an increased risk of person‐to‐person transmission of pathogens, and HH is a simple and cost‐effective tool for infection control; however, compliance with HH is poor in nursing homes.	Dispensers and pocket‐sized containers of hand rub solution Posters promoting hand hygiene Developed local HH guidelines eLearning module on infection control and HH training with online quizzes requiring sufficient performance	Facilitated access to hand rub solution Campaign to promote HH with posters and event organisation Formation of local work groups in each NH Development of local HH guidelines Staff education using eLearning Monitoring of quantity of hand rub solution used	Same nurse provided HH training for all NHs. Provision of hand rub by NH Local work group developed guideline. eLearning module and posters presumably developed by research team.	Provision of materials face‐to‐face Education and quizzes via eLearning	Nursing homes in France	1 year overall One‐off provision of hand rub One‐off eLearning repeated if unsatisfactory performance.	If staff did not score sufficiently on online quiz, they were invited to repeat the eLearning.	None described.	Estimated mean amount of hand rub solution used per resident per day assessed as proxy for HH frequency, based on quantity of hand rub solution bought by NH (which was routinely monitored in all the NHs).	Hand rub solution used: baseline quantity of consumed hand rub solution was 4.5 mL per resident per day. Over the 1 year, mean quantity consumed was significantly higher in intervention NH (7.9 mL per resident per day) than control (5.7 per resident per day).
Turner 2004a Clinical trial 1	3 active interventions (no control) Product: A. Ethanol B. Salicylic acid C. Salicylic acid with pyroglutamic acid	Healthy volunteers	Assess the residual virucidal activity of organic acids used in currently available over‐the‐counter skin products for the prevention of experimental rhinovirus colds	1.7 mL of hand products: A. 62% ethanol, 1% ammonium lauryl sulphate, and 1% Klucel) B. 3.5% salicylic acid, or vehicle containing C. 1% salicylic acid and 3.5% pyroglutamic acid	Disinfection of hands then application of test product then allowed to dry. 15 min later, fingertips of each hand contaminated with 155 TCID_50_ of rhinovirus type 39 in a volume of 100 μL. Hands air‐dried for 10 min. Intentional attempted inoculation with virus by contact with fingers, conjunctiva, and nasal mucosa with fingers of right hand. Left hand eluted in 2 mL of virus‐collecting broth.	Researchers	Face‐to‐face individually	Communities in Manitoba, Canada	1.7 mL of product applied. See What for timing	Not described	Not described	Not described	Not described
Turner 2004b Clinical trial 2	2 active interventions (no control) Skin cleaner wipe product: A. Pyroglutamic acid B. Ethanol	Healthy volunteers	Assess the residual virucidal activity of organic acids used in currently available over‐the‐counter skin products for the prevention of experimental rhinovirus colds	Skin cleanser wipe containing: A. 4% pyroglutamic acid formulated with 0.1% benzalkonium chloride B. 62% ethanol	Application of product to hands with towelette then allowed to dry. 15 min later, fingertips of each hand contaminated with 106 TCID_50_ of rhinovirus type 39 in a volume of 100 μL. Intentional attempted inoculation with virus by contact with fingers, conjunctiva, and nasal mucosa with fingers of right hand. Left hand eluted in 2 mL of virus‐collecting broth.	Researchers	Face‐to‐face individually	Communities in Manitoba, Canada	Dose not reported; see What for timing Additional group challenged 1 h after application; final group challenged 3 h after application (remained at study site and not allowed to use or wash hands between).	Not described	Not described	Not described	Not described
Turner 2012	Antiviral hand lotion	Healthy adults	Reduce rhinovirus infection and illness through hand disinfection with ethanol and organic acid sanitiser	Lotion containing 62% ethanol, 2% citric acid, and 2% malic acid Daily diary	Provision of lotion and instructions for use Meetings with participants to check compliance	Staff of study site presumably supplied lotion. Study site staff met with participants.	Face‐to‐face and presumably individually, but not specified	Study site at university community in the USA	9 weeks Every 3 hours whilst awake and after hand‐washing for 9 weeks Compliance meetings twice weekly for first 5 weeks then weekly meetings with participants	None reported.	None reported.	Self‐reported daily diary of time of each product application Twice weekly for 5 weeks then weekly meetings with participants to reinforce compliance with treatment	“All subjects … applied at least 90% of the expected amount of hand treatment” (p. 1424)
Yeung 2011	Multifaceted hand hygiene programme (including alcohol‐based hand rub)	Long‐term care facilities and their healthcare workers	Promote use of alcohol‐based hand rub by staff in LTCFs as an effective, timely, and low‐irritant method of hand hygiene in a high‐risk environment	Free supply of pocket‐sized containers of alcohol‐based antiseptic hand rub (either WHO formulation I (80% ethanol) or II (80% propanol) carried by each HCW (supplier: Vickmans Laboratories) Replacement hand rub as required Hand hygiene seminar content Reminder materials (3 to 5 posters and specially designed ballpoint pens)	Provision of materials Provision of hand hygiene seminars to HCWs covering: indications, proper method, and importance of antiseptic hand rubbing and washing according to WHO 2006a) guidelines Provision of feedback session Direct, unobtrusive observation of hand hygiene adherence Training of observation staff	Study team delivered the materials, seminars, and observer training. Administrative staff of LTCF provided replacement hand rub and communicated with HCWs. 6 registered nurses conducted direct observation of adherence after 2‐hour training (100% interrater reliability).	Delivered face‐to‐face and individually for hand rub and pens; not described if education was individually or by group, but seminar implies as a group	LTCFs in Hong Kong Posters posted in common areas. Adherence observations occurred in common rooms and resident rooms but not bathing or toilet areas.	7 months overall Initial 2‐week intervention period, then 7 months of hand rub provision and reminders 3 identical seminars at start of intervention; each staff member to attend once Feedback session 3 months after start of intervention 2‐hour training of observers Adherence observations either 9 am to 12 pm or 3 pm to 6 pm, 1 LTCF at a time	Replacement of hand rub as required	As adherence dropped off in the middle months, the feedback session was delivered.	Direct observation of HCW adherence to hand‐washing and antiseptic hand rubbing (recorded separately and anonymously) during bedside procedures or physical contact with residents 3300 hand hygiene opportunities during 248.5 hours of observation on 92 days	90% attendance of seminars Hand rubbing with gel increased significantly from 1.5% to 15.9%. Hand‐washing decreased significantly from 24.3% to 17.4%. Control: 30% Overall hand‐washing adherence increased from 25.8% to 33.3%.
Zomer 2015	Hand hygiene products and training	Daycare centres and their caregivers (staff)	Reduce infections in children attending DCCs through improved access to HH materials (Zomer 2013a) and compliance of their DCC caregivers to hand hygiene guidelines based on socio‐cognitive and environmental determinants of caregivers’ HH behaviour^[30]^ (Zomer 2013b)	HH products: dispensers for paper towels, soap, alcohol‐based hand sanitiser, and hand cream, with refills for 6 months Reminder posters and stickers for children and DCC caregivers Training materials including booklet	Provision of free HH products sponsored by SCA Hygiene Products, Sweden. Provision of posters and stickers for children and staff Provision of training about RIVM 2011 for mandatory HH^[31]^ Distribution of training booklet Team training sessions aimed at goal‐setting and formulating HH improvement activities (Erasmus 2011; Huis 2013)	Study team arranged supply of HH products and presumably provided training.	Products provided to DCCs in person for staff use. Mode of training not specified.	DCCs in regions of the Netherlands	6 months overall Initial one‐off supply of products 3 training sessions with 1‐month interval 2 team training sessions	Replacement hand hygiene provided as required.	None described.	6‐month follow‐up observation of whether intervention dispensers and posters/stickers in use Survey of DCC caregivers HH guidelines compliance observed at 1, 3, and 6 months' follow‐up: no. of HH actions/no. of opportunities	2 DCCs did not use any HH products. Sanitiser products used in at least 1 of 2 groups in 94%, 89%, 86%, and 45% of intervention DCCs. Posters used in 86%, stickers in 74%. DCC survey results: 79% attended at least 1 training session; 77% received HH guidelines booklet. HH compliance at 6 months: IG: 59% vs CG: 44% (Zomer TP, et al, unpublished data) All intervention DCCs received guidelines training; all but 2 received at least 1 team training.
**Hand hygiene and masks**													
Aelami 2015	Hygienic education and package	Religious pilgrims	Prevent influenza‐like illness by reduced infection transmission through personal hygiene measures	Hygiene package of: alcohol‐based hand rub (gel or spray) surgical masks soap paper handkerchiefs user instructions	Not clearly described, but it appears that packages may have been distributed by trained physicians before departure to or on site of country of pilgrimage	Not specifically described	Not described, but it appears that packages were distributed face‐to‐face and individually	Not described if before departure (from Iran) or on site (in Saudi Arabia)	One‐off during Hajj season	Not described	Not described	Not described	None described
Aiello 2010	2 active interventions: A. Face mask (FM) B. Face mask and hand hygiene (FM + HH)	Students living in university residences	Reduce the incidence of and mitigate ILI by use of non‐pharmaceutical interventions of personal protection measures	7 face masks (standard medical procedure masks with ear loops TECNOL procedure masks; Kimberly‐Clark) 7 re‐sealable plastic bags for mask storage when not in use (e.g. eating) and for disposal Alcohol‐based hand sanitiser (62% ethyl alcohol in a gel base, portable 2‐ounce squeeze bottle, 8‐ounce pump) Hand hygiene education (proper hand hygiene practices and cough etiquette) via emailed video, study website, written materials detailing appropriate hand sanitiser and mask use	Weekly supply of masks through student mailboxes Provision of basic hand hygiene education through an email video link, the study website, and written materials; instruction to wear mask as much as possible; education in correct mask use, change of masks daily, use of provided re‐sealable bags for mask storage and disposal Provision of replacement supplies which students signed for upon receipt	Not described, except education provided via study website (URL not provided) “Trained staff” for compliance monitoring Study‐affiliated residence hall staff provided replacement supplies.	Education via email and study website; provision of masks and sanitiser in person to residences	University residence halls in the USA	One‐off education, 6 weeks (excluding spring break) of face mask and/or hand hygiene measures which commenced at “the beginning of the influenza season just after identification of the first case of influenza on campus” (p.496). Replacement supplies provided as needed.	Mask wearing during sleep optional and encouraged outside of residence.	University spring break occurred during weeks 4 and 5 of the study, with most students leaving campus and travelling; they were not required to continue protective measures at that time.	Weekly web‐based student survey included: self‐reported average number of times hands washed/day and average duration of hand‐washing to obtain composite "optimal handwashing” score (at least 20 s ≥ 5/day); average no. of mask hours/day/week; average hand sanitiser use/day/week and amount used. Trained staff in residence hall common areas observed silently and anonymously improper mask use, instances of hand sanitiser use.	Average mask use hours/day: FM + HH 2.99 versus FM 3.92 Average hand‐washing times/day: FM + HH 6.11 versus FM 8.18 vs control group 8.75 Daily washing seconds/day: FM + HH 20.65 versus FM 23.15 vs control 22.35 Hand sanitiser use times/day: FM + HH: 5.2 versus FM 2.31 vs control 2.02 No. of proper mask wearing participants/hour of observation: FM + HH 2.26 versus FM 1.94
Aiello 2012	2 interventions: A. Face mask (FM) B. Face mask and hand sanitiser (FM + HH)	Students living in university residences	Prevent ILI and laboratory‐confirmed influenza by use of non‐pharmaceutical interventions of personal protection measures (e.g. face masks and hand hygiene)	Packets of 7 standard medical procedure masks with ear loops (TECNOL procedure masks, Kimberly‐Clark, Roswell, GA, USA) and plastic bags for storage during interruptions in mask use (e.g. whilst eating, sleeping) and for daily disposal Hand sanitiser (2‐ounce squeeze bottle, 8‐ounce pump bottle with 62% ethyl alcohol in a gel base) Replacement face masks and hand sanitiser Educational video: proper hand hygiene and use of standard medical procedure face masks	Intervention materials and educational video provided. Supply of masks and instructions on wearing Provision of replacement masks or sanitisers as needed on site	Trained study staff available at tables in each residence hall for surplus masks and sanitiser and for observing compliance	Hygiene packs delivered to student mailboxes; face‐to‐face supply also available	University residence halls in the USA	One‐off educational video at start Weekly supply of hygiene packs Masks to be worn at least 6 hours/day Study staff available onsite with replacement supplies as needed for duration of intervention (6 weeks, excluding spring break)	Students encouraged but not obliged to wear masks outside of residence hall.	1‐week university spring break during the study when majority of students left campus	Weekly student survey including compliance (e.g. masks hours/day, frequency and amount of sanitiser use, number of hand washes/day, duration of hand‐washing (seconds) Observed compliance completed by trained study staff who daily and anonymously observed mask wearing in public areas of residences.	Self‐reported mask wearing: no significant difference Sanitiser use: significantly more in FM + HH than FM or control groups More results in S1 of paper. Staff observed an average of 0.0007 participants properly wearing a mask for each hour of observation.
Cowling 2009	2 active interventions in addition to control of lifestyle education: A. Enhanced hand hygiene (HH) B. Face masks and enhanced hygiene (FM + HH)	Householders with index patient with influenza	Reduce transmission of influenza in households through personal protective measures	A. and B. Liquid soap for each kitchen and bathroom: 221 mL Ivory liquid hand soap (Proctor & Gamble, Cincinnati, OH, USA) Alcohol hand rub in individual small bottles (100 mL) WHO recommended formulation I, 80% ethanol, 1.45% glycerol, and 0.125% hydrogen peroxide (Vickmans Laboratories, Hong Kong, China) B. Adults: box of 50 surgical face masks (Tecnol–The Lite One (Kimberly‐Clark, Roswell, GA, USA) to each household member or C. Children 3 to 7: box of 75 paediatric masks	Home visits Provision of soap, hand rub, and masks as applicable and when to use them HH: education about efficacy of hand hygiene Demonstration of proper hand‐washing and antisepsis techniques + FM: education about efficacy of surgical face masks in reducing disease spread to household contacts if all parties wear masks Demonstration of proper wearing and hygienic disposal All groups: provision of education about the importance of a healthy diet and lifestyle, both in terms of illness prevention (for household contacts) and symptom alleviation (for the index case)	Trained study nurse provided interventions.	Face‐to‐face to householders	Households in Hong Kong	Initial home visit scheduled within 2 days (ideally 12 h) of index case identification. Further home visits day 3 and 6, 7‐day follow‐up HH: use of liquid soap after every washroom visit, sneezing or coughing, when their hands were soiled. Use rub when first returning home and immediately after touching any potentially contaminated surfaces FM: masks worn as often as possible at home (except eating or sleeping) and when the index patient was with the household members outside of the household	Not described	Not described	Monitoring of adherence during home visits Evaluation of adherence on final visit by interview or self‐reported practices and counting of amount of soap and rub left in bottles and remaining masks for FM group	Most initial visits completed within 12 h. Intervention groups “reported higher adherence … than the control group. Self‐reported data were consistent with measurements of amount of soap, alcohol hand rub, and face masks used” (p.443) (see Table 6 in paper). “Adherence to the hand hygiene intervention was slightly higher in the hand hygiene group than the face mask plus hand hygiene group.” Median masks used: Index: 9 Contact: 4 More details in paper and Appendices
Larson 2010	2 active interventions in addition to control of URI education: A. Alcohol‐based hand sanitiser (HS) B. Face masks and hand sanitiser (FM + HS)	Hispanic householders with at least 1 preschool or elementary school child	Reduce incidence and secondary transmission of URIs and influenza through non‐pharmaceutical household level interventions	A. and B. 2‐month supply of hand sanitiser in 8‐, 4‐, and 1‐ounce containers: PURELL (Johnson & Johnson, Morris Plains, NJ, USA) B. 2‐month supply of masks: Procedure Face Masks for adults and children (Kimberly‐Clark, Roswell, GA, USA) Replacement supplies at least once every 2 months Disposable thermometers Educational materials about URI prevention, treatment, and vaccination (written in Spanish or English language)	Provision of materials and instructions for when to use including demonstration of use and observation of return demonstration by householder A. Mask worn when householder had: “temperature of ≥37.8°C and cough and/or sore throat in the absence of a known cause other than influenza” (CDC definition of influenza‐like illness at the time). Home visits to reinforce adherence, replenish supplies and record use, answer questions B. Telephone calls to reinforce mask use All groups received URI educational materials.	4 trained bilingual research assistants (RAs) with minimum baccalaureate degree and experience in community‐based research; procedures were practised with each other until demonstrated proficiency	Face‐to‐face to householders	Households in New York, USA	19‐month follow‐up Initial home visit, then at least every 2 months Sanitiser for use at home, work, and school B. Telephone calls days 1, 3, 6 Masks worn for 7 days when within 3 feet of person with ILL or no symptoms.	Change masks between interactions with person with ILL Householders' questions and misconceptions addressed on home visits.	None described.	RA home visits for adherence with random accompaniment by project manager, who also made random calls to householders Telephone calls to reinforce mask use Used bottles or face masks, or both, monitored for usage.	Sanitiser use (mean ounces/month) HH: 12.1 FM + HH: 11.6 Mask compliance was “poor”: 22/44 (50%) used within 48 hours of onset. Mask users reported mean mask use of 2.
Simmerman 2011	2 active interventions: A. Hand‐washing education and hand‐washing kit (HW) B. Hand‐washing education, hand‐washing kit, and face masks (HW + FM)	Households with a febrile, influenza‐positive child	Decrease influenza virus transmission in household with a febrile influenza‐positive child through promoted use of hand‐washing or hand‐washing with face mask use	A. and B. Hand‐washing kit per household including graduated dispenser with standard unscented liquid hand soap (Teepol brand. Active ingredients: linear alkyl benzene sulfonate, potassium salt, and sodium lauryl ether sulphate) Replacement soap as needed Written materials from education including pamphlets and posters attached near sinks in household. B. Box of 50 standard paper surgical face masks and 20 paediatric face masks (Med‐con company, Thailand #14IN‐20AMB‐30IN)	A. and B. Provision of intensive hand‐washing education on initial home visit to household members with 5 approaches: discussion, individual hand‐washing training, self‐monitoring diary, provision of soap, and provision of written materials (Kaewchana 2012) Individual hand‐washing training ("why to wash", "when to wash", and "how to wash" in 7 hand‐washing steps described in Thailand Ministry of Public Health guidelines) B. Provision of education of benefits of and appropriate face mask wearing Soap replaced as needed. More details (Kaewchana 2012)	Study nurse conducted home visits, provided education and monitoring activities.	Education provided face‐to‐face as a group to household member and individually for hand‐washing training.	In homes (in Bangkok, Thailand)	One‐off provision of kits at initial home visit conducted within 24 hours of enrolment Subsequent home visits on days 3, 7, and 21 90‐day supply of hand‐washing supplies 30‐minute education provided at initial home visit	B. No face masks whilst eating or sleeping as impractical and could hinder breathing in ill child Impromptu education and training provided by nurses as questions arose.	None described.	Self‐monitoring diary recording hand‐washing frequency > 20 s and face mask use for that group Reinforcement of messages by nurses on subsequent home visits Amount of household liquid soap and number of face masks used	Reported average hand‐washing episodes/day: HW: 4.7 HW + FM: 4.9 Parents had highest frequency (5.7), others (4.8), siblings (4.3), index cases (4.1). Average soap used/week: HW: 54 mL/person HW + FM: 58.1 mL/person B. Mask use: 12/person/week Mask wearing median minutes/day: 211 Parents 153, other relations 59, index patients 35, siblings 17
Suess 2012	2 active interventions in addition to written information: A. Mask/hygiene (MH) B. Mask (M)	Households with an influenza‐positive index case in the absence of further respiratory illness within the preceding 14 days	Prevent influenza transmission in households through easily applicable and accessible non‐pharmaceutical interventions such as face masks or hand hygiene measures	A. Alcohol‐based hand rub (Sterilium, Bode Chemie, Germany) A. and B. Surgical face masks in 2 different sizes: children < 14 years (Child’s Face Mask, Kimberly‐Clark, USA) and adults (Aérokyn Masques, LCH Medical Products, France) Written information provided on correct use of intervention and on infection prevention (Suess 2011) (tips and information on the new flu A/H1N1) (URL provided is no longer active) Digital tympanic thermometer General written information on infection prevention	A. Provision of hand rub and masks A. and B. Provision of masks only Provision of thermometer and how to use it Mask fit assessed (at first household visit) Information provided by telephone and written instructions at home visit on proper use of interventions and recommendations to sleep in a different room than the index patient, not to take meals with the index patient, etc. (Suess 2011) In‐person demonstration of interventions at first home visit All participating households received general written information on infection prevention.	Study personnel arranged provision of materials, rang the participants, visited the homes, demonstrated and assessed fit of masks.	Provision of materials in person to households Initial telephone delivery of information Face‐to‐face home visits	Households in Berlin, Germany	Over 2 consecutive flu seasons Day 1 households received all necessary material instructions. Household visits no later than 2 days after symptom onset of the index case, then days 2, 3, 4, 6, 8 (5 times) or on days 3, 4, 6, 8 (4 times) depending on the day of recruitment Hand rub use: after direct contact with the index patient (or other symptomatic household members), after at‐risk activities or contact^[31]^ Mask use: at all times when index patient and/or any other household member with respiratory symptoms were together in 1 room Regular change of face masks, not worn during the night or outside the household	Adult masks worn if masks for under 14‐year‐olds did not fit properly. If other household members developed fever (> 38.0 °C), cough, or sore throat, they were asked to adopt the same preventive behaviour as the index patient.	In the season 2010/11 participants also recorded number of masks used per day.	Self‐reported daily adherence with face masks, i.e. if they wore masks “always”, “mostly”, “sometimes”, or “never” as instructed. Participants of the MH households additionally noted the number of hand disinfections per day. Exit questionnaire about (preventive) behaviour during the past 8 days, general attitudes towards NPI, the actual amount of used intervention materials, and, if applicable, problems with wearing face masks. Used intervention material per household member was calculated by dividing the amount used per household by the number of household members. See paper and Suess 2011 for more details.	Face mask use (median/individual): MH: 12.6 M: 12.9 Daily adherence was good, reaching a plateau of over 50% in nearly all groups from the third day on. MH hand rub use (median): 87 mL (Suess 2011) MH mean frequency of daily hand disinfection: 7.6 (SD 6.4) times per day See paper and Suess 2011 for more results.
**Hand hygiene and surface/object disinfection**													
Ban 2015	Hand hygiene and surface cleaning or disinfection	Kindergartens and the families of their students	Reduce transmission of infection in young children from contaminated surfaces or hands through hand hygiene and surface cleaning or disinfection	Antibacterial products for hand hygiene and surface cleaning or disinfection: liquid antimicrobial soap for hand‐washing (0.2% to 0.3% parachlorometaxylenol). Instant hand sanitiser for hand disinfecting (72% to 75% ethanol), antiseptic germicide (4.5% to 5.5% parachlorometaxylenol, diluting before use). Bleach (4.5% to 5.0% sodium hypochlorite, diluting before use) for surface disinfecting. Produced by Whealthfields Lohmann (Guangzhou) Company Ltd.	Provision of products to kindergartens and families Instruction of parents or guardians and teachers in hand hygiene techniques and use of antibacterial products Daily cleaning of kindergartens with products At least twice/week cleaning of homes and weekly cleaning or disinfecting of items such as children’s toys, house furnishings, frequently touched objects (doorknobs, tables or desks), kitchen surfaces (utensils, cutlery, countertops, chopping boards, sinks, floors, etc.), bathroom surfaces (toilet, sink, floor, etc.) Monitoring activities	Research team provided products and instructions and monitoring.	Materials provided to kindergartens and families in person and presumably instructions in person to families and staff.	In kindergartens (hard surfaces) and families’ homes (Xiantao, China)	1 year overall Daily hand‐washing with soap before eating, after using bathroom, nose blowing, and outdoor activities Hand sanitiser carried daily. Kindergarten cleaning daily Home cleaning at least twice/week	Families and teachers could contact study management at any time as needed. Exchange of empty bottles for new ones at any time	Not described	Close contact with teachers and families for monitoring, e.g. unscheduled parents’ meetings, quarterly home visits, phone interviews, and monthly cell phone messages Monthly survey of consumption of products by volume, total usage, person usage	Consumption of products by person (mL/person/day). Liquid soap: 7.7 Sanitiser: 1.4 Bleach: 25.0 Antiseptic‐germicide: 12.5
Carabin 1999	Hygiene programme	Daycare centres and their staff and children	Reduce infections in at‐risk children (under 3 years old) in DCCs with inexpensive, easily implementable and practical interventions	Hygiene materials and documents, e.g. colouring books, hand‐washing posters, hygiene videotapes Materials for training Reimbursement of equivalent of 1 full‐time educator’s salary Bleach (diluted 1:10) for toy and play area cleaning	Provision of comprehensive hygiene training session to entire DCC staff, especially the educators of participating classrooms Training in recommendations for hygiene practices: i. toy cleaning ii. hand‐washing technique and schedule iii. use of creative reminder cues for hand‐washing iv. open window for daily period v. sandbox and play area cleaning Payment of salary of educator for the day to encourage participation DCC meetings to discuss training session with all staff	Training appears to have been provided by study team.	Appears staff trained as a group, i.e. “entire DCC staff”	Daycare centres in Canada Location of training not described, except may have been off‐site from DCCs since 1 DCC did not “send” staff to training.	15‐month trial One‐off 1‐day training Toy cleaning at least every 2 days Hand‐washing at least after DCC arrival, after outside play, after bathroom, before lunch Open windows at least 30 min/day Biweekly cleaning of sandbox/play area	Teachers to use creative reminder cues for hand‐washing with children	Not described	Follow‐up telephone questionnaire for DCC directors about following training recommendations	Use of materials: colouring book: 22/24 poster: 23/24 videotapes: 18/24 staff meetings: 19/24 Increased frequency of toy cleaning: 6/24 Use of rake and shovel for sandpit: 17/24 Frequency of cleaning sandbox: 14/24
Kotch 1994	Hygiene	Caregivers at child daycare centres (CDCCs)	Develop feasible, multi component hygienic intervention to reduce infections in children at CDCCs who are at increased risk	Hygiene curriculum for caregivers Availability of soap, running water, and disposable towels Waterless disinfectant scrub (Cal Stat) used only if alternative was not washing at all. Handouts posted in CDCC.	Delivery of hygiene curriculum to caregivers through initial training session which required demonstration of participants’ hand‐washing and diapering skills Local procedures: Hand‐washing of children and staff Disinfection of toilet and diapering areas Physical separation of diapering areas from food preparation and serving areas Hygienic diaper disposal Daily washing and disinfection of toys, sinks, kitchen and bathroom floors Daily laundering of blankets, sheets, dress‐up clothes Hygienic preparation, serving, and clean up of food Separate training of food handlers As‐required induction training for new staff Onsite follow‐up training reinforcing adaptations, demonstrations and discussion of hygiene techniques, responding to questions, and review of handouts Monthly meeting with centre directors to encourage leadership and support	Research team delivered training. Scrub donated by Calgon Vetal Laboratories.	Face‐to‐face training and follow‐up group and individually	Classrooms of child daycare centres in the USA	8 months overall 3‐hour initial training session Cleaning schedules as described in column What (procedures) Onsite follow‐up training 1 week and 5 weeks later	Follow‐up sessions addressed questions and local adaptations to procedures. As‐required induction training	During intervention, research team encouraged directors to address physical barrier to hygiene practice, such as distance between sink and diapering areas and sink access in rooms.	Follow‐up sessions reinforced training. Meeting with directors 5 weekly unobtrusive recorded observation by training staff	Rate of compliance to barrier modification was better in younger centres, which were more likely to have written guidelines.
McConeghy 2017	Multifaceted hand‐washing and surface‐cleaning intervention	Nursing homes and their staff	Reduce exposure to pathogens and person‐person transmission in high‐risk facility of close environment and potentially contaminated surfaces through multifaceted intervention equipping staff to protect residents from infection within the “culture” of care	Education and launch materials Online module for certified nursing assistants about: infection prevention, product, and monitoring "Essential bundle" of hygiene products supplied at no cost: ‐ hand sanitiser gel and foam ‐ antiviral facial tissues ‐ disinfecting spray ‐ hand and face wipes Plus additional: ‐ 4 skin cream and wipe products iPads for compliance audits Newsletters for support during intervention	Pre‐intervention: NH administrators required to: ‐ identify a "Heroes In Prevention" champion and team ‐ allow all staff participation in education ‐ iPad use for staff in each floor or community ‐ ask staff to incorporate intervention into workflow Delivery of 3 components: ‐ education ‐ cleaning products ‐ compliance audit and feedback Education: Launch event for all staff to publicise programme and explain roles Intensive training of "hygiene monitors" for data collection and compliance audit and feedback tool Training of site champion Training of select group of certified nursing assistants (online module) Audit and feedback activities Ongoing support during intervention: ‐ newsletter with best practices ‐ teleconferences with each NH ‐ "onboarding" education of new staff	Study personnel equipped staff with knowledge and tools and support. NH staff (e.g. champion, hygiene monitors, nursing assistants) delivered aspects of interventions after specific training.	Face‐to‐face interaction with staff for planning and some aspects and delivery of products Some aspects delivered online (e.g. nursing modules, compliance auditing)	Nursing homes in the USA Onsite and at unit/team levels Online training	6 months overall: training period: 3 months 1‐hour launch event 1 or 2 hygiene monitors/site 1 champion/site 1‐hour online module for selected nursing assistants iPads for each community or floor Weekly teleconferences initially decreased in frequency over time. Weekly measurement of product consumption	Sites could use existing comparable products from another vendor and fill in any gaps with study products. New staff provided with education, as needed and came onboard. Retraining of sites with low training participation rates	2 sites retrained due to low training participation rate.	Cloud‐based audit and feedback system via secure login to web browsers on NHs’ existing computers or via iPads included weekly product consumption to get measure: weekly count of product units consumed x no. of hand hygiene occasions	Online training participation rates: > 90% for 3/5 sites, 13% and 23% for 2/5 Administrators demonstrated high fidelity in reporting measures of hand‐washing (> 80% of time). Hand‐washing rates in Figure 1B in paper reported as “relatively constant” and “not ideal in the first few months”, but improved significantly over time.
Sandora 2008	Multifactorial intervention, including alcohol‐based hand sanitiser and surface disinfection	Elementary school and its students	Reduce transmission of infections in schoolchildren through improved hand hygiene and environmental disinfection	1 container of disinfecting wipes (Clorox Disinfecting Wipes (The Clorox Company, Oakland, CA, USA); active ingredient, 0.29% quaternary ammonium chloride compound) Pre‐labeled 1.7‐ounce containers of alcohol‐based hand sanitiser (AeroFirst non‐aerosol alcohol‐based foaming hand sanitiser (DEB SBS Inc, Stanley, NC, USA, for The Clorox Company); active ingredient, 70% ethyl alcohol) Receptacle in classrooms for empty containers	Sanitiser and wipes provided to classroom/teacher with instructions for use. Teachers disinfected desks once daily. Hand sanitiser to be used: before and after lunch, after use of the restroom (on return to the classroom; hand hygiene with soap and water occurred in the restroom, because sanitisers were not placed there), after any contact with potentially infectious secretions (e.g. after exposure to other ill children or shared toys that had been mouthed)	Research team arranged supply of materials and instructed teachers on use. Teachers instructed in use of materials and in collecting empty containers and distributing new product.	Products provided to schools. Instruction provided face‐to‐face to teachers and children.	Elementary schools and their classrooms in the USA	8‐week period Desks disinfected once a day.	Products replenished as needed.	None described.	Individually labelled containers collected every 3 weeks from the classroom to assess adherence.	Product usage: average wipes used/week: 897 (128 wipes/classroom/week) Average bottles of hand sanitiser used per week: 8.75 (1.25 bottles/classroom/week)
**Quarantine/Physical distancing**													
Helsingen 2021	Rapid‐Cycle Re‐Implementation of TRAining Facilities in Norway (TRAiN) hygiene and physical distancing measures	Members of health and fitness training facilities aged 18 to 64 years not at increased risk for severe COVID‐19	Enable safe re‐opening of fitness training facilities to maintain health and fitness by reducing the risk of SARS‐CoV2 transmission	Infection mitigation measures described by “Norwegian guidelines for Hygiene and Social Distancing in Training Facilities during the COVID‐19 Pandemic” (in Norwegian t-i.no/wp-content/uploads/2020/04/Bransjestandard-for-sentre.pdf) See Supplementary Appendix for “Standard for COVID‐19 infection prevention measures in fitness centers during the TRAiN‐study” Disinfectant readily available at workstations and strategic places (reception, booking station, changing rooms, toilets, water taps used for drinking or refilling bottles) Rubbish cans without lids Washbasin with soap or hand disinfection Personal microphones for instructors (i.e. not shared) Infection preventive measures reminders online and via posters in facilities	Implementation of the following during regular floor training facilities and group classes: ‐ avoidance of body contact ‐ 1 metre distance between individuals, ‐ 2 metre distance for high intensity activities Provision of disinfectants at all workstations Requirement of HW and cleaning of all equipment by members before and after use with utensils provided No physical contact between participants or participants and instructors Regular cleaning of facilities by facility employees Create lists of what should be cleaned and how often Disinfection of instructor microphones Extra cleaning of frequently touched surfaces (e.g. door handles, card readers, washbasin batteries) Frequent refilling at all hygiene stations Avoid queuing by making sure group classes do not start and stop at same time and keep 15 min minimum between group classes Access control by facility employees Closure of showers and sauna but changing rooms open Staff presence during all opening hours Removal of lids on trash cans Reminders of infection preventive measures Communication to members about changes to training for social distancing Advice to members to stay home if any COVID‐19 related symptoms Advice to members to avoid touching eyes, nose and mouth Closure of childcare facilities	Facility employees controlled access and enforced implementation of guidelines and procedures at all times Staff present during all opening hours Not reported if training needed for facility staff	Face‐to‐face individually and as a group	5 health and fitness training facilities in Oslo, Norway	3 weeks May 22nd to June 15th, 2020 Hours of access not reported; presumably the participants had unlimited access to training facility within the procedures for distancing	Masks not required, so were optional Change rooms available Access controlled to avoid overcrowding Staff monitored that distance measures were ensured Number of people attending depended on size of gym and associated changing rooms, showers and toilets. Facility to calculate the maximum number who could train at the same time while maintaining 1 to 2 m distance, as well as toilet, shower and change room capacity	None described	Staff monitored access and distancing No apparent measures of fidelity	None described
Miyaki 2011	Quarantine from work (stay‐at‐home order)	Employees	Prevent spread of influenza in workplaces by quarantining workers who had a co‐habiting family member with an ILI	Full wages to employee	Non‐compulsory asking of workers whose family members developed an ILI to stay at home voluntarily on full wages. Daily measuring of temperature before leaving work. Where symptoms were doubtful, industrial physician made judgement. Company doctors provided input on cancelling of stay‐at‐home orders as required.	Health management department oversaw the procedures and decisions.	Mode of advice to employees not described.	Car industries in Japan	Stay‐at‐home order for 5 days after resolution of ILI symptoms or 2 days after alleviation of fever over 7.5 months	Strict standard for cancelling of stay‐at‐home orders described.	None described.	Recording of compliance with stay‐at‐home request	100% compliance to stay at home reported.
Young 2021 (additional source: Denford 2022)	Daily contact testing (DCT) with Lateral Flow Device (LFD) for contacts of COVID‐19 cases	Students and staff from secondary schools and further education colleges	Provide a quicker, more convenient and alternative testing option and policy for COVID‐19 close contact testing in schools, as an alternative to self‐isolation	SARS‐CoV‐2 Lateral Flow Device (LFD) (Orient Gene, Huzhou, China)^[47]^	In addition to twice weekly asymptomatic testing with LFD according to national policy: students and staff who were close contacts^[48]^ of students or staff members who had a positive LFD or PCR were identified and offered daily LFD testing on arrival at school or college each morning (if asymptomatic and no household member isolating due to testing positive for COVID‐19) Participants swabbed own nose (anterior nares), supervised by trained staff. Swabs tested by school staff using LFC Contacts with negative LFC attended education but were asked to self‐isolate at home after school and on weekends/holidays Contacts with 5 negative tests (tests done over 7 consecutive days) including one on or after the 7th day of testing were released from self‐isolation Contacts with positive test were required to self‐isolate for 10 days, along with their contacts. Their school‐based contacts were identified and process repeated	A study worker was funded at each school but role not specified School staff tested the swabs that were taken by students Study staff trained according to national NHS Test and Trace standard process supervised LFD testing	Individually and face to face	172 secondary government funded, residential, special and independent day schools and further education colleges in England	March to May 2021 Daily contact testing was performed at arrival at school each morning Day 1 of testing began the day after a case was identified Testing was done over 7 consecutive days (allowing for no testing on weekends) Schools actively participate between 19 April 2021 to 27 June 2021 (considered periods of low to moderate COVID‐19 incidence)	When testing could not start immediately following identification of a case (e.g. due to a weekend), testing could start within 3 days of case identification	None reported	Daily participation rates in IG measured per day and per participant Compliance was calculated / school / week, and participant type, (= sum of all study school days of individuals eligible for DCT returning a test result or already having completed follow up each day, divided by the sum of individuals eligible for DCT. Qualitative interviews conducted to understand reasons for participation and not (reported separately in Denford 2022)	Testing did not occur on 15.8% of person‐school‐days due to school or public health agency directives IG participation rate: 42.4% with marked variation between schools (range 0% to 100%). See Figure 2 for non‐participation reasons breakdown (e.g. testing kit unavailable, whole cohort moved to isolation). Staff more likely to participate than students. See Figure 2 for participation by school type breakdown “Although contacts at government‐funded schools with students 11–16 years old with a low proportion of free school meals were most likely to participate, other school types were similar, such that differences in participation related to factors other than school type.” (p. 1227) Qualitative analysis of interviews indicated daily testing may be feasible and acceptable but needs improved communication to students and parents about rationale, test interpretation and actions (Denford 2022)
**Other (miscellaneous/multimodal) interventions**													
Ashraf 2020 (additional sources: Arnold 2013, Luby 2018, Parvez 2018, Rahman 2018, Unicomb 2018)	6 active interventions of Water, sanitation, hygiene (WASH) and nutrition components: A. Water (W) B. Sanitation (S) C. Handwashing (H) D. Water + sanitation + handwashing (WSH) E. Nutrition F. Nutrition + WSH (WSHN	Residents of households of village compounds and for some interventions, particularly pregnant women and their infants and children < 5 years	Improve environmental conditions to interrupt transmission of respiratory pathogens and improve child malnutrition thereby reducing childhood respiratory illness and improving childhood morbidity based on the Integrated Behavioural Model for Water Sanitation and Hygiene^[33]^ and 2 years of iterative testing and revision. Intervention specific behavioural objectives: W: drink treated and safely stored water S: safe faeces disposal H: HW with soap at key times N: age‐appropriate nutrition birth to 24 months	Free technologies and supplies: W: chlorine (sodium dichloroisocyanurate) tablets (Aquatabs, Medentech, Wexford, Ireland) ‐ 10 L insulated safe storage vessel (Lion Star Plastics, Sri Lanka) with a lid and tap for drinking water per household S: Dual‐pit pour flush latrines with water seals for all compound households. Each pit had 5 concrete rings 0.3 m high; ‐ Potties^[34]^ (RFL, Bangladesh) ‐ Sani‐scoops^[35]^ (locally developed hand‐tool made for the trial for removal of faeces from compound) for households with index children H: 2 HW stations, 1 water reservoir near kitchen (16 L) and 1 near latrine (40 L), each with basins for rinsing with a soapy water bottle (RFL, Bangladesh) and detergent sachets for index households^[36]^ N: supply of lipid‐based nutrient supplements (LNS, Nutriset; Malaunay, France) (for 6 to 24 months olds) 2 10g sachets per day per child; (118 kcal, 9.6g fat, 2.6g protein, 12 vitamins and 10 minerals) Cost: USD 0.08/day 18‐month shelf life Stipends for CHWs (USD 20/month for 24 months) delivered through mobile phone network to ensure timely payments Promoter’s guide for visits for each relevant intervention including: ‐ visit objective, ‐ target audience ‐ steps and materials to be used CHW ID badges Cell phones for CHW supervisors Training Plan and Manual for CHW supervisors covering: i) basic training ‐ introduction of project, CHW roles and responsibilities, introduction to behaviour‐change principles based on the IBM‐WASH theoretical framework and interpersonal and counselling communication skills. ii) Intervention‐specific training iii) classroom practice / role playing	Provision and delivery of supplies or installations as described in Materials column according to intervention type or combination. Interventions deployed so that they were in place before index children were born In combined intervention arms, the sanitation measures were delivered first, followed by handwashing, then water treatment. Household visits and community discussions based on behaviour change strategy by CHWs (paid a monthly stipend), including interactive sessions for developing solutions to improve practice. Key recommendations per IG: W: children drink treated, safely stored water from vessel (filled vessel with added 1 33 mg tablet, wait 30 min before drinking) S: family use double pit latrines, potty train children and how to safely dispose of faeces and clean and maintain latrines H: family wash hands with soap after defecation, after cleaning a child who has defecated, before eating or before feeding a child, and before food preparation N: recommendations for exclusive breastfeeding up to 180 days and maternal and infant nutrition to mothers and index children; introduce diverse complementary food at 6 months; feed LNS from 6 to 24 months, mixed into the child’s food (not intended as a replacement for breastfeeding or complementary foods). Messages adapted from the Alive & Thrive programme^[37]^ On household visits, following a structured plan, CHWs greeted targeted household members, checked presence and functionality of relevant hardware and signs of use, observed recommended practice using a guide. CHWs used discussions, video dramas, storytelling, games and songs and provided training on hardware maintenance, where applicable Adherence observed and measured by separate team Supervision meetings of CHWs and periodic internal monitoring of their performance Intervention Delivery Team managed delivery through regular team phone calls, field meetings, field reports and liaison with relevant government and other stakeholders. It co‐ordinated CHWs to ensure rapid identification of issues with delivery. Including a dedicated training officer, it also trained the CHW supervisors who then trained the CHWs under their supervision (“train the trainer” approach)	540 CHW or ‘promoters’ who were local women and residents of study villages recruited through transparent merit‐based selection methods and consultation with community leaders CHWs had completed minimum of 8 years formal education, lived within walking distance of IG cluster and passed a written and oral examination. They attended multiple training sessions and quarterly refreshers. Training covered active listening, strategies for developing collaborative solutions and technical aspects of interventions (see Table 1 of Luby 2018 for more details) CHWs were trained by 47 CHW supervisors who received direct training on intervention delivery Hardware installation team (n = 18) 9 field research officers The Intervention Delivery Team^[38]^ co‐ordinated delivery including CHWs, overseen by Principal Investigators with consultation from Technical Advisory Group (see Unicomb, 2018) Dedicated Training Officer and Communication Development officer Adherence observed by separate team who received formal 21 day training	Mostly face to face in groups and individually with some activities by phone	Households and compounds (n = 5551) of rural villages in Gazipur, Kishoreganj, Mymensingh and Tangail Districts in Bangladesh Households spread across 0.2 to 2.2 km radius	2 years from May 2012 6 to 8 households / CHW 1:12 supervisor to CHW ratio CHWs visited households 1 / week for first 6 months, then at least 1 / fortnight Promoter training: Initial: W, S, HW: 4 days; N, WSH: 5 days; WSHN: 9 days Refresher training: 1 day each 21 day training of adherence team Monthly CHW supervisor meetings	CHWs identified and addressed any barriers that arose through ongoing dialogue with caregivers CHWs met with supervisors monthly to adapt technology and behaviour‐change approaches to meet evolving conditions CHW supervisors available by cell phone as needed Training of promoter varied in content and length depending on intervention type Potties provided if children < 3 years	S: latrine pits adapted when insufficient space (2% of cases) Functional water seals count was low (< 80% benchmark) in initial months which triggered a rapid response which improved uptake (Rahman 2018); households were using own latrines with broken water seals in parallel with trial latrines so pre‐existing latrines were closed, visits by CHWs were increased and water‐seal removal or breakage was discouraged Initial professional trainer for CHW training did not engage trainees enough so replaced with internal training resource group Due to observation of intervention fatigue reported by CHWs and sub‐optimal practices observed, new behaviour change activities were developed (e.g. further technology use, increasing self‐efficacy and roles for men)	Measured by a separate trained team (university graduates) at regular intervals using a priori benchmarks: a) surveys and spot checks in 30 to 35 households / IG / per month, over 20‐month period; b) 5‐hours of structured observations in 324 IG and 108 control households, approximately 15 months after interventions commenced. Measured: W: Presence of stored drinking water with detectable free chlorine (> 0.1 mg/L) S: a latrine with functional water seal, sani‐scoop accessibility H: presence of soap at primary HW stations N: reported consumption of LNS sachets See Rahman 2018 for more details (Table 1) Continuous oversight and periodic monitoring of CHWs performance (CHW replaced within 1 month of attrition or critically low performance	CHWs visited more than planned (5 to 7 / month) which researchers suggest may have affected uptake Reported “high adherence to all interventions” with “marked differences in promoted behaviors from the control group at both year 1 and year 2,” with over 75% adherence in the single IG and combined IGs. Similar adherence in single W, S, H and N IGs compared with WSH and WSHN S: observed use of latrines: 94% to 97%; child sanitation practices (37% to 54%) H: HW with soap in IG more common after toilet use (67% to 74%) versus 18% to 40% in non‐IGs and after cleaning child’s anus (61% to 72%) but low before food handling W: > 65% mothers and children observed drinking chlorine‐treated water from safe container N: LNS feeding > 80% 33 low performing CHWs discontinued See Luby 2018, Parvez 2018, Arnold 2013, Unicomb 2018 for more details
Farr 1988a trial 1	2 active interventions in addition to control of no tissues: A. Virucidal nasal tissues B. Placebo tissues	Families	Reduce transmission of viruses from hand contamination via hand‐to‐hand contact or large‐particle aerosol through tissues for nose blowing and coughs and sneezes	3‐ply tissues with: A. 5.1 mg/inch^2^ (2.54 cm^2^) of the virucidal mixture (58.8% citric acid, 29.4% malic acid, 11.8% sodium lauryl sulphate) B. 3 mg/inch^2^ (2.54 cm^2^) of saccharin applied uniformly to all 3 plies of the tissue Tissues prepared by Kimberly‐Clark Corporation, Neenah, WI, USA.	Family visits to distribute tissues Weekly contact of mother Families instructed to only use supplied tissues.	Nurse epidemiologist visited families.	Face‐to‐face visits to families and individuals in families (especially mothers)	Communities in the USA	6 months overall Monthly family visits Weekly contact with mother	Not described	Not described	Family visits and weekly contact with mother to encourage compliance	Not described
Farr 1988b trial 2	2 active interventions (no control): A. Virucidal nasal tissues B. Placebo tissues	Families	Reduce transmission of viruses from hand contamination via hand‐to‐hand contact or large‐particle aerosol through tissues for nose blowing and coughs and sneezes	2‐ply tissues containing: A. 4.0 mg/inch^2^ (2.54 cm^2^) of antiviral mixture (53.3% citric acid, 26.7% malic acid, 20% sodium lauryl sulphate) B. 3 mg/inch^2^ (2.54 cm^2^) of succinic acid, malic acid, sodium hydroxide, and polyethylene glycol Tissues prepared by Kimberly‐Clark Corporation, Neenah, WI, USA.	Family visits to distribute tissues and encourage compliance Weekly contact of mother Families instructed to only use supplied tissues.	Nurse epidemiologist visited families monthly. Study monitor visited bimonthly.	Face‐to‐face visits to families and individuals in families (especially mothers)	Communities in the USA	6 months overall Monthly family visits Weekly contact with mother Bimonthly study monitor visit	None described.	None described.	Bimonthly study monitor visits to encourage compliance as well as monthly and weekly contact by nurse	In 124/222 families, 1 or more family members reported not using the tissues regularly and/or reported having side effects from the tissues.
Fretheim 2022a (additional source: Fretheim 2022b (protocol)	GLASSY (GLasses Against transmission of SARS‐CoV‐2 in the communitY	Adult members of the public who did not regularly wear glasses and who owned or could borrow glasses to use (e.g. sunglasses)	Provide a simple, readily available, environmentally friendly, safe and sustainable means of personal protection from infection with respiratory viruses including SARS‐CoV‐2	Instructions via online portal Regular eyewear, e.g. sunglasses owned by participant or that could be borrowed by participant	Request to wear sunglasses or other types of glasses when outside home and close to others in public spaces for 14 days	Research team	Individually Instructions provided via email and online portal (Nettskjema‐platform)accessed via web‐page hosted by the Norwegian Institute of Public Health	Outside the home, e.g. on public transport, in shopping malls (in Norway)	14 days when outside and close to others in public spaces Over 11 to 12 week period (February – April 2022)	Could borrow glasses if did not own any	None reported.	No contact was made with participants between enrolment and data collection.	Reported use of glasses often, almost always, or always: IG: 71% CG: 11% Negative experiences (especially fogging with mask use): IG: 21/76
Longini 1988	2 active interventions (no control): A. Virucidal nasal tissues B. Placebo tissues	Households and their families	Prevent intrafamilial transmission of viral agents in a community setting	Treated tissues of 3‐ply material identified with no specific identifiers (Kimberly‐Clark Corporation) with inside layer containing: A. citric and malic acid plus sodium lauryl sulphate; B. succinic acid.	Tissues delivered to households with specific instructions on use (all purposes, when blowing nose, coughing or sneezing) and to discard after use and to help young children use tissues if develop a cold.	Tissues assigned by study sponsor (Kimberly‐Clark Corporation).	Supply of tissues throughout 5‐month trial period	Households in the USA	5 months' overall supply	Resupply of tissues as required	None described.	Reported use of tissues “not at all, some of the time, most of the time, or all of the time”	Reported use “all of the time”: A. versus B. 82% versus 71%
Chard 2019 (additional details from Chard 2018)	Water, Sanitation, and Hygiene for Health and Education in Laotian Primary Schools (WASH HELPS)	Primary schools and their students	Prevent the spread of pathogens within schools through improved water supply and hygiene facilities and improved WASH habits in children at home and throughout the life course	For each school: Water supply for school compound: (borehole, protected dug well with pump, or gravity‐fed system) Water tank to supply toilet and hand‐washing station School sanitation facilities (3 toilet compartments) Hand‐washing facilities: 2 sinks with tapped water and supply of soap available (1 bar of soap/pupil) 3 group hand‐washing tables with soap and water At least 1 drinking water filter per classroom Schedules of daily group hand‐washing, compound and toilet cleaning Cost per school: USD 13,000 to 17,500	Provision of school: Water supply, sanitation facilities, hand‐washing facilities (individual and group), drinking water filters Behaviour change education and promotion including daily group hygiene activities Daily hand‐washing and cleaning schedules	UNICEF paid for materials. School and teachers conducted daily hand‐washing activities with children. Students participated in daily group cleaning activities.	Facilities provided within schools. Children participated in group hand‐washing and cleaning.	Primary schools and their classrooms (in Laos)	One‐off provision of water and hygiene facilities Daily hand‐washing activities and cleaning for 1 school year Cleaning schedules posted in at least 1 classroom near toilet.	Water supply tailored to the school requirements/environment. Sanitation facilities provided as needed and designated for boys, girls, and students with disabilities.	Rain water tank provision affected by rain water supply, so changed to tanks with motorised hand pumps or gravity‐fed water supply systems. Theft and animal consumption of supplied soap reduced supply.	Unannounced visits every 6 to 8 weeks for structured observations to measure fidelity and adherence Fidelity Index score (0 to 20): for hardware provided see Table 1 in paper and protocol Adherence index: student report of behavioural outcomes index score (0 to 4)	Fidelity: 30.9% across all schools and visits Adherence: 29.4% Hardware provision: 87.8% of schools School‐level adherence: 61.4% Group compound cleaning: 94.8%, toilet use: 75.5%, group toilet cleaning: 68.3%, group hand‐washing: 48.7%, individual hand‐washing with soap after toilet use: 23.9%. Further details (Chard 2018)
Hartinger 2016	Integrated environmental home‐based intervention package (IHIP)	Households and their householders including children	Reduce infections and improve child growth in households in rural communities with limited facilities through a multi component, low‐cost environmental intervention to improve drinking water, sanitation, personal hygiene, and household air quality developed in pilot (Hartinger 2011; Hartinger 2012) using a participatory approach that addressed local beliefs and cultural views	Per household: "OPTIMA‐improved stove": improved ventilated solid‐fuel stove Kitchen sink with in‐kitchen water connection providing piped water Point‐of‐use water quality intervention applying solar disinfection to drinking water	Community engagement with local and regional stakeholders in design and development Provision of stoves, kitchen sinks, and plastic bottles for solar water treatment, and hygiene education Training of mothers/caretakers in: ‐ solar drinking‐water disinfection (SODIS)^[39]^ according to standard procedures ‐ hand hygiene (washing own and children’s hands with soap at critical times^[40]^) ‐ advice to separate animals and their excreta from the kitchen environment Project‐initiated repairs	Health promoters hired local elementary school teachers and implemented and promoted the interventions. 4 teams of field staff conducted spot‐check observations.	Face‐to‐face and to individual households; mode of delivery of training as individual or group not described	Households in rural communities in Peru	Stoves and sinks installed over initial 3 months. Monthly reinforcement over 12 months of SODIS, child and kitchen hygiene Weekly spot checks of compliance Repairs after 9 months Environmental samples test middle and end of 12‐month surveillance.	Tailored to particular household facilities and environments as needed and to local beliefs and cultural customs Repairs to stoves as needed and checked at 9 months	Not described	Weekly spot‐check observations of household hygiene and environmental health conditions (e.g. presence of SODIS bottles on the roof or kitchen) using a checklist Monthly self‐report by mothers of stove and sink use	SODIS use: 60% initially and 10% at end of study Self‐reported use by mothers: 90% with slight decrease at end Self‐reported stove use: 90% daily Sink use: 66% daily 35% of stoves needed minor repairs, 1% needed major repairs. Best‐functioning stoves achieved mean 45% and 27% reduction of PM_2.5_ and CO, respectively, in mothers’ personal exposure.
Huda 2012	Sanitation Hygiene Education and Water Supply in Bangladesh (SHEWA‐B)	Villages and their households with a child < 5 years old	Reduce illness in children < 5 years by improving hygiene practices, sanitation and water supply and treatment in their household	Materials for training of community hygiene promoters and promotion activities including flip charts and flash cards with messages alerting participants to presence of unobservable “germs” and practices to minimise germs See Box 1 in paper for 11 key messages.^[41]^	Engaging local residents under guidance of local NGOs to develop community action plans addressing: Latrine coverage and usage Access to and use of arsenic‐free water Improved hygiene practices, especially hand‐washing with soap Recruitment and appointment of community hygiene promoters Household visits, courtyard meetings, and social mobilisation activities (e.g. water, sanitation and hygiene fairs, village theatre, group discussions in tea stalls (the social meeting point for village men)) by community promoters Structured observation in households	Community hygiene promoters (local residents with at least 10 years' schooling trained for 10 days on behaviour change communication in water, sanitation, and hygiene)	Face‐to‐face delivery to groups (villages and households) and individuals	Villages and households in districts of Bangladesh Community activities held in villages. Meetings held in courtyards of groups of households. Household visits	18 months overall Expected household visit and courtyard meeting every 2 months Hand‐washing opportunities: after own or child’s defecation, prior to preparing and serving food, prior to eating and feeding a child	Community action plans developed for and by local residents.	Not described	Structured observation of hand‐washing and child faeces disposal behaviour in households and spot checks of type of household water and sanitation facilities	HW: Food‐related: No significant difference from baseline to 18 months; IG versus CG After anus cleaning: 36% versus 27% Defecation: 30% versus 23% No access to latrine decreased from 10.3% to 6.8%. No significant improvement in access to improved latrines, solid waste disposal, drainage systems, and covered containers for water storage
Ibfelt 2015	Disinfection of toys	Daycare nurseries	Reduce transmission of pathogens via shared toys in daycare environment through regular disinfection treatment	Disinfectants: Turbo Oxysan (Ecolab, Valby, Denmark) for washing machines Sirafan M, Ecolab (1% to 3% benzalkonium chloride, 1% to 3% didecyldimethylammonium chloride, and 5% to 7% alcohol ethoxylates) for immersion or wiping	Collection and commercial cleaning of toys from nurseries: ‐ linen and toys suitable for washing machines were washed at 46 °C and subsequently disinfected ‐ toys not suitable for washing machines immersed in disinfectant or wiped with microfibre cloth	Commercial cleaning company: Berendsen A/S, Søborg, Denmark	Cleaning companies collected the toys and linen and cleaned them offsite, then returned them.	Daycare nurseries in Denmark Commercial industrial cleaning facility	2 to 3 months overall Cleaning every 2 weeks	Staggered cleaning to ensure children had toys to play with whilst others were being cleaned	None described.	None described.	None described.
Najnin 2019 (see also Qadri 2015 for further details)	2 active interventions: A. Combined cholera vaccine and 'behaviour change communication' intervention B. Cholera vaccine‐alone group	Low‐income households and compounds	Prevent or reduce transmission of respiratory illness based on the Integrated Behavioural Model for Water Sanitation and Hygiene (IBM‐WASH) theoretical framework (Dreibelbis 2013; Hulland 2013)	A. and B. Cholera vaccine ShanChol™ (Shantha Biotechnics‐Sanofi, India) A. Following hardware per compound: a. Hand‐washing hardware: (i) Bucket with a tap (provided free of charge) (ii) Soapy water bottle (mixture of a commercially available sachet of powdered detergent (∼USD 0.03) with 1.5 L of water in a plastic bottle with a hole punched in the cap) supplied by participating compounds (iii) Bowl to collect rinse water after washing hands (see photo in text or in Najnin 2017 doi.org/10.1093/ije/dyx187) b. Water treatment hardware: Dispenser containing liquid sodium hypochlorite See Figure 2 in Najnin 2017 for photos of both doi.org/10.1093/ije/dyx187 and more details. Participants own water vessels for water treatment Print materials for behaviour change to compounds and households	A. and B. Provision of cholera vaccine (2 doses at least 14 days apart) Provision of hand‐washing hardware and behaviour change communication activities Encouragement of hand‐washing after defecation, after cleaning child’s anus, and before preparing food Encouragement to add chlorine to own water vessels Benefits were again explained. Follow‐up visits by health promoters	Dushtha Shasthya Kendra (DSK), an NGO, delivered the hardware and behavioural intervention (through community health promoters). Separate data collectors observed soap availability.	Hand‐washing and water treatment hardware mostly delivered at the compound level in person. Behaviour change communication messages were delivered both at compound and household levels.	Households and compounds (where several households share a common water source, kitchen, and toilets) in Bangladesh	Behaviour change communication messages delivered first (within 3 months of cholera vaccination). Point‐of‐use water hardware provided 3 months later. Follow‐up health promoter visits 3 times in 2 months after hardware installation, then 2 times/month (over nearly 2 years).	Hardware‐related problems (breakage/leakage) were addressed on health promoter follow‐up visits.	None described.	Unannounced home visits by data collectors who observed presence of soap/soapy water and water in most convenient place for hand‐washing (either reserved in a container or available at the tap) Residual chlorine was measured indicating uptake of chlorine dispenser.	Presence of soap / soapy water and water: A. Handwashing group compounds: 45% (1729 / 3886); B. Vaccine‐only group compound: 22% (438 / 1965); C. Control: 28% (556 / 1991) Residual chlorine present in stored drinking water of 4% (160/3886) of households in the vaccine‐plus‐behaviour‐ change compound and none in the other 2 compounds.
Swarthout 2020 (additional sources: Arnold 2013, Christensen 2015, Dentz 2017, Null 2018, Pickering 2019)	6 active interventions of water, sanitation, and handwashing (WASH), and nutrition components: A. Water (W) B. Sanitation (S) C. Handwashing (H) D. Combined (WSH) E. Nutrition (N) F. Combined (WSHN)	Residents of households of villages and for some interventions, particularly pregnant women (Mamas) and their infants and children < 5 years; Landowners of communal water sources and compound heads for latrine upgrades and construction	Improve environmental conditions to interrupt transmission of respiratory pathogens and improve child malnutrition thereby reducing childhood respiratory illness and improving childhood morbidity based on a literature review, a theory‐based approach (health belief, social cognitive theory and persuasion theory),^[42],[43],[44]^ formative research and the WASH Benefits pilot RCT (Christensen 2015)	Free technologies as appropriate to IG: W: water treated with sodium hypochlorite (1.25% solution / 2 mg/L) using chlorine dispensers installed at communal water source collection points or bottled chlorine (1L for 333 20‐l jerry‐cans worth)^[45]^ provided to households in compounds Chlorine strips to test chlorine levels S: installation of new or improvement of existing latrines with plastic slab latrines with tight‐fitting lids; plastic potties and sani‐scoops H: 2 HW stations (2‐foot pedal‐operated jerry‐cans that dispensed soapy and rinse water), 1 near food preparation, 1 near latrine. Rinse water provided by households; bar soap for soapy water container N: 2 x 10 g sachets / day / child of lipid‐based nutrient supplementation (LNS) “Mwanzobora”, (Nutriset, Malaunay, France) (118 kcal/day and 12 essential vitamins and 10 minerals) See Figure 2 of Christensen 2015 for photos of examples of some of the materials Community meeting and household visit summary sheets (in Kiswahili and English) and list of materials provided as PDFs at osf.io/7j9sk/ Key messages and visual aids provided at osf.io/7j9sk/ Including ~6 primary key messages per intervention, each with a series of specific topics, visual aids, and engagement activities (e.g. storytelling, mottos, etc.). Visual aids included: ‐ cue card reminders ‐ picture sheets for use by promoters ‐ calendars for households with key messages ‐ stickers for LNS box depicting appropriate feeding and storage Promoter Training Materials for trainers and trainees for each intervention for initial training and for refresher training including detailed PDF training manuals available at osf.io/7j9sk/ focusing on key hygiene messages, visitation scripts and visual aids and hardware for each intervention^[46]^ Promoters’ supplies: Branded t‐shirt, mobile phone, job aids and intervention materials, payment ($US15/month for first 6 months, then $9/month thereafter), detailed plans for every visit (key messages, scripts for visual aids, instructions for activities)	Provision and delivery of supplies or installations as described in Materials column according to intervention type or combination Provision of study materials to promoters Community meetings Household and community visits by promoters who: ‐ delivered intervention‐specific behaviour change messaging focusing on themes of nurture, aspiration and self‐efficacy, considering convenience and cultural norms to improve adherence using scripts and visual aids; ‐ provided instructions on hardware use and consumable supplies where applicable ‐ advocated: W: drinking water treatment with sodium hypochlorite S: use of improved latrines for defecation and safe disposal of children’s and animals’ faeces and use of plastic potties by children < 3 years and sani‐scoops for faeces removal H: HW with soap before food preparation and after defecating (including assisting child); helped participants identify compound members to refill taps and manage barriers to use such as running out of soap N: early initiation of breastfeeding, exclusive breastfeeding 0 to 6 months and continued till 24 months; at 6 months, introduction of appropriate and diverse complementary foods; feeding frequency and during illness; supply of LNS to children 6 to 24 months and instruction to mix it was foods twice/day Promoters used visual aids to promote messages: ‐ cue cards provided to Mamas at initial visits to hang on walls for reminders ‐ picture sheets used by promoter to explain key concepts or messages ‐ calendars provided to households during first compound visit ‐ stickers attached to LNS box Adherence checking unannounced visits Initial training on intervention‐specific behaviour change messages and materials Refresher training Periodic observation and supportive supervision by study staff	Community‐based health promoters nominated by their local communities and trained in the relevant intervention to be implemented Field enumerators assessed adherence in compounds Study staff trained promoters, provided periodic observation and supervision and monthly phone calls	Face to face in groups (e.g. households or compounds) or individuals (mothers and their children)	8246 households and 7960 compounds of rural villages in Bungoma, Kakamega, and Vihiga counties in western Kenya	Installation and supply of materials before community meetings Community meeting 6 weeks after enrolment Monthly visits (45 to 60 min in 1^st^ year) by promoters over 2 years (2012 to 2014) Timing of visits detailed in procedures provided at osf.io/7j9sk/ W: 1 L bottle of chlorine / 6 months H: bar soap provided every 3 months N: LNS introduced at 6 months of age of child Promoter training: 6 days single IGs. 7 days combined IGs. Refresher training at 6, 12 and 18 months after initial training Supervision and observation of promoter by study staff at 2, 4, 9, 14 and 21 months and monthly phone calls	Training tailored for different interventions Troubleshooting of solutions to barriers to adherence by promoter and participants as needed Nutrition messaging was tailored to be age‐appropriate Materials provided in both in Kiswahili and English Chlorine dispensers located based on list of sources participants reported (at baseline) using for water collection Sani‐scoops and potties were to be washed by caregivers with soap and water after use and tools kept out of reach of children (see the visual aids provided to participants: osf.io/9r4kg/ for potties and osf.io/mz2c6/ for sani‐scoops)	None described	Participant reports of visits by promoters in past month Unannounced visits by staff to a random sample of at least 20% of participants in IGs at 2, 6, 10, and 19 months after the interventions began to confirm delivery of materials and monitor availability of intervention materials and recommended behaviours after the interventions began (Null 2018) W: monthly tests of chlorine concentration in stored water; negative results prompted discussions to address chlorination barriers S: participant report of access to improved latrine; field enumerators observed if latrine had plastic or cement slab or ventilation pipe; caregiver report that child faeces safely disposed H: field enumerator observed if water and soap available N: report of LNS sachets consumed by child in last week / 14	All interventions delivered within 3 months of enrolment Increased adherence indicators of ≥ 30% higher in all IGs relative to the control in the first year Adherence was comparable between the Individual IGs compared with combined IGs. W: 5 chlorine dispensers installed / cluster Year 1: 74% Year 2: 37% households were visited by a promoter in previous month W: Year 1: 42% Year 2: 21% had detectable total chlorine CG: 3% S: Year 1 and 2: > 80% had latrine access CG: 20% HW: Year 1: 77% Year 2: 21% had HW materials CG: 9% N: Year 1: 95% Year 2: 115% of expected sachets consumed See Null 2018 for more details
**Oral and/or nasal applications**													
Almanza‐Reyes 2021	Mouthwash and nose rinse with ARGOVIT silver nanoparticles (AgNPs)	Healthcare personnel (doctors, nurses, administrative staff) of a metropolitan hospital caring for patients diagnosed with atypical pneumonia and/or COVID‐19	Reduce morbidity in healthcare professionals exposed to SARS‐Co V‐2 by inhibiting virus replication	Per participant: ‐ 50 ml bottle of RGOVIT® AgNPs mouthwash and nasal rinse [Investigation and Production Center Vector‐Vita Ltd., Novosibirsk, Russia] (metallic silver 0.06%, polyvinylpyrrolidone 0.63%, hydrolyzed collagen 0.31%, distilled water 99% wt.) ‐ water ‐ cotton swabs	Individuals provided with spray bottle containing AgNPs solution with 1 wt% concentration (0.6 mg/mL metallic silver) and instructed to do 1 of the following or a combination: a) mix 4 to 6 spray shots (~ 0.5 mL) with 20 mL of water and gargle solution for 15 to 30 seconds at least 3 times/day (gargle) or b) do not dilute with water and cover the oral cavity evenly with 1 to 2 direct spray shots (spray) c) apply the same solution to the inner part of the nasal alae and nasal passage with cotton swab twice a day (nasal rinse)	Researchers supplied materials and instructions Participants self‐applied the mouthwash and nasal rinse materials	Individually and face to face	General hospital in Tijuana, Mexico	Over a 9 week period (April to June 2020) 4 to 6 spray shots of AgNP solution (0.5 mL) with 20 mL of water or 1 to 2 spray shots of solution without water for 15 to 30 seconds ≥ 3 times / day and 1 nasal lavage 2 times / day	Participants could choose application method	None described	Weekly self‐report of number of: daily gargles; mouthwashes with spray; mouthwashes by gargle + spray; and nasal rinses	Mean applications/ day: Gargle only: IG: 2 (n = 28) CG: 2.14 Spray only: IG: 2 (n = 34). Both gargle and spray: IG: 2 gargles, 4 sprays (n = 52) Nasal rinse: IG: 0.70 (n = 64) CG: 0.25
Gutiérrez‐García 2022	Nasopharyngeal and oropharyngeal rinses with a neutral electrolyzed water (SES)	COVID‐19 front‐line medical staff (nurses and physicians, males or females)	Reduce risk of COVID‐19 in frontline unvaccinated medical staff	SES (pH 6.5 to 7.5; REDOX potential 750‑950 mV; 0.0015% of active species of chlorine and oxygen) provided by Esteripharma S.A. de C.V Per participant: ‐ 4 plastic flasks of 240 mL oral SES (ESTERICIDE® Bucofaríngeo, COFEPRIS registration no. 1003C2013 SSA) with a graduated cap and ‐ 4 plastic flasks of 30 mL nasal rinse (EsteriFlu®, COFEPRIS registration no. 308C2015 SSA), with a valve for spraying	Written instructions provided to follow a prophylactic rinse protocol with SES 3 times/day for 4 weeks with advice on correct way to use the mouthwashes and sprays and the need to report possible side effects immediately: a) nasal cavity: 4 vertical sprays in each nostril, inhaled deeply at the time of each spray b) oral cavity: mouthwash and gargle 10 mL for 60 seconds, then spit out In addition to standard COVID‐19 safety protocols requiring wearing of adequate personal protection equipment at all times,^[49]^ frequent handwashing^[50]^ and disinfection of secondary uniform and footwear^[51]^ and bath at end of working day	Not clearly specified; leaders of nursing and other relevant healthcare department distributed the study information and were the point of contact and monitored the protocol so they may have distributed intervention materials	Individually and face to face	Mexican COVID‐19 hospital	4 nasal sprays (~ 0.4 mL) and 10 mL mouthwash gargle for 60 seconds 3 times / day for 4 weeks (September to November 2020)	None described	None described	None described	None described
Goodall 2014	2 active interventions: A. Vitamin D_3_ supplementation B. Gargling water	University students	Decrease the incidence of URTI through increased vitamin D levels (associated with greater frequency and severity of URTI) and gargling (as preventative measure against URTI)	A. Vitamin D_3_: container of 8 capsules of 10,000 IU (purchased from Euro‐Pharm International Canada Inc.) Weekly email reminder B. Gargling: 30 mL of tap water 2/day	A. Vitamin D: instructed to take 1 pill weekly B. Gargling: instructed to gargle twice daily for 30 seconds All participants received general lifestyle and health advice on sleep, nutrition, hand hygiene, and exercise.	Not specified, presumably the researchers, including a study pharmacist	Vitamin D_3_ supplied individually, but no further details. Method of lifestyle and health advice provision also not described.	In university student housing (in residences or off‐campus) in Canada	2 months overall Vitamin D_3_: weekly supplementation and email reminder Gargling: 30 mL of water for 30 seconds twice daily	None described.	None described.	None described.	None described.
Ide 2014	2 active interventions (no control): A. Green tea gargling B. Water gargling	High school students	Prevent influenza spread and infection in high school students who are at increased risk from close interaction through gargling as a non‐pharmaceutical intervention, specifically green tea containing highly bioactive catechin (‐)‐epigallocatechin gallate, with possible anti‐influenza virus properties	A. Bottled green tea (500 mL) containing a catechin concentration of 37 ± 0.2 mg/dL, including approximately 18% (‐)‐epigallocatechin gallate (manufactured by the Kakegawa Tea Merchants Association). Concentration measured by high‐performance liquid chromatography based on the average concentration in 10 bottles from the same production lot (September 2011) used for gargling in the study. B. Tap water	A. Provision of green tea B. Advice to gargle with tap water and not to gargle green tea during study A. and B. Advice to gargle at least 3 times/day (after arriving at school, after lunch, and after school) Consumption of green tea and other tea was not restricted for either group. Safety monitoring carried out throughout the study (not further described).	Materials supplied by researchers. High schools’ vice principals and head teachers assisted with safety monitoring.	Green tea supplied individually to students. Mode of gargling advice not described.	High schools in Japan	Gargling 3 times/day for 90 days	None described.	None described.	Daily questionnaire included questions about daily adherence to gargling regimen. Adherence rate of gargling at or above 75%, and absence of green tea gargling when in the water gargling group.	Gargling adherence rate: green tea group: 73.7%; water group: 67.2%
Satomura 2005	2 active interventions: A. Water gargling B. Povidone‐iodine gargling	Healthy adults	Prevent URTIs through gargling water alone, which may wash out pathogens from the pharynx and oral cavity through whirling water or through chlorine, or povidone‐iodine for its perceived virucidal properties	A. Water B. 15 to 30 times diluted 7% povidone‐iodine (as indicated by manufacturer)	Local administrators instructed participants to: ‐ gargle dose of water or povidone‐iodine 3 times/day; ‐ maintain hand‐washing routine; ‐ not change other hygiene habits; ‐ not take any cold remedies; ‐ complete gargling diary. Weekly monitoring of hygienic actions and encouragement to keep up assigned intervention every week	Local project administrators (18 healthcare professionals) provided instructions and monitoring and encouragement.	Not specified, but likely to have been face‐to‐face and individually, at least initially for instructions	18 healthcare sites in Japan (4 in northern region, 9 in central region, 5 in western region)	60 days overall 1. Water gargling: 20 mL for 15 s at least 3 times/day 2. Povidone‐iodine gargling: 20 mL of dilution 3 times/day	If diluted povidone‐iodine caused serious discomfort or was not available, participants were allowed to gargle with water instead.	3 participants assigned to povidone‐iodine gargled with water instead as the povidone‐iodine “did not agree with them”.	Completion of gargling diary: frequency of gargling and hand‐washing Weekly monitoring and encouragement by local administrators	9 participants did not complete diary. Average frequency of gargling / person / day: With water: A: 3.6 B: 0.8 Control: 0.9 With povidone‐iodine: A.: < 0.1 B: 2.9 Control: 0.2

ABH: alcohol‐based rub AGNPs: ARGOVIT silver nanoparticles ARI: acute respiratory infection CDC: Centers for Disease Control and Prevention CG: control group CHG: chlorhexidine gluconate CHW: community health worker CO: carbon monoxide DCCs: daycare centres DCT: daily contact testing FM: face masks H: handwashing HCP: healthcare personnel HCW: healthcare worker HH: hand hygiene HSG: hand sanitiser group HSW: hand‐washing with soap and water HW: hand‐washing HWWS: hand‐washing with soap IG: intervention group IHIP: integrated environmental home‐based intervention package ILI: influenza‐like illness IU: international units LFD: lateral flow device LNS: lipid‐based nutrient supplements LTCFs: long‐term care facilities m: metre min: minute N: nutrition NGOs: non‐governmental organisations NH: nursing home NHS: National Health Service no.: number NPIs: non‐pharmaceutical interventions PCR: polymerase chain reaction PM2.5: particulate matter of less than 2.5 microns RAs: research assistants RIs: respiratory infections RTIs: respiratory tract infections S: sanitation SD: standard deviation SES: electrolysed water SSTI: skin and soft‐tissue infection SWG: soap‐and‐water group TCID: tissue‐culture infectious dose URTI: upper respiratory tract infection W: water WHO: World Health Organization wk: week WSH: combined water, sanitation and handwashing WSHN: combined water, sanitation, handwashing and nutrition w/w: weight for weight  [1] Filtration efficiency testing was conducted using a Fluke 985 particle counter (volumetric sampling rate of 2.83 litres/ minute. The measurement was taken of particles 0.3–0.5 μm in diameter flowing through the material with a face velocity of 8.5 cm/s. Internal testing found that cloth masks with an external layer made of Pellon 931 polyester fusible interface ironed onto interlocking knit with a middle layer of interlocking knit could achieve a 60% filtration efficiency. Upon discussions with the manufacturers, the researchers learned that those materials could not be procured. Using materials that were available, the highest filtration efficiency possible was 37%. [2] “the exterior and interiors were spunbond and the middle layer was meltblown” [3] 10 times with bar soap and water [4] Featured the Honorable Prime Minister of Bangladesh Sheikh Hasina, the head of the Imam Training Academy, and the national cricket star Shakib Al Hasan. [5] A grassroots organization with a network of volunteers across the country [6] “consistent with the WHO guideline that defines physical distancing as one meter of separation.” www.who.int/westernpacific/emergencies/covid-19/information/physical-distancing (accessed 13 June 2022). [7] Occupational Safety and Health Administration (OSHA). OSHA technical manual: section VIII: chapter 2: respiratory protection. US Department of Labor. www.osha.gov/dts/osta/otm/otm_viii/otm_viii_2.html (accessed 21 April 2020). [8] Ministry of Health and Long‐Term Care, Public Health Division, Provincial Infectious Diseases Advisory Committee. Preventing respiratory illnesses: protecting patient and staff: infection control and surveillance standards for febrile respiratory illness (FRI) in non‐outbreak conditions in acute care hospitals [September 2005] http://www.health.gov.on.ca/english/providers/program/infectious/diseases/best_prac/bp_fri_080406.pdf (accessed September 11 2009). [URL inactive] [9] Before eating, after sneezing, coughing, handling money, using restroom, returning to desk and interacting with others who may be sick [10] after coming into classroom, before and after lunch, after break, after physical education, when they went home and after coughing, sneezing or blowing their noses [11] after toileting and when visibly dirty plus a protocol for particular circumstances: after coming into the classroom; before and after lunch; after playing outside; when they went home; after coughing, sneezing, or blowing their noses; and after diapering [12] 1) when entering into the classroom; 2) after sneezing, coughing, or blowing their nose; 3) after using the toilet/washroom; 4) before eating any food; and 5) when leaving the school at the end of the day [13] what to do if hands were dirty, why students should wash their hands, benefits of washing hands and using hand sanitiser, procedure for washing hands using hand sanitiser, to cover mouth and nose with upper part of sleeve while coughing and/or sneezing [14] Boyce JM, Pittet D, Healthcare Infection Control Practices Advisory Committee, HICPAC/ SHEA/APIC/IDSA Hand Hygiene Task Force. Guideline for hand hygiene in healthcare settings. Recommendations of the Healthcare Infection Control Practices Advisory Committee and the HICPAC/SHEA/APIC/ IDSA Hand Hygiene Task Force. MMWR Recommendations and Reports 2002;51(RR‐16):1–45. www.cdc.gov/mmwr/preview/mmwrhtml/rr5116a1.htm (accessed 21 April 2020). International Bank for Reconstruction and Development/ World Bank, Bank‐Netherlands Water Partnership, Water and Sanitation Program. Hand washing manual: a guide for developing a hygiene promotion program to increase handwashing with soap. http://go.worldbank.org/PJTS4A53C0 (Accessed 16 May 2007). *[URL inactive]* California State Department of Education. *Techniques for Preventing the Spread of Infectious Diseases*. Sacramento (CA): California State Department of Education, 1983. Geiger BF, Artz L, Petri CJ, Winnail SD, Mason JW. *Fun with Handwashing Education*. Birmingham (AL): University of Alabama, 2000. Roberts A, Pareja R, Shaw W, Boyd B, Booth E, Mata JI. A tool box for building health communication capacity. www.globalhealthcommunication.org/tools/29 (Accessed 10 October 2007). *[URL inactive]* Stark P. *Handwashing Technique. Instructor’s Packet. Learning Activity Package*. Sacramento (CA): California State Department of Education, 1982. [15] DIN EN 1500: Chemische Desinfektionsmittel und Antiseptika, Hygienische Händedesinfektion, Prüfverfahren und Anforderungen (Phase 2/Stufe 2). Brüssel (Belgium): CEN, European Comittee for Standardization 1997;1‐20. [16] DIN EN 12791: Chemische Desinfektionsmittel und Antiseptika, Chirugische Händedesinfektionsmittel ‐ Prüfverfahren und Anforderungen (Phase 2/Stufe 2). Brüssel (Belgium): CEN, European Comittee for Standardization 2005;1‐31. [17] after defaecation, after cleaning an infant who had defaecated, before preparing food, before eating, and before feeding infants [18] non‐governmental organisation that supports community‐based health and development initiatives [19] “Healthy Hands” Rules (from Figure 3 in paper): Do use “special soap” when arrive to school, before lunch, after go to bathroom (only if soap and water not available), if rub nose or eyes or if fingers in mouth, if teacher asks. Do not: use “special soap” if hand dirt on them, put “special soap” on another student, play with ‘special soap”, put hands near eyes after using “special soap”. [20] Calculated by subtracting each day’s soap weight from the previous day’s weight. Maximum number of grams of soap consumed for each compound was identified and the day on which the maximum soap consumption was recorded. A per capita estimate of daily soap consumption was calculated [21] National Health and Medical Research Council. Staying Healthy in Child Care. Canberra (Australia): Australian Government Publishing Service, 1994 [22] upon arrival, before and after lunch, and prior to departure [23] World Health Organization. (‎2012)‎. Hand hygiene in outpatient and home‐based care and long‐term care facilities: a guide to the application of the WHO multimodal hand hygiene improvement strategy and the “My Five Moments For Hand Hygiene” approach. World Health Organization. apps.who.int/iris/handle/10665/78060 (accessed 15 June 2022) [24] Moment 1 (before touching a resident) = Room In; Moment 4 (after touching a resident) and Moment 5 (after touching a resident’s surroundings) = Room Out; Moment 2 (before a clean/antiseptic procedure) = Before Clean; Moment 3 (after body fluid exposure risk) – After Dirty [25] Handsome: handhygiëne in verpleeghuizen.: Zorg voor beter; 2019 May 03. URL: www.zorgvoorbeter.nl/handsome (accessed 7 June 2022) [26] Veiligheid en Kwaliteit: Project Handen uit de Mouwen.: Stichting Samenwerkende Rijnmond Ziekenhuizen [27] Auditor training.: Hand Hygiene Australia URL: www.hha.org.au/audits/auditor-training (accessed 7 June 2022) [28] no long nails, acrylic nails, or polished nails and not wearing a ring, bracelet, wristwatch, brace, or long sleeves. [29] Persoonlijke hygiëne: Verpleeghuizen, woonzorgcentra, voorzieningen voor kleinschalig wonen voor ouderen.: Werkgroep Infectie Preventie; 2014. URL: tinyurl.com/wpfqr8p (accessed 7 June 2022) [30] knowledge and awareness of HH guidelines, perceived importance of performing HH, perceived behavioural control (i.e. perceived ease or difficulty of performing the behaviour), and habit [31] “According to the Dutch national guidelines, HH is mandatory for caregivers before touching/preparing food, before caregivers themselves ate or assisted children with eating, and before wound care; and after diapering, after toilet use/wiping buttocks, after caregivers themselves coughed/sneezed/wiped their own nose, after contact with body fluids (e.g. saliva, vomit, urine, blood, or mucus when wiping children’s noses), after wound care, and after hands were visibly soiled.” (p. 2495) [32] Having touched household items being used by the index patients and/or other symptomatic household contacts, and after coughing/sneezing, before meals, before preparing meals and when returning home [33] Which addresses “contextual, psychosocial, and technological factors at the societal, community, interpersonal, individual, and habitual levels”. (Luby 2018) [34] Hussain F, Luby SP, Unicomb L, Leontsini E, Naushin T, Buckland AJ, et al. Assessment of the acceptability and feasibility of child potties for safe child feces disposal in rural Bangladesh. The American Journal of Tropical Medicine and Hygiene. 2017;97: 469–76. [35] Sultana R, Mondal UK, Rimi NA, Unicomb L, Winch PJ, Nahar N, et al. An improved tool for household faeces management in rural Bangladeshi communities. Tropical Medicine & International health 2013;18: 854–60. [36] Hulland KR, Leontsini E, Dreibelbis R, Unicomb L, Afroz A, Dutta NC, et al. Designing a handwashing station for infrastructure‐restricted communities in Bangladesh using the integrated behavioural model for water, sanitation and hygiene interventions (IBM‐WASH). BMC Public Health 2013; 13: 877. [37] Menon P, Nguyen PH, Saha KK, Khaled A, Sanghvi T, Baker J, et al. Combining intensive counseling by frontline workers with a nationwide mass media campaign has large differential impacts on complementary feeding practices but not on child growth: results of a cluster‐randomized program evaluation in Bangladesh. The Journal of Nutrition 2016;146:2075–84. [38] comprised of: senior program manager‐intervention delivery, senior program manager‐operations, Sanitation Intervention Team leader, senior field research officer, training officer, field research officers, CHW supervisors and CHWs [39] SODIS: www.sodis.ch/index_EN.html [40] after defecation, after changing diapers, before food preparation and before eating [41] 1. Wash both hands with water and soap before eating/ handling food 2. Wash both hands with water and soap/ash after defecation 3. Wash both hands with water and soap/ash after cleaning baby’s bottom 4. Use hygienic latrine by all family members including Children 5. Dispose of children’s faeces into hygienic latrines 6. Clean and maintain latrine 7. Construct a new latrine if the existing one is full and fill the pit with soil/ash. 8. Safe collection and storage of drinking water 9. Draw drinking water from arsenic safe water point 10. Wash raw fruits and vegetables with safe water before eating and cover food properly 11. Manage menstruation period safely (p.605) [42] Rosenstock IM, Strecher VJ, Becker MH. Social learning theory and the Health Belief Model. Health Education Quarterly 1988;15:175–83. [43] Glanz K, Rimer BK, 2005. Theory at a Glance: A Guide for Health Promotion Practice. Washington, DC:US Department of Health and Human Services, Public Health Service, National Institutes of Health, National Cancer Institute. [44] Hovland CI, Janis IL, Kelley HH, 1953. Communication and Persuasion; Psychological Studies of Opinion Change. New Haven, CT: Yale University Press. [45] Based on family of five, consuming 2L of water per person per day, the bottle would last almost a year [46] W: key concepts for water treatment and contamination, procedures for refilling dispenser and distributing bottled chlorine, chlorine testing and reporting; H: HW with soap at critical times and creating supportive environment; S: contamination pathways; N: early initiation and exclusive breastfeeding, complementary and supplementary feeding, LNS procedures for collection from health facility and delivery tracking, teaching mamas how to feed Mwanzobora to the child, cooking demonstration, age‐specific messaging about nutrition [47] Department of Health and Social Care. Lateral flow device performance data. July 7, 2021. www.gov.uk/government/publications/lateral-flow-device-performance-data (accessed 15 June 2022). [48] “applicable to schools as defined in national guidelines were, face to face contact (within 1 metre for any length of time) or skin to skin contact or someone the case coughed on; or within 1 metre for ≥1 minute; or within 1‐2 metres for >15 minutes.” P.2 of Supplementary appendix [49] i.e., surgical uniform, N95 mask, eye‑sealing glasses and plastic wallet, disposable cap, latex gloves, rubber footwear for hospital use and disposable shoe covers, while working. Additionally, third level care health professionals wore a full protective mask, Dermacare®, overalls with zipper, and an integrated hood with elastic hand and ankle cuffs, double disposable boot covers and double latex gloves. [50] With liquid soap (2% chlorhexidine gluconate) and hand disinfection (0.05% chlorhexidine gluconate and 60‑80% ethyl alcohol). [51] With 80% ethyl alcohol

#### Assessment of risk of bias in included studies

Four review authors (EF/EB/GB/MJ) independently assessed risk of bias for the method of random sequence generation and allocation concealment (selection bias), blinding of participants and personnel (performance bias), blinding of outcome assessment (detection bias), outcome reporting (attrition bias), and selective reporting (reporting bias). In addition, for the cluster trials, we assessed selection bias due to how recruitment of participants was conducted. Participants should be identified before the cluster is randomised or, if not, recruitment should be by someone masked to the cluster allocation. Further, we considered whether there were sufficient numbers of clusters in each treatment group to ensure comparable groups, and excluded one study from the analysis due to insufficient number of clusters. We used the Cochrane risk of bias tool to assess risk of bias, classifying each risk of bias domain as 'low', ‘high’, or ‘unclear’. The following were indications for low risk of bias:

1. method of random sequence generation: the method was well‐described and is likely to produce balanced and truly random groups;
2. allocation concealment: the next treatment allocation was not known to participant/cluster or treating staff until after consent to join the study;
3. blinding of participants and personnel: the method is likely to maintain blinding throughout the study;
4. blinding of outcome assessors: all outcome assessors were unaware of treatment allocation;
5. outcome reporting: participant attrition throughout the study is reported, and reasons for loss are appropriately described; and
6. selective reporting: all likely planned and collected outcomes have been reported.

#### Measures of treatment effect

When possible, we performed meta‐analysis and summarised effectiveness as risk ratio (RR) using 95% confidence intervals (CIs). For studies that could not be pooled, we used the effect measures reported by the trial authors (such as RR or incidence rate ratio (IRR) with 95% CI or, when these were not available, relevant P values). Where multiple analyses were reported on the same outcome we chose the analysis based on preferences for: (1) an adjusted analysis (over an unadjusted analysis), and (2) an analysis based on a longer follow‐up period, or a greater number of outcomes events.

#### Unit of analysis issues

Many of the included studies were cluster‐RCTs. To avoid any unit of analysis issues, we only included treatment effect estimates that were based on methods that were appropriate for the analysis of cluster trials, such as mixed models and generalised estimating equations. Given this restriction, we used the generalised inverse‐variance method of meta‐analysis. Some cluster‐RCTs that did not report cluster‐adjusted treatment effects provided sufficient data (number of events and participants by treatment group and intraclass correlations) for us to calculate appropriate treatment effect estimates and standard errors using the methods described in the *Cochrane Handbook for Systematic Reviews of Interventions* (Higgins 2021a). For studies with multiple treatment groups but only one control group, where appropriate, we adjusted standard errors upwards to avoid unit of analysis errors in the meta‐analyses. We did this by splitting the control group into equal sized groups and adjusting standard errors upwards to account for the reduced sample size of the control subgroups (Higgins 2021b).

#### Dealing with missing data

Previously, whenever details of studies were unclear, or studies were only known to us by abstracts or communications at meetings, we corresponded with first or corresponding authors. For this 2022 review, we did not contact authors of studies. 

#### Assessment of heterogeneity

Aggregation of data was dependent on types of comparisons, sensitivity and homogeneity of definitions of exposure, populations and outcomes used. We calculated the I²statistic and Chi² test for each pooled estimate to assess the presence of statistical heterogeneity (Higgins 2002; Higgins 2003).

#### Assessment of reporting biases

Given the widely disparate nature of our evidence base, we limited our assessment of possible reporting biases to funnel plot visual inspection if we had > 10 included studies for any single meta‐analysis.

#### Data synthesis

If possible and appropriate, we combined studies in a meta‐analysis. We used the generalised inverse‐variance random‐effects model where cluster‐RCTs were included in the analysis. We chose the random‐effects model because we expected clinical heterogeneity due to differences in pooled interventions and outcome definitions, and methodological heterogeneity due to pooling of RCTs and cluster‐RCTs.

#### Subgroup analysis and investigation of heterogeneity

We conducted one post hoc subgroup analyses of adults (18 years +) versus children (0 to 18 years) for the comparison of hand hygiene versus control.

We did not conduct further investigation of heterogeneity due to insufficient numbers of studies included in the comparisons. 

#### Sensitivity analysis

We conducted a sensitivity analysis for hand hygiene versus control where we included the most precise and unequivocal measure of viral respiratory illness reported for each included study.

#### Summary of findings and assessment of the certainty of the evidence

We created three summary of findings tables using the following outcomes: numbers of cases of viral respiratory illness (including ARIs, ILI, COVID‐like illness and laboratory‐confirmed influenza/SARS‐CoV‐2 or other respiratory viruses), and adverse events related to the intervention (Table 1; Table 2; Table 3). We planned to include the secondary outcomes of deaths; severity of viral respiratory illness as reported in the studies; absenteeism; hospital admissions; and complications related to the illness (e.g. pneumonia). However, these data were poorly reported in the included studies. We used the five GRADE considerations (study limitations, consistency of effect, imprecision, indirectness, and publication bias) to assess the certainty of evidence as it related to the studies which contributed data to the meta‐analyses for the prespecified outcomes (Atkins 2004). We used the methods and recommendations described in Section 8.5 and Chapter 12 of the *Cochrane Handbook for Systematic Reviews of Interventions* (Higgins 2011), employing GRADEpro GDT software (GRADEpro GDT). We justified all decisions to down‐ or upgrade the certainty of the evidence in footnotes, and made comments to aid the reader's understanding of the review where necessary.

## Results

### Description of studies

See Characteristics of included studies and Characteristics of excluded studies tables. Five trials were funded by government and pharmaceutical companies (Aiello 2010; Aiello 2012; Chard 2019; Yeung 2011; Zomer 2015), and nine trials were funded by pharmaceutical companies (Arbogast 2016; Carabin 1999; Luby 2005; Nicholson 2014; Sandora 2005; Sandora 2008; Turner 2004a; Turner 2004b; Turner 2012).

#### Results of the search

For this 2022 update we found 2667 records through database and trial registry searching, as well as 738 record through citation searching. After removing duplicates we had 2936 records that underwent title and abstract screening.  We identified a total of 202 titles in this 2022 update. We excluded 180 titles and retrieved the full papers of 35 studies, to include 11 new studies. See Figure 1.

**1:**
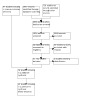
Study flow diagram.

#### Included studies

In this 2022 update we included 11 new studies (610,872 participants); randomised controlled trials (RCTs) (n = 5) or cluster‐RCTs (n = 6) published between 2020 and 2022. In total 78 studies are included in this review update. For detailed descriptions of the interventions of the included studies, see Table 4.

Eighteen trials focused on using masks (Abaluck 2022; Aiello 2010; Aiello 2012; Alfelali 2020; Barasheed 2014; Bundgaard 2021; Canini 2010; Cowling 2008; Ide 2016; Jacobs 2009; Loeb 2009; MacIntyre 2009; MacIntyre 2011; MacIntyre 2013; MacIntyre 2015; MacIntyre 2016; Radonovich 2019; Suess 2012). Thirteen of the 18 trials compared medical/surgical masks to no mask (control) (Abaluck 2022; Aiello 2010; Aiello 2012; Alfelali 2020; Barasheed 2014; Bundgaard 2021; Canini 2010; Cowling 2008; Jacobs 2009; MacIntyre 2009; MacIntyre 2015; MacIntyre 2016; Suess 2012). One study compared catechin‐treated masks to no mask (Ide 2016), and one study included cloth masks versus control (third arm in MacIntyre 2015). Three of the 18 trials were in healthcare workers (Ide 2016; Jacobs 2009; MacIntyre 2015), whilst the remaining trials were in non‐healthcare workers (students, households, families, or pilgrims). Only one trial was conducted during the H1N1 pandemic season (Suess 2012), and two trials were conducted during the SARS‐CoV‐2 pandemic (Abaluck 2022; Bundgaard 2021).

Five of the 18 trials compared N95 masks or P2 masks to medical/surgical masks (Loeb 2009; MacIntyre 2009; MacIntyre 2011; MacIntyre 2013; Radonovich 2019). All of these trials, except for one study that was conducted on household individuals (MacIntyre 2009), included healthcare workers either in a hospital setting, Loeb 2009; MacIntyre 2011; MacIntyre 2013, or an outpatient setting (MacIntyre 2009; Radonovich 2019).

One trial evaluated the effectiveness of quarantining workers of one of two sibling companies in Japan whose family members had developed an influenza‐like illness (ILI) during the 2009 to 2010 H1N1 influenza pandemic (Miyaki 2011). Another trial conducted during the SARS‐CoV‐2 pandemic in Norway investigated fitness centre access with physical distancing compared to no access (Helsingen 2021); and one cluster trial compared daily testing for contacts of individuals with SARS‐CoV‐2 compared to self‐isolation at home in English secondary schools (Young 2021). 

Nineteen trials compared hand hygiene interventions with no hand hygiene (control) and provided data suitable for meta‐analysis. The populations in these trials included adults, children, and families, in settings such as schools (Biswas 2019; Stebbins 2011), childcare centres (Azor‐Martinez 2018; Correa 2012; Roberts 2000; Zomer 2015), homes/households (Cowling 2008; Cowling 2009; Larson 2010; Little 2015; Nicholson 2014; Ram 2015; Sandora 2005; Simmerman 2011), offices (Hubner 2010), military trainees (Millar 2016), villages (Ashraf 2020; Swarthout 2020), and nursing homes (Teesing 2021). None of the trials were conducted during a pandemic, although some of the studies were conducted during peak influenza seasons. 

A further 10 trials that compared a variety of hand hygiene modalities to control provided insufficient information to include in meta‐analyses. Three trials were in children: one was conducted in daycare centres in Denmark examining a multimodal hygiene programme (Ladegaard 1999), and two trials compared a hand hygiene campaign or workshop in an elementary school environment in Saudi Arabia, Alzaher 2018, and Egypt, Talaat 2011. Three trials tested virucidal hand treatment in an experimental setting, Gwaltney 1980; Turner 2004a, and in a community, Turner 2012, in the USA. Feldman 2016 compared hand‐washing with chlorhexidine gluconate amongst Israeli sailors. One trial compared hand sanitiser packaged in a multimodal hygiene programme amongst office employees in the USA (Arbogast 2016). Two trials were conducted in a long‐term facility setting: one trial examined the effect of a bundled hand hygiene programme on infectious risk in nursing home residents in France (Temime 2018), and the other trial compared the effect of using hand sanitisers in healthcare workers on the rate of infections (including respiratory infections) in nursing home residents in Hong Kong (Yeung 2011).

Five trials compared different hand hygiene interventions in a variety of settings such as schools (Morton 2004, in kindergartens and elementary schools in the USA; Priest 2014, in primary schools in New Zealand; and Pandejpong 2012 in kindergartens in Thailand). One study was conducted in low‐income neighbourhoods in Karachi, Pakistan (Luby 2005), and one was conducted in a workplace environment in Finland (Savolainen‐Kopra 2012). A variety of interventions were used across these trials such as soap and water (Luby 2005; Savolainen‐Kopra 2012), hand sanitiser (Morton 2004; Pandejpong 2012; Priest 2014; Savolainen‐Kopra 2012), body wash (Luby 2005), and alcohol‐based hand wipes (Morton 2004), with or without additional hygiene education. There was considerable variation in interventions, and the information in the trial reports was insufficient to permit meta‐analysis.

Seven trials compared a combined intervention of hand hygiene and face masks with control. Four of these trials were carried out in households in Germany (Suess 2012), Thailand (Simmerman 2011), Hispanic immigrant communities in the USA (Larson 2010), and households in Hong Kong (Cowling 2009). Two trials were conducted amongst university student residences (Aiello 2010; Aiello 2012), and two trials in groups of pilgrims at the annual Hajj (Aelami 2015; Alfelali 2020). Moreover, six trials evaluated the incremental benefit of combining surgical masks in addition to hand hygiene with soap (Simmerman 2011), hand sanitiser (Aiello 2010; Aiello 2012; Larson 2010; Suess 2012), or both (Cowling 2009), versus mask or hand hygiene alone on the outcomes of ILI and influenza. Aelami 2015 investigated a hygienic package (alcohol‐based hand rub (gel or spray), surgical masks, soap, and paper handkerchiefs) with a control group.

Seven trials compared a multimodal combination of hand hygiene and disinfection of surfaces, toys, linen, or other components of the environment with a control (Ban 2015; Carabin 1999; Ibfelt 2015; Kotch 1994; McConeghy 2017; Sandora 2008; White 2001). Variation in scope and type of interventions and insufficient data in trial reports precluded meta‐analysis. All studies except for one were in children (McConeghy 2017), which was in a nursing home population).

Three trials included in two papers investigated the role of virucidal tissues in interrupting transmission of naturally occurring respiratory infections in households (Farr 1988a; Farr 1988b; Longini 1988). Four cluster‐RCTs implemented complex, multimodal sanitation, education, cooking, and hygiene interventions (Chard 2019; Hartinger 2016; Huda 2012; Najnin 2019). All four of these trials were conducted in low‐income countries in settings with minimal to no access to basic sanitation.

Three trials assessed the effect of gargling on the incidence of upper respiratory tract infections (URTIs) or influenza: gargling with povidone‐iodine (Satomura 2005), green tea (Ide 2014), and tap water (Goodall 2014). Two trials investigated the use of mouth/nasal washes on the incidence of SARS‐CoV‐2 infection in healthcare workers during the COVID‐19 pandemic (Almanza‐Reyes 2021; Gutiérrez‐García 2022). One trial investigated the use of glasses against the transmission of SARS‐CoV‐2 (Fretheim 2022a).

##### Ongoing studies

We identified four ongoing studies during the course of the COVID‐19 pandemic, of which one is completed, but unreported (NCT04471766). The trials evaluated masks concurrent with the COVID‐19 pandemic. Three trials on other interventions are ongoing (Brass 2021; NCT03454009; NCT04267952).

##### Studies awaiting classification

We identified five studies awaiting classification (Contreras 2022; Croke 2022; Delaguerre 2022; Loeb 2022; Varela 2022). 

A previous RCT (NCT04296643) reported as ongoing in the last version has now been recently published but was not able to be included in the summary of findings pooled results (Loeb 2022). In a multicentre, randomised non‐inferiority trial of 1009 healthcare workers (HCWs) across four countries randomised to medical mask versus fit‐tested N95 respirators for direct care of COVID‐19 patients or long‐term care residents, laboratory‐confirmed SARS‐CoV‐2 was found in 10.46% (52/497) versus 9.27% (47/507) in the medical/surgical mask group and fit‐tested N95 respirator group (hazard ratio 1.14 (95% CI 0.77 to 1.69), respectively. There was a 1.19% absolute increase in risk of COVID‐19 with medical masks versus N95 respirator  95% CI (‐2.5% to 4.9%). There were 47 (10.8%) adverse events related to the intervention reported in the medical mask group and 59 (13.6%) in the N95 respirator group. The use of medical masks was found to be non‐inferior to N95 respirators in the direct care of COVID‐19 patients and the study crossed over into the more transmissible Omicron variant period of the COVID‐19 pandemic. 

#### Excluded studies

We excluded a total of 180 studies. We identified 20 new studies for exclusion at the data extraction stage of this 2022 update, all of which appeared to be eligible at screening. Five of the 20 studies were ineligible due to evaluating treatments for patients with disease (Cyril Vitug 2021; Ferrer 2021; Meister 2022; Sanchez Barrueco 2022; Sevinc Gul 2022), two were excluded because they did not assess clinical outcomes (Costa 2021; Seneviratne 2021), four were excluded due to not assessing viral outcomes (Gharebaghi 2020; Giuliano 2021; Karakaya 2021; Kawyannejad 2020), five were excluded as they were experiments that did not measure any of our outcomes of interest (Ahmadian 2022; Dalakoti 2022; Egger 2022; Malaczek 2022; Montero‐Vilchez 2022); three were excluded because they were not RCTs (Chen 2022; Lim 2022; Mo 2022), and one was excluded as it was a report of another study (Munoz‐Basagoiti 2022).

### Risk of bias in included studies

The overall risk of bias is presented graphically in Figure 2 and summarised by included study in Figure 3. Details on the judgements can be found in the descriptions of individual included studies (Characteristics of included studies table). Out of 78 included studies, only two were rated as low risk of bias for all domains. One of those studies compared two different types of masks (Radonovich 2019), and the other compared hand sanitiser to no treatment (Turner 2012). Notably, neither of these two studies was blinded, however, trial procedures were sufficiently robust that the risk of performance bias was low. Overall,approximately only 20% of the studies were rated as low risk of performance bias. This risk of bias domain was particularly problematic because most interventions studied could not be blinded from participants and/or investigators. The two risks of bias domains that were rated the least problematic were attrition bias and random sequence generation where around 50% of studies were rated as low risk of bias. Allocation concealment, blinded outcome assessment and selective reporting were rated as low risk of bias for around 40% of the included studies. Many of the included studies were cluster‐RCTs where the randomisation process was not well reported leading to ratings of unclear risk of bias. 

**2:**
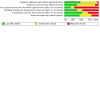
'Risk of bias' graph: review authors' judgements about each risk of bias item presented as percentages across all included trials.

**3:**
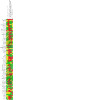
'Risk of bias' summary: review authors' judgements about each risk of bias item for each included trial.

#### Allocation

For this 2022 review, 10 of the 11 newly included studies provided adequate information on randomisation and were  judged to have low risk of bias (Abaluck 2022; Alfelali 2020; Almanza‐Reyes 2021; Ashraf 2020; Bundgaard 2021; Fretheim 2022a; Helsingen 2021; Swarthout 2020; Teesing 2021; Young 2021). Six of these studies described the use of a computerised random number generator (Almanza‐Reyes 2021; Bundgaard 2021; Helsingen 2021; Swarthout 2020; Teesing 2021; Young 2021). Almanza‐Reyes 2021 described the use of computer‐generated stratified block scheme, while Bundgaard 2021 reported the use of a computer algorithm stratified by the five regions of Denmark. In Fretheim 2022a, the investigators used a digital platform (Nettskjema) for recruitment, randomisation and allocation. Three studies mentioned the use of a random number generator, with no additional specifics (Helsingen 2021; Swarthout 2020; Teesing 2021), while Young 2021 mentioned that randomisation was performed in blocks of two and stratified using nine strata to ensure a sample representative of schools and colleges in England. Abaluck 2022 reported pairwise cross randomisation, whilst Ashraf 2020 reported using a block random number generator. Alfelali 2020 described using coin‐tossing by an individual who was not a member of the research team (i.e. a fellow pilgrim who was not a participant in the trial, a tour operator, or a medical volunteer). One study provided insufficient information to judge the sequence generation bias (Gutiérrez‐García 2022). 

The success of randomisation was judged as low risk of bias in one study only that used an off‐site investigator to allocate groups (Ashraf 2020). Four new studies provided insufficient information to make a judgment on the adequacy of the process (Bundgaard 2021; Swarthout 2020; Teesing 2021; Young 2021). The remaining six newly included studies were judged as high risk of allocation bias (Abaluck 2022; Alfelali 2020; Almanza‐Reyes 2021; Fretheim 2022a; Gutiérrez‐García 2022; Helsingen 2021). In Abaluck 2022, there was a significant difference in the numbers of households included in each treatment group, suggestive of a lack of allocation concealment. Alfelali 2020 used coin tossing, which can lead to a large imbalance. In Almanza‐Reyes 2021 baseline prognostic factors (vaccination and frequency of handwashing) were unbalanced between the two arms. In Fretheim 2022a, a higher number of participants used face masks in the intervention group. In Gutiérrez‐García 2022 there as a significant age difference between the two groups. Helsingen 2021 described assigning the randomised sequence by a member of the research team, with no further description.

For the review published in 2020, information on sequence generation was overall poorly reported in most of the  included studies. Nineteen of the included studies provided adequate information on the randomisation scheme and were judged as at low risk of bias (Aiello 2012; Azor‐Martinez 2016; Azor‐Martinez 2018; Biswas 2019; Canini 2010; Correa 2012; Ide 2014; MacIntyre 2015; MacIntyre 2016; Millar 2016; Najnin 2019; Radonovich 2019; Ram 2015; Simmerman 2011; Stebbins 2011; Suess 2012; Talaat 2011; Turner 2012; Zomer 2015). Nine studies described the use of computerised sequence generation program/software (Aiello 2012; Azor‐Martinez 2018; Biswas 2019; Canini 2010; Millar 2016; Najnin 2019; Radonovich 2019; Talaat 2011; Turner 2012). One study used random number tables for sequence generation (Azor‐Martinez 2016). Three studies described using the random function in Microsoft Excel (Microsoft Excel 2018) (Correa 2012; MacIntyre 2016; Suess 2012). Two studies used statistical software to generate a randomisation allocation (MacIntyre 2015; Priest 2014). Two studies reported using block randomisation: Ram 2015 used block randomisation, and an independent investigator‐generated the list of random assignments, whilst Simmerman 2011 performed block randomisation. Stebbins 2011 used constrained randomisation, and Zomer 2015 reported using stratified randomisation by means of computer generation with a 1:1 ratio in each of the strata. 

Fourteen studies reported insufficient information to permit a judgement on the adequacy of the process to minimise selection bias (Aelami 2015; Alzaher 2018; Arbogast 2016; Barasheed 2014; Chard 2019; DiVita 2011; Feldman 2016; Hubner 2010; Ibfelt 2015; McConeghy 2017; Miyaki 2011; Pandejpong 2012; Savolainen‐Kopra 2012; Yeung 2011). Six studies provided some description about sequence generation, but it was still unclear (Hartinger 2016; Huda 2012; Ide 2016; Little 2015; MacIntyre 2011; MacIntyre 2013). Huda 2012 mentioned random number tables, but it was unclear if this was for random selection or randomisation. Ide 2016 used computer‐generated randomisation, but the method was not stated. Hartinger 2016 used covariate‐constrained randomisation, but the method was not described. In Little 2015, participants were automatically randomly assigned by the intervention software, but the sequence generation was not described. Two studies used a secure computerised randomisation program (MacIntyre 2011; MacIntyre 2013), but the sequence generation was not described.

Three of the studies included in the 2020 review, were poorly randomised (Ban 2015; Nicholson 2014; Temime 2018). Ban 2015 included only two clusters, and the randomisation scheme was not reported. Nicholson 2014 used coin tossing, which can lead to a large imbalance. Temime 2018 used “simple randomisation” with no further description. 

For the RCTs included in previous versions of the review, three were poorly reported with no description of randomisation sequence or concealment of allocation (Gwaltney 1980; Turner 2004a; Turner 2004b). The quality of the cluster‐RCTs varied, with four studies not providing a description of the randomisation procedure (Carabin 1999; Kotch 1994; Morton 2004; White 2001). We rated seven studies as at low risk of bias for sequence generation (Cowling 2008; Cowling 2009; Luby 2005; Roberts 2000; Sandora 2005; Sandora 2008; Satomura 2005), and a further six studies as at unclear risk of bias (Farr 1988a; Farr 1988b; Ladegaard 1999; Loeb 2009; Longini 1988; MacIntyre 2009).

Many of the newly included cluster‐RCTs did not report adequately on allocation concealment. Twenty‐one of these studies reported adequate allocation and were judged as at low risk of bias (Aiello 2012; Alzaher 2018; Azor‐Martinez 2016; Azor‐Martinez 2018; Biswas 2019; Canini 2010; Chard 2019; Goodall 2014; Ide 2014; Ide 2016; Little 2015; MacIntyre 2011; MacIntyre 2015; Nicholson 2014; Priest 2014; Radonovich 2019; Ram 2015; Savolainen‐Kopra 2012; Stebbins 2011; Suess 2012; Turner 2012). Aiello 2012 randomised all residence houses in each of the residence halls prior to the intervention implementation. Alzaher 2018 allocated schools prior to all schoolgirls attending selected schools being invited to participate. Azor‐Martinez 2016 allocated schools/classes prior to children's recruitment. Azor‐Martinez 2018 assigned clusters prior to recruitment. Biswas 2019 completed the allocation prior to individuals being recruited. Chard 2019 allocated schools prior to individuals being recruited. Goodall 2014 used opaque, sealed, serially numbered envelopes that were only accessed when two study personnel were present. Ide 2014 also reported using individual drawing of sealed, opaque envelopes to randomly assign participants to the study groups. MacIntyre 2011 randomised hospitals prior to inclusion of participants. In MacIntyre 2015, hospital wards were randomised prior to recruitment of individuals. Nicholson 2014 used coin tossing to assign communities to intervention or control arms. Radonovich 2019 used constrained randomisation to resolve any potential imbalance between covariates between the trial arms. Four studies reported the use of central randomisation: Canini 2010 used central randomisation by employing an interactive voice response system; Ide 2016 used central randomisation services; Little 2015 participants were automatically randomly assigned by the intervention software; and Ram 2015 described a central allocation through data collectors notifying the field research officer, who consulted the block randomisation list to make the assignment of the household compound to intervention or control. Savolainen‐Kopra 2012 randomised clusters by matching prior to the onset of the interventions. Four studies reported that allocation was assigned by personnel (investigator, physician, or statistician) unaware of the randomisation sequence (Priest 2014; Stebbins 2011; Suess 2012; Turner 2012). Twenty‐two studies reported insufficient information to permit a judgement on the adequacy of the process to minimise selection bias (Aelami 2015; Arbogast 2016; Ban 2015; Barasheed 2014; Correa 2012; DiVita 2011; Feldman 2016; Hartinger 2016; Hubner 2010; Huda 2012; Ibfelt 2015; MacIntyre 2013; McConeghy 2017; Millar 2016; Miyaki 2011; Najnin 2019; Pandejpong 2012; Simmerman 2011; Talaat 2011; Temime 2018; Yeung 2011; Zomer 2015). Two studies provided some information about allocation, but it was not enough to permit a judgement on the risk of bias (Barasheed 2014; Simmerman 2011). Barasheed 2014 randomised pilgrim tents using an independent study co‐ordinator who was not an investigator, but did not describe how this was done. Simmerman 2011 described using a study co‐ordinator to assign households to the study arm (after consent was obtained). Only one of the newly added studies was judged as at high risk of bias, where the random assignment was allocated by doctors enrolling the participants (MacIntyre 2016). Of the previously included RCTs, 14 provided no or an insufficient description of concealment of allocation (Carabin 1999; Farr 1988a; Farr 1988b; Gwaltney 1980; Kotch 1994; Ladegaard 1999; Larson 2010; MacIntyre 2009; Morton 2004; Roberts 2000; Sandora 2008; Turner 2004a; Turner 2004b; White 2001). We assessed all of the remaining studies as at low risk of bias (Canini 2010; Cowling 2008; Cowling 2009; Loeb 2009; Longini 1988; LLuby 2005; Sandora 2005;Satomura 2005). Aiello 2010 used the drawing of a uniform ticket with the name of each hall out of a container and was rated as at high risk of bias.

#### Blinding

Although blinding is less of a concern in cluster‐RCTs, the risk of bias is substantial when the outcomes are subjective and the outcome assessor is not blinded. 

In this 2022 review, five RCTs (Almanza‐Reyes 2021; Bundgaard 2021; Fretheim 2022a; Gutiérrez‐García 2022; Helsingen 2021), and six cluster‐RCTs were all judged to have a high risk of detection bias (Abaluck 2022; Alfelali 2020; Ashraf 2020; Swarthout 2020; Teesing 2021; Young 2021). 

We judged two of the newly included studies to have a low risk of detection bias as the outcome is laboratory‐confirmed (Alfelali 2020; Gutiérrez‐García 2022). One study provided insufficient information to enable judgment (Almanza‐Reyes 2021). The remaining eight of the 11 new studies have a high risk of detection bias (Abaluck 2022; Ashraf 2020; Bundgaard 2021; Fretheim 2022a; Helsingen 2021; Swarthout 2020; Teesing 2021; Young 2021). In Abaluck 2022, investigators dropped individuals for whom symptom data were missing. In addition, other outcomes were subjective and can be influenced by the unblinded mask promoters, and mask surveillance staff. Moreover, blood testing in the protocol specified baseline testing which was not done, and no further explanation was provided. In Ashraf 2020, although the data collection team was separate from the intervention team, they were not blinded, and the outcome was respiratory illness measured through caregiver‐reported symptoms. In Bundgaard 2021, case detection was based on patient‐reported symptoms on home tests. In Fretheim 2022a, the outcome was self‐reported positive COVID‐19 test result, notified to the Norwegian Surveillance System for Communicable Diseases (MSIS). However, the public policy requiring confirmatory PCR‐test had changed during the study, which may have affected reporting. In Helsingen 2021, although the outcome was a positive test for COVID‐19 based on SARS‐CoV‐2 ribonucleic acid, the samples were collected and sent by participants, and there was a difference in adherence in testing between the two groups. Swarthout 2020, Teesing 2021, and Young 2021 all had subjective outcomes and assessors were not blinded. As for the detection bias,  six of the newly included studies were considered to have a high risk of detection bias (Bundgaard 2021; Gutiérrez‐García 2022; Helsingen 2021; Swarthout 2020; Teesing 2021; Young 2021. In Bundgaard 2021, case detection was based on patient‐reported symptoms and results from home point‐of‐care (POCT) testing. The primary outcome of Gutiérrez‐García 2022 was participants' self‐reported symptoms. Case detection in Helsingen 2021 was based on a home‐test kit. Swarthout 2020, Teesing 2021, and Young 2021 had subjective outcomes.

In the 2020 review, we judged 36 studies to have a high risk of bias (Aiello 2012; Abaluck 2022; Alfelali 2020; Almanza‐Reyes 2021; Alzaher 2018; Arbogast 2016; Ashraf 2020; Azor‐Martinez 2016; Azor‐Martinez 2018; Ban 2015; Biswas 2019; Bundgaard 2021; Carabin 1999; Chard 2019; Correa 2012; Cowling 2008; Gutiérrez‐García 2022; Helsingen 2021; Ide 2014; Kotch 1994; Ladegaard 1999; Little 2015; MacIntyre 2011; MacIntyre 2015; MacIntyre 2016; McConeghy 2017; Najnin 2019; Nicholson 2014; Ram 2015; Sandora 2008; Savolainen‐Kopra 2012; Swarthout 2020; Teesing 2021; Temime 2018; Young 2021; Zomer 2015). We assessed five cluster‐RCTs as at low risk of bias. Farr 1988a and Farr 1988b were double‐blinded studies and were judged as at low risk of bias. MacIntyre 2013 and Simmerman 2011 reported laboratory‐confirmed influenza, and blinding would not have affected the result. In Miyaki 2011 the self‐reported respiratory symptoms were confirmed by a physician.

We judged four cluster‐RCTs to have a low risk of detection bias because the outcome was laboratory‐confirmed influenza (Alfelali 2020; Barasheed 2014; Suess 2012), or physician‐confirmed ILI, Pandejpong 2012. Another two cluster‐RCTs were judged to have a low risk of bias because outcome assessors were blinded (Abaluck 2022; Ashraf 2020). One RCT (Almanza‐Reyes 2021) and two cluster‐RCTs (Talaat 2011; Yeung 2011) provided insufficient data to judge the effect of non‐blinding. Talaat 2011 included outcomes that were both self‐reported ILI and laboratory‐confirmed influenza. In Yeung 2011 the detection of cases was based on records for hospitalisation related to infection (including pneumonia). Eleven cluster‐RCTs were not blinded, but we judged the primary outcome to be unaffected by non‐blinding. Seven trials reported laboratory‐confirmed influenza (Aiello 2012; Cowling 2009; Larson 2010; Loeb 2009; MacIntyre 2009; Millar 2016; Stebbins 2011). Four studies reported self‐reported outcomes (Canini 2010; Priest 2014; Roberts 2000; Sandora 2008), but outcome assessors were not aware of the intervention assignment. Five RCTs were double‐blinded and were judged as at low risk of bias (Goodall 2014; Ide 2016; Longini 1988; Luby 2005; White 2001), whilst two studies were single‐blinded where investigators, Radonovich 2019, or laboratory personnel, Turner 2012, were blinded. Four RCTs were not blinded and were judged as at high risk of bias given the subjective nature of the outcome assessed (Hubner 2010; Ibfelt 2015; Jacobs 2009; Satomura 2005). Turner 2004a and Turner 2004b were double‐blind studies, but insufficient information was provided to assess the risk of bias.

#### Incomplete outcome data

In this 2022 review, six of the 11 newly included studies had reasonable attrition and provided sufficient evidence about participant flow throughout the study and reasons of loss to follow‐up, and hence were assessed as having a low risk of attrition bias (Alfelali 2020; Ashraf 2020; Bundgaard 2021; Fretheim 2022a; Gutiérrez‐García 2022; Swarthout 2020). Two studies provided insufficient information to assess the attrition risk (Almanza‐Reyes 2021; Teesing 2021). The remaining three studies were judged at high risk of attrition bias. In Abaluck 2022, laboratory testing results were only available for 40% of the symptomatic participants. In Helsingen 2021, more people in the control group withdrew from the study and reasons for withdrawal were not provided. In the Young 2021 study there was high attrition at different rates between the two groups.

In the 2020 review, we assessed 26 newly included trials as having a low risk of attrition bias, with sufficient evidence from the participant flow chart, and explanation of loss to follow‐up (which was minimal) similar between groups (Aiello 2012; Alzaher 2018; Arbogast 2016; Azor‐Martinez 2018; Barasheed 2014; Canini 2010; Chard 2019; Correa 2012; Goodall 2014; Hartinger 2016; Hubner 2010; Ide 2014; Ide 2016; MacIntyre 2011; MacIntyre 2013; MacIntyre 2015; MacIntyre 2016; Miyaki 2011; Pandejpong 2012; Radonovich 2019; Ram 2015; Simmerman 2011; Suess 2012; Turner 2012; Yeung 2011; Zomer 2015). Seven studies did not report sufficient information on incomplete data (attrition bias) (Aelami 2015; DiVita 2011; Feldman 2016; Hartinger 2016; Ibfelt 2015; McConeghy 2017; Priest 2014). Twelve studies had a high risk of attrition bias (Azor‐Martinez 2016; Ban 2015; Biswas 2019; Huda 2012; Little 2015; Millar 2016; Najnin 2019; Nicholson 2014; Savolainen‐Kopra 2012; Stebbins 2011; Talaat 2011; Temime 2018). In Azor‐Martinez 2016, attrition levels were high and differed between the two groups. Ban 2015 did not report on reasons for loss to follow‐up. Biswas 2019 did not provide information on missing participants (28 children in the control schools and two children in the intervention schools). Huda 2012 did not provide a flow diagram of study participants. Little 2015 had high attrition that differed between the two groups. Attrition in Millar 2016 differed amongst the three groups. In addition, ARI cases were captured utilising clinic‐based medical records for those participants who sought hospital care only. In Najnin 2019, there was high migration movement during the study, which could have distorted the baseline characteristics even more. There was no description of how such migration and changes in the intervention group were dealt with. In Nicholson 2014, households were removed from the study if they provided no data for five consecutive weeks. Although attrition was reported in Savolainen‐Kopra 2012, and 76% of volunteers who were recruited at the beginning of the reporting period completed the study, new recruits were added during the study to replace volunteers lost in most clusters. The total number of reporting participants at the end of the trial was 626 (91.7%) compared to the beginning, meaning that 15.7% of participants were replaced during the study. In Stebbins 2011,reasons for episodes of absence in 66% of the study participants were not reported. Talaat 2011 did not provide a flow chart of clusters flow during the study period and provided no information on withdrawal. Temime 2018 was greatly biased due to underreporting of outcomes in the control groups. Furthermore, no study flow chart was provided, and there was no reporting on any exclusions.

#### Selective reporting

For this 2022 review update, six of the 11 newly included studies reported all specified outcomes and were judged to have a low risk of selective reporting (Ashraf 2020; Bundgaard 2021; Fretheim 2022a; Gutiérrez‐García 2022; Helsingen 2021; Young 2021). Three studies had no published protocol and were considered to have an unclear risk of selective reporting (Alfelali 2020; Almanza‐Reyes 2021; Teesing 2021). The remaining two new included studies are considered to have a high risk of bias in this domain. Abaluck 2022 did not report on prespecified seroconversion, while in Swarthout 2020, none of the outcomes reported were prespecified in the trial registry.

In the 2020 review, 22 included studies reported all specified outcomes and were judged as at low risk of reporting bias (Aiello 2012; Barasheed 2014; Canini 2010; Chard 2019; Goodall 2014; Hartinger 2016; Ibfelt 2015; Ide 2016; Little 2015; MacIntyre 2011; MacIntyre 2013; MacIntyre 2015; MacIntyre 2016; Pandejpong 2012; Priest 2014; Radonovich 2019; Savolainen‐Kopra 2012; Simmerman 2011; Suess 2012; Temime 2018; Turner 2012; Zomer 2015). For 18 studies, it is unlikely that other outcomes were measured and not reported, although no protocol was available to assess reporting bias (Aelami 2015; Alzaher 2018; Arbogast 2016; Azor‐Martinez 2016; Azor‐Martinez 2018; Ban 2015; Biswas 2019; Correa 2012; DiVita 2011; Feldman 2016; Hubner 2010; Huda 2012; Ide 2014; Miyaki 2011; Nicholson 2014; Stebbins 2011; Talaat 2011; Yeung 2011). Three studies were at high risk of reporting bias (McConeghy 2017; Millar 2016; Najnin 2019). In McConeghy 2017, URTI was mentioned in the methods (the intervention presumably would have targeted these), but only lower respiratory tract infection (LRTI) and overall infection were reported. Millar 2016 was originally conducted for another purpose; we could not find the respiratory outcomes reported in the study as part of the original study protocol. In Najnin 2019, the published study protocol did not include respiratory illness as an outcome.

#### Other potential sources of bias

An additional consideration for cluster‐RCTs is identification/recruitment bias, where individuals are recruited in the trial after clusters are randomised. Such bias can introduce an imbalance amongst groups. 

In this 2022 review, of the six cluster‐RCTs included, we judged four to have a low risk of identification/recruitment bias (Abaluck 2022; Ashraf 2020; Swarthout 2020; Teesing 2021). In Abaluck 2022, all of people in the village were assigned to one study arm (control, cloth mask or surgical mask villages). In Ashraf 2020, participants were unaware of their intervention group assignment until after the baseline survey and randomisation. In Swarthout 2020, village clusters comprised of 12 enrolled households, while in Teesing 2021 randomisation was done per nursing home. Alfelali 2020 recruited individuals after cluster‐randomisation and is judged to have a high risk of recruitment bias, while in Young 2021, participation of students and staff contacts were made after random assignment of the school through written consent or electronic completion of a consent form.

Of the cluster‐RCTs included in our 2020 review, we judged 13 to have a low risk of identification/recruitment bias (Arbogast 2016; Biswas 2019; Canini 2010; Cowling 2008; Longini 1988; Luby 2005; MacIntyre 2015; MacIntyre 2016; Roberts 2000; Sandora 2005; Suess 2012; Temime 2018; White 2001). In Arbogast 2016, all identified individuals (office workers) were included in the assigned cluster. Schools were identified and then randomised to the clusters; students were then randomly selected from each classroom and school. Nine studies described the identification of participants, consenting/enrolling, and then randomising to the clusters (Canini 2010; Cowling 2008; Longini 1988; Luby 2005; MacIntyre 2015; MacIntyre 2016; Roberts 2000; Sandora 2005; White 2001). Suess 2012 identified and consented patients, then recruitment was performed by physicians unaware of cluster assignment. In Temime 2018, directors of the included nursing homes agreed to participate in the study before randomisation, and written consent was not required from the residents. 

Amongst the newly included studies, we judged four cluster‐RCTs as at low risk of identification/recruitment bias (Abaluck 2022; Swarthout 2020; Teesing 2021; Young 2021). In Abaluck 2022, the village was the unit of randomisation and all households received one arm of the study (control, surgical mask or cloth mask). In Swarthout 2020, village clusters were each randomised by blocks (group of nine adjacent clusters) into eight groups. In Teesing 2021 nursing homes were computer randomised after baseline hand hygiene measurements to either the intervention arm or the control arm. In Young 2021, schools were randomly assigned (1:1) to either a policy of offering contacts daily testing over seven days to allow continued school attendance (intervention group) or to follow the usual policy of isolation of contacts for 10 days (control group). In two studies there were insufficient details to permit a judgement of the risk of bias (Alfelali 2020; Ashraf 2020). 

In the 2020 review, we judged 11 cluster‐RCTs as at high risk of identification/recruitment bias (Aiello 2010; Aiello 2012; Azor‐Martinez 2018; Chard 2019; Correa 2012; Cowling 2009; Larson 2010; McConeghy 2017; Nicholson 2014; Priest 2014; Savolainen‐Kopra 2012). In Aiello 2010 and Aiello 2012, recruitment continued for two weeks after the start of the study, which could have introduced bias. Six trials identified and recruited participants after cluster randomisation (Azor‐Martinez 2018; Chard 2019; Cowling 2009; Larson 2010; McConeghy 2017; Nicholson 2014). Three trials recruited new participants after the start of the study to replace those lost to follow‐up (Correa 2012; Priest 2014; Savolainen‐Kopra 2012). We judged five cluster‐RCTs to have probable identification/recruitment bias (Alzaher 2018; Barasheed 2014; MacIntyre 2011; Najnin 2019; Radonovich 2019), whereas in 19 studies there were insufficient details to permit a judgement of risk of bias (Carabin 1999; DiVita 2011; Feldman 2016; Hartinger 2016; Huda 2012; Ibfelt 2015; Kotch 1994; Ladegaard 1999; MacIntyre 2009; MacIntyre 2013; Millar 2016; Miyaki 2011; Pandejpong 2012; Radonovich 2019; Sandora 2008; Stebbins 2011; Talaat 2011; Yeung 2011; Zomer 2015).

Two of the newly included cluster‐RCTs reported intracluster correlation coefficient (ICC) to adjust sample size, taking into consideration clustering effects, and described adjusting outcomes for clustering effect using different statistical methods, or provided justification for not performing adjusted analysis for clustering (Alfelali 2020; Swarthout 2020). For four studies there were insufficient details to permit a judgement of risk of bias (Abaluck 2022; Ashraf 2020; Teesing 2021; Young 2021) since they provided insufficient details on ICC and/or did not perform adjusted analysis or justified the absence of it.

Twenty‐six cluster‐RCTs identified in the 2020 review reported intracluster correlation coefficient (ICC) to adjust sample size, taking into consideration clustering effects, and described adjusting outcomes for clustering effect using different statistical methods, or provided justification for not performing adjusted analysis for clustering (Aiello 2010; Aiello 2012; Arbogast 2016; Canini 2010; Carabin 1999; Correa 2012; Cowling 2008; Cowling 2009; Hartinger 2016; Huda 2012; Little 2015; Luby 2005; MacIntyre 2009; MacIntyre 2011; MacIntyre 2013; MacIntyre 2015; MacIntyre 2016; McConeghy 2017; Priest 2014; Radonovich 2019; Ram 2015; Roberts 2000; Stebbins 2011; Suess 2012; Talaat 2011; Temime 2018). Five cluster‐RCTs did not report the ICC but described adjusting outcomes for clustering effect using different statistical methods, or explained why adjusted analysis for clustering was not performed (Biswas 2019; Chard 2019; McConeghy 2017; Simmerman 2011; Zomer 2015). Thirteen cluster‐RCTs provided insufficient details on ICC and/or did not perform adjusted analysis or justified the absence of it (Alzaher 2018; Azor‐Martinez 2016; Azor‐Martinez 2018; Barasheed 2014; Feldman 2016; Larson 2010; Millar 2016; Miyaki 2011; Najnin 2019; Nicholson 2014; Pandejpong 2012; Savolainen‐Kopra 2012; Yeung 2011). Two cluster‐RCTs reported the ICC but did not perform adjusted analysis or justified the absence of it (Sandora 2005; Sandora 2008).

### Effects of interventions

See: Table 1; Table 2; Table 3

#### Comparison 1: Medical/surgical masks compared to no masks

We included 12 trials (10 of which were cluster‐RCTs) comparing medical/surgical masks versus no masks (Abaluck 2022; Alfelali 2020; Aiello 2012; Barasheed 2014; Bundgaard 2021; Canini 2010; Cowling 2008; Jacobs 2009; MacIntyre 2009; MacIntyre 2015; MacIntyre 2016; Suess 2012). Two trials were conducted with healthcare workers (HCWs) (Jacobs 2009; MacIntyre 2015), whilst the other 10 studies included people living in the community. In the acute care hospital setting, as opposed to the community setting, variable mask use occurred, according to usual practices in the settings where the studies were undertaken, varying from just under 16% most of the time to 23.6% wearing for > 70% of all working hours (Jacobs 2009; MacIntyre 2015). We therefore excluded the two studies in the acute care hospital setting from the meta‐analysis, and report results from these studies narratively. Ten trials were conducted in non‐pandemic settings, and two were conducted during the SARS‐CoV‐2 pandemic (Abaluck 2022; Bundgaard 2021).

##### Primary outcomes

###### 1. Numbers of cases of viral respiratory illness

####### Influenza/COVID‐like illness

Pooling of nine trials conducted in the community found an estimate of effect for the outcomes of influenza/COVID‐like illness cases (risk ratio (RR) 0.95, 95% confidence interval (CI) 0.84 to 1.09; 9 trials; 276,917 participants; moderate‐certainty evidence; Analysis 1.1) suggesting that wearing a medical/surgical mask will probably make little or no difference for this outcome. Two studies in healthcare workers provided inconclusive results with very wide confidence intervals: RR 0.88, 95% CI 0.02 to 32; and RR 0.26, 95% CI 0.03 to 2.51, respectively (Jacobs 2009; MacIntyre 2015). 

####### Laboratory‐confirmed influenza/SARS‐CoV‐2 cases

Similarly, the estimate of effect for laboratory‐confirmed influenza/SARS‐CoV‐2 cases (RR 1.01, 95% CI 0.72 to 1.42; 6 trials, 13,919 participants; moderate‐certainty evidence; Analysis 1.1) suggests that wearing a medical/surgical mask probably makes little or no difference compared to not wearing a mask for this outcome. 

####### Laboratory‐confirmed other respiratory viruses

One community study reported on laboratory‐confirmed other respiratory viruses, showing RR 0.58, 95% CI 0.25 to 1.31; Analysis 1.1, and another study in healthcare workers reported RR 0.79, 95% CI 0.42 to 1.52 (MacIntyre 2015). 

####### Assessing both source control and personal protection

The design of most trials assessed whether masks protected the wearer. Six trials were cluster‐RCTs, with all participants in the intervention clusters required to wear masks, thus assessing both source control and personal protection. In two trials the clusters were households with a member with new influenza; neither of these studies found any protective effect (RR 1.03 in 105 households (Canini 2010); RR 1.21 in 145 households (MacIntyre 2009)). In two trials the clusters were college dormitories during the influenza season; neither study found any reduction (RR 1.10 in 37 dormitories (Aiello 2012); RR 0.90 in three dormitories (Aiello 2010)). 

####### Studies conducted during the SARS‐CoV‐2 pandemic

Two studies were conducted during the SARS‐CoV‐2 pandemic (Abaluck 2022; Bundgaard 2021), with the former being a very large cluster‐RCT of villages in Bangledesh and the latter a large RCT conducted in Denmark.  

####### Exclusion of study due to insufficient number of clusters

We excluded Aiello 2010 from the meta‐analysis since we did not consider 'randomisation' of three clusters to three arms to be a proper randomised trial.

###### 2. Adverse events related to the intervention

Canini 2010 reported that 38 (75%) of participants in the intervention arm experienced discomfort with the mask use due to warmth (45%), respiratory difficulties (33%), and humidity (33%). Children reported feeling pain more frequently (3/12) than other participants wearing adult face masks (1/39; P = 0.04). In MacIntyre 2015, adverse events associated with face mask use were reported in 40.4% (227/562) of HCWs in the medical‐mask arm. General discomfort (35.1%; 397/1130) and breathing problems (18.3%; 207/1130) were the most frequently reported adverse events. Suess 2012 reported that the majority of participants (107/172; 62%) did not report any problems with mask‐wearing. More adults reported no problems (71%) compared to children (36/72; 50%; P = 0.005). The main issues when wearing a face mask for adults as well as for children were "heat/humidity" (18/34; 53% of children; 10/29; 35% of adults; P = 0.1), followed by "pain" and "shortness of breath". Alfelali 2020 reported the most common side effects of wearing a mask in Hajj pilgrims were difficulty in breathing (26%) and discomfort (22%). Although no details were provided, Bundgaard 2021 mentioned that 14% of participants had adverse reactions. Cowling 2008 and Abaluck 2022 mentioned that no adverse events were reported. The other trials did not report measuring adverse outcomes.

##### Secondary outcomes

###### 1. Deaths

Not reported.

###### 2. Severity of viral respiratory illness as reported in the studies

Jacobs 2009 reported that participants in the mask group were significantly more likely to experience more days with headache and feeling bad. They found no significant differences between the two groups for symptom severity scores. None of the other trials reported this outcome.

###### 3. Absenteeism

Not reported.

###### 4. Hospital admissions

Not reported.

###### 5. Complications related to the illness (e.g. pneumonia)

Not reported.

#### Comparison 2: N95/P2 respirators compared to medical/surgical masks

We included five trials comparing medical/surgical masks with N95/P2 respirators (Loeb 2009; MacIntyre 2009; MacIntyre 2011; MacIntyre 2013; Radonovich 2019). All of these trials except MacIntyre 2009 included HCWs. MacIntyre 2009 included carers and household members of children with a respiratory illness recruited from a paediatric outpatient department and a paediatric primary care practice in Sydney, Australia. None of the trials were conducted during the SARS‐CoV‐2 pandemic.

##### Primary outcomes

###### 1. Numbers of cases of viral respiratory illness

####### Clinical respiratory illness

Pooling of three trials found an estimate of effect suggesting considerable uncertainty as to whether an N95/P2 respirator provides any benefit compared to medical/surgical masks for the outcome of clinical respiratory illness (RR 0.70, 95% CI 0.45 to 1.10; 7799 participants, very low‐certainty evidence; Analysis 2.1) (MacIntyre 2011; MacIntyre 2013 (two arms); Radonovich 2019). 

####### Influenza‐like‐illness

Based on five trials conducted in four healthcare settings and one household, the estimates of effect for the outcome of ILI (RR 0.82, 95% CI 0.66 to 1.03; 8407 participants, low‐certainty evidence; Analysis 2.1) suggest that N95/P2 respirators may make little or no difference for this outcome (Loeb 2009; MacIntyre 2009; MacIntyre 2011; MacIntyre 2013; Radonovich 2019). 

####### Laboratory‐confirmed influenza

The estimate of the effect for the outcome of laboratory‐confirmed influenza infection (RR 1.10, 95% CI 0.90 to 1.34; 8407 participants, moderate‐certainty evidence; Analysis 2.1) suggests that the use of a N95/P2 respirator compared to a medical/surgical mask probably makes little or no difference for this more precise and objective outcome. 

The outcomes clinical respiratory illness and ILI were reported separately. Considering how these outcomes were defined, it is highly likely that there was considerable overlap between the two, therefore these outcomes were not combined into a single clinical outcome (Analysis 2.1). The laboratory‐confirmed viral respiratory infection outcome included influenza primarily but multiple other common viral respiratory pathogens were also included in several studies. The laboratory‐confirmed viral infection outcome was considered more precise and objective in comparison to the clinical outcomes, which were more subjective and considered to be less precise. The findings did not change when we restricted the evidence to HCWs (Analysis 2.2).

###### 2. Adverse events related to the intervention

Harms were poorly reported, but generally discomfort wearing medical/surgical masks and N95/P32 respirators was mentioned in several studies. Radonovich 2019 mentioned that participants wearing the N95 respirator reported skin irritation and worsening of acne. MacIntyre 2011 reported that adverse events were more common with N95 respirators; in particular, discomfort was reported in 41.9% of N95 wearers versus 9.8% of medical‐mask wearers (P < 0.01); headaches were more common with N95 (13.4% versus 3.9%; P < 0.01); difficulty breathing was reported more often in the N95 group (19.4% versus 12.5%; P = 0.01); and N95 caused more problems with pressure on the nose (52.2% versus 11.0%; P < 0.01). In MacIntyre 2013, fewer participants using the N95 respirator reported problems (38% (195/512) versus 48% (274/571) of participants in the medical‐mask arm; P = 0.001). Loeb 2009 mentioned that no adverse events were reported.

The one trial conducted in the community mentioned that more than 50% of participants reported concerns with both types of masks, mainly that wearing them was uncomfortable, but there were no significant differences between the P2 (N95) and surgical‐mask groups (MacIntyre 2009).

##### Secondary outcomes

###### 1. Deaths

Not reported.

###### 2. Severity of viral respiratory illness as reported in the studies

Not reported.

###### 3. Absenteeism

Loeb 2009 reported that 42 participants (19.8%) in the surgical‐mask group reported an episode of work‐related absenteeism compared with 39 (18.6%) of participants in the N95 respiratory group (absolute risk difference −1.24%, 95% CI −8.75% to 6.27%; P = 0.75).

###### 4. Hospital admissions

Not reported.

###### 5. Complications related to the illness (e.g. pneumonia)

Loeb 2009 reported that there were no episodes of LRTIs.

#### Comparison 3: Hand hygiene compared to control

Nineteen trials compared hand hygiene interventions with control and provided sufficient data to include in meta‐analyses (Ashraf 2020; Azor‐Martinez 2018; Biswas 2019; Correa 2012; Cowling 2008; Cowling 2009; Hubner 2010; Larson 2010; Little 2015; Millar 2016; Nicholson 2014; Ram 2015; Roberts 2000; Sandora 2005; Simmerman 2011; Stebbins 2011; Swarthout 2020; Teesing 2021; Zomer 2015). The populations of these studies included adults, children, and families, in settings such as schools, childcare centres, homes, and offices. None of the studies was conducted during a pandemic, although a few studies were conducted during peak influenza seasons. A further 16 trials comparing hand hygiene to a control had other outcomes or insufficient information to include in meta‐analyses (Alzaher 2018; Arbogast 2016; Azor‐Martinez 2016; DiVita 2011; Feldman 2016; Gwaltney 1980; Ladegaard 1999; Luby 2005; Morton 2004; Priest 2014; Savolainen‐Kopra 2012; Talaat 2011; Temime 2018; Turner 2012; White 2001; Yeung 2011). The results of these trials were consistent with the findings of our meta‐analyses. The results for all outcomes from the 19 trials that were meta‐analysed and the 16 trials that were not meta‐analysed are shown in Table 5.

**2:** Results from trials of hand hygiene compared to control

**Study**	**Comparison** (see Table 4 for details of interventions)	**Reported outcomes**	**Results**
Alzaher 2018 cluster‐RCT Saudi Arabia	Hand‐washing workshop and posters versus usual practice	% absence days due to URI	0.39% and 0.72% in intervention group schools; 0.86% and 1.39% in control schools
Arbogast 2016 cluster‐RCT USA	Hand sanitiser + wipes + hand foam versus none Both groups received education + signage about hand‐washing	1. Health insurance claims for preventable illnesses per employee 2. Absences per employee	1. 0.30 claims in intervention; 0.37 in control (27% relative reduction; P = 0.03) 2. 1.45 in intervention; 1.53 in control (5.0% relative reduction in intervention; P = 0.30)
Ashraf 2020 cluster‐RCT Bangladesh	6 intervention arms: water quality, sanitation, hand washing, combined WSH, nutrition, nutrition + WSH	7‐day prevalence of acute respiratory illness (ARI).	Hand washing reduced ARI cases by 32% (RR 0.68, 95% CI 0.52 to 0.88)
Azor‐Martinez 2016 RCT Spain	Hand‐washing with soap and water plus hand sanitiser versus usual hand‐washing practices	% absence days due to URI	1.15% in intervention; 1.68% in control. Significantly lower in intervention (P < 0.001)
Azor‐Martinez 2018 cluster‐RCT Spain	Education and hand hygiene with soap and water versus hand hygiene with sanitiser versus usual hand‐washing procedures	1. URI incidence rate ratio (primary) 2. Percentage difference in absenteeism days	1. HH soap versus control 0.94 (95% CI 0.82 to 1.08); HH sanitiser versus control 0.77 (95% CI 0.68 to 0.88); HH soap versus HH sanitiser 1.21 (95% CI 1.06 to 1.39) 2. HH soap 3.9% versus control 4.2% (P < 0.001); HH sanitiser 3.25% versus control 4.2% (P = 0.026); HH soap 3.9% versus HH sanitiser 3.25% (P < 0.001)
Biswas 2019 cluster‐RCT Bangladesh	Hand sanitiser and respiratory hygiene education and cough/sneeze hygiene versus no intervention	1. ILI incidence rate (at least 1 episode) 2. Laboratory‐confirmed influenza	1. 22 per 1000 student‐weeks in intervention; 27 per 1000 student‐weeks in control, not statistically significantly different 2. 3 per 1000 student‐weeks in intervention; 6 per 1000 student‐weeks in control, P = 0.01
Correa 2012 cluster‐RCT Colombia	Alcohol‐based hand sanitiser in addition to hand‐washing versus usual hand‐washing practice	ARIs in 3rd trimester of follow‐up	Hazard ratio for intervention to control 0.69 (95% CI 0.57 to 0.83)
Cowling 2008 cluster‐RCT Hong Kong	Hand hygiene (36 households) versus face mask (mask) versus education (control)	Secondary attack rate for: 1. laboratory‐confirmed influenza; 2. ILI definition 1; 3. ILI definition 2; 4. ILI definition 3.	1. HH 0.06; mask 0.07; control 0.06 2. HH 0.18; mask 0.18; control 0.18 3. HH 0.11; mask 0.10; control 0.11 4. HH 0.04; mask 0.08; control 0.04
Cowling 2009 cluster‐RCT Hong Kong	Hand hygiene (HH) versus face mask + hand hygiene (HH + mask) versus education (control)	Secondary attack rate for: 1. laboratory‐confirmed influenza; 2. ILI definition 1; 3. ILI definition 2.	1. HH 5; HH + mask 7; control 10 2. HH 16; HH + mask 21; control 19 3. HH 4; HH + mask 7; control 5
DiVita 2011 (conference abstract) RCT Bangladesh	Hand‐washing stations with soap and motivation vs none	1. SAR for laboratory‐confirmed influenza 2. SAR for ILI	1. SAR higher in intervention group (11.0% versus 7.5%) 2. SAR higher in intervention group (14.2% versus 11.9%)
Feldman 2016 cluster‐RCT Israel	Hand disinfection + soap and water installed versus none	1. Number of respiratory infections 2. Number of off‐duty days	1. 11 in each group 2. 112 in intervention; 104 in control
Gwaltney 1980 RCT USA	Virucidal hand wash versus placebo	1. Number with illness after immediate exposure 2. Number with illness after 2‐hour delay in exposure	1. 0 of 8 in intervention; 7 of 7 in control 2. 1 of 10 in intervention; 6 of 10 in control
Hubner 2010 RCT Germany	Hand disinfection provided versus none	Odds ratios (95% CI) (intervention:control) 1. Influenza 2. Common cold 3. Sinusitis 4. Sore throat 5. Fever 6. Cough	1. 1.02 (0.20 to 5.23) 2. 0.35 (0.17 to 0.71) 3. 1.87 (0.52 to 6.74) 4. 0.62 (0.31 to 1.25) 5. 0.38 (0.14 to 0.99) 6. 0.45 (0.22 to 0.91)
Ladegaard 1999 RCT Denmark	Hand hygiene and education versus none	Sick days during the "effect period"	22 days/child in the intervention group versus 36 days/child in the control group
Larson 2010 cluster‐RCT USA	Education versus education with alcohol‐based hand sanitiser versus education with hand sanitiser and face masks	Incidence rate ratios (episodes per 1000 person‐weeks) for: 1. URI; 2. ILI; 3. influenza. Secondary attack rates for: 4. URI/ILI/influenza; 5. ILI/influenza.	1. HS 29; HS + masks 39; control 35 2. HS 1.9; HS + masks 1.6; control 2.3 3. HS 0.6; HS + masks 0.5; control 2.3 4. HS 0.14; HS + masks 0.12; control 0.14 5. HS 0.02; HS + masks 0.02; control 0.02
Little 2015 RCT England	Bespoke automated web‐based hand hygiene motivational intervention with tailored feedback versus none	Number of participants with 1 or more episodes of URI	Risk ratio for intervention to control 0.86 (95% CI 0.83 to 0.89; P < 0.001)
Luby 2005 RCT Pakistan	Antibacterial soap and education about hand‐washing versus plain soap and education versus none	1. Cough or difficulty breathing in children < 15 yrs (episodes/100 person‐weeks) 2. Congestion or coryza in children < 15 yrs (episodes/100 person‐weeks) 3. Pneumonia in children < 5 yrs (episodes/100 person‐weeks)	All outcomes significantly lower than control 1. 4.21 in antibacterial soap group; 4.16 in plain soap group; 8.50 in control group 2. 7.32 in antibacterial soap group; 6.87 in plain soap group; 14.78 in control group 3. 2.42 in antibacterial soap group; 2.20 in plain soap group; 4.40 in control group
Millar 2016 cluster‐RCT USA	Standard educational promotion of hand‐washing versus enhanced promotion versus promotion plus a once‐weekly application of chlorhexidine‐based body wash	Incidence rates of ARI over 20 months	37.7 enhanced + body wash; 29.3 enhanced; 35.3 standard; RR for enhanced + body wash to standard 1.07 (95% CI 1.03 to 1.11); RR for enhanced to enhanced + body wash 0.78 (95% CI 0.75 to 0.81)
Morton 2004 cluster‐RCT cross‐over study USA	Alcohol gel plus education versus regular hand‐washing	Absence due to infectious illness	Results not stated numerically
Nicholson 2014 cluster‐RCT India	Combination hand‐washing promotion with provision of free soap versus none	Target children: 1. Episodes of ARI (per 100 person‐weeks) 2. School absence episodes (per 100 person‐days) Families: 3. Episodes of ARI	1. 16 in intervention; 19 in control 2. 1.2 in intervention; 1.7 in control 3. 10 in intervention; 11 in control
Priest 2014 cluster‐RCT New Zealand	Hand hygiene education and hand sanitiser versus education alone	1. % absence days due to respiratory illness 2. % absence days due to any illness	1. 0.84% in intervention group; 0.80% in control (P = 0.44) 2. 1.21% in intervention group; 1.16% in control (P = 0.35)
Ram 2015 RCT Bangladesh	Education to promote intensive hand‐washing in households plus soap provision versus none	1. Secondary attack ratio for intervention to control for ILI 2. Laboratory‐confirmed influenza	1. 1.24 (95% CI 0.93 to 1.65) 2. 2.40 (95% CI 0.68 to 8.47)
Roberts 2000 cluster‐RCT Australia	Hand‐washing programme with training for staff and children versus none	Incidence rate ratio for ARI	IRR 0.92 for intervention to control (95% CI 0.86 to 0.99)
Sandora 2008 cluster‐RCT USA	Hand sanitiser and education versus none	Incidence rates for ARI (episodes per person‐month)	0.43 in intervention; 0.42 in control
Savolainen‐Kopra 2012 cluster‐RCT Finland	Hand hygiene with soap and water (IR1 group) versus with alcohol‐based hand rub (IR2 group) versus control (none); intervention groups also received education	1. Number of respiratory infection episodes/week 2. Number of reported infection episodes/week 3. Number of reported sick leave episodes/week	1. 0.076 in IR1; 0.085 in IR2; 0.080 in control, NS 2. 0.097 in IR1; 0.107 in IR2; 0.104 in control, NS 3. 0.042 in IR1; 0.035 in IR2; 0.035 in control. Significantly higher in IR1 compared with control
Simmerman 2011 cluster‐RCT Thailand	Hand‐washing (HW) versus handwashing plus paper surgical face masks (HW + FM) versus control (none)	Odds ratios for secondary attack rates for influenza	OR for HW: control 1.20 (95% CI 0.76 to 1.88) OR for HW + masks: control 1.16 (95% CI 0.74 to 1.82) OR for HW + masks: HW 0.72 (95% CI 0.21 to 2.48)
Stebbins 2011 cluster‐RCT USA	Training in hand and respiratory (cough) hygiene + hand sanitiser versus none	Incidence rate ratios for intervention to control for: 1. laboratory‐confirmed influenza (RT‐PCR); 2. influenza‐A; 3. absence.	1. IRR 0.81 (95% CI 0.54 to 1.23) 2. IRR 0.48 (95% CI 0.26 to 0.87) 3. IRR 0.74 (95% CI 0.56 to 0.97)
Swarthout 2020 cluster‐RCT Kenya	There were 6 intervention groups: chlorinated drinking water (W), improved sanitation (S), handwashing with soap (H), combined WSH, improved nutrition (N) through counselling lipid based nutrient supplementation (LNS) combined WSHN There were 2 control groups passive control (no promotional visits), a double‐sized active control (monthly visits to measure mid–upper arm circumference)	Prevalence of ARIs in children	No evidence of an effect: RR 0.97, 95% CI 0.90 to 1.04.
Talaat 2011 cluster‐RCT Egypt	Mandatory hand‐washing intervention + education versus none	1. Number of absence days due to ILI 2. Number of absence days	1. 917 in intervention; 1671 in control (P < 0.001) 2. 13,247 in intervention; 19,094 in control (P < 0.001)
Teesing 2021 cluster‐RCT Netherlands	Hand hygiene enhancement activities versus no activities.	Incidence of gastroenteritis, influenza‐like illness (ILI), assumed pneumonia, urinary tract infections (UTIs), and infections caused MRSA in residents	Hand hygiene reduced risk of ILI (RR 0.51, 95% CI 0.31 to 0.83)
Temime 2018 cluster‐RCT France	Hand hygiene with alcohol‐based hand rub, promotion, staff education, and local work groups versus none	Incidence rate of ARI clusters (5 or more people in same nursing home)	2 ARI clusters in intervention; 1 in control
Turner 2012 RCT USA	Antiviral hand treatment versus no treatment	1. Number of rhinovirus infections 2. Common cold infections 3. Rhinovirus‐associated illnesses	1. 49 in intervention; 49 in control, NS 2. 56 in intervention; 72 in control, NS 3. 26 in intervention; 24 in control, NS
White 2001 DB‐RCT USA	Hand rub with benzalkonium chloride (hand sanitiser) versus placebo	ARI symptoms Laboratory: testing of virucidal and bactericidal activity of the product	30% to 38% decrease of illness and absenteeism (RR for illness absence incidence 0.69; RR for absence duration 0.71)
Yeung 2011 cluster‐RCT Hong Kong	Alcohol‐based hand gel + materials + education versus control (basic life support workshop)	Difference between pre‐study period and post study in pneumonia infections recorded in residents	0.63/1000 reduction in intervention group; 0.16/1000 increase in control
Zomer 2015 cluster‐RCT Netherlands	4 components: 1. Hand hygiene products, paper towel dispensers, soap, alcohol‐based hand sanitiser, and hand cream provided for 6 months 2. Training and booklet 3. 2 team training sessions aimed at hand hygiene improvement 4. Posters and stickers for caregivers and children as reminders. Combination versus usual practice	Incidence rate ratio for intervention to control for common cold	IRR 1.07 (95% CI 0.97 to 1.19) 8.2 episodes per child‐year in intervention; 7.4 episodes per child‐year in control

ARI: acute respiratory infection CI: confidence interval cluster‐RCT: cluster‐randomised controlled trial DB‐RCT: double‐blind randomised controlled trial HH: hand hygiene HS: hand sanitiser HW: hand‐washing ILI: influenza‐like illness IRR: incidence rate ratio NS: non‐significant OR: odds ratio RCT: randomised controlled trial RR: risk ratio RT‐PCR: reverse‐transcriptase polymerase chain reaction SAR: secondary attack rate URI: upper respiratory infection yrs: years

##### Primary outcomes

###### 1. Numbers of cases of viral respiratory illness

####### Acute respiratory infection (ARI)

Pooling of nine trials for the broad outcome of ARI showed a 14% relative reduction in the numbers of participants with ARI (RR 0.86, 95% CI 0.81 to 0.90; 52,105 participants, moderate‐certainty evidence; Analysis 3.1.1) in the hand hygiene group (Analysis 3.1), suggesting a probable benefit (Ashraf 2020; Azor‐Martinez 2018; Correa 2012; Larson 2010; Little 2015; Millar 2016; Nicholson 2014; Sandora 2005; Swarthout 2020). 

####### Influenza‐like‐illness (ILI) and laboratory‐confirmed influenza

When considering the more strictly defined outcomes of ILI (Biswas 2019; Cowling 2008; Cowling 2009; Hubner 2010; Larson 2010; Little 2015; Ram 2015; Roberts 2000; Simmerman 2011; Teesing 2021; Zomer 2015), and laboratory‐confirmed influenza (Biswas 2019; Cowling 2008; Cowling 2009; Hubner 2010; Larson 2010; Ram 2015; Simmerman 2011; Stebbins 2011) the estimates of the effect were heterogeneous, suggesting that hand hygiene may make little or no difference (RR 0.94, 95% CI 0.81 to 1.09 for ILI; 34,503 participants, low‐certainty evidence; Analysis 3.1.2); (RR 0.91, 95% CI 0.63 to 1.30 for laboratory‐confirmed influenza; 8332 participants; low‐certainty evidence; Analysis 3.1.3).

####### Composite outcome ‘ARI or ILI or influenza'

All 19 trials could be pooled for analysis of the composite outcome ‘ARI or ILI or influenza’, with each study only contributing once with the most comprehensive outcome (in terms of number of events) reported showing an 11% relative reduction in participants with a respiratory illness, suggesting that hand hygiene may offer a benefit (RR 0.89, 95% CI 0.83 to 0.94; low‐certainty evidence; Analysis 3.2), but with high heterogeneity. A funnel plot of the 19 trial results did not appear to suggest any small study effects for this outcome (Figure 4). 

**4:**
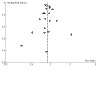


####### Sensitivity analysis

In a sensitivity analysis we used only the most precise and unequivocal (with laboratory confirmed considered the most precise and an undefined ARI considered the least precise) outcome reported in each of 12 studies identified by JMC, an infectious disease physician, and found an estimate of effect in favour of hand hygiene, but with wider CIs (RR 0.88, 95% CI 0.77 to 1.02; Analysis 3.3). 

####### Subgroup analysis by age group

We considered that studies in children might have a different effect than studies in adults, so we conducted subgroup analysis by age group. We found no evidence of a difference in treatment effect by age group (P = 0.18; Analysis 3.4).

###### 2. Adverse events related to the intervention

Correa 2012 reported that no adverse events were observed; in the study by Priest 2014, skin reaction was recorded for 10.4% of participants in the hand sanitiser group versus 10.3% in the control group (RR 1.01, 95% CI 0.78 to 1.30).

##### Secondary outcomes

###### 1. Deaths

Not reported.

###### 2. Severity of viral respiratory illness as reported in the studies

Not reported.

###### 3. Absenteeism

Three trials measured absenteeism from school or work and demonstrated a 36% relative reduction in the numbers of participants with absence in the hand hygiene group (RR 0.64, 95% CI 0.58 to 0.71; Analysis 3.5) (Azor‐Martinez 2016; Hubner 2010; Nicholson 2014).

###### 4. Hospital admissions

Not reported.

###### 5. Complications related to the illness (e.g. pneumonia)

Not reported.

#### Comparison 4: Hand hygiene + medical/surgical masks compared to control

##### Primary outcomes

###### 1. Numbers of cases of viral respiratory illness (including ARIs, ILI, and laboratory‐confirmed influenza)

Six trials (Aelami 2015; Aiello 2012; Cowling 2009; Larson 2010; Simmerman 2011; Suess 2012) were able to be pooled to compare the use of the combination of hand hygiene and medical/surgical masks with control. Four of these trials were in households, two in university student residences, and one at the annual Hajj pilgrimage. For the outcomes ILI and laboratory‐confirmed influenza, pooling demonstrated an estimate of effect suggesting little or no difference between the hand hygiene and medical/surgical mask combination and control. The number of trials and events was lower than for comparisons of hand hygiene alone, or medical/surgical masks alone, and the confidence interval was wide. For ILI, the RR for intervention compared to control was 1.03 (95% CI 0.77 to 1.37; 4504 participants; Analysis 4.1.1), and for influenza it was 0.97 (95% CI 0.69 to 1.36; 3121 participants; Analysis 4.1.2). Full results of these trials are shown in Table 6

**3:** Results from trials of hand hygiene + medical/surgical masks compared to control

**Study**	**Comparison** (see Table 4 for details of interventions)	**Reported outcomes**	**Results**
Aelami 2015 (conference abstract) RCT Saudi Arabia	Hand hygiene education + alcohol‐based hand rub + soap + surgical masks vs none	Proportion with ILI (defined as presence of ≥ 2 of the following during their stay: fever, cough, and sore throat)	52% in intervention; 55.3% in control (P < 0.001)
Aiello 2010 cluster‐RCT USA	Face mask use (FM) vs face masks + hand hygiene (FM + HH) vs control Note that this study is not included in meta‐analysis as each treatment group included only 1 cluster.	1. ILI 2. Laboratory‐confirmed influenza A or B	Significant reduction in ILI cases in both intervention groups compared with control over weeks 3 to 6 No significant differences between FM and FM + HH
Aiello 2012 cluster‐RCT USA	Face mask use (FM) vs face masks + hand hygiene (FM + HH) vs control	1. Clinical ILI 2. Laboratory‐confirmed influenza A or B	1. Non‐significant reductions in FM group compared with control over all weeks. Significant reduction in FM + HH group compared with control in weeks 3 to 6 2. Non‐significant reductions in both intervention groups compared with control
Cowling 2009 cluster‐RCT Hong Kong	Hand hygiene (HH) vs hand hygiene plus face masks (HH + mask) vs control	Secondary attack ratio for: 1. laboratory‐confirmed influenza; 2. ILI definition 1; 3. ILI definition 2.	1. HH 5; HH + mask 7; control 10 2. HH 16; HH + mask 21; control 19 3. HH 4; HH + mask 7; control 5
Larson 2010 cluster‐RCT USA	Education (control) vs education with alcohol‐based hand sanitiser (HS) vs education + HS + face masks (HS + mask)	Incidence rate ratios (episodes per 1000 person‐weeks) for: 1. URI; 2. ILI; 3. influenza. Secondary attack rates for: 4. URI/ILI/influenza; 5. ILI/influenza.	1. HS 29; HS + mask 39; control 35 2. HS 1.9; HS + mask 1.6; control 2.3 3. HS 0.6; HS + mask 0.5; control 2.3 4. HS 0.14; HS + mask 0.12; control 0.14 5. HS 0.02; HS + mask 0.02; control 0.02
Simmerman 2011 cluster‐RCT Thailand	Control vs hand‐washing (HW) vs hand‐washing + paper surgical face masks (HW + mask)	Odds ratio for secondary attack rates for influenza	OR for HW: control 1.20 (95% CI 0.76 to 1.88) OR for HW + mask: control 1.16 (95% CI 0.74 to 1.82) OR for HW + mask: HW 0.72 (95% CI 0.21 to 2.48)
Suess 2012 cluster‐RCT Germany	Face mask + hand hygiene (mask + HH) vs face masks only (mask) vs none (control)	Secondary attack rates in household contacts: 1. Laboratory‐confirmed influenza 2. ILI	1. Mask 9; mask + HH 15; control 23 2. Mask 9; mask + HH 9; control 17

CI: confidence interval cluster‐RCT: cluster‐randomised controlled trial FM: face mask HH: hand hygiene HS: hand sanitiser HW: hand‐washing ILI: influenza‐like illness OR: odds ratio RCT: randomised controlled trial URI: upper respiratory infection vs: versus

###### 2. Adverse events related to the intervention

Adverse events related to mask wearing in the study by Suess 2012 are reported under Comparison 1 (medical/surgical masks). There was no mention of adverse events related to hand hygiene.

##### Secondary outcomes

###### 1. Deaths

Not reported.

###### 2. Severity of viral respiratory illness as reported in the studies

Not reported.

###### 3. Absenteeism

Not reported.

###### 4. Hospital admissions

Not reported.

###### 5. Complications related to the illness, e.g. pneumonia

Not reported.

#### Comparison 5: Hand hygiene + medical/surgical masks compared to hand hygiene

##### Primary outcomes

###### 1. Numbers of cases of viral respiratory illness (including ARIs, ILI and laboratory‐confirmed influenza)

Three trials studied the addition of medical/surgical masks to hand hygiene (Cowling 2009; Larson 2010; Simmerman 2011). All three trials had three arms, and are also included in the comparison of hand hygiene plus medical/surgical mask versus control (Comparison 4). All three studies showed no difference between hand hygiene plus medical/surgical mask groups and hand hygiene alone, for all outcomes. The estimates of effect suggested little or no difference when adding masks to hand hygiene compared to hand hygiene alone: for the outcome ILI (RR 1.03, 95% CI 0.69 to 1.53; 3 trials) and the outcome laboratory‐confirmed influenza (RR 0.99, 95% CI 0.69 to 1.44), the estimates of effect were not different and the CIs were relatively wide, suggesting little or no difference (Analysis 5.1). However, the CIs around the estimates were wide and do not rule out an important benefit.

###### 2. Adverse events related to the intervention

Not reported.

##### Secondary outcomes

###### 1. Deaths

Not reported.

###### 2. Severity of viral respiratory illness as reported in the studies

Not reported.

###### 3. Absenteeism

Not reported.

###### 4. Hospital admissions

Not reported.

###### 5. Complications related to the illness (e.g. pneumonia)

Not reported.

#### Comparison 6: Medical/surgical masks compared to other (non‐N95) masks

One trial compared medical/surgical masks with cloth masks in hospital healthcare workers (MacIntyre 2015), and another trial compared catechin‐treated masks versus control masks in healthcare workers and staff of hospitals, rehabilitation centres, and nursing homes in Japan (Ide 2016).

##### Primary outcomes

###### 1. Numbers of cases of viral respiratory illness (including ARIs, ILI, and laboratory‐confirmed influenza)

MacIntyre 2015 found that the rate of ILI was higher in the cloth mask arm compared to the medical/surgical masks arm (RR 13.25, 95% CI 1.74 to 100.97).

Ide 2016 did not find a benefit from the catechin‐treated masks over untreated masks on influenza infection rates (adjusted odds ratio (OR) 2.35, 95% CI 0.40 to 13.72; P = 0.34).

###### 2. Adverse events related to the intervention

In MacIntyre 2015 adverse events associated with face mask use were reported in 40.4% (227/562) of HCWs in the medical/surgical mask arm and 42.6% (242/568) in the cloth mask arm (P = 0.45). The most frequently reported adverse events were general discomfort (35.1%; 397/1130) and breathing problems (18.3%; 207/1130). Laboratory tests showed the penetration of particles through the cloth masks to be very high (97%) compared with medical/surgical masks (44%). Ide 2016 reported that there were no serious adverse events associated with the intervention.

##### Secondary outcomes

###### 1. Deaths

Not reported.

###### 2. Severity of viral respiratory illness as reported in the studies

Not reported.

###### 3. Absenteeism

Not reported.

###### 4. Hospital admissions

Not reported.

###### 5. Complications related to the illness (e.g. pneumonia)

Not reported.

#### Comparison 7: Soap + water compared to sanitiser, and comparisons of different types of sanitiser

Two trials compared soap and water with sanitiser (Azor‐Martinez 2018; Savolainen‐Kopra 2012). Another trial compared different types of hand sanitiser in a virus challenge study (Turner 2004a; Turner 2004b), and one trial studied the frequency of use of hand sanitiser (Pandejpong 2012). The full results of these four trials are shown in Table 7.

**4:** Results from trials of soap + water compared to hand sanitisers

**Study**	**Comparison** (see Table 4 for details of interventions)	**Reported outcomes**	**Results**
Azor‐Martinez 2018 cluster‐RCT Spain	Education and hand hygiene with soap and water (HH soap) vs hand hygiene with sanitiser (HH sanitiser) vs usual hand‐washing procedures	1. URI incidence rate ratio (primary) 2. Percentage difference in absenteeism days	1: HH soap vs control 0.94 (95% CI 0.82 to 1.08); HH sanitiser vs control 0.77 (95% CI 0.68 to 0.88); HH soap vs HH sanitiser 1.21 (95% CI 1.06 to 1.39) 2: HH soap 3.9% vs control 4.2% (P < 0.001); HH sanitiser 3.25% vs control 4.2% (P = 0.026); HH soap 3.9% vs HH sanitiser 3.25% (P < 0.001)
Pandejpong 2012 cluster‐RCT Thailand	Alcohol hand gel applied every 60 minutes vs every 120 minutes vs once before lunch (3 groups).	Absent days due to confirmed ILI/present days	0.017 in every hour group; 0.025 in every 2 hours group; 0.026 in before lunch group. Statistically significant difference between every hour group and before lunch group, and between every hour and every 2 hours groups
Savolainen‐Kopra 2012 cluster‐RCT Finland	Hand hygiene with soap and water (IR1 group) vs with alcohol‐based hand rub (IR2 group) vs control (none); intervention groups also received education	1. Number of respiratory infection episodes/week 2. Number of reported infection episodes/week 3. Number of reported sick leave episodes/week	1. 0.076 in IR1; 0.085 in IR2; 0.080 in control, NS 2: 0.097 in IR1; 0.107 in IR2; 0.104 in control, NS 3: 0.042 in IR1; 0.035 in IR2; 0.035 in control. Significantly higher in IR1 compared with control
Turner 2004a and Turner 2004b RCT Canada	Study 1. Ethanol vs salicylic acid 3.5% vs salicylic acid 1% and pyroglutamic acid 3.5% Study 2. Skin cleanser wipe vs ethanol (control)	% of volunteers infected with rhinovirus	7% in each intervention group; 32% in control (study 1) 22% in intervention, 30% in control (study 2)

CI: confidence interval cluster‐RCT: cluster‐randomised controlled trial HH: hand hygiene ILI: influenza‐like illness NS: non‐significant RCT: randomised controlled trial URI: upper respiratory infection vs: versus

##### Primary outcomes

###### 1. Numbers of cases of viral respiratory illness (including ARIs, ILI, and laboratory‐confirmed influenza)

In the trial by Azor‐Martinez 2018, ARI incidence was significantly higher in the soap‐and‐water group compared with the hand sanitiser group (rate ratio 1.21, 95% CI 1.06 to 1.39). In contrast, there was no significant difference between interventions in Savolainen‐Kopra 2012. In the rhinovirus challenge study (Turner 2004a; Turner 2004b), all hand sanitisers tested led to a significant lowering of infection rates, but no differences between sanitisers were observed. The study sample size was small.

###### 2. Adverse events related to the intervention

Two trials stated that no adverse events were observed (Pandejpong 2012; Savolainen‐Kopra 2012).

##### Secondary outcomes

###### 1. Deaths

Not reported.

###### 2. Severity of viral respiratory illness as reported in the studies

Not reported.

###### 3. Absenteeism

The authors of Azor‐Martinez 2018 also observed a significant benefit for hand sanitiser in reduction in days absent, whereas there was no difference between intervention groups in the Savolainen‐Kopra 2012 trial. The study on frequency of use of sanitiser found that use of sanitiser every hour significantly reduced days absent compared with use every two hours or with use only before the lunch break (Pandejpong 2012).

###### 4. Hospital admissions

Not reported.

###### 5. Complications related to the illness (e.g. pneumonia)

Not reported.

#### Comparison 8: Surface/object disinfection (with or without hand hygiene) compared to control

##### Primary outcomes

###### 1. Numbers of cases of viral respiratory illness (including ARIs, ILI, and laboratory‐confirmed influenza)

Six trials contributed data to this comparison (Ban 2015; Carabin 1999; Ibfelt 2015; Kotch 1994; McConeghy 2017; Sandora 2008). Full results of these trials are shown in Table 8. Five of the six trials combined disinfection with other interventions such as hand hygiene education, provision of hand hygiene products, and audits. Ban 2015 utilised a combination of provision of hand hygiene products, and cleaning and disinfection of surfaces, and demonstrated a significant reduction in ARI in the intervention group (OR 0.47, 95% CI 0.48 to 0.65). A similar result was seen in Carabin 1999, with a significant reduction in episodes of ARI. Two studies tested multi component interventions and observed no significant difference in ARI outcomes (Kotch 1994; McConeghy 2017).

**5:** Results from trials of surface/object disinfection (with or without hand hygiene) compared to control

**Study**	**Comparison** (see Table 4 for details of interventions)	**Reported outcomes**	**Results**
Ban 2015 cluster‐RCT China	Hand hygiene products, surface cleaning and disinfection provided to families and kindergartens vs none	1. Respiratory illness 2. Cough and expectoration	1. OR 0.47 for intervention to control (95% CI 0.38 to 0.59) 2. OR 0.56 (95% CI 0.48 to 0.65)
Carabin 1999 cluster‐RCT Canada	One‐off hygiene education and disinfection of toys with bleach vs none	Difference in incidence rate for URTI (cluster‐level result)	0.28 episodes per 100 child‐days lower in intervention group (95% CI 1.65 lower to 1.08 higher); URTI incidence rate IRR 0.80 (95% CI 0.68 to 0.93)
Ibfelt 2015 cluster‐RCT Denmark	Disinfectant washing of linen and toys by commercial company every 2 weeks vs usual care	Presence of respiratory viruses on surfaces	Statistically significant reduction in intervention group in adenovirus, rhinovirus, RSV, metapneumovirus, but not other viruses including coronavirus
Kotch 1994 RCT USA	Training in hand‐washing and diapering and disinfection of surfaces vs none	Respiratory illness incidence rate in: 1. children < 24 months; 2. children >= 24 months.	1. 14.78 episodes per child‐year in intervention; 15.66 in control 2. 12.87 in intervention; 11.77 in control
McConeghy 2017 RCT USA	Staff education, cleaning products, and audit of compliance and feedback vs none	Infection rates	Upper respiratory infections not reliably recorded or reported.
Sandora 2008 cluster‐RCT USA	Hand sanitiser and disinfection of classroom surfaces vs materials about good nutrition (control)	Absence due to respiratory illness (multivariable analysis)	Rate ratio 1.10 for intervention to control (95% CI 0.97 to 1.24)

CI: confidence interval cluster‐RCT: cluster‐randomised controlled trial IRR: incident rate ratio OR: odds ratio RCT: randomised controlled trial RSV: respiratory syncytial virus URTI: upper respiratory tract infection vs: versus

One trial compared disinfection alone to usual care (Ibfelt 2015). This study demonstrated a significant reduction in some viruses detected on surfaces in the childcare centres (adenovirus, rhinovirus, respiratory syncytial virus (RSV), and metapneumovirus), but not in other viruses, including coronavirus.

###### 2. Adverse events related to the intervention

Not reported.

##### Secondary outcomes

###### 1. Deaths

Not reported.

###### 2. Severity of viral respiratory illness as reported in the studies

Not reported.

###### 3. Absenteeism

Only one study measured this outcome (Sandora 2008), observing no significant difference between groups for the outcome of absence due to respiratory illness (rate ratio for intervention to control 1.10, 95% CI 0.97 to 1.24).

###### 4. Hospital admissions

Not reported.

###### 5. Complications related to the illness (e.g. pneumonia)

Not reported.

#### Comparison 9: Complex interventions compared to control

Complex interventions are either multifaceted environmental programmes (such as those in low‐income countries) or combined interventions including hygiene measures and gloves, gowns, and masks.

Four trials studied complex hygiene and sanitation interventions in low‐income country settings (Chard 2019; Hartinger 2016; Huda 2012; Najnin 2019). Full results from these studies are given in Table 9.

**6:** Results from trials of complex interventions compared to control

**Study**	**Comparison** (see Table 4 for details of interventions)	**Reported outcomes**	**Results**
**Complex hygiene and sanitation interventions compared to control**			
Chard 2019 cluster‐RCT Laos	Complex sanitation intervention and education vs none	Pupil‐reported symptoms of respiratory infection over 1 week	NS difference between groups. 29% of intervention group; 32% control group; adjusted risk ratio 1.08 (95% CI 0.95 to 1.23)
Hartinger 2016 cluster‐RCT Peru	Cooking and sanitation provision and education vs none	Number of ARI episodes per child‐year	NS difference between groups. Risk ratio for intervention to control 0.95 (95% CI 0.82 to 1.10)
Huda 2012 cluster‐RCT Bangladesh	Sanitation provision and education vs none	Respiratory illness	12.6% in intervention group; 13.0% in control group. Not adjusted for multiple outcome measurements. No CIs reported.
Najnin 2019 cluster‐RCT Bangladesh	Sanitation and behaviour change intervention (plus cholera vaccine) vs none	Respiratory illness in past 2 days	2.8% in intervention group; 2.9% in control group

ARI: acute respiratory infection CI: confidence interval cluster‐RCT: cluster‐randomised controlled trial NS: non‐significant RCT: randomised controlled trial vs: versus

##### Primary outcomes

###### 1. Numbers of cases of viral respiratory illness (including ARIs, ILI, and laboratory‐confirmed influenza)

All four trials of complex interventions observed no significant differences between groups in rates of viral respiratory illness.

###### 2. Adverse events related to the intervention

Not reported.

##### Secondary outcomes

###### 1. Deaths

Not reported.

###### 2. Severity of viral respiratory illness as reported in the studies

Not reported.

###### 3. Absenteeism

Not reported.

###### 4. Hospital admissions

Not reported.

###### 5. Complications related to the illness (e.g. pneumonia)

Not reported.

#### Comparison 10: Physical distancing/quarantine

We found three RCTs that assessed physical distancing/quarantine interventions. A quasi‐cluster‐RCT assessed the effectiveness of quarantining workers of one of two sibling companies in Japan whose family members developed an ILI during the 2009 to 2010 H1N1 influenza pandemic (Miyaki 2011). Workers in the intervention group were asked to stay home on full pay until five days after the household member(s) showed resolution of symptoms or two days after alleviation of fever. A second RCT conducted during the SARS‐CoV‐2 pandemic investigated whether attending fitness centres with physical distancing  was non‐inferior compared to no access in terms of COVID‐19 transmission (Helsingen 2021). The third study was a cluster‐RCT conducted during the SARS‐CoV‐2 pandemic that compared voluntary daily lateral flow device testing for seven days with negative contacts remaining at school to self‐isolation of school‐based COVID‐19 contacts for 10 days in a non‐inferiority design (Young 2021). 

##### Primary outcomes

###### 1. Numbers of cases of viral respiratory illness (including laboratory‐confirmed influenza and SARS‐CoV‐2)

Miyaki 2011 reported adherence with the intervention was 100%. In the intervention group 2.75% of workers contracted influenza, compared with 3.18% in the control group (Cox hazard ratio 0.799, 95% CI 0.66 to 0.97; P = 0.02), indicating that the rate of infection was reduced by 20% in the intervention group. However, the risk of a worker being infected was 2.17‐fold higher in the intervention group where workers stayed at home with their infected family members. The authors concluded that quarantining workers who have infected household members could be a useful additional measure to control the spread of respiratory viruses in an epidemic setting.

Helsingen 2021 reported 3016 participants were tested for SARS‐CoV‐2 resulting in one positive case in the fitness centre access arm versus zero in the no access arm at 14 days (risk difference (RD) 0.053%, 95% CI − 0.050 to 0.156%;  P = 0.32). In addition, 11 in the fitness centre access arm versus 27 in the no access arm tested positive for SARS‐CoV‐2 antibodies at one month (RD − 0.87%, 95% CI − 1.52% to − 0.23%; P = 0.001). The authors concluded that access to fitness centres with physical distancing and low population prevalence of SARS‐CoV‐2 infection did not increase risk of SARS‐CoV‐2 infection. 

Results from Young 2021 suggested no difference between the two treatment arms for SARS‐CoV‐2 infection (RR 0.96, 95% CI 0.75 to 1.22) leading the study authors to conclude non‐inferiority of daily contact testing of school‐based contacts (intervention) compared to self‐isolation (control). 

###### 2. Adverse events related to the intervention

Not reported.

##### Secondary outcomes

###### 1. Deaths

Not reported.

###### 2. Severity of viral respiratory illness as reported in the studies

Not reported.

###### 3. Absenteeism

Young 2021 reported COVID‐19 related absences from school were similar in the two treatment groups (RR 0.80, 95% CI  0.54 to 1.19). 

###### 4. Hospital admissions

Helsingen 2021 reported no hospital admissions in either treatment arm. 

###### 5. Complications related to the illness (e.g. pneumonia)

Not reported.

#### Comparison 11: Eye protection compared to control

##### Primary outcomes

###### 1. Numbers of cases of viral respiratory illness (including laboratory‐confirmed influenza and SARS‐CoV‐2)

We only identified one trial of eye protection which was a preprint only (Fretheim 2022a). This was a pragmatic RCT conducted in Norway from 2 February to 24 April 2022, where 3717 participants were randomised to an intervention group asked to wear glasses (e.g. sunglasses) for two weeks when close to others in public spaces. COVID‐19 cases in the national registry were 3.7% in the intervention group (68/1852) and 3.5% (65/1865) in the control group (RR 1.10, 95% CI 0.75 to 1.50). Positive COVID‐19 tests based on self‐reporting were 9.6% and 11.5% (RR 0.83, 95% CI 0.69 to 1.00). Given the high risk of bias and wide CIs, no policy conclusions can be drawn, but replication studies are clearly warranted. Almost a third of the participants reported respiratory infections. However, a lower proportion of those (215 participants) were in the intervention group compared to the control group (RR 0.90; 95% CI 0.82 to 0.99). 

###### 2. Adverse events related to the intervention

A total of 76 participants reported a negative experience from participating in the trial (53 in the intervention group and 23 in the control group). The most common complaint related to the combination of wearing glasses and face masks, and 21 participants in the intervention group cited fogging as an issue. Some participants reported feeling tired or uncomfortable wearing glasses, and a few participants complained of reduced vision when wearing sunglasses or reading glasses. In the control group some participants reported headaches from not being able to wear glasses, and one participant in the intervention group reported a fall due to reduced vision. 

##### Secondary outcomes

###### 1. Deaths

Not reported.

###### 2. Severity of viral respiratory illness as reported in the studies

Not reported.

###### 3. Absenteeism

Not reported.

###### 4. Hospital admissions

Not reported.

###### 5. Complications related to the illness, e.g. pneumonia

Not reported.

#### Comparison 12: Gargling/nose rinsing compared to control

Five trials investigated the effect of gargling/nose rinsing. Satomura 2005 compared throat gargling with povidone‐iodine versus tap water in healthy adults. Ide 2014 compared gargling with green tea versus tap water in high school students, and Goodall 2014 compared gargling with tap water with no gargling in university students. Two additional trials were conducted during the SARS‐CoV‐2 pandemic: Almanza‐Reyes 2021 compared silver mouth wash/nose rinse versus conventional mouthwashes and nose rinse in health workers; and Gutiérrez‐García 2022 compared neutral electrolysed water mouth and nose rinses versus no rinses in health workers.   

##### Primary outcomes

###### 1. Numbers of cases of viral respiratory illness (including ARIs, ILI, and laboratory‐confirmed influenza and SARS‐CoV‐2)

Satomura 2005 reported that gargling with tap water reduced the incidence of URTIs compared to the control group (usual care) (hazard ratio (HR) 0.60, 95% CI 0.39 to 0.95). Gargling with povidone‐iodine did not reduce the incidence of URTIs compared to the control group (HR 0.88, 95% CI 0.58 to 1.34).

Goodall 2014 found no difference in laboratory‐confirmed URTIs between the gargling (tap water) and no‐gargling groups (RR for gargling versus no gargling 0.82, 95% CI 0.53 to 1.26; P = 0.36).

In a meta‐analysis of gargling versus control based on two trials the pooled estimate of effect suggested little or no difference for the outcome of clinical URTI due to gargling (RR 0.91, 95% CI 0.63 to 1.31; 830 participants; Analysis 6.1) (Goodall 2014; Satomura 2005).

There was no difference in the incidence of laboratory‐confirmed influenza between high school students gargling with green tea compared with those using tap water (adjusted OR 0.69, 95% CI 0.37 to 1.28; P = 0.24) (Ide 2014). There was also no difference in the incidence of clinically defined influenza (adjusted OR 0.75, 95% CI 0.50 to 1.13; P = 0.17). However, the authors reported that adherence to the interventions amongst students was low.

Almanza‐Reyes 2021 reported the incidence of SARS‐CoV‐2 infection was statistically significantly lower in the silver mouth wash/nose rinse group (two out of 114, 1.8%) compared to the conventional mouthwash group (33 out of 117, 28.2%), and Gutiérrez‐García 2022 reported the incidence of COVID‐19‐positive cases in the nasal and oral rinses group was 1% compared to 13% in the control group (RR 0.09, 95% CI of 0.01 to 0.72). A meta‐analysis of these two studies showed a 93% reduction in risk of SARS‐CoV‐2 (RR 0.07, 95% CI 0.02 to 0.23; 394 participants; Analysis 6.2). 

###### 2. Adverse events related to the intervention

Satomura 2005 reported no adverse events during the 60‐day intervention period. Ide 2014 also did not observe any adverse events during the study. Goodall 2014 did not report on adverse effects. There were no adverse reactions in the study by Almanza‐Reyes 2021 or side effects in the study by Gutiérrez‐García 2022.  

##### Secondary outcomes

###### 1. Deaths

Not reported.

###### 2. Severity of viral respiratory illness as reported in the studies

Satomura 2005 reported that the mean peak score in bronchial symptoms was lower in the water gargling group (0.97) than in the povidone‐iodine gargling group (1.41) and the control group (1.40), P = 0.055. Other symptoms were not significantly different between groups. Goodall 2014 reported that symptom severity was greater in the gargling group for clinical and laboratory‐confirmed URTI, but this was not statistically significant (225.3 versus 191.8, and 210.5 versus 191.8, respectively). Ide 2014 did not report symptom or illness severity.

###### 3. Absenteeism

Not reported.

###### 4. Hospital admissions

Not reported.

###### 5. Complications related to the illness (e.g. pneumonia)

Not reported.

#### Comparison 13: Virucidal tissues compared to control

Two reports (three trials) conducted in the USA studied the effect of virucidal tissues (Farr 1988a; Farr 1988b; Longini 1988). Full results from these studies are given in Table 10.

**7:** Results from trials of virucidal tissues compared to control

**Study**	**Comparison**	**Reported outcomes**	**Results**
**Virucidal tissues compared with placebo or no tissues**			
Farr 1988a and Farr 1988b cluster‐RCT USA Trial 1 and Trial 2	Trial 1. Virucidal nasal tissues vs placebo vs none Trial 2. Virucidal nasal tissues vs placebo	Respiratory illnesses per person over 24 weeks Trial 1 Trial 2	Trial 1: 3.4 in tissues group; 3.9 in placebo group; 3.6 in no‐tissues group Trial 2: 3.4 in tissues group; 3.6 in placebo group NS
Longini 1988 DB‐PC RCT USA	Virucidal nasal tissues vs placebo	Secondary attack rate of viral infections (number of infections in household members of index case)	10.0 in intervention; 14.3 in placebo; NS

cluster‐RCT: cluster‐randomised controlled trial DB‐PC: double‐blind, placebo‐controlled NS: non‐significant RCT: randomised controlled trial vs: versus

##### Primary outcomes

###### 1. Numbers of cases of viral respiratory illness (including ARIs, ILI, and laboratory‐confirmed influenza)

The three trials of virucidal tissues reported no differences in infection rates between tissues and placebo, and between tissues and no tissues (Farr 1988a; Farr 1988b; Longini 1988).

###### 2. Adverse events related to the intervention

Farr 1988b reported cough in 4% of participants using virucidal tissues versus 57% in the placebo group, but 24% reported nasal burning in the virucidal tissue group versus 8% in the placebo group. Longini 1988 did not report on adverse effects.

##### Secondary outcomes

###### 1. Deaths

Not reported.

###### 2. Severity of viral respiratory illness as reported in the studies

Not reported.

###### 3. Absenteeism

Not reported.

###### 4. Hospital admissions

Not reported.

###### 5. Complications related to the illness (e.g. pneumonia)

Not reported.

## Discussion

### Summary of main results

See Table 11. 

**8:** Summary of main results of the review for the primary outcomes

**Interventions **	**RCT/cluster‐RCT (N = 78)**
Medical/surgical masks	**Masks (medical/surgical) compared to no masks** 9 trials in the community showed no effect on ILI (RR 0.95, 0.84 to 1.09) (Abaluck 2022; Aiello 2010; Alfelali 2020; Barasheed 2014; Canini 2010; Cowling 2008;; MacIntyre 2009;; MacIntyre 2016; Suess 2012); and 6 trials in the community showed no effect on laboratory‐confirmed influenza 95% CI RR 1.01 (0.72 to 1.42) (Aiello 2012; Alfelali 2020; Bundgaard 2021; Cowling 2008; MacIntyre 2009; Suess 2012). Two trials in health care workers where the control group wore masks if they were required provided inconclusive results with very wide confidence intervals (Jacobs 2009; MacIntyre 2015). **Medical/surgical masks versus other (non‐N95) masks*:*** 1 trial showed more ILI with cloth mask (RR 13.25, 1.74 to 100.97) (MacIntyre 2015); 1 trial showed no effect of catechin‐treated masks on influenza (adjusted OR 2.35, 0.40 to 13.72) (Ide 2016).
N95 respirator	**N95 respirators compared to medical/surgical masks** 3 trials showed no difference for clinical respiratory illness (RR 0.70, 0.45 to 1.10) (MacIntyre 2011; MacIntyre 2013; Radonovich 2019); 4 trials showed no difference for ILI (95% CI RR 0.81, 0.62 to 1.05) (Loeb 2009; MacIntyre 2009; MacIntyre 2011; Radonovich 2019); and 4 trials showed no difference for laboratory‐confirmed influenza (95% CI RR 1.06, 0.81 to 1.38) (Loeb 2009; MacIntyre 2009; MacIntyre 2011; Radonovich 2019). 4 trials conducted in HCWs: 3 trials showed no difference for clinical respiratory illness (RR 0.70, 0.45 to 1.10) (MacIntyre 2011; MacIntyre 2013; Radonovich 2019); 3 trials showed no difference for ILI (RR 0.64, 0.32 to 1.31) (Loeb 2009; MacIntyre 2011; Radonovich 2019); and 3 trials showed no difference for laboratory‐confirmed ILI (RR 1.02, 0.73 to 1.43) (Loeb 2009; MacIntyre 2011; Radonovich 2019).
Hand hygiene	**Hand hygiene compared to control** 19 trials found an effect on combined outcome (ARI or ILI or influenza) (RR 0.89, 0.83 to 0.94) (Ashraf 2020; Azor‐Martinez 2018; Biswas 2019; Correa 2012; Cowling 2008; Cowling 2009; Hubner 2010; Larson 2010; Little 2015; Millar 2016; Nicholson 2014; Ram 2015; Roberts 2000; Sandora 2005; Simmerman 2011; Stebbins 2011; Swarthout 2020; Teesing 2021; Zomer 2015); 9 trials showed an effect on ARI (RR 0.86, 0.81 to 0.90) (Ashraf 2020; Azor‐Martinez 2018; Correa 2012; Larson 2010; Little 2015; Millar 2016; Nicholson 2014; Sandora 2005; Swarthout 2020); 11 trials showed no effect on ILI (RR 0.94, 0.81 to 1.09) (Biswas 2019; Cowling 2008; Cowling 2009; Hubner 2010; Larson 2010; Little 2015; Ram 2015; Roberts 2000; Simmerman 2011; Teesing 2021; Zomer 2015); and 8 trials no effect on laboratory‐confirmed influenza (RR 0.91, 95% CI 0.63 to 1.30) (Biswas 2019; Cowling 2008; Cowling 2009; Hubner 2010; Larson 2010; Ram 2015; Simmerman 2011; Stebbins 2011).
Hand hygiene + medical/surgical masks	**Hand hygiene + medical/surgical masks compared to control** 7 trials showed no effect on ILI (95% CI RR 0.97, 0.80 to 1.19) (Aelami 2015; Aiello 2010; Aiello 2012; Cowling 2009; Larson 2010; Simmerman 2011; Suess 2012); and 4 trials showed no effect on laboratory‐confirmed influenza (RR 0.97, 0.69 to 1.36) (Cowling 2009; Larson 2010; Simmerman 2011; Suess 2012). **Hand hygiene + medical/surgical masks compared to hand hygiene** 3 trials showed no effect on ILI (RR 1.03, 0.69 to 1.53) or laboratory‐confirmed influenza (RR 0.99, 0.69 to 1.44) (Cowling 2009; Larson 2010; Simmerman 2011).
Soap + water compared to sanitiser, and comparisons of different types of sanitiser	**Soap + water compared to sanitiser, and comparisons of different types of sanitiser** 1 trial hand sanitiser was more effective than soap and water (Azor‐Martinez 2018); 1 trial there was no difference (Savolainen‐Kopra 2012). 2 trials in children antiseptic was more effective (Morton 2004; White 2001); 1 trial in children antiseptic = soap (Luby 2005). 1 trial hand sanitisers were better than placebo, but no difference between sanitisers (Turner 2004a); 1 trial no difference between different wipes (Turner 2004b).
Surface/object disinfection (with or without hand hygiene) compared to control	**Surface/object disinfection compared to control** 2 trials were effective on ARI (Ban 2015; Carabin 1999); 1 trial was effective for viruses detected on surfaces (Ibfelt 2015); 2 trials showed no difference in ARIs (Kotch 1994; McConeghy 2017).
Disinfection of living quarters	‐
Complex interventions	**Complex interventions compared to control** 4 trials in low‐income countries found no effect on respiratory viral illness (Chard 2019; Hartinger 2016; Huda 2012; Najnin 2019).
Physical interventions (masks, gloves, gowns combined)	‐
Gloves	‐
Gowns	‐
Physical distancing	**Physical distancing compared to self‐isolation** 1 trial reported 1 positive SARS‐CoV‐2 case in the fitness centre access arm versus 0 in the no access arm (risk difference 0.05%, 95% CI − 0.05 to 0.16%) (Helsingen 2021)
Quarantine in the community	**Quarantine compared to control** 1 trial effective for influenza (Cox hazard ratio 0.799, 95% CI 0.66 to 0.97) (Miyaki 2011). **Daily contact testing compared to self‐isolation** 1 trial showed non‐inferiority of daily contact testing of school‐based contacts compared to self‐isolation for SARS‐CoV‐2 (RR 0.96, 95% CI 0.75 to 1.22) (Young 2021)
Eye protection	**Glasses compared to no glasses** 1 pragmatic RCT conducted in Norway wearing any type of eyeglasses when close to other people outside their home (on public transport, in shopping malls etc.), over a 14‐day period. Positive COVID‐19 tests based on self‐reporting were 9.6% and 11.5% (RR 0.83, 95% CI 0.69 to 1.00) (Fretheim 2022a).
Gargling	**Gargling compared to control** 1 trial gargling with tap water was effective, povidone‐iodine was not effective (Satomura 2005); 1 trial gargling with green tea was not more effective than tap water (Ide 2014); 1 trial gargling with water was not effective (Goodall 2014); pooling of 2 trials showed no effect of gargling (RR 0.91, 95% CI 0.63 to 1.31) (Goodall 2014; Satomura 2005). **Mouth/nose rinse compared to control ** 2 trials found a large protective effect on SARS‐CoV‐2 (RR 0.07, 0.01 to 0.23) (Almanza‐Reyes 2021; Gutiérrez‐García 2022).
Virucidal tissues	**Virucidal tissues compared to control** 1 trial had a small effect (Farr 1988a) ("The study authors conclude that virucidal tissues have only a small impact upon the overall rate of natural acute respiratory illnesses"); 2 trials showed a non‐significant difference (Farr 1988b; Longini 1988).
Nose wash	‐

ARI: acute respiratory infection CI: confidence interval HCW: healthcare worker ILI: influenza‐like illness OR: odds ratio RCT: randomised controlled trial RR: risk ratio

#### 1. Medical/surgical masks compared to no masks

The pooled estimates of effect from randomised controlled trials (RCTs) and cluster‐RCTs for wearing medical/surgical masks compared to no masks in the community suggests probably little or no difference in interrupting the spread of influenza‐like illness (ILI)/COVID‐19 like illness (risk ratio (RR) 0.95, 95% confidence interval (CI) 0.84 to 1.09; moderate‐certainty evidence), or laboratory‐confirmed influenza/SARS‐CoV‐2 (RR 1.01, 95% CI 0.72 to 1.42; moderate‐certainty evidence). Six trials were cluster‐RCTs, with all participants in the intervention clusters required to wear masks, thus assessing both source control and personal protection. In two trials the clusters were households with a member with new influenza; neither trial found any protective effect (RR 1.03 in 105 households (Canini 2010); RR 1.21 in 145 households (MacIntyre 2009). In two trials the clusters were college dormitories during the influenza season; neither trial found any reduction (RR 1.10 in 37 dormitories (Aiello 2012); RR 0.90 in three dormitories (Aiello 2010)). Two studies were conducted during the COVID‐19 pandemic and their addition had minimal impact on the pooled estimate of effect previously reported from the earlier studies focused on influenza (Abaluck 2022; Bundgaard 2021). We excluded Aiello 2010 from meta‐analysis since we did not consider 'randomisation' of three clusters to three arms was a proper randomised trial.

Less than half of the trials comparing masks with no masks addressed harms of mask wearing (Canini 2010; Cowling 2008; MacIntyre 2015; Suess 2012). Warmth, respiratory difficulties, humidity, and general discomfort were the most frequently reported adverse events. Neither of the RCTs conducted during the COVID‐19 pandemic directly assessed harms of mask wearing. More adults reported no harms compared to children.

In one trial cloth masks were associated with a significantly higher risk of both ILI and laboratory‐confirmed respiratory virus infection in healthcare workers (HCWs) (MacIntyre 2015). In addition, filtration capacity of the two‐ply cotton cloth masks was found to be only 3% and markedly less than with medical/surgical masks based on standardised particle testing. The authors suggested moisture retention, poor filtration, and penetration of the virus through the mask as plausible explanations for the increased risk of infection.

We did not find any randomised trials assessing the effectiveness of barrier interventions using a combination of masks, gloves, and gowns.

#### 2. N95 respirators compared to medical/surgical masks

Comparisons between N95 respirators and medical/surgical masks, used as needed for exposure to at‐risk patients, for the outcomes of clinical respiratory illness and the outcome of laboratory‐confirmed influenza showed estimates of effect suggesting considerable uncertainty for any benefit of N95 respirators for the former outcome and probably little or no difference for the latter outcome. Five trials (four in healthcare settings and one in a household setting) compared N95/P2 respirators with medical/surgical masks. Pooling of three of these trials showed an estimate of effect suggesting considerable uncertainty as to whether there was any benefit comparing N95 respirators and medical/surgical face masks for the outcome of clinical respiratory illness (RR 0.70, 95% CI 0.45 to 1.10; very low‐certainty evidence), and that N95 respirators may make little or no difference for the outcome of ILI (RR 0.82, 95% CI 0.66 to 1.03; low‐certainty evidence), and probably little or no difference for the outcome of laboratory‐confirmed influenza (RR 1.10, 95% CI 0.90 to 1.34; moderate‐certainty evidence). The presence of imprecision (wide CIs) and heterogeneity, particularly for the more subjective and less precise outcomes of clinical respiratory illness and ILI compared to laboratory‐confirmed influenza infection, makes it difficult to assess whether there may be a benefit of either medical/surgical masks or N95/P2 respirators. Restricting the pooling to HCWs made no difference to the overall findings. The two trials with the largest event rates were quite consistent in their findings of no significant differences between N95 and medical/surgical masks for the outcomes of laboratory‐confirmed influenza and all laboratory‐confirmed viral infections (Loeb 2009; Radonovich 2019). Three of the trials contributing to this analysis were carried out by members of the same group (MacIntyre 2009; MacIntyre 2011; MacIntyre 2013).

In general, harms were poorly reported or not reported at all in trials comparing N95 respirators with surgical masks. General discomfort resulting in reduced wear adherence was the most frequently reported harm. 

#### 3. Hand hygiene compared to control

We found that the estimate of effect may offer a benefit for hand hygiene for the composite outcome 'acute respiratory infections (ARI) or ILI or influenza' (RR 0.89, 95% CI 0.83 to 0.94; low‐certainty evidence), and probably offers a benefit for the outcomes ARI alone (RR 0.86, 95% CI 0.81 to 0.90; moderate‐certainty evidence), and absenteeism (RR 0.64, 95% CI 0.58 to 0.71). An observed estimate of effect in favour of hand hygiene for laboratory‐confirmed influenza, but with wider CIs may be a consequence of smaller sample sizes in conjunction with a more rigorous outcome measure.

#### 4. Hand hygiene + medical/surgical masks compared to control

The estimate of effect of combined hand hygiene and medical/surgical mask interventions compared to control in six (mostly small) trials suggested that the intervention may make little or no difference for the outcomes ILI (RR 1.03, 95% CI 0.77 to 1.37), and laboratory‐confirmed influenza (four trials) (RR 0.97, 95% CI 0.69 to 1.36).

#### 5. Hand hygiene + medical/surgical masks compared to hand hygiene

We also found an estimate of effect suggesting that adding medical/surgical masks to hand hygiene compared to hand hygiene alone may make little or no difference for the outcomes ILI (RR 1.03, 95% CI 0.69 to 1.53; 3 trials), and laboratory‐confirmed influenza (RR 0.99, 95% CI 0.69 to 1.44).

#### 6. Medical/surgical masks compared to other (non‐N95) masks

One trial found that medical/surgical masks were more effective than cloth masks at reducing the rate of ILI (RR 13.25, 95% CI 1.74 to 100.97) (MacIntyre 2015), but the extremely wide CIs make this finding difficult to interpret. One trial did not find a benefit from catechin‐treated masks over untreated masks on influenza infection rates (adjusted odds ratio (OR) 2.35, 95% CI 0.40 to 13.72; P = 0.34) (Ide 2016).

Harms of wearing masks were reported in 40.4% of HCWs using medical/surgical masks, and in 42.6% of those wearing cloth masks (P = 0.45) (MacIntyre 2015). The penetration of particles was higher in cloth masks (97%) compared to medical/surgical masks (44%).

#### 7. Soap + water compared to sanitiser, and comparisons of different types of sanitiser

There were too few trials comparing different types of hand hygiene interventions to be certain of any true differences between soap and water, alcohol‐based hand sanitisers, or other types of interventions. Also, it is uncertain whether the incremental effect of adding virucidals or antiseptics to hand‐washing actually decreased the respiratory disease burden outside the confines of the rather atypical studies. The extra benefit may have been, at least in part, accrued by confounding additional routines.

#### 8. Surface/object disinfection (with or without hand hygiene) compared to control

We identified six trials on surface/object disinfection (with or without hand hygiene), and although they were heterogeneous (and therefore could not be pooled), three of them showed a clear benefit compared to controls (Ban 2015; Carabin 1999; Ibfelt 2015).

We found no RCTs of nose disinfection, or disinfection of living quarters, as described in observational studies reported in Jefferson 2011.

#### 9. Complex interventions compared to control

Four trials studied complex hygiene and sanitation interventions, all in low‐income country settings (Chard 2019; Hartinger 2016; Huda 2012; Najnin 2019). These trials could not be pooled due to the heterogeneity of the interventions and settings. All four trials found no significant differences between groups in the rates of viral respiratory illness.

#### 10. Physical distancing/quarantine compared to control

We identified one trial that evaluated the effect of quarantine and found a reduction in influenza transmission to co‐workers when those with infected household members stayed home from work (Miyaki 2011). However, staying home increased their risk of being infected two‐fold. Two studies conducted during the COVID‐19 pandemic on SARS‐cov‐2 transmission showed (1) non‐inferiority of daily contact testing of school‐based contacts (intervention) compared to self‐isolation (control) (Young 2021); and (2) access to fitness centres with physical distancing and low population prevalence of SARS‐CoV‐2 infection did not increase risk of SARS‐cov‐2 infection (Helsingen 2021). 

#### 11. Eye protection compared to control

We only identified one trial of eye protection which was a preprint only (Fretheim 2022a).

#### 12. Gargling compared to control

Three trials addressed the use of gargling in preventing respiratory infections (Goodall 2014; Ide 2014; Satomura 2005). Although the trials used a variety of liquids and different outcomes, pooling the results of the two trials that compared gargling with tap water versus control did not show a favourable effect in reducing URTIs (RR 0.91, 95% CI 0.63 to 1.31) (Goodall 2014; Satomura 2005). Two trials of mouthwash/nose rinse were conducted during the SARS‐cov‐2 pandemic in HCWs: Almanza‐Reyes 2021 compared silver mouth wash/nose rinse versus conventional mouthwashes and nose rinse; and Gutiérrez‐García 2022 compared neutral electrolysed water mouth and nose rinses versus no rinses. Both studies reported large protective effects of the intervention on SARS‐CoV‐2 infection with reported outcomes of  SARS‐C0V‐2 infection in 28.2% and 12.7% in the HCWs not using the interventions versus 1.8% and 1.2% in those using the intervention, despite the use of  full personal protective equipment (PPE) and the high outcome rates raise questions about risk of bias, and no data were provided about baseline rates in other settings with full use of PPE.

#### 13. Virucidal tissues compared to control

Two reports (three trials) identified in Jefferson 2011 studied the effect of virucidal tissues compared to placebo or no tissues (Farr 1988a; Farr 1988b; Longini 1988). These trials found no differences in infection rates and could not be pooled.

### Overall completeness and applicability of evidence

Several features need consideration before making generalisations based on the included studies.

The settings of the included studies, which were conducted over five decades, were heterogeneous and ranged from suburban schools, Carabin 1999, to emergency departments, intensive care units, and paediatric wards, Loeb 2009, in high‐income countries; slums in low‐income countries (Luby 2005); and an upper Manhattan immigrant Latino neighbourhood (Larson 2010). Few attempts were made to obtain socio‐economic diversity by (for example) involving more schools in the evaluations of the same programme. We identified only a few studies from low‐income countries, where the vast majority of the burden of ARIs lies and where inexpensive interventions are so critical. Additionally, limited availability of over‐the‐counter medications and national universal comprehensive health insurance provided with consequent physician prescription of symptomatic treatment may further limit the generalisability of findings.

The included trials generally reported few events and were conducted mostly during non‐epidemic periods with the exception of the trials carried out during the influenza H1N1 and SARS‐CoV‐2 pandemics. The large study by Radonovich 2019 is an exception as it crossed over two of the highest reporting years for influenza in the USA between 2010 and 2017 (Elflein 2019). None of the trials were conducted during pandemics of SARS‐CoV‐1or in outbreaks of Middle East respiratory syndrome (MERS).

Of the trials assessing the effect of masks, six were carried out in those at greater exposure (i.e. HCWs) (Jacobs 2009; Loeb 2009; MacIntyre 2011; MacIntyre 2013; MacIntyre 2015; Radonovich 2019). None of these studies included HCWs undertaking aerosol‐generating procedures, for which the World Health Organization (WHO) currently recommends the N95 or equivalent mask. Three trials on hand hygiene interventions were carried out in nursing homes, and included HCWs (McConeghy 2017; Temime 2018; Yeung 2011). The scarcity of RCTs on HCWs limits the generalisability of such results.

The variable quality of the methods of some studies is striking. Incomplete or no reporting of randomisation (Turner 2004a), blinding (Farr 1988a; Farr 1988b), numerators and denominators (Carabin 1999; Kotch 1994), interventions, and cluster coefficients in the relevant trials (Carabin 1999), led to a considerable loss of information. Potential biases were often not discussed. 

Inappropriate placebos caused design problems. In some studies the placebo probably carried sufficient effect to dilute the intervention effects (Longini 1988). Two valiant attempts with virucidal tissues probably failed because placebo handkerchiefs were impregnated with a dummy compound that stung the users' nostrils (Farr 1988a; Farr 1988b).

Some studies used impractical interventions. Volunteers subjected to the intervention hand cleaner (organic acids) were not allowed to use their hands between cleaning and virus challenge, so the effect of normal use of the hands on the intervention remains unknown (Turner 2004a; Turner 2004b). Two per cent aqueous iodine painted on the hands, although a successful antiviral intervention, causes unacceptable cosmetic staining, which is impractical for all but those at the highest risk of epidemic contagion (Gwaltney 1980).

Adherence with interventions, especially educational programmes, was a problem for many studies despite the importance of many such low‐cost interventions. Adherence with mask wearing varied; it was generally around 60% to 80%, but was reported to be as low as 40% (see Table 4). Overall, the logistics of carrying out trials that involve sustained behaviour change are demanding, particularly in challenging settings such as immigrant neighbourhoods or students' halls of residence.

The identified trials provided sparse and unsystematic data on adverse effects of the intervention, and few of the RCTs measured or reported adherence with the intervention, which is especially important for the use of medical/surgical masks or N95 respirators. No studies investigated how the level of adherence may have influenced the effect size.

We identified one study assessing the effects of eye protection (Fretheim 2022a), and we identified three studies on physical distancing/quarantine (Helsingen 2021; Miyaki 2011; Young 2021). The dearth of evidence and predominant setting of seasonal viral circulation limits generalisability of our findings to other contexts and any future epidemics due to other respiratory viruses such as the COVID‐19 pandemic although there have been increasing numbers of RCTs and cluster‐RCTs in the latter setting which are adding to the evidence base.

The two recent small trials from Mexico assessing local mouth/nose rinses airways prophylactic as interventions treatments report large but uncertain reductions in transmission to healthcare workers which warrant further study and replication by other investigator (Almanza‐Reyes 2021; Gutiérrez‐García 2022).

### Certainty of the evidence

We found the available evidence base identified through our search processes to be of variable quality. Reporting of sequence generation and allocation concealment were poor in 30% to 50% of studies across the categories of intervention comparisons. Given the nature of the intervention comparison, blinding of treatment allocation after randomisation was rarely achieved. Although blinding of outcome assessment is highly feasible and desirable, most outcomes were assessed by self‐reports. Outcomes in some studies were poorly defined, with a lack of clarity as to the possible aetiological agents (bacterial versus viral). Some studies used laboratory‐confirmed outcomes, both adding precision and avoiding indirectness by having an accurate outcome measure and lowering the risk of bias (see Table 12 for heterogeneity of trial outcome definitions). We found no evidence of selective reporting of outcomes within the included studies. We believe publication bias is unlikely, as the included studies demonstrated a range of effects, both positive and negative, over all study sizes. The variable quality of the studies hampers drawing any firm conclusions.

**9:** Trial authors’ outcome definitions

**Study**	**Outcome definitions**
**Masks (n = 16)**	
Abaluck 2022 cluster‐RCT Bangladesh	COVID‐19 symptoms as per the WHO case definition of probable COVID‐19 given epidemiological risk factors: (i) fever and cough; (ii) 3 or more of the following symptoms (fever, cough, general weakness and/or fatigue, headache, myalgia, sore throat, coryza, dyspnoea, anorexia, nausea, and/or vomiting, diarrhoea, and altered mental status); or (iii) loss of taste or smell. The owner of the household’s primary phone completed surveys by phone or in‐person at weeks 5 and 9 after the start of the intervention. They were asked to report symptoms experienced by any household member consistent with the WHO. COVID‐19 case definition. Laboratory: seropositivity was defined by having detectable IgG antibodies in blood samples against SARS‐CoV‐2, using the SCoV‐2 Detect™ IgG ELISA kit (InBios, Seattle, Washington). This assay detects IgG antibodies against the spike protein subunit (S1) of SARS‐CoV‐2. Safety: harms were not directly assessed in this study, but it is stated no adverse events were reported.
Alfelali 2020 cluster‐RCT Haj in Makkah, Saudi Arabia	Laboratory: swabs were placed it into UTM™ (COPAN) viral transport media. Swabs labelled with the participant’s unique barcode number were stored in an icebox at –20˚C before being re‐stored by day’s end in a –80˚C freezer at the laboratory of the Hajj Research Center at Umm Al‐Qura University, Makkah. After Hajj, these swabs were shipped in refrigerated or cold containers to the Centre for Infectious Disease and Microbiology Laboratory Services, Westmead Hospital, NSW, Australia. There, nucleic acid was extracted with the Qiagen bioROBOT EZ instrument (Qiagen, Valencia, CA), and amplification was performed using the Roche LC 480 (Roche Diagnostics GmbH, Mannheim, Germany) instrument. Respiratory viruses were detected using a real‐time, multiplex reverse transcription polymerase chain reaction assay targeting human coronaviruses (OC43, 229E and NL63), influenza A and B viruses, respiratory syncytial virus (RSV), parainfluenza viruses 1 to 3, human metapneumovirus, rhinovirus, enterovirus and adenovirus. Middle East respiratory syndrome coronavirus (MERS‐CoV) assay targeting the upstream region of the E gene (upE) was also performed. Safety: harms of using face masks were difficulty in breathing (26.2%); discomfort (22%); and a small minority (3%) reported feeling hot, sweating, a bad smell or blurred vision with eyeglasses.
Bundgaard 2021 RCT Denmark	Laboratory: viral RNA was extracted from swab samples in DNA/RNA Shield (Zymo Research) using Quick‐RNA Microprep Kit (Zymo Research) with the below modifications. 200 µl samples were incubated for 1 min with proteinase K (Qiagen) in a final concentration of 0.2 µg/µl prior to treatment with lysis buffer (Quick‐RNA Microprep Kit). Only a single washing step using 400 µl RNA Wash Buffer (Quick‐RNA Microprep Kit) was performed before elution in 15µl RNase free water. Participants tested for SARS‐CoV‐2 IgM and IgG antibodies in whole blood using a point‐of‐care test (Lateral Flow test [Zhuhai Livzon Diagnostics]) according to the manufacturer's recommendations. After puncturing a fingertip with a lancet, they withdrew blood into a capillary tube and placed 1 drop of blood followed by 2 drops of saline in the test chamber in each of the 2 test plates (IgM and IgG). Safety: harms were not mentioned as an outcome in the methods, but psychological adverse effects were mentioned, and 14% reported adverse reactions from other people regarding wearing a face mask.
Cowling 2008 cluster‐RCT Hong Kong	Laboratory: QuickVue Influenza A+B rapid test Viral culture on MDCK (Madin‐Darby canine kidney cells) Samples were harvested using NTS, but the text refers to a second procedure from June 2007 onwards with testing for influenza viruses on index participants with a negative QuickVue result but a fever ≥ 38 °C who were also randomised and further followed up. Data on clinical signs and symptoms were collected for all participants, and an additional NTS was collected for later confirmation of influenza infection by viral culture. It is noteworthy that dropout was higher in households of index participants who had a negative result on the rapid influenza test (25/44, 57%) compared to those who had a positive result (45/154, 29%). Effectiveness: secondary attack ratios (SAR): SAR is the proportion of household contacts of an index case who subsequently were ill with influenza (symptomatic contact individuals with at least 1 NTS positive for influenza by viral culture or PCR) 3 clinical definitions were used for secondary analysis: fever ≥ 38 °C or at least 2 of the following symptoms: headache, coryza, sore throat, muscle aches and pains; at least 2 of the following S/S: fever ≥ 37.8 °C, cough, headache, sore throat and muscle aches and pains; and fever of ≥ 37.8 °C plus cough or sore throat. Safety: harms were not mentioned as an outcome in the methods, but it was reported in the results that there were no adverse events.
Jacobs 2009 RCT Japan	Laboratory‐confirmation not reported. Effectiveness: URTI is defined on the basis of a symptom score with a score > 14 being a URTI according to Jackson’s 1958 criteria ("Jackson score"). These are not explained in text, although the symptoms are listed in Table 3 (any, sore throat, runny nose, stuffy nose, sneeze, cough, headache, earache, feel bad) together with their mean and scores (SD) by intervention arm. Safety: the text does not mention or report harms. These appear to be indistinguishable from URTI symptoms (e.g. headache, which is reported as of significantly longer duration in the intervention arm). Compliance is self‐reported as high (84.3% of participants).
Loeb 2009 cluster‐RCT HCW Canada	Clinical respiratory illness, influenza‐like illness, and laboratory‐confirmed respiratory virus infection. Clinical respiratory illness, defined as 2 or more respiratory symptoms or 1 respiratory symptom and a systemic symptom. Influenza‐like illness, defined as fever ≥ 38 °C plus 1 respiratory symptom. Laboratory‐confirmed viral respiratory infection. Laboratory confirmation was by nucleic acid detection using multiplex RT‐PCR for 17 respiratory viruses. Safety: harms were not mentioned as an outcome in the methods, but it is stated in the results that no adverse events were reported by participants.
MacIntyre 2009 cluster‐RCT Australia	Eligibility criteria were stipulated as follows: the household contained > 2 adults > 16 years of age and 1 child 0 to 15 years of age; the index child had fever (temperature > 37.8 °C) and either a cough or sore throat; the child was the first and only person to become ill in the family in the previous 2 weeks; adult caregivers consented to participate in the study; and the index child was not admitted to the hospital. Definitions used for outcomes: ILI defined by the presence of fever (temperature > 37.8 °C), feeling feverish or a history of fever, > 2 symptoms (sore throat, cough, sneezing, runny nose, nasal congestion, headache), or 1 of the symptoms listed plus laboratory confirmation of respiratory viral infection. Laboratory confirmation: multiplex RT‐PCR tests to detect influenza A and B and RSV, PIV types 1 to 3, picornaviruses (enteroviruses or rhinoviruses), adenoviruses, coronaviruses 229E and OC43, and hMPV plus > 1 symptom Effectiveness: presence of ILI or a laboratory diagnosis of respiratory virus infection within 1 week of enrolment. Safety: harms not mentioned as an outcome in the methods, but it is reported in the results that more than 50% of participants reported concerns with mask wearing, mainly that wearing a face mask was uncomfortable, but there were no significant differences between the P2 (N95) and surgical mask groups. Other concerns were that the child did not want the parent wearing a mask.
Aiello 2010 cluster‐RCT USA	Laboratory details are described in appendix. Effectiveness: ILI, defined as cough and at least 1 constitutional symptom (fever/feverishness, chills, headache, myalgia). ILI cases were given contact nurses phone numbers to record the illness and paid USD 25 to provide a throat swab. 368 participants had ILI, 94 of which had a throat swab analysed by PCR. 10 of these were positive for influenza (7 for A and 3 for B), respectively by arm 2, 5 and 3 using PCR, 7 using cell culture. Safety: no outcomes on harms planned or reported.
Canini 2010 cluster‐RCT USA	The primary endpoint was the proportion of household contacts who developed an ILI during the 7 days following inclusion. Exploratory cluster‐level efficacy outcome, the proportion of households with 1 or more secondary illness in household contacts. A temperature over 37.8 °C with cough or sore throat was used as primary clinical case definition. The authors also used a more sensitive case definition based on a temperature over 37.8 °C or at least 2 of the following: sore throat, cough, runny nose, or fatigue. Safety: adverse reactions due to mask wearing were reported, with 38 (75%) participants in the intervention arm experiencing discomfort with mask use due to warmth (45%), respiratory difficulties (33%), and humidity (33%). Children wearing children face masks reported feeling pain more frequently than other participants wearing adult face masks (P = 0.036).
Aiello 2012 cluster‐RCT in halls of residence in the USA	Clinically verified ILI ‐ case definition (presence of cough and at least 1 or more of fever/feverishness, chills, or body aches) Laboratory‐confirmed influenza A or B. Throat swab specimens were tested for influenza A or B using real‐time PCR. Safety: no outcomes on harms planned or reported.
Barasheed 2014 cluster‐RCT Saudi Arabia	Laboratory: 2 nasal swabs from all ILI cases and contacts. 1 for influenza POCT using the QuickVue Influenza (A+B) assay (Quidel Corporation, San Diego, USA) and 1 for later NAT for influenza and other respiratory viruses. However, there was a problem with getting POCT on time during Hajj. Effectiveness: to assess the effectiveness of face masks in the prevention of transmission of ILI. ILI was defined as subjective (or proven) fever plus 1 respiratory symptom (e.g. dry or productive cough, runny nose, sore throat, shortness of breath). Safety: no outcomes on harms planned or reported.
MacIntyre 2011 cluster‐RCT China	Clinical respiratory illness Influenza‐like illness Laboratory‐confirmed viral respiratory infection Laboratory‐confirmed influenza A or B Clinical respiratory illness, defined as 2 or more respiratory or 1 respiratory symptom and a systemic symptom. Influenza‐like illness, defined as fever ≥ 38 °C plus 1 respiratory symptom (i.e. cough, runny nose, etc.). Laboratory‐confirmed viral respiratory infection (detection of adenoviruses, human metapneumovirus, coronavirus 229E ⁄ NL63, parainfluenza viruses 1, 2, and 3, influenza viruses A and B, respiratory syncytial virus A and B, rhinovirus A/B and coronavirus OC43/HKU1 by multiplex PCR). Laboratory‐confirmed influenza A or B. Adherence with mask/respirator use. Safety: adherence and adverse effects of mask wearing were collected at exit interviews 4 weeks' post study. Significantly higher adverse events with N95 respirator compared to medical mask for discomfort, headache, difficulty breathing, nose pressure, trouble communicating, not wearing, and unspecified “other” side effects. Over 50% of those wearing N95 respirators reported adverse events. Of those wearing medical masks versus N95 respirators, 85.5% (420/491) versus 47.4% (447/943) reported no adverse events (P < 0.001), respectively.
MacIntyre 2013 cluster‐RCT China	Laboratory: Laboratory‐confirmed viral respiratory infection in symptomatic participants, defined as detection of adenoviruses; human metapneumovirus; coronaviruses 229E/NL63 and OC43/HKU1; parainfluenza viruses 1, 2, and 3; influenza viruses A and B; respiratory syncytial viruses A and B; or rhinoviruses A/B by NAT using a commercial multiplex PCR (Seegen, Inc., Seoul, Korea). Laboratory‐confirmed influenza A or B in symptomatic participants. Laboratory‐confirmed bacterial colonisation in symptomatic participants, defined as detection of *Streptococcus pneumoniae*, *Legionella*, *Bordetella pertussis*, *Chlamydia*, *Mycoplasma pneumoniae*, *or Haemophilus influenzae* type B by multiplex PCR (Seegen, Inc.). Effectiveness: clinical respiratory illness defined as 2 or more respiratory symptoms or 1 respiratory symptom and a systemic symptom. ILI defined as fever (38 °C) plus 1 respiratory symptom. Safety: adverse effects measured using a semi‐structured questionnaire. Investigators stated that there was higher reported adverse effects and discomfort of N95 respirators compared with the other 2 arms. In terms of comfort, 52% (297 of 571) of the medical mask arm reported no problems, compared with 62% (317 of 512) of the targeted arm and 38% (217 of 574) of the N95 arm (P < 0.001).
MacIntyre 2015 cluster‐RCT Vietnam	Clinical respiratory illness, influenza‐like illness, and laboratory‐confirmed respiratory virus infection. Clinical respiratory illness, defined as 2 or more respiratory symptoms or 1 respiratory symptom and a systemic symptom. Influenza‐like illness, defined as fever ≥ 38 °C plus 1 respiratory symptom. Laboratory‐confirmed viral respiratory infection. Laboratory confirmation was by nucleic acid detection using multiplex RT‐PCR for 17 respiratory viruses. Safety: adverse events associated with face mask use were reported in 40.4% (227/562) of HCWs in the medical/surgical mask arm and 42.6% (242/568) in the cloth mask arm (P = 0.45). The most frequently reported adverse events were: general discomfort (35.1%; 397/1130) and breathing problems (18.3%; 207/1130). The rate of ILI was higher in the cloth mask arm compared to medical/surgical masks (RR 13.25, 95% CI 1.74 to 100.97).
MacIntyre 2016 cluster‐RCT China	Clinical respiratory illness, influenza‐like illness, and laboratory‐confirmed viral respiratory infection. Clinical respiratory illness, defined as 2 or more respiratory symptoms (cough, nasal congestion, runny nose, sore throat, or sneezes) or 1 respiratory symptom and a systemic symptom (chill, lethargy, loss of appetite, abdominal pain, muscle or joint aches). Influenza‐like illness, defined as fever ≥ 38 °C plus 1 respiratory symptom. Laboratory‐confirmed viral respiratory infection, defined as detection of adenoviruses, human metapneumovirus, coronaviruses 229E/NL63 and OC43/HKU1, parainfluenza viruses 1, 2, and 3, influenza viruses A and B, respiratory syncytial virus A and B, or rhinovirus A/B by NAT using a commercial multiplex PCR. Safety: no outcomes on harms planned or reported.
Radonovich 2019 cluster‐RCT USA	Laboratory. Primary outcome: incidence of laboratory‐confirmed influenza, defined as: detection of influenza A or B virus by RT‐PCR in an upper respiratory specimen collected within 7 days of symptom onset; detection of influenza from a randomly obtained swab from an asymptomatic participant; and influenza seroconversion (symptomatic or asymptomatic), defined as at least a 4‐fold rise in haemagglutination inhibition antibody titres to influenza A or B virus between pre‐season and postseason serological samples deemed not attributable to vaccination. Effectiveness. Secondary outcomes: incidence of 4 measures of viral respiratory illness or infection as follows: acute respiratory illness with or without laboratory confirmation; laboratory‐detected respiratory infection, defined as detection of a respiratory pathogen by PCR or serological evidence of infection with a respiratory pathogen during the study surveillance period(s), which was added to the protocol prior to data analysis; and laboratory‐confirmed respiratory illness, identified as previously described (defined as self‐reported acute respiratory illness plus the presence of at least PCR–confirmed viral pathogen in a specimen collected from the upper respiratory tract within 7 days of the reported symptoms and/or at least a 4‐fold rise from pre‐intervention to postintervention serum antibody titres to influenza A or B virus). Influenza‐like illness, defined as temperature of at least 100 °F (37.8 °C) plus cough and/or a sore throat, with or without laboratory confirmation. Safety: 19 participants reported skin irritation or worsening acne during years 3 and 4 at 1 site in the N95 respirator group.
**Hand and hygiene (n = 35)**	
Alzaher 2018 cluster‐RCT Saudi Arabia	Episode of URI was defined as having 2 of the following symptoms for a day or 1 of the symptoms for 2 or more consecutive days: 1) a runny nose, 2) a stuffy or blocked nose or noisy breathing, 3) sneezing, 4) a cough, 5) a sore throat, and 6) feeling hot, having a fever or a chill.
Arbogast 2016 cluster‐RCT USA	ICD‐9 used: 46611: acute bronchiolitis due to respiratory syncytial virus, 46619: acute bronchiolitis due to other infectious organisms, 4800: pneumonia due to adenovirus, 4809: viral pneumonia, unspecified, 4870: influenza with pneumonia, 07999: unspecified viral infection, 4658: acute upper respiratory infections of other multiple sites, 4659: acute upper respiratory infections of unspecified site, 4871: influenza with other respiratory manifestations.
Ashraf 2020 cluster‐RCT Bangladesh	Main outcome: 7‐day prevalence of acute respiratory infection (ARI), defined as caregiver‐reported symptoms of persistent cough or panting, wheezing, or difficulty breathing (1 or 2) in the 7 days before the interview.
Azor‐Martinez 2016 RCT Spain	Upper respiratory illness was defined as 2 of the following symptoms during 1 day, or 1 of the symptoms for 2 consecutive days: (1) runny nose; (2) stuffy or blocked nose or noisy breathing; (3) cough; (4) feeling hot or feverish or having chills; (5) sore throat; or (6) sneezing.
Azor‐Martinez 2018 RCT Spain	Respiratory illness (RI) was defined as the presence of 2 of the following symptoms during 1 day or the presence of 1 of the symptoms for 2 consecutive days: (1) runny nose, (2) stuffy or blocked nose or noisy breathing, (3) cough, (4) feeling hot or feverish or having chills, (5) sore throat, or (6) sneezing. ICD‐10 and ICD‐9 diagnosis codes used: nonspecific upper respiratory tract infection (465.9), otitis media (382.9), pharyngotonsillitis (463), lower respiratory tract infections (485 and 486), acute bronchitis (490), and bronchiolitis (466.19). Study authors combined the bronchopneumonia code (485) and pneumonia code (486) under the label “lower respiratory tract infections.” If > 1 antibiotic was prescribed during an episode, they used the first prescription for analysis. The final diagnosis was done by the medical researchers on the basis of the symptoms described above and a review of the medical history of children with RIs.
Biswas 2019 cluster‐RCT Bangladesh	Influenza‐like illness: an ILI episode was defined as measured fever > 38 °C or subjective fever and cough. Laboratory‐confirmed influenza Nasal swabs for real‐time RT‐PCR.
Correa 2012 cluster‐RCT Colombia	Acute respiratory infection was defined as 2 or more of the following symptoms for at least 24 hours, lasting at least 2 days: runny, stuffy, or blocked nose or noisy breathing; cough; fever, hot sensation, or chills; and/or sore throat. Ear pain alone was considered ARI alternately.
Cowling 2009 cluster‐RCT Hong Kong	Laboratory‐confirmed of influenza virus infection by RT‐PCR for influenza A and B virus. Clinical influenza‐like illness: used 2 clinical definitions of influenza based on self‐reported data from the symptom diaries as secondary analyses. The first definition of clinical influenza was at least 2 of the following signs and symptoms: temperature 37.8 °C or greater, cough, headache, sore throat, and myalgia; the second definition was temperature 37.8 °C or greater plus cough or sore throat.
DiVita 2011 (conference abstract) RCT Bangladesh	Influenza‐like illness was defined as fever in children < 5 years old and fever with cough or sore throat in individuals > 5 years old.
Feldman 2016 cluster‐RCT Israel	Infectious diseases grouped into diarrhoeal, respiratory, and skin infection. Based on ICD‐9, but no supplementary material was accessible for further definition (Supplementary Material C lists all ICD‐9 diagnoses tallied in this ”outcome”).
Gwaltney 1980 RCT USA	Viral cultures and serology if rhinovirus in laboratory‐inoculation
Hubner 2010 RCT Germany	Assessing illness rates due to common cold and diarrhoea. Collecting data on illness symptoms (common cold, sinusitis, sore throat, fever, cough, bronchitis, pneumonia, influenza, diarrhoea) and associated absenteeism at the end of every month. Definitions of symptoms were given to the participants as part of the individual information at the beginning of the study. Whilst most symptoms are quite self‐explanatory, "influenza" and "pneumonia" are specific diagnoses that were confirmed by professional diagnosis only. Similarly, (self‐) diagnosis of "fever" required objective measurement with a thermometer.
Ladegaard 1999 RCT Denmark	Laboratory: serological evidence Effectiveness: influenza‐like illness (described as fever, history of fever or feeling feverish in the past week, myalgia, arthralgia, sore throat, cough, sneezing, runny nose, nasal congestion, headache). However, a positive laboratory finding for influenza converts the ILI definition into one of influenza.
Larson 2010 cluster‐RCT USA	Study goals: rates of symptoms and secondary transmission of URIs, incidence of virologically confirmed influenza, knowledge of prevention and treatment strategies for influenza and URIs, and rates of influenza vaccination. Laboratory‐confirmed influenza: nasal swabs to test for influenza types A and B as well as other common respiratory viruses by rapid culture (R‐Mix, Diagnostic Hybrids, Inc., Athens, OH, USA). PCR and subtyping of the samples was done during the second half of the second year of the study. Influenza‐like illness: CDC definition of ILI from the Sentinel Physicians' Network was used to determine when masks should be worn: “temperature of ≥37.8°C and cough and/or sore throat in the absence of a known cause other than influenza". Episodes of URI = upper respiratory infection: not clear, no explicitly stated definition, reported that the most commonly reported URI symptoms are cough or rhinorrhoea.
Little 2015 RCT England	Respiratory tract infections defined as 2 symptoms of an RTI for at least 1 day or 1 symptom for 2 consecutive days. For reported ILI, study authors did not use WHO or CDC definitions because these definitions require measured temperature, and thus were not appropriate (participants were not included after a clinical examination), and they did not use the European Centre for Disease Prevention and Control definition (1 systemic and 1 respiratory symptom) because, according to the international influenza collaboration, this definition does not necessarily differentiate ILI from a common cold. Influenzanet suggests making high temperature a separate element. Their pragmatic definition of ILI therefore required a high temperature (feeling very hot or very cold; or measured temperature > 37.5 °C), a respiratory symptom (sore throat, cough, or runny nose), and a systemic symptom (headache, severe fatigue, severe muscle aches, or severe malaise).
Luby 2005 RCT Pakistan	Defined pneumonia in children according to the WHO clinical case definition: cough or difficulty breathing with a raised respiratory rate (> 60 per minute in individuals younger than 60 days old, > 50 per minute for those aged 60 to 364 days, and > 40 per minute for those aged 1 to 5 years)
Millar 2016 cluster‐RCT USA	Medically attended, outpatient cases of acute respiratory infection in the study population. The case definition was any occurrence of the following International Classification of Disease, 9 Revision, Clinical Modification (ICD‐9) symptom or disease‐specific codes: 460 to 466, 480 to 488, and specifically 465.9, 482.9, 486, and 487.1. **Acute respiratory infections (460 to 466)** 460 Acute nasopharyngitis (common cold) 461 Acute sinusitis 462 Acute pharyngitis 463 Acute tonsillitis 464 Acute laryngitis and tracheitis 465 Acute upper respiratory infections of multiple or unspecified sites 466 Acute bronchitis and bronchiolitis **Pneumonia and influenza (480 to 488)** 480 Viral pneumonia 481 Pneumococcal pneumonia (*Streptococcus pneumoniae* pneumonia) 482 Other bacterial pneumonia 483 Pneumonia due to other specified organism 484 Pneumonia in infectious diseases classified elsewhere 485 Bronchopneumonia, organism unspecified 486 Pneumonia, organism unspecified 487 Influenza 488 Influenza due to identified avian influenza virus 465.9 Acute upper respiratory infections of unspecified site 482.9 Bacterial pneumonia NOS 487.1 Diagnosis of influenza with other respiratory manifestations
Morton 2004 cluster‐RCT Cross‐over study USA	Respiratory illnesses defined by symptoms of upper respiratory infections such as nasal congestion, cough, or sore throat, in any combination, with or without fever
Nicholson 2014 cluster‐RCT India	Acute respiratory infections Operational definitions for all the illnesses were taken from Black's Medical Dictionary. ARIs defined as "Pneumonia, cough, fever, chest pain and shortness of breath, cold, inflammation of any or all of the airways, that is, nose, sinuses, throat, larynx, trachea and bronchi".
Pandejpong 2012 cluster‐RCT Thailand	Influenza‐like illness defined if 2 or more symptoms of stuffy nose, cough, fever or chills, sore throat, headache, diarrhoea, presence of hand, foot, or mouth ulcers.
Priest 2014 cluster‐RCT New Zealand	Respiratory illness was defined as an episode of illness that included at least 2 of the following caregiver‐reported symptoms for 1 day, or 1 of these symptoms for 2 days (but not fever alone): runny nose, stuffy or blocked nose or noisy breathing, cough, fever, sore throat, or sneezing.
Ram 2015 RCT Bangladesh	Influenza‐like illness Age‐specific definitions of ILI. For individuals ≥ 5 years old, ILI was defined as history of fever with cough or sore throat. For children < 5 years old, ILI was defined as fever; study authors used this relatively liberal case definition in order to include influenza cases with atypical presentations in children. Laboratory‐confirmed influenza infection Oropharyngeal swabs from index case patients for laboratory testing for influenza. All swabs were tested by PCR for influenza A and B, with further subtyping of influenza A isolates.
Roberts 2000 cluster‐RCT Australia	The symptoms of acute upper respiratory illness elicited from parents were: a runny nose, a blocked nose, and cough. Study authors used a definition of colds based on a community intervention trial of virucidal impregnated tissues. A cold was defined as either 2 symptoms for 1 day or 1 of the respiratory symptoms for at least 2 consecutive days, but not including 2 consecutive days of cough alone. Study authors defined a new episode of a cold as the occurrence of respiratory symptoms after a period of 3 symptom‐free days.
Sandora 2005 cluster‐RCT USA	The overall rates of secondary respiratory and GI illness. Respiratory illness was defined as 2 of the following symptoms for 1 day or 1 of the symptoms for 2 consecutive days: (1) runny nose; (2) stuffy or blocked nose or noisy breathing; (3) cough; (4) fever, feels hot, or has chills; (5) sore throat; and (6) sneezing. An illness was considered new or separate when a period of at least 2 symptom‐free days had elapsed since the previous illness. An illness was defined as a secondary case when it began 2 to 7 days after the onset of the same illness type (respiratory or GI) in another household member.
Savolainen‐Kopra 2012 cluster‐RCT Finland	Nasal and pharyngeal stick samples from participants with respiratory symptoms
Simmerman 2011 cluster‐RCT Thailand	Influenza‐like illness defined by WHO as fever plus cough or sore throat, based on self‐reported symptoms. Laboratory‐confirmed secondary influenza virus infections amongst household members described as the secondary attack rate. The secondary influenza virus infection was defined as a positive rRT‐PCR result on days 3 or 7 or a four‐fold rise in influenza HI antibody titres with the virus type and subtype matching the index case.
Stebbins 2011 cluster‐RCT USA	The primary outcome was an absence episode associated with an influenza‐like illness that was subsequently laboratory‐confirmed as influenza A or B. The following CDC definition for ILI was used: fever ≥ 38 °C with sore throat or cough.
Swarthout 2020 cluster‐RCT Kenya	The primary outcome in this study is ARI symptoms ‐ defined as having caregiver‐reported cough or difficulty breathing, including panting or wheezing, within 7 days before the interview ‐ in children younger than 3 years. Prespecified secondary outcomes in this study include difficulty breathing, including panting or wheezing, in the past 7 days (a more specific indicator of respiratory infection than a cough alone); ARI symptoms presenting with fever in the past 7 days (a potentially more severe infection); and enumerator‐observed runny nose (an objective outcome).
Talaat 2011 cluster‐RCT Egypt	Nasal swab for QuickVue test for influenza A and B viruses. Influenza‐like illness (defined as fever > 38 °C and either cough or sore throat).
Teesing 2021 cluster‐RCT The Netherlands	Incidence of gastroenteritis, ILI, assumed pneumonia, UTIs using the McGeer criteria, and infections caused by MRSA.
Temime 2018 cluster‐RCT France	ARIs were defined as the combination of at least 1 respiratory symptom and 1 symptom of systemic infection.
Turner 2004b RCT Canada	Virologic assays
Turner 2012 RCT USA	Laboratory‐confirmed rhinovirus infection by PCR assay. Common cold illness was defined as the presence of any of the symptoms of nasal obstruction, rhinorrhoea, sore throat, or cough on at least 3 consecutive days. Illnesses separated by at least 3 symptom‐free days were considered as separate illnesses.
Yeung 2011 cluster‐RCT Hong Kong	Pneumonia
Zomer 2015 cluster‐RCT Netherlands	Incidence of gastrointestinal and respiratory infections in children monitored by parents. The common cold was defined as a blocked or runny nose with at least 1 of the following symptoms: coughing, sneezing, fever, sore throat, or earache.
**Hand hygiene and masks (n = 6)**	
Aelami 2015 (conference abstract) RCT Saudi Arabia	Influenza‐like illness was defined as the presence of at least 2 of the following during their stay: fever, cough, and sore throat. Safety: no outcomes on harms planned or reported.
Aiello 2010 cluster‐RCT USA	Influenza‐like illness case definition (presence of cough and at least 1 constitutional symptom (fever/feverishness, chills, or body aches). Safety: no outcomes on harms planned or reported.
Cowling 2009 cluster‐RCT Hong Kong	2 clinical definitions of influenza. First definition was at least 2 of the following signs and symptoms: temperature 37.8 °C or greater, cough, headache, sore throat, and myalgia. The second was temperature 37.8 °C or greater plus cough or sore throat. Safety: no outcomes on harms planned or reported.
Larson 2010 cluster‐RCT USA	Study goals: rates of symptoms and secondary transmission of URIs, incidence of virologically‐confirmed influenza, knowledge of prevention and treatment strategies for influenza and URIs, and rates of influenza vaccination. Laboratory‐confirmed influenza: nasal swabs to test for influenza types A and B as well as other common respiratory viruses by rapid culture (R‐Mix, Diagnostic Hybrids, Inc., Athens, OH, USA). PCR and subtyping of the samples was done during the second half of the second year of the study. Influenza‐like illness: CDC definition of ILI from the Sentinel Physicians' Network was used to determine when masks should be worn: “temperature of ≥37.8°C and cough and/or sore throat in the absence of a known cause other than influenza". Episodes of URI = upper respiratory infection: not clear, no explicitly stated definition, reported that the most commonly reported URI symptoms are cough or rhinorrhoea. Safety: no outcomes on harms planned or reported.
Simmerman 2011 cluster‐RCT Thailand	Laboratory‐confirmed secondary influenza virus infections amongst household members described as the secondary attack rate. The secondary influenza virus infection was defined as a positive rRT‐PCR result on days 3 or 7 or a four‐fold rise in influenza HI antibody titres with the virus type and subtype matching the index case. Influenza‐like illness defined by WHO as fever plus cough or sore throat, based on self‐reported symptoms. Safety: no outcomes on harms planned or reported.
Suess 2012 cluster‐RCT Germany	Quantitative RT‐PCR for samples of nasal wash. Influenza virus infection as a laboratory‐confirmed influenza infection in a household member who developed fever (> 38.0 °C), cough, or sore throat during the observation period. Also secondary outcome measure of the occurrence of ILI as defined by WHO as fever plus cough or sore throat. Safety: the study reported that the majority of participants (107/172, 62%) did not report any problems with mask wearing. This proportion was significantly higher in the group of adults (71/100, 71%) compared to the group of children (36/72, 50%) (P = 0.005). The main problem stated by participants (adults and children) was "heat/humidity" (18/34, 53% of children; 10/29, 35% of adults) (P = 0.1), followed by "pain" and "shortness of breath" when wearing a face mask.
**Surface/object disinfection (with or without hand hygiene)(n = 8)**	
Ban 2015 cluster‐RCT China	Acute respiratory illness classified as the appearance of 2 or more of the following symptoms: fever, cough and expectoration, runny nose and nasal congestion.
Carabin 1999 cluster‐RCT Canada	The presence of nasal discharge (runny nose) accompanied by 1 or several of the following symptoms: fever, sneezing, cough, sore throat, ear pain, malaise, irritability. A URTI was defined as a cold for 2 consecutive days.
Chard 2019 cluster‐RCT Laos	Pupils were considered to have symptoms of respiratory infection if they reported cough, runny nose, stuffy nose, or sore throat.
Ibfelt 2015 cluster‐RCT Denmark	Laboratory confirmation of 16 respiratory viruses: influenza A; influenza B; coronavirus NL63229E, OC43 and HKU1; parainfluenza virus 1, 2, 3, and 4; rhinovirus; RSV A/B; adenovirus; enterovirus; parechovirus; and bocavirus using quantitative PCR
Kotch 1994 RCT USA	Respiratory symptoms include coughing, runny nose, wheezing or rattling in the chest, sore throat, or earache.
McConeghy 2017 RCT USA	Classified infections as lower respiratory tract infections (i.e. pneumonia, bronchitis, or chronic obstructive pulmonary disease exacerbation) or other.
Sandora 2008 cluster‐RCT USA	RI was defined as an acute illness that included > 1 of the following symptoms: runny nose, stuffy or blocked nose, cough, fever or chills, sore throat, or sneezing.
White 2001 DB‐RCT USA	RI was defined as: cough, sneezing, sinus trouble, bronchitis, fever alone, pink‐eye, headache, mononucleosis, and acute exacerbation of asthma.
**Other (miscellaneous) interventions (n = 5)**	
Fretheim 2022a pragmatic RCT Norway	Respiratory infection was defined as having 1 respiratory symptom (stuffed or runny nose, sore throat, cough, sneezing, heavy breathing) and fever, or 1 respiratory symptom and at least 2 more symptoms (body ache, muscular pain, fatigue, reduced appetite, stomach pain, headache, loss of smell.
Hartinger 2016 cluster‐RCT Peru	ARI was defined as a child presenting cough or difficulty breathing, or both. ALRI was defined as a child presenting cough or difficulty breathing, with a raised respiratory rate > 50 per minute in children aged 6 to 11 months and > 40 per minute in children aged > 12 months on 2 consecutive measurements. An episode was defined as beginning on the first day of cough or difficulty breathing and ending with the last day of the same combination, followed by at least 7 days without those symptoms.
Huda 2012 cluster‐RCT Bangladesh	Study authors classified acute respiratory illness as having cough and fever or difficulty breathing and fever within 48 h prior to interview.
Najnin 2019 cluster‐RCT Bangladesh	Classified participants as having respiratory illness if they reported having fever plus either cough or nasal congestion or fever plus breathing difficult.
Satomura 2005 RCT Japan	Upper respiratory tract infection defined as all of the following conditions: both nasal and pharyngeal symptoms; severity of at least 1 symptom increased by 2 grades or more; and worsening of a symptom of 1 increment or more for > 3 days. Because of the difference in the mode of transmission, study authors excluded influenza‐like diseases featured by moderate or severe fever; anti‐influenza vaccination in the preseason and arthralgia, and treated them separately. The incidence was determined by 1 study physician who was blinded to group assignment.
**Virucidal tissues (n = 2)**	
Farr 1988a cluster‐RCT USA trial 1 and trial 2	RI defined as: occurrence of at least 2 respiratory symptoms on the same day or the occurrence of a single respiratory symptom on 2 consecutive days (except for sneezing). The respiratory symptoms were as follows: sneezing, nasal congestion, nasal discharge, sore throat, scratchy throat, hoarseness, coughing, malaise, headache, feverishness, chilliness and myalgia.
Longini 1988 DB‐PC RCT USA	Respiratory illness defined as 1 or more of the following symptoms occurring during the course of acute episode: coryza, sore throat or hoarseness, earache, cough, pain on respiration, wheezy breathing or phlegm from the chest.

ALRI: acute lower respiratory infection ARIs: acute respiratory infections CDC: Centers for Disease Control and Prevention CI: confidence interval cluster‐RCT: cluster‐randomised controlled trial CRI: clinical respiratory illness DB‐PC: double‐blind, placebo‐controlled DB‐RCT: double‐blind randomised controlled trial DNA: deoxyribonucleic acid ELISA: enzyme‐linked immunosorbent assay GI: gastrointestinal h: hours HCW: healthcare workers HI: haemagglutinin hMPV: human metapneumo virus ICD‐9: International Classification of Disease, 9th Revision, Clinical Modification ICD‐10: International Classification of Disease, 10th Revision, Clinical Modification IgG: immunoglobulin G IgM: immunoglobulin M ILI: influenza‐like illness min: minutes MRSA: methicillin‐resistant Staphylococcus aureus NAT: nucleic acid testing NOS: not otherwise specified NTS: nasal and throat swab PCR: polymerase chain reaction PIV: parainfluenza virus POCT: point‐of‐care testing RCT: randomised controlled trial RI: respiratory infection RNA: ribonucleic acid RR: risk ratio rRT‐PCR: real‐time reverse transcriptase polymerase chain reaction RSV: respiratory syncytial virus RTI: respiratory tract infection RT‐PCR: reverse transcriptase polymerase chain reaction SAR: secondary attack ratios SD: standard deviation S/S: signs and symptoms URI: upper respiratory infection URTI: upper respiratory tract infection UTI: urinary tract infection WHO: World Health Organization

### Potential biases in the review process

The non‐drug (and often locally manufactured) nature of most of the interventions in this review, the lack of effective regulation in some settings, and the possible endless number of manufacturers make it difficult to gauge the existence of unpublished data. Non‐drug interventions typically have no or very loose regulation.

In this 2022 update, we again focused on RCTs and cluster‐RCTs, providing a higher level of evidence compared with the previous version of the review, which also meta‐analysed observational studies when appropriate (Jefferson 2011). However, many of the trials were small and hence underpowered, and at high or unclear risk of bias due to poor reporting of methods and lack of blinding. The populations, outcomes, comparators, and interventions tested were heterogeneous.

Due to the urgency of this update in the context of the COVID‐19 pandemic, we did not contact trial authors to request missing data. This means that we have not considered studies that included other non‐respiratory infections, and did not provide stratified data by type of infection.

### Agreements and disagreements with other studies or reviews

Several reviews of RCTs have found broadly similar results to this review for face masks. In a meta‐analysis comparing surgical masks with N95 respirators, Smith 2016 pooled three trials and found an estimate of effect suggesting no difference for laboratory‐confirmed respiratory infections (OR 0.89, 95% CI 0.64 to 1.24) or ILI (OR 0.51, 95% CI 0.19 to 1.41) (Loeb 2009; MacIntyre 2011; MacIntyre 2013). A similar meta‐analysis, Offeddu 2017, based on two trials concluded that masks (either N95/P2 respirators or medical/surgical masks) were effective against clinical respiratory infections (RR 0.59, 95% CI 0.46 to 0.77) and ILI (RR 0.34, 95% CI 0.14 to 0.82) (MacIntyre 2011; MacIntyre 2015). Pooling of two studies (MacIntyre 2011; MacIntyre 2013) also found an estimate of effect that favoured N95 respirators to medical/surgical masks for clinical respiratory infections (RR 0.47, 95% CI 0.36 to 0.62), but not for ILI, (RR 0.59, 95% CI 0.27 to 1.28) based on three studies (Loeb 2009: MacIntyre 2011; MacIntyre 2013). The outcome of clinical respiratory infection is considered to be the most subjective and least precise outcome.

A recent meta‐analysis included five trials comparing N95/P2 respirators with medical/surgical masks and found no difference between groups for either influenza (RR 1.09, 95% CI 0.92 to 1.28), or respiratory viral infections (RR 0.89, 95% CI 0.70 to 1.11) (Long 2020). By excluding Loeb 2009 (an open, non‐inferiority RCT that compared medical/surgical masks with N95 respirators in protecting HCWs against influenza), the authors reported a significant protective effect against viral infections (RR 0.61, 95% CI 0.39 to 0.98). The authors do not report a rationale for the exclusion in the sensitivity analysis, and do not report on exclusion of the studies with low weighting, which arguably would be more relevant in a sensitivity analysis. The two trials that make up 96% of the weighting demonstrated no significant differences in the outcome events (Loeb 2009; Radonovich 2019). A recent meta‐analysis of four RCTs adjusting for clustering, which compared N95 respirators with the use of medical/surgical  masks, found pooled estimates of effect that did not demonstrate any difference in any laboratory‐confirmed viral respiratory infection (OR 1.06, 95% CI 0.90 to 1.25), laboratory‐confirmed influenza (OR 0.94, 95% CI 0.73 to 1.20), or clinical respiratory illness (OR 1.49, 95% CI 0.98 to 2.28), with the evidence profile suggesting that there was greater imprecision and inconsistency in the outcome of clinical respiratory illness (Bartoszko 2020). Moreover, in another recent systematic review that assessed the effectiveness of personal protective and environmental measures in non‐healthcare settings (funded by the WHO), 10 RCTs reporting estimates of the effectiveness of face masks in reducing laboratory‐confirmed influenza virus infections in the community were identified (Xiao 2020). The evidence from these RCTs suggested that the use of face masks either by infected persons or by uninfected persons does not have a substantial effect on influenza transmission. 

The findings from several systematic reviews and meta‐analyses over the last decade have not demonstrated any difference in the clinical effectiveness of N95 respirators or equivalent compared to the use of surgical masks when used by HCWs in multiple healthcare settings for the prevention of respiratory virus infections, including influenza.

Reviews based on observational studies have usually found a stronger protective effect for face masks, but have important biases. The review by Chu 2020 did not consider RCTs of influenza transmission, but only the observational studies examining impact on SARS, MERS, or SARS‐CoV‐2. For N95 masks versus no mask in HCWs, there was a large protective effective with an OR of 0.04 (95% CI 0.004 to 0.30); for surgical masks versus no masks, there was an OR of 0.33 (0.17 to 0.61) overall, but four of these studies were in healthcare settings. Chu 2020 has been criticised for several reasons: use of an outdated 'Risk of bias' tool; inaccuracy of distance measures; and not adequately addressing multiple sources of bias, including recall and classification bias and in particular confounding. Confounding is very likely, as preventive behaviours such as mask use, social distancing, and hand hygiene are correlated behaviours, and hence any effect estimates are likely to be overly optimistic.

The two RCTs of medical/surgical masks during the SARS‐CoV‐2 pandemic found uncertain evidence of a small or no effect (Abaluck 2022; Bundgaard 2021). The study by Abaluck 2022 found a statistically significant benefit of masks versus no masks for COVID‐like‐illness, however, this study was rated at high risk of bias for five of the six domains due to issues including baseline imbalance, subjective outcome assessment and incomplete follow‐up across the groups. Despite this study contributing 45% of the weight towards the meta‐analysis of influenza/COVID‐like‐illness for masks versus no masks, the updated conclusions from the analysis strengthened around little or no effect of mask use. 

Also based on observational studies, Jefferson 2011 found a protective effect of wearing surgical masks with hygienic measures compared to not wearing masks in the SARS 2003 outbreak (OR 0.32, 95% CI 0.26 to 0.39). However, the evidence was based on case‐control studies carried out during the outbreak. There was some additional but very limited supportive evidence from the cohort studies in Jefferson 2011. 

Although the use of eye protection and physical distancing measures are widely believed to be effective in reducing transmission of respiratory viruses and mitigating the impact of an influenza pandemic, we found only one trial investigating the role of self‐quarantine in reducing the incidence of H1N1 influenza events in the workplace, and no trials examining the effect of eye protection. The evidence for these measures was derived largely from observational studies and simulation studies, and the overall certainty of supporting evidence is relatively low. The finding of limited evidence evaluating these interventions was also consistent with a recent review funded by the WHO for the preparation of its guidelines on the use of non‐pharmaceutical interventions for pandemic influenza in non‐medical settings (Fong 2020).

There are several previous systematic reviews on hand hygiene and respiratory infections. Five of them reviewed the evidence in a community setting (Moncion 2019; Rabie 2006; Saunders‐Hastings 2017; Warren‐Gash 2013: Wong 2014), and three focused on children (Mbakaya 2017; Willmott 2016; Zivich 2018). The earliest review in 2006 included eight studies, three of which were RCTs (Rabie 2006). The pooled estimate of seven studies was described as “indicative” of the effect of hand hygiene, but the studies were of poor quality. The Warren‐Gash 2013 review included 16 studies (10 of which were RCTs) and reported mixed and inconclusive results. A 2014 review identified 10 RCTs and reported that the combination of hand hygiene with face masks in high‐income countries (five trials) significantly reduced the incidence of laboratory‐confirmed influenza and ILI, whilst hand hygiene alone did not (Wong 2014). This significant reduction in laboratory‐confirmed influenza and ILI for hand hygiene and face masks may have been based on the raw numbers without adjusting for any clustering effects in the included cluster trials, which produced inappropriately narrow CIs, and possibly biased treatment effect estimates. Moreover, trials from the low‐income countries were not included in the review, and this significant effect was not demonstrated when all the trials identified in the review were combined. The Saunders‐Hastings 2017 review of studies evaluating the effectiveness of personal protective measures in interrupting pandemic influenza transmission only identified two RCTs (Azor‐Martinez 2014; Suess 2012), which reported a significant effect of hand hygiene. The Moncion 2019 review identified seven RCTs of hand hygiene compared to control, with mixed results for preventing the transmission of laboratory‐confirmed or possible influenza. Systematic reviews of RCTs of hand hygiene interventions amongst children, Mbakaya 2017 and Willmott 2016, or at a non‐clinical workplace, Zivich 2018, identified heterogeneous trials with quality problems including small numbers of clusters and participants, inadequate randomisation, and self‐reported outcomes. Evidence of impact on respiratory infections was equivocal. 

A rapid search for other systematic reviews of RCTs was conducted in September 2022, and none of high quality were found.

## Authors' conclusions

**Implications for practice**

The evidence summarised in this review on the use of masks is largely based on studies conducted during traditional peak respiratory virus infection seasons up until 2016. Two relevant randomised trials conducted during the COVID‐19 pandemic have been published, but their addition had minimal impact on the overall pooled estimate of effect. The observed lack of effect of mask wearing in interrupting the spread of influenza‐like illness (ILI) or influenza/COVID‐19 in our review has many potential reasons, including: poor study design; insufficiently powered studies arising from low viral circulation in some studies; lower adherence with mask wearing, especially amongst children; quality of the masks used; self‐contamination of the mask by hands; lack of protection from eye exposure from respiratory droplets (allowing a route of entry of respiratory viruses into the nose via the lacrimal duct); saturation of masks with saliva from extended use (promoting virus survival in proteinaceous material); and possible risk compensation behaviour leading to an exaggerated sense of security (Ammann 2022; Brosseau 2020; Byambasuren 2021; Canini 2010; Cassell 2006; Coroiu 2021; MacIntyre 2015; Rengasamy 2010; Zamora 2006).

Our findings show that hand hygiene has a modest effect as a physical intervention to interrupt the spread of respiratory viruses, but several questions remain. First, the high heterogeneity between studies may suggest that there are differences in the effect of different interventions. The poor reporting limited our ability to extract the information needed to assess any 'dose response' relationship, and there are few head‐to‐head trials comparing hand hygiene materials (such as alcohol‐based sanitiser or soap and water). Second, the sustainability of hand hygiene is unclear where participants in some studies achieved 5 to 10 hand‐washings per day, but adherence may have diminished with time as motivation decreased, or due to adverse effects from frequent hand‐washing. Third, there is little evidence about the effectiveness of combinations of hand hygiene with other interventions, and how those are best introduced and sustained. Finally, some interventions were intensively implemented within small organisations, and involved education or training as a component, and the ability to scale these up to broader interventions is unclear. 

Our findings with respect to hand hygiene should be considered generally relevant to all viral respiratory infections, given the diverse populations where transmission of viral respiratory infections occurs. The participants were adults, children and families, and multiple congregation settings including schools, childcare centres, homes, and offices. Most respiratory viruses, including the pandemic SARS‐CoV‐2, are considered to be predominantly spread via respiratory particles of varying size or contact routes, or both (WHO 2020c). Data from studies of SARS‐CoV‐2 contamination of the environment based on the presence of viral ribonucleic acid and infectious virus suggest significant fomite contamination (Lin 2022; Onakpoya 2022b; Ong 2020; Wu 2020). Hand hygiene would be expected to be beneficial in reducing the spread of SARS‐CoV‐2 similar to other beta coronaviruses (SARS‐CoV‐1, Middle East respiratory syndrome (MERS), and human coronaviruses), which are very susceptible to the concentrations of alcohol commonly found in most hand‐sanitiser preparations (Rabenau 2005; WHO 2020c). Support for this effect is the finding that poor hand hygiene, despite the use of full personal protective equipment (PPE), was independently associated with an increased risk of SARS‐CoV‐2 transmission to healthcare workers in a retrospective cohort study in Wuhan, China in both a high‐risk and low‐risk clinical unit for patients infected with COVID‐19 (Ran 2020). The practice of hand hygiene appears to have a consistent effect in all settings, and should be an essential component of other interventions.

The highest‐quality cluster‐RCTs indicate that the most effect on preventing respiratory virus spread from hygienic measures occurs in younger children. This may be because younger children are least capable of hygienic behaviour themselves (Roberts 2000), and have longer‐lived infections and greater social contact, thereby acting as portals of infection into the household (Monto 1969). Additional benefit from reduced transmission from them to other members of the household is broadly supported by the results of other study designs where the potential for confounding is greater.

Routine long‐term implementation of some of the interventions covered in this review may be problematic, particularly maintaining strict hygiene and barrier routines for long periods of time. This would probably only be feasible in highly motivated environments, such as hospitals. Many of the trial authors commented on the major logistical burdens that barrier routines imposed at the community level. However, the threat of a looming epidemic may provide stimulus for their inception.

**Implications for research**

Public health measures and physical interventions can be highly effective to interrupt the spread of respiratory viral infections, especially when they are part of a structured and co‐ordinated programme that includes instruction and education, and when they are delivered together and with high adherence. Our review has provided important insights into research gaps that need to be addressed with respect to these physical interventions and their implementation and have been brought into a sharper focus as a result of the COVID‐19 pandemic. The 2014 WHO document 'Infection prevention and control of epidemic ‐ and pandemic‐prone acute respiratory infections in health care' identified several research gaps as part of their GRADE assessment of their infection prevention and control recommendations, which remain very relevant (WHO 2014). Research gaps identified during the course of our review and the WHO 2014 document may be considered from the perspective of both general and specific themes.

A general theme identified was the need to provide outcomes with explicitly defined clinical criteria for acute respiratory infections (ARIs) and discrete laboratory‐confirmed outcomes of viral ARIs using molecular diagnostic tools which are now widely available. Our review found large disparities between studies with respect to the clinical outcome events, which were imprecisely defined in several studies, and there were differences in the extent to which laboratory‐confirmed viruses were included in the studies that assessed them. Another general theme identified was the lack of consideration of sociocultural factors that might affect adherence with the interventions, especially those employed in the community setting. A prime example of this latter point was illustrated by the observations of the use of masks versus mask mandates during the COVID‐19 pandemic. In addition, the cost and resource implications of the physical interventions employed in different settings would have important relevance for low‐ to middle‐income countries. Resources have been a major issue with the COVID‐19 pandemic, with global shortages of several components of PPE. Several specific research gaps related to physical interventions were identified within the WHO 2014 document and are congruent with many of the findings of this 2022 update, including the following: transmission dynamics of respiratory viruses from patients to healthcare workers during aerosol‐generating procedures; a continued lack of precision with regards to defining aerosol‐generating procedures; the safety of cohorting of patients with the same suspected but unconfirmed diagnosis in a common unit or ward with patients infected with the same known pathogen in healthcare settings; the optimal duration of the use of physical interruptions to prevent spread of ARI viruses; use of spatial separation or physical distancing (in healthcare and community settings, respectively) alone versus spatial separation or physical distancing with the use of other added physical interventions coupled with examining discrete distance parameters (e.g. one metre, two metres, or > two metres); the effectiveness of respiratory etiquette (i.e. coughing/sneezing into tissues or a sleeved bent elbow); the effectiveness of triage and early identification of infected individuals with an ARI in both hospital and community settings; the utility of entrance screening to healthcare facilities; use of frequent disinfection techniques appropriate to the setting (high‐touch surfaces in the environment, gargling with oral disinfectants, and virucidal tissues or clothing) alone or in combination with facial masks and hand hygiene; the use of visors, goggles or other eyewear; the use of ultraviolet light germicidal irradiation for disinfection of air in healthcare and selected community settings; the use of air scrubbers and /or high‐efficiency particulate absorbing filters and the use of widespread adherence with effective vaccination strategies.

There is a clear requirement to conduct large, pragmatic trials to evaluate the best combinations in the community and in healthcare settings with multiple respiratory viruses and in different sociocultural settings. Randomised controlled trials (RCTs) with a pragmatic design, similar to the Luby 2005 trial or the Bundgaard 2020 trial, should be conducted whenever possible. Similar to what has been observed in pharmaceutical interventions where multiple RCTs were rapidly and successfully completed during the COVID‐19 pandemic, proving they can be accomplished, there should be a deliberate emphasis and directed funding opportunities provided to conduct well‐designed RCTs to address the effectiveness of many of the physical interventions in multiple settings and populations, especially in those most at risk, and in very specific well‐defined populations with monitoring of the adherence to the interventions. 

Several specific research gaps deserve expedited attention and may be highlighted within the context of the COVID‐19 pandemic. The use of face masks in the community setting represents one of the most pressing needs to address, given the polarised opinions around the world, and the increasing concerns over widespread microplastic pollution from the discarding of masks (Shen 2021). Both broad‐based ecological studies, adjusting for confounding and high quality RCTs, may be necessary to determine if there is an independent contribution to their use as a physical intervention, and how they may best be deployed to optimise their contribution. The type of fabric and weave used in the face mask is an equally pressing concern, given that surgical masks with their cotton‐polypropylene fabric appear to be effective in the healthcare setting, but there are questions about the effectiveness of simple cotton masks. In addition, any masking intervention studies should focus on measuring not only benefits but also adherence, harms, and risk compensation if the latter may lead to a lower protective effect. In addition, although the use of medical/surgical masks versus N95 respirators demonstrates no differences in clinical effectiveness to date, their use needs to be further studied within the context of a well‐designed RCT in the setting of COVID‐19, and with concomitant measurement of harms, which to date have been poorly studied. The recently published Loeb RCT conducted over a prolonged course in the current pandemic has provided the only evidence to date in this area (Loeb 2022).

Physical distancing represents another major research gap which needs to be addressed expediently, especially within the context of the COVID‐19 pandemic setting as well as in future epidemic settings. The use of quarantine and screening at entry ports needs to be investigated in well‐designed, high‐quality RCTs given the controversies related to airports and travel restrictions which emerged during the COVID‐19 pandemic. We found only one RCT investigating quarantine, and no trials of screening at entry ports or physical distancing. Given that these and other physical interventions are some of the primary strategies applied globally in the face of the COVID‐19 pandemic, future trials of high quality should be a major global priority to be  conducted within the context of this pandemic, as well as in future epidemics with other respiratory viruses of less virulence.

The variable quality and small scale of some studies is known from descriptive studies (Aiello 2002; Fung 2006; WHO 2006b), and systematic reviews of selected interventions (Meadows 2004). In summary, more high‐quality RCTs are needed to evaluate the most effective strategies to implement successful physical interventions in practice, both on a small scale and at a population level. It is very unfortunate that more rigorous planning, effort and funding was not provided during the current COVID‐19 pandemic towards high‐quality RCTs of the basic public health measures. Finally, we emphasise that more attention should be paid to describing and quantifying the harms of the interventions assessed in this review, and their relationship with adherence.

## What's new

**Table:** 

**Date**	**Event**	**Description**
4 April 2023	Amended	John Conly's declaration of interest statement has been clarified in response to a feedback comment.

## History

Protocol first published: Issue 4, 2006 Review first published: Issue 4, 2007

**Table:** 

**Date**	**Event**	**Description**
27 January 2023	New citation required but conclusions have not changed	Our conclusions remain unchanged.
27 January 2023	New search has been performed	Searches updated. We included 11 new trials (Abaluck 2022; Alfelali 2020; Almanza‐Reyes 2021; Ashraf 2020; Bundgaard 2021; Fretheim 2022a; Gutiérrez‐García 2022; Helsingen 2021; Swarthout 2020; Teesing 2021; Young 2021), and excluded 20 new trials (Ahmadian 2022; Chen 2022; Costa 2021; Cyril Vitug 2021; Dalakoti 2022; Egger 2022; Ferrer 2021; Gharebaghi 2020; Giuliano 2021; Karakaya 2021; Kawyannejad 2020; Lim 2022; Malaczek 2022; Meister 2022; Mo 2022; Montero‐Vilchez 2022; Munoz‐Basagoiti 2022; Sanchez Barrueco 2022; Seneviratne 2021; Sevinc Gul 2022). We identified two new ongoing trials (Brass 2021; NCT04471766), and five trials awaiting classification (Contreras 2022; Croke 2022; Delaguerre 2022; Loeb 2022; Varela 2022).
1 April 2020	New search has been performed	Searches updated. In this 2020 update we only searched for RCTs and cluster‐RCTs. We included 44 new trials (Aelami 2015; Aiello 2012; Alzaher 2018; Arbogast 2016; Azor‐Martinez 2016; Azor‐Martinez 2018; Ban 2015; Barasheed 2014; Biswas 2019; Canini 2010; Chard 2019; Correa 2012; DiVita 2011; Feldman 2016; Goodall 2014; Hartinger 2016; Hubner 2010; Huda 2012; Ibfelt 2015; Ide 2014; Ide 2016; Little 2015; MacIntyre 2011; MacIntyre 2013; MacIntyre 2015; MacIntyre 2016; McConeghy 2017; Millar 2016; Miyaki 2011; Najnin 2019; Nicholson 2014; Pandejpong 2012; Priest 2014; Radonovich 2019; Ram 2015; Savolainen‐Kopra 2012; Simmerman 2011; Stebbins 2011; Suess 2012; Talaat 2011; Temime 2018; Turner 2012; Yeung 2011; Zomer 2015). We excluded 12 new trials (Azor‐Martinez 2014; Bowen 2007; Chami 2012; Denbak 2018; Lennell 2008; Nandrup‐Bus 2009; Patel 2012; Rosen 2006; Slayton 2016; Stedman‐Smith 2015; Uhari 1999; Vessey 2007). We identified 5 new ongoing trials (NCT03454009; NCT04267952; NCT04296643; NCT04337541; Wang 2015) one of which – NCT04337541 ‐ published as this review was going to press. We focused on RCTs and cluster‐RCTs only and removed observational studies from this update.
1 April 2020	New citation required and conclusions have changed	There is now sufficient randomised controlled trial (RCT) evidence to show that hand hygiene is likely to provide a modest benefit. Uncertainty remains for the other interventions. Further RCT evidence is needed.
22 October 2010	New citation required but conclusions have not changed	We updated the review again at the behest of the World Health Organization (WHO). External sources of support amended. External support from the WHO. The WHO interim guidelines document on 'Infection Prevention and Control of Epidemic and Pandemic Prone Acute Respiratory Diseases in Health Care' was published in 2007 to provide infection control guidance to help prevent the transmission of acute respiratory diseases in health care. The update of these guidelines will be evidence‐based, and an update of this review was requested to assist in informing the evidence base for the revision of the WHO guidelines. Dr John Conly, Dr Mark Jones, and Sarah Thorning joined the review team.
22 October 2010	New search has been performed	Searches conducted. We included 7 new trials: 4 randomised controlled trials and 3 non‐randomised comparative studies. We excluded 36 new trials.
7 May 2009	New search has been performed	For the 2009 update, we included 3 cluster‐randomised controlled trials, Cowling 2009; MacIntyre 2009; Sandora 2008, and 1 individual randomised controlled trial (Satomura 2005, with its linked publication Kitamura 2007). We also included 1 retrospective cohort study (Foo 2006), 1 case‐control study (Yu 2007), and 2 prospective cohort studies (Wang 2007; Broderick 2008). The content and conclusions of the 2007 review changed little, but the additional 8 studies add more information and certainty. Our meta‐analysis remains unchanged as there were no new studies for pooling.
30 April 2009	New citation required but conclusions have not changed	New author joined the review team.
8 July 2008	Amended	Converted to new review format.
20 August 2007	Amended	Review first published Issue 4, 2007.

## Notes

In Issue 1, 2010, the title of the review was changed from 'Interventions for the interruption or reduction of the spread of respiratory viruses' to 'Physical interventions to interrupt or reduce the spread of respiratory viruses'.

The original review was subsequently published as Jefferson T, Foxlee R, Del Mar C, Dooley L, Ferroni E, Hewak B, Prabhala A, Nair S, Rivetti A. Physical interventions to interrupt or reduce the spread of respiratory viruses: systematic review. BMJ 2008;336:77‐80 and Jefferson T, Del Mar C, Dooley L, Ferroni E, Al‐Ansary LA, Bawazeer GA, van Driel ML, Foxlee R, Rivetti A. Physical interventions to interrupt or reduce the spread of respiratory viruses: systematic review. BMJ 2009;339:b3675. DOI: 10.1136/bmj.b3675.

## Appendices

### Appendix 1. Cochrane Central Register of Controlled Trials (CENTRAL) search string

([mh "Influenza, Human"] OR [mh "Influenzavirus A"] OR [mh "Influenzavirus B"] OR [mh "Influenzavirus C"] OR Influenza:ti,ab OR [mh "Respiratory Tract Diseases"] OR Influenzas:ti,ab OR “Influenza‐like”:ti,ab OR ILI:ti,ab OR Flu:ti,ab OR Flus:ti,ab OR [mh ^"Common Cold"] OR "common cold":ti,ab OR colds:ti,ab OR coryza:ti,ab OR [mh coronavirus] OR [mh "sars virus"] OR coronavirus:ti,ab OR Coronaviruses:ti,ab OR [mh "coronavirus infections"] OR [mh "severe acute respiratory syndrome"] OR "severe acute respiratory syndrome":ti,ab OR "severe acute respiratory syndromes":ti,ab OR sars:ti,ab OR [mh "respiratory syncytial viruses"] OR [mh "respiratory syncytial virus, human"] OR [mh "Respiratory Syncytial Virus Infections"] OR "respiratory syncytial virus":ti,ab OR "respiratory syncytial viruses":ti,ab OR rsv:ti,ab OR parainfluenza:ti,ab OR “Respiratory illness”:ti,ab OR ((Transmission) AND (Coughing OR Sneezing)) OR ((respiratory:ti,ab AND Tract) AND (infection:ti,ab OR Infections:ti,ab OR illness:ti,ab))) AND ([mh "Hand Hygiene"] OR handwashing:ti,ab OR “hand‐washing”:ti,ab OR ((Hand:ti,ab OR Alcohol:ti,ab) AND (wash:ti,ab OR Washing:ti,ab OR Cleansing:ti,ab OR Rinses:ti,ab OR hygiene:ti,ab OR rub:ti,ab OR Rubbing:ti,ab OR sanitizer:ti,ab OR sanitiser:ti,ab OR cleanser:ti,ab OR disinfected:ti,ab OR Disinfectant:ti,ab OR Disinfect:ti,ab OR antiseptic:ti,ab OR virucid:ti,ab)) OR [mh "gloves, protective"] OR Glove:ti,ab OR Gloves:ti,ab OR [mh Masks] OR [mh "respiratory protective devices"] OR facemask:ti,ab OR Facemasks:ti,ab OR mask:ti,ab OR Masks:ti,ab OR respirator:ti,ab OR respirators:ti,ab OR [mh ^"Protective Clothing"] OR [mh "Protective Devices"] OR "patient isolation":ti,ab OR ((school:ti,ab OR Schools:ti,ab) AND (Closure:ti,ab OR Closures:ti,ab OR Closed:ti,ab)) OR [mh Quarantine] OR quarantine:ti,ab OR "Hygiene intervention":ti,ab OR [mh Mouthwashes] OR gargling:ti,ab OR "nasal tissues":ti,ab OR [mh "Eye Protective Devices"] OR Glasses:ti,ab OR Goggle:ti,ab OR "Eye protection":ti,ab OR Faceshield:ti,ab OR Faceshields:ti,ab OR Goggles:ti,ab OR "Face shield":ti,ab OR "Face shields":ti,ab OR Visors:ti,ab) AND ([mh "Communicable Disease Control"] OR [mh "Disease Outbreaks"] OR [mh "Disease Transmission, Infectious"] OR [mh "Infection Control"] OR "Communicable Disease Control":ti,ab OR "Secondary transmission":ti,ab OR ((Reduced:ti,ab OR Reduce:ti,ab OR Reduction:ti,ab OR Reducing:ti,ab OR Lower:ti,ab) AND (Incidence:ti,ab OR Occurrence:ti,ab OR Transmission:ti,ab OR Secondary:ti,ab))

### Appendix 2. PubMed search string

("Influenza, Human"[Mesh] OR "Influenzavirus A"[Mesh] OR "Influenzavirus B"[Mesh] OR "Influenzavirus C"[Mesh] OR Influenza[tiab] OR "Respiratory Tract Diseases"[Mesh] OR "Bacterial Infections/transmission"[Mesh] OR Influenzas[tiab] OR “Influenza‐like”[tiab] OR ILI[tiab] OR Flu[tiab] OR Flus[tiab] OR "Common Cold"[Mesh:NoExp] OR "common cold"[tiab] OR colds[tiab] OR coryza[tiab] OR coronavirus[Mesh] OR "sars virus"[Mesh] OR coronavirus[tiab] OR Coronaviruses[tiab] OR "coronavirus infections"[Mesh] OR "severe acute respiratory syndrome"[Mesh] OR "severe acute respiratory syndrome"[tiab] OR "severe acute respiratory syndromes"[tiab] OR sars[tiab] OR "respiratory syncytial viruses"[Mesh] OR "respiratory syncytial virus, human"[Mesh] OR "Respiratory Syncytial Virus Infections"[Mesh] OR "respiratory syncytial virus"[tiab] OR "respiratory syncytial viruses"[tiab] OR rsv[tiab] OR parainfluenza[tiab] OR “Respiratory illness”[tiab] OR ((Transmission[tiab]) AND (Coughing[tiab] OR Sneezing[tiab])) OR ((respiratory[tiab] AND Tract[tiab]) AND (infection[tiab] OR Infections[tiab] OR illness[tiab]))) AND ("Hand Hygiene"[Mesh] OR handwashing[tiab] OR hand‐washing[tiab] OR ((Hand[tiab] OR Alcohol[tiab]) AND (wash[tiab] OR Washing[tiab] OR Cleansing[tiab] OR Rinses[tiab] OR hygiene[tiab] OR rub[tiab] OR Rubbing[tiab] OR sanitizer[tiab] OR sanitiser[tiab] OR cleanser[tiab] OR disinfected[tiab] OR Disinfectant[tiab] OR Disinfect[tiab] OR antiseptic[tiab] OR virucid[tiab])) OR "gloves, protective"[Mesh] OR Glove[tiab] OR Gloves[tiab] OR Masks[Mesh] OR "respiratory protective devices"[Mesh] OR facemask[tiab] OR Facemasks[tiab] OR mask[tiab] OR Masks[tiab] OR respirator[tiab] OR respirators[tiab] OR "Protective Clothing"[Mesh:NoExp] OR "Protective Devices"[Mesh] OR "patient isolation"[tiab] OR ((school[tiab] OR Schools[tiab]) AND (Closure[tiab] OR Closures[tiab] OR Closed[tiab])) OR Quarantine[Mesh] OR quarantine[tiab] OR “Hygiene intervention”[tiab] OR "Mouthwashes"[Mesh] OR gargling[tiab] OR “nasal tissues”[tiab] OR "Eye Protective Devices"[Mesh] OR Glasses[tiab] OR Goggle[tiab] OR “Eye protection”[tiab] OR Faceshield[tiab] OR Faceshields[tiab] OR Goggles[tiab] OR “Face shield”[tiab] OR “Face shields”[tiab] OR Visors[tiab]) AND ("Communicable Disease Control"[Mesh] OR "Disease Outbreaks"[Mesh] OR "Disease Transmission, Infectious"[Mesh] OR "Infection Control"[Mesh] OR Transmission[sh] OR “Prevention and control”[sh] OR "Communicable Disease Control"[tiab] OR “Secondary transmission”[tiab] OR ((Reduced[tiab] OR Reduce[tiab] OR Reduction[tiab] OR Reducing[tiab] OR Lower[tiab]) AND (Incidence[tiab] OR Occurrence[tiab] OR Transmission[tiab] OR Secondary[tiab]))) AND (Randomized controlled trial[pt] OR controlled clinical trial[pt] OR randomized[tiab] OR randomised[tiab] OR placebo[tiab] OR "drug therapy"[sh] OR randomly[tiab] OR trial[tiab] OR groups[tiab]) NOT  (Animals[Mesh] not (Animals[Mesh] and Humans[Mesh])) NOT (“Case Reports”[pt] OR Editorial[pt] OR Letter[pt] OR Meta‐Analysis[pt] OR “Observational Study”[pt] OR “Systematic Review”[pt] OR “Case Report”[ti] OR “Case series”[ti] OR Meta‐Analysis[ti] OR “Meta Analysis”[ti] OR “Systematic Review”[ti])

### Appendix 3. Embase (Elsevier) search string

('influenza'/exp OR Influenza:ti,ab OR 'Respiratory Tract Disease'/exp OR Influenzas:ti,ab OR Influenza‐like:ti,ab OR ILI:ti,ab OR Flu:ti,ab OR Flus:ti,ab OR 'Common Cold'/de OR "common cold":ti,ab OR colds:ti,ab OR coryza:ti,ab OR 'coronavirus'/exp OR 'SARS coronavirus'/exp OR coronavirus:ti,ab OR Coronaviruses:ti,ab OR 'coronavirus infection'/exp OR 'severe acute respiratory syndrome'/exp OR "severe acute respiratory syndrome":ti,ab OR "severe acute respiratory syndromes":ti,ab OR sars:ti,ab OR 'Pneumovirus'/exp OR 'Human respiratory syncytial virus'/exp OR  "respiratory syncytial virus":ti,ab OR "respiratory syncytial viruses":ti,ab OR rsv:ti,ab OR parainfluenza:ti,ab OR “Respiratory illness”:ti,ab OR ((Transmission) AND (Coughing OR Sneezing)) OR ((respiratory:ti,ab AND Tract) AND (infection:ti,ab OR Infections:ti,ab OR illness:ti,ab))) AND ('hand washing'/exp OR handwashing:ti,ab OR hand‐washing:ti,ab OR ((Hand:ti,ab OR Alcohol:ti,ab) AND (wash:ti,ab OR Washing:ti,ab OR Cleansing:ti,ab OR Rinses:ti,ab OR hygiene:ti,ab OR rub:ti,ab OR Rubbing:ti,ab OR sanitizer:ti,ab OR sanitiser:ti,ab OR cleanser:ti,ab OR disinfected:ti,ab OR Disinfectant:ti,ab OR Disinfect:ti,ab OR antiseptic:ti,ab OR virucid:ti,ab)) OR 'protective glove'/exp OR Glove:ti,ab OR Gloves:ti,ab OR 'mask'/exp OR 'gas mask'/exp OR facemask:ti,ab OR Facemasks:ti,ab OR mask:ti,ab OR Masks:ti,ab OR respirator:ti,ab OR respirators:ti,ab OR 'protective clothing'/de OR 'protective equipment'/exp OR "patient isolation":ti,ab OR ((school:ti,ab OR Schools:ti,ab) AND (Closure:ti,ab OR Closures:ti,ab OR Closed:ti,ab)) OR 'Quarantine'/exp OR quarantine:ti,ab OR "Hygiene intervention":ti,ab OR 'mouthwash'/exp OR gargling:ti,ab OR "nasal tissues":ti,ab OR ‘eye protective device'/exp OR Glasses:ti,ab OR Goggle:ti,ab OR "Eye protection":ti,ab OR Faceshield:ti,ab OR Faceshields:ti,ab OR Goggles:ti,ab OR "Face shield":ti,ab OR "Face shields":ti,ab OR Visors:ti,ab) AND ('Communicable Disease Control'/exp OR 'epidemic'/exp OR 'disease transmission'/exp OR 'Infection Control'/exp OR "Communicable Disease Control":ti,ab OR "Secondary transmission":ti,ab OR ((Reduced:ti,ab OR Reduce:ti,ab OR Reduction:ti,ab OR Reducing:ti,ab OR Lower:ti,ab) AND (Incidence:ti,ab OR Occurrence:ti,ab OR Transmission:ti,ab OR Secondary:ti,ab))) AND (random* OR factorial OR crossover OR placebo OR blind OR blinded OR assign OR assigned OR allocate OR allocated OR 'crossover procedure'/exp OR 'double‐blind procedure'/exp OR 'randomized controlled trial'/exp OR 'single‐blind procedure'/exp NOT ('animal'/exp NOT ('animal'/exp AND 'human'/exp)))

### Appendix 4. CINAHL (EBSCO) search string

((MH "Influenza, Human+") OR (MH "Orthomyxoviridae+") OR TI Influenza OR AB Influenza OR (MH "Respiratory Tract Diseases+") OR TI Influenzas OR AB Influenzas OR TI Influenza‐like OR AB Influenza‐like OR TI ILI OR AB ILI OR TI Flu OR AB Flu OR TI Flus OR AB Flus OR (MH "Common Cold+") OR TI "common cold" OR AB "common cold" OR TI colds OR AB colds OR TI coryza OR AB coryza OR (MH "coronavirus+") OR (MH "sars virus+") OR TI coronavirus OR AB coronavirus OR TI Coronaviruses OR AB Coronaviruses OR (MH "coronavirus infections+") OR (MH "severe acute respiratory syndrome+") OR TI "severe acute respiratory syndrome" OR AB "severe acute respiratory syndrome" OR TI "severe acute respiratory syndromes" OR AB "severe acute respiratory syndromes" OR TI sars OR AB sars OR (MH "respiratory syncytial viruses+") OR TI "respiratory syncytial virus" OR AB "respiratory syncytial virus" OR TI "respiratory syncytial viruses" OR AB "respiratory syncytial viruses" OR TI rsv OR AB rsv OR TI parainfluenza OR AB parainfluenza OR TI “Respiratory illness” OR AB “Respiratory illness” OR ((Transmission) AND (Coughing OR Sneezing)) OR ((TI respiratory OR AB respiratory AND Tract) AND (TI infection OR AB infection OR TI Infections OR AB Infections OR TI illness OR AB illness))) AND ((MH "Handwashing+") OR TI handwashing OR AB handwashing OR TI hand‐washing OR AB hand‐washing OR ((TI Hand OR AB Hand OR TI Alcohol OR AB Alcohol) AND (TI wash OR AB wash OR TI Washing OR AB Washing OR TI Cleansing OR AB Cleansing OR TI Rinses OR AB Rinses OR TI hygiene OR AB hygiene OR TI rub OR AB rub OR TI Rubbing OR AB Rubbing OR TI sanitizer OR AB sanitiser OR TI sanitizer OR AB sanitiser OR TI cleanser OR AB cleanser OR TI disinfected OR AB disinfected OR TI Disinfectant OR AB Disinfectant OR TI Disinfect OR AB Disinfect OR TI antiseptic OR AB antiseptic OR TI virucid OR AB virucid)) OR (MH "gloves+") OR TI Glove OR AB Glove OR Gloves OR (MH "Masks+") OR (MH "respiratory protective devices+") OR TI facemask OR AB facemask OR TI Facemasks OR AB Facemasks OR TI mask OR AB mask OR TI Masks OR AB Masks OR TI respirator OR AB respirator OR TI respirators OR AB respirators OR (MH "Protective Clothing") OR (MH "Protective Devices+") OR TI "patient isolation" OR AB "patient isolation" OR ((TI school OR AB school OR TI Schools OR AB Schools) AND (TI Closure OR AB Closure OR TI Closures OR AB Closures OR TI Closed OR AB Closed)) OR (MH "Quarantine+") OR TI quarantine OR AB quarantine OR TI "Hygiene intervention" OR AB "Hygiene intervention" OR (MH "Mouthwashes+") OR TI gargling OR AB gargling OR TI "nasal tissues" OR AB "nasal tissues" OR (MH "Eye Protective Devices+") OR TI Glasses OR AB Glasses OR TI Goggle OR AB Goggle OR TI "Eye protection" OR AB "Eye protection" OR TI Faceshield OR AB Faceshield OR TI Faceshields OR AB Faceshields OR TI Goggles OR AB Goggles OR TI "Face shield" OR AB "Face shield" OR TI "Face shields" OR AB "Face shields" OR TI Visors OR AB Visors) AND ((MH "Infection Control+") OR (MH "Disease Outbreaks+") OR (MH "Infection Control+") OR TI "Communicable Disease Control" OR AB "Communicable Disease Control" OR TI "Secondary transmission" OR AB "Secondary transmission" OR ((TI Reduced OR AB Reduced OR TI Reduce OR AB Reduce OR TI Reduction OR AB Reduction OR TI Reducing OR AB Reducing OR TI Lower OR AB Lower) AND (TI Incidence OR AB Incidence OR TI Occurrence OR AB Occurrence OR TI Transmission OR AB Transmission OR TI Secondary OR AB Secondary))) AND ((MH "Clinical Trials+") OR (MH "Quantitative Studies") OR TI placebo* OR AB placebo* OR (MH "Placebos") OR (MH "Random Assignment") OR TI random* OR AB random* OR TI ((singl* or doubl* or tripl* or trebl*) W1 (blind* or mask*)) OR AB ((singl* or doubl* or tripl* or trebl*) W1 (blind* or mask*)) OR TI clinic* trial* OR AB clinic* trial* OR PT clinical trial)

### Appendix 5. Previous search strategies (pre‐2010)

Details of the 2010 update and the search strategy used in the original review and the 2009 search strategy updates for MEDLINE, CENTRAL, EMBASE and CINAHL

In the 2010 update we searched, as we have done previously, the Cochrane Central Register of Controlled Trials (CENTRAL) 2010, Issue 3, which includes the Acute Respiratory Infections Group's Specialised Register, MEDLINE (April 2009 to October week 2, 2010), EMBASE (April 2009 to October 2010) and CINAHL (January 2009 to October 2010). Details of previous searches are in Appendix 1. In addition, to include more of the literature of low‐income countries in this update, we ran searches in LILACS (2008 to October 2010), Indian MEDLARS (2008 to October 2010) and IMSEAR (2008 to October 2010).

We used the following search strategy (updated to include new and emerging respiratory viruses) to search MEDLINE and CENTRAL. We combined the MEDLINE search strategy with the Cochrane Highly Sensitive Search Strategy for identifying randomised trials in MEDLINE: sensitivity‐ and precision‐maximising version (2008 revision) (Ovid format) (Lefebvre 2011). We also included an additional search strategy based on the work of Fraser, Murray and Burr (Fraser 2006) to identify observational studies.

1 Influenza, Human/ 2 exp Influenzavirus A/ 3 exp Influenzavirus B/ 4 Influenzavirus C/ 5 (influenza* or flu).tw. 6 Common Cold/ 7 common cold*.tw. 8 Rhinovirus/ 9 rhinovir*.tw. 10 adenoviridae/ or mastadenovirus/ or adenoviruses, human/ 11 adenoviridae infections/ or adenovirus infections, human/ 12 adenovir*.tw. 13 coronavirus/ or coronavirus 229e, human/ or coronavirus oc43, human/ or infectious bronchitis virus/ or sars virus/ 14 coronavir*.tw. 15 coronavirus infections/ or severe acute respiratory syndrome/ 16 (severe acute respiratory syndrome* or sars).tw. 17 respiratory syncytial viruses/ or respiratory syncytial virus, human/ 18 Respiratory Syncytial Virus Infections/ 19 (respiratory syncytial virus* or rsv).tw. 20 Pneumovirus Infections/ 21 parainfluenza virus 1, human/ or parainfluenza virus 3, human/ 22 parainfluenza virus 2, human/ or parainfluenza virus 4, human/ 23 (parainfluenza* or para‐influenza* or para influenza).tw. 24 enterovirus a, human/ or exp enterovirus b, human/ or enterovirus c, human/ or enterovirus d, human/ 25 Enterovirus Infections/ 26 enterovir*.tw. 27 Human bocavirus/ 28 bocavirus*.tw. 29 Metapneumovirus/ 30 metapneumovir*.tw. 31 Parvovirus B19, Human/ 32 parvoviridae infections/ or erythema infectiosum/ 33 parvovirus*.tw. 34 Parechovirus/ 35 parechovirus*.tw. 36 acute respiratory tract infection*.tw. 37 acute respiratory infection*.tw. 38 or/1‐37 39 Handwashing/ 40 (handwashing or hand washing or hand‐washing).tw. 41 hand hygiene.tw. 42 (sanitizer* or sanitiser*).tw. 43 (cleanser* or disinfectant*).tw. 44 gloves, protective/ or gloves, surgical/ 45 glov*.tw. 46 masks/ or respiratory protective devices/ 47 (mask or masks or respirator or respirators).tw. 48 Protective Clothing/ 49 Protective Devices/ 50 Patient Isolators/ 51 Patient Isolation/ 52 patient isolat*.tw. 53 (barrier* or curtain* or partition*).tw. 54 negative pressure room*.tw. 55 ((reverse barrier or reverse‐barrier) adj3 (nurs* or unit or isolation)).tw. 56 Cross Infection/pc [Prevention & Control] 57 (cross infection* adj2 prevent*).tw. 58 Communicable Disease Control/ 59 Infection Control/ 60 (school* adj3 (clos* or dismissal*)).tw. 61 temporary closur*.tw. 62 mass gathering*.tw. 63 (public adj2 (gathering* or event*)).tw. 64 (bans or banning or banned or ban).tw. 65 (outbreak adj3 control*).tw. 66 distancing*.tw. 67 Quarantine/ 68 quarantine*.tw. 69 (protective adj2 (cloth* or garment* or device* or equipment)).tw. 70 ((protective or preventive) adj2 (procedure* or behaviour* or behavior*)).tw. 71 personal protect*.tw. 72 (isolation room* or isolation strateg*).tw. 73 (distance adj2 patient*).tw. 74 ((spatial or patient) adj separation).tw. 75 cohorting.tw. 76 or/39‐75 77 38 and 76 78 (animals not (animals and humans)).sh. 79 77 not 78

**Ovid MEDLINE**

1 Influenza, Human/ 2 exp Influenzavirus A/ 3 exp Influenzavirus B/ 4 Influenzavirus C/ 5 (influenza* or flu).tw. 6 Common Cold/ 7 common cold*.tw. 8 Rhinovirus/ 9 rhinovir*.tw. 10 adenoviridae/ or mastadenovirus/ or adenoviruses, human/ 11 adenoviridae infections/ or adenovirus infections, human/ 12 adenovir*.tw. 13 coronavirus/ or coronavirus 229e, human/ or coronavirus oc43, human/ or infectious bronchitis virus/ or sars virus/ 14 coronavir*.tw. 15 coronavirus infections/ or severe acute respiratory syndrome/ 16 (severe acute respiratory syndrome* or sars).tw. 17 respiratory syncytial viruses/ or respiratory syncytial virus, human/ 18 Respiratory Syncytial Virus Infections/ 19 (respiratory syncytial virus* or rsv).tw. 20 Pneumovirus Infections/ 21 parainfluenza virus 1, human/ or parainfluenza virus 3, human/ 22 parainfluenza virus 2, human/ or parainfluenza virus 4, human/ 23 (parainfluenza* or para‐influenza* or para influenza).tw. 24 enterovirus a, human/ or exp enterovirus b, human/ or enterovirus c, human/ or enterovirus d, human/ 25 Enterovirus Infections/ 26 enterovir*.tw. 27 Human bocavirus/ 28 bocavirus*.tw. 29 Metapneumovirus/ 30 metapneumovir*.tw. 31 Parvovirus B19, Human/ 32 parvoviridae infections/ or erythema infectiosum/ 33 parvovirus*.tw. 34 Parechovirus/ 35 parechovirus*.tw. 36 acute respiratory tract infection*.tw. 37 acute respiratory infection*.tw. 38 or/1‐37 39 Handwashing/ 40 (handwashing or hand washing or hand‐washing).tw. 41 hand hygiene.tw. 42 (sanitizer* or sanitiser*).tw. 43 (cleanser* or disinfectant*).tw. 44 gloves, protective/ or gloves, surgical/ 45 glov*.tw. 46 masks/ or respiratory protective devices/ 47 (mask or masks or respirator or respirators).tw. 48 Protective Clothing/ 49 Protective Devices/ 50 Patient Isolators/ 51 Patient Isolation/ 52 patient isolat*.tw. 53 (barrier* or curtain* or partition*).tw. 54 negative pressure room*.tw. 55 ((reverse barrier or reverse‐barrier) adj3 (nurs* or unit or isolation)).tw. 56 Cross Infection/pc [Prevention & Control] 57 (cross infection* adj2 prevent*).tw. 58 Communicable Disease Control/ 59 Infection Control/ 60 (school* adj3 (clos* or dismissal*)).tw. 61 temporary closur*.tw. 62 mass gathering*.tw. 63 (public adj2 (gathering* or event*)).tw. 64 (bans or banning or banned or ban).tw. 65 (outbreak adj3 control*).tw. 66 distancing*.tw. 67 Quarantine/ 68 quarantine*.tw. 69 (protective adj2 (cloth* or garment* or device* or equipment)).tw. 70 ((protective or preventive) adj2 (procedure* or behaviour* or behavior*)).tw. 71 personal protect*.tw. 72 (isolation room* or isolation strateg*).tw. 73 (distance adj2 patient*).tw. 74 ((spatial or patient) adj separation).tw. 75 cohorting.tw. 76 or/39‐75 77 38 and 76 78 (animals not (animals and humans)).sh. 79 77 not 78

**Embase.com search strategy, October 2010** The search strategy was broadened in 2010 to be more inclusive of new and emerging viruses.

#3 #1 AND #25899 #2 766172 #2.8 #2.3 NOT #2.7766172 #2.7 #2.4 NOT #2.6 #2.6 #2.4 AND #2.5 #2.5 'human'/de AND [embase]/lim #2.4 'animal'/de OR 'nonhuman'/de OR 'animal experiment'/de AND [embase]/lim #2.3 #2.1 OR #2.2 #2.2 random*:ab,ti OR placebo*:ab,ti OR crossover*:ab,ti OR 'cross over':ab,ti OR allocat*:ab,ti OR trial:ti OR (doubl* NEXT/1 blind*):ab,ti AND [embase]/lim #2.1 'randomized controlled trial'/exp OR 'single blind procedure'/exp OR 'double blind procedure'/exp OR 'crossover procedure'/exp AND [embase]/lim

#1 74545 #1.65 #1.28 AND #1.6474545 #1.64 #1.29 OR #1.30 OR #1.31 OR #1.32 OR #1.33 OR #1.34 OR #1.35 OR #1.36 OR #1.37 OR #1.38 OR #1.39 OR #1.40 OR #1.41 OR #1.42 OR #1.43 OR #1.44 OR #1.45 OR #1.46 OR #1.47 OR #1.48 OR #1.49 OR #1.50 OR #1.51 OR #1.52 OR #1.53 OR #1.54 OR #1.55 OR #1.56 OR #1.57 OR #1.58 OR #1.59 OR #1.60 OR #1.61 OR #1.62 OR #1.63 #1.63 cohorting:ab,ti OR 'cohort isolation':ab,ti AND [embase]/lim #1.62 ((spatial OR patient*) NEAR/2 separation):ab,ti AND [embase]/lim #1.61 (distance NEAR/2 patient*):ab,ti AND [embase]/lim #1.60 (isolation NEXT/1 (room* OR strateg*)):ab,ti AND [embase]/lim #1.59 'personal protection':ab,ti AND [embase]/lim #1.58 ((protective OR preventive) NEAR/2 (procedure* OR behaviour* OR behavior*)):ab,ti AND [embase]/lim #1.57 (protective NEAR/2 (cloth* OR garment* OR device* OR equipment)):ab,ti AND [embase]/lim #1.56 quarantin*:ab,ti AND [embase]/lim #1.55 distancing:ab,ti AND [embase]/lim #1.54 ((outbreak* OR transmission OR infection*) NEAR/2 control):ab,ti AND [embase]/lim #1.53 bans:ab,ti OR banning:ab,ti OR banned:ab,ti OR ban:ab,ti AND [embase]/lim #1.52 (public NEAR/2 (gathering* OR event*)):ab,ti AND [embase]/lim #1.51 'mass gathering':ab,ti OR 'mass gatherings':ab,ti AND [embase]/lim #1.50 (temporar* NEAR/2 closur*):ab,ti AND [embase]/lim #1.49 (school* NEAR/3 (clos* OR dismissal*)):ab,ti AND [embase]/lim #1.48 'infection control'/de AND [embase]/lim #1.47 'epidemic'/dm_pc AND [embase]/lim #1.46 (('cross infection' OR 'cross infections') NEAR/2 prevent*):ab,ti AND [embase]/lim #1.45 'cross infection'/dm_pc AND [embase]/lim #1.44 (('reverse barrier' OR 'reverse‐barrier') NEAR/3 (nurs* OR unit OR isolat*)):ab,ti AND [embase]/lim #1.43 'negative pressure room':ab,ti OR 'negative pressure rooms':ab,ti AND [embase]/lim #1.42 barrier*:ab,ti OR curtain*:ab,ti OR partition*:ab,ti AND [embase]/lim #1.41 (patient* NEAR/2 isolat*):ab,ti AND [embase]/lim #1.40 'patient isolator'/de AND [embase]/lim #1.39 'protective equipment'/de AND [embase]/lim #1.38 'protective clothing'/de AND [embase]/lim #1.37 facemask*:ab,ti OR mask:ab,ti OR masks:ab,ti OR goggles:ab,ti OR respirator*:ab,ti OR respirators:ab,ti AND [embase]/lim #1.36 'face mask'/exp OR 'mask'/de OR 'surgical mask'/de AND [embase]/lim #1.35 glov*:ab,ti AND [embase]/lim #1.34 'surgical glove'/de AND [embase]/lim #1.33 cleanser*:ab,ti OR disinfect*:ab,ti OR antiseptic*:ab,ti OR virucid*:ab,ti AND [embase]/lim #1.32 sanitizer*:ab,ti OR sanitiser*:ab,ti AND [embase]/lim #1.31 (alcohol NEAR/2 rub*):ab,ti AND [embase]/lim #1.30 handwash*:ab,ti OR (hand* NEAR/2 (wash* OR cleans* OR hygiene)):ab,ti AND [embase]/lim #1.29 'hand washing'/de AND [embase]/lim #1.28 #1.1 OR #1.2 OR #1.3 OR #1.4 OR #1.5 OR #1.6 OR #1.7 OR #1.8 OR #1.9 OR #1.10 OR #1.11 OR #1.12 OR #1.13 OR #1.14 OR #1.15 OR #1.16 OR #1.17 OR #1.18 OR #1.19 OR #1.20 OR #1.21 OR #1.22 OR #1.23 OR #1.24 OR #1.25 OR #1.26 OR #1.27 #1.27 (respiratory NEAR/2 (infect* OR illness* OR virus* OR pathogen* OR acute)):ab,ti AND [embase]/lim #1.26 parechovirus*:ab,ti AND [embase]/lim #1.25 'parechovirus'/de AND [embase]/lim #1.24 parvovirus*:ab,ti AND [embase]/lim #1.23 'parvovirus infection'/de OR 'erythema infectiosum'/exp AND [embase]/lim #1.22 'parvovirus'/de OR 'human parvovirus b19'/de AND [embase]/lim #1.21 'human metapneumovirus'/de OR 'human metapneumovirus infection'/de AND [embase]/lim #1.20 'bocavirus'/de OR 'bocavirus infection'/de AND [embase]/lim #1.19 enterovir*:ab,ti AND [embase]/lim #1.18 'enterovirus infection'/de OR 'coxsackie virus infection'/de OR 'echovirus infection'/de AND [embase]/lim #1.17 'enterovirus'/de OR 'coxsackie virus'/exp OR 'echo virus'/de AND [embase]/lim #1.16 parainfluenza:ab,ti OR 'para influenza':ab,ti OR 'para‐influenza':ab,ti AND [embase]/lim #1.15 'parainfluenza virus'/exp AND [embase]/lim #1.14 'pneumovirus infection'/de AND [embase]/lim #1.13 'respiratory syncytial virus':ab,ti OR 'respiratory syncytial viruses':ab,ti OR rsv:ab,ti AND [embase]/lim #1.12 'respiratory syncytial pneumovirus'/de OR 'respiratory syncytial virus infection'/exp AND [embase]/lim #1.11 coronavir*:ab,ti OR sars:ab,ti OR 'severe acute respiratory syndrome':ab,ti AND [embase]/lim #1.10 'coronavirus infection'/de OR 'severe acute respiratory syndrome'/de AND [embase]/lim #1.9 'coronavirus'/de OR 'human coronavirus nl63'/de OR 'sars coronavirus'/de OR 'transmissible gastroenteritis virus'/de #1.8 adenovir*:ab,ti AND [embase]/lim #1.7 'adenovirus infection'/de OR 'human adenovirus infection'/de OR 'human adenovirus'/exp AND [embase]/lim #1.6 rhinovir*:ab,ti AND [embase]/lim #1.5 'rhinovirus infection'/de OR 'human rhinovirus'/de AND [embase]/lim #1.4 'common cold':ab,ti OR 'common colds':ab,ti OR coryza:ab,ti OR colds:ab,ti AND [embase]/lim #1.3 'common cold'/de OR 'common cold symptom'/de AND [embase]/lim #1.2 influenza*:ab,ti OR flu:ab,ti AND [embase]/lim #1.1 'influenza'/exp AND [embase]/lim

**CINAHL (EBSCO) search strategy, October 2010** The search strategy was broadened in 2010 to be more inclusive of new and emerging viruses.

S54 S32 and S53 S53 S44 or S52 S52 S45 or S46 or S47 or S48 or S49 or S50 or S51 S51 TI observational stud* or AB observational stud* S50 TI cohort stud* or AB cohort stud* S49 (MH "Cross Sectional Studies") S48 (MH "Nonconcurrent Prospective Studies") S47 (MH "Correlational Studies") S46 (MH "Case Control Studies+") S45 (MH "Prospective Studies") S44 S33 or S34 or S35 or S36 or S37 or S38 or S39 or S40 or S41 or S42 or S43 S43 TI allocat* N1 random* or AB allocat* N1 random* S42 (MH "Quantitative Studies") S41 TI placebo* or AB placebo* S40 (MH "Placebos") S39 TI random* allocation* or AB random* allocation* S38 (MH "Random Assignment") S37 TI ( randomised control* trial* or randomized control* trial* ) or AB ( randomised control* trial* or randomized control* trial ) S36 TI ( (singl* W1 blind*) or (singl* W1 mask*) or (doubl* W1 blind*) or (doubl* W1 mask*) or (trebl* W1 blind*) or (trebl* W1 mask*) or (tripl* W1 blind*) or (tripl* W1 mask*) ) or AB ( (singl* W1 blind*) or (singl* W1 mask*) or (doubl* W1 blind*) or (doubl* W1 mask*) or (trebl* W1 blind*) or (trebl* W1 mask*) or (tripl* W1 blind*) or (tripl* W1 mask*) ) S35 TI clinic* W1 trial* or AB clinic* W1 trial* S34 PT clinical trial S33 (MH "Clinical Trials+") S32 S15 and S31 S31 S16 or S17 or S18 or S19 or S20 or S21 or S22 or S23 or S24 or S25 or S26 or S27 or S28 or S29 or S30 S30 TI ( bans or banning or banned or ban or "outbreak control" or "outbreak controls" or distancing* or quarantine* or "protective clothing" or "protective garment" or "protective garments" or "protective gown" or "protective gowns" or "protective device" or "protective devices" or "protective equipment" or "protective behaviour" or "protective behavior" or "protective behaviours" or "protective behaviors" or "protective procedure" or "protective procedures" or "preventive behaviours" or "preventive behaviour" or "preventive behavior" or "preventive behaviors" or "preventive procedure" or "preventive procedures" or "personal protective" or "isolation room" or "isolation rooms" or "isolation strategy" or "isolation strategies" or "patient distance" or "patient distancing" or "patient separation" or "spatial separation" ) or AB (handwashing or "hand washing" or hand‐washing or "hand hygiene" or sanitizer or sanitiser or cleanser* or disinfectant* or glov* or mask or masks or respirator or respirators or "patient isolation" or "patient isolators" or barrier* or curtain* or partition* or "negative pressure room" or "negative pressure rooms" or "reverse barrier nursing" or "reverse barrier unit" or "reverse barrier isolation" or "cross infection" or "infection control" or "disease control" or "school closure" or "school closures" or "school dismissal" or "school dismissals" or "temporary closure" or "temporary closures" or "mass gathering" or "mass gatherings" or "public gathering" or "public gatherings" or "public event" or "public events" ) S29 TI ( handwashing or "hand washing" or hand‐washing or "hand hygiene" or sanitizer or sanitiser or cleanser* or disinfectant* or glov* or mask or masks or respirator or respirators or "patient isolation" or "patient isolators" or barrier* or curtain* or partition* or "negative pressure room" or "negative pressure rooms" or "reverse barrier nursing" or "reverse barrier unit" or "reverse barrier isolation" or "cross infection" or "infection control" or "disease control" or "school closure" or "school closures" or "school dismissal" or "school dismissals" or "temporary closure" or "temporary closures" or "mass gathering" or "mass gatherings" or "public gathering" or "public gatherings" or "public event" or "public events" ) or AB ( handwashing or "hand washing" or hand‐washing or "hand hygiene" or sanitizer or sanitiser or cleanser* or disinfectant* or glov* or mask or masks or respirator or respirators or "patient isolation" or "patient isolators" or barrier* or curtain* or partition* or "negative pressure room" or "negative pressure rooms" or "reverse barrier nursing" or "reverse barrier unit" or "reverse barrier isolation" or "cross infection" or "infection control" or "disease control" or "school closure" or "school closures" or "school dismissal" or "school dismissals" or "temporary closure" or "temporary closures" or "mass gathering" or "mass gatherings" or "public gathering" or "public gatherings" or "public event" or "public events" ) S28 (MH "Sterilization and Disinfection") S27 (MH "Quarantine") S26 (MH "Area Restriction (Iowa NIC)") OR (MH "Infection Protection (IowaNIC)") S25 (MH "Infection Control") S24 (MH "Cross Infection/PC") S23 (MH "Isolation, Reverse") S22 (MH "Patient Isolation") S21 (MH "Protective Devices") S20 (MH "Protective Clothing") S19 (MH "Respiratory Protective Devices") S18 (MH "Masks") S17 (MH "Gloves") S16 (MH "Handwashing+") S15 S1 or S2 or S3 or S4 or S5 or S6 or S7 or S8 or S9 or S10 or S11 or S12 or S13 or S14 S14 TI ( "acute respiratory tract infection" or "acute respiratory tract infections" or "acute respiratory infection" or "acute respiratory infections" ) or AB ( influenza* or flu or "common cold" or "common colds" or rhinovir* or adenovir* or coronavir* or sars or "severe acute respiratory syndrome" or "respiratory syncytial virus" or "respiratory syncytial viruses" or rsv or pneumovir* or parainfluenza* or "para influenza" or para‐influenza or enterovir* or bocavir* or metapneumovir* or parvovir* or parechovir* ) S13 TI ( influenza* or flu or "common cold" or "common colds" or rhinovir* or adenovir* or coronavir* or sars or "severe acute respiratory syndrome" or "respiratory syncytial virus" or "respiratory syncytial viruses" or rsv or pneumovir* or parainfluenza* or "para influenza" or para‐influenza or enterovir* or bocavir* or metapneumovir* or parvovir* or parechovir* ) or AB ( influenza* or flu or "common cold" or "common colds" or rhinovir* or adenovir* or coronavir* or sars or "severe acute respiratory syndrome" or "respiratory syncytial virus" or "respiratory syncytial viruses" or rsv or pneumovir* or parainfluenza* or "para influenza" or para‐influenza or enterovir* or bocavir* or metapneumovir* or parvovir* or parechovir* ) S12 (MH "Respiratory Tract Infections+") S11 (MH "Parvovirus Infections+") S10 (MH "Enterovirus Infections+") S9 (MH "Enteroviruses+") S8 (MH "Respiratory Syncytial Virus Infections") S7 (MH "Respiratory Syncytial Viruses") S6 (MH "SARS Virus") S5 (MH "Severe Acute Respiratory Syndrome") S4 (MH "Coronavirus Infections+") S3 (MH "Coronavirus+") OR (MH "Coronavirus Infections") S2 (MH "Common Cold") S1 (MH "Influenza+") OR (MH "Influenza A H5N1") OR (MH "Influenza A

**LILACS (Latin America and Caribbean) search strategy** (mh:"Influenza, Human" OR "Gripe Humana" OR "Influenza Humana" OR influenza* OR flu OR grippe OR gripe OR mh:"Influenzavirus A" OR mh:b04.820.545.405* OR mh:b04.909.777.545.405* OR mh:"Influenzavirus B" OR mh:b04.820.545.407* OR mh:b04.909.777.545.407* OR "influenzavirus B" OR mh:"Influenzavirus C" OR "Influenzavirus C" OR mh:"Common Cold" OR "common cold" OR "common colds" OR "Resfriado Común" OR "Resfriado Comum" OR coryza OR "Coriza Aguda") AND (mh:handwashing OR "Lavado de Manos" OR "Lavagem de Mãos" OR "Desinfección de Manos" OR "Desinfecção de Mãos" OR "Higienização de Mãos Pré‐Cirúrgica" OR handwash* OR "hand washing" OR "hand hygiene" OR "hand cleaning" OR "hand cleanse" OR "hand cleansing" OR higiene OR sanitizer* OR sanitiser* OR cleanser* OR disinfect* OR esteriliza* OR desinfectar* OR virucid* OR antiseptic* OR mh:"Gloves, Protective" OR "protective glove" OR "protective gloves" OR "Guantes Protectores" OR "Luvas Protetoras" OR mh:e07.700.600.400* OR mh:j01.637.215.600.400* OR mh:j01.637.708.600.400* OR glov* OR guantes OR luvas OR mh:masks OR mask* OR máscaras OR mascarillas OR facemask* OR goggles OR respirator* OR mh:"Respiratory Protective Devices" OR "Dispositivos de Protección Respiratoria" OR "Dispositivos de Proteção Respiratória" OR mh:"Protective Clothing" OR "Ropa de Protección" OR "Roupa de Proteção" OR mh:e07.700.600* OR mh:j01.637.215.600* OR mh:j01.637.708.600* OR mh:"Protective Devices" OR "Equipos de Seguridad" OR "Equipamentos de Proteção" OR mh:e07.700* OR mh:j01.637.708* OR mh:vs2.006.001.001* OR mh:vs4.002.001.001.007.002.002* OR mh:"Patient Isolation" OR "patient isolation" OR "Aislamiento de Pacientes" OR "Isolamento de Pacientes" OR mh:"Patient Isolators" OR "patient isolators" OR "Aisladores de Pacientes" OR "Isoladores de Pacientes" OR barrier* OR curtain* OR partition* OR barrera OR barreira OR cortina OR tabique OR mh:"Cross Infection" OR "cross infection" OR "Infección Hospitalaria" OR "Infecção Hospitalar" OR "Infecciones en Hospitales" OR "Infecciones Nosocomiales" OR "Infecções Nosocomiais" OR mh:"Infection Control" OR mh:n06.850.780.200.450* OR "Control de Infecciones" OR "Controle de Infecções" OR mh:"Communicable Disease Control" OR "Control de Enfermedades Transmisibles" OR "Controle de Doenças Transmissíveis" OR mh:n06.850.780.200* OR mh:sp8.946.819.811* OR mh:"Disease Outbreaks/prevention & control" OR mh:quarantine OR cuarentena OR quarentena OR "personal protection" OR "isolation room" OR "sala de aislamiento" OR "quarto de isolamento" OR "patient distance" OR "distancia del paciente" OR "spatial separation" OR cohort* OR ban OR bans OR banning OR banned OR prohibici* OR proibi* OR "outbreak control" OR distanc* OR "school closure" OR "school closures" OR "temporary closure" OR "temporary closures" OR "cierre de la escuela" OR "fechamento da escola" OR "public gathering" OR "public gatherings" OR "reunion publica" OR "reverse barrier nursing" OR "reverse barrier unit" OR "reverse barrier isolation" OR "negative pressure room" OR "negative pressure rooms" OR "patient separation") AND db:("LILACS") AND type_of_study:("clinical_trials" OR "cohort" OR "case_control")

**Indian MEDLARS search strategy** (influenza$ or flu or common cold$ or rhinovir$ or coronavir$ or adenovir$ or severe acute respiratory syndrome$ or sars or respiratory syncytial virus$ or rsv or parainfluenza$ or enterovir$ or metapneumovir$ or parvovir$ or bocavir$ or parechovir$) and (handwashing or hand washing or mask$ or glov$ or protect$ or isolat$ or barrier$ or curtain$ or partition$ or cross infection$ or infection control$ or disease control$ or school$ or quarantine$ or ban$ or cohort$ or distanc$ or spatial separation$) IMSEAR (Index Medicus for the South East Asia Region) search strategy (influenza or flu or common cold or rhinovirus or coronavirus or adenovirus or severe acute respiratory syndrome or sars or respiratory syncytial virus or rsv or parainfluenza or enterovirus or bocavirus or metapneumovirus or parvovirus or parechovirus) and (handwashing or hand washing or hand hygiene or sanitizer or sanitiser or cleanser or disinfectant or gloves or masks or mask or protective clothing or protective devices or patient isolation or barrier or curtain or partition or cross infection or disease control or infection control or school or schools or bans or banning or banned or ban or distancing or quarantine or isolation or spatial separation or cohorting or cohort isolation)

In the first publication of this review we searched the Cochrane Central Register of Controlled Trials (CENTRAL) (The Cochrane Library 2006, issue 4); MEDLINE (1966 to November 2006); OLDMEDLINE (1950 to 1965); EMBASE (1990 to November 2006) and CINAHL (1982 to November 2006). The MEDLINE search terms were modified for OLDMEDLINE, EMBASE and CINAHL.

In this 2009 update we searched the Cochrane Central Register of Controlled Trials (CENTRAL) (The Cochrane Library 2009, issue 2); Ovid MEDLINE (2006 to May Week 1 2009); OLDMEDLINE (1950 to 1965); Ovid EMBASE (2006 to Week 18, 2009) and Ovid CINAHL (2006 to May Week 1 2009).

**Ovid MEDLINE** 1 exp Influenza/ 2 influenza.tw. 3 flu.tw. 4 exp Common Cold/ 5 common cold.tw. 6 exp Rhinovirus/ 7 rhinovirus*.tw. 8 exp Adenoviridae/ 9 adenovirus*.tw. 10 exp Coronavirus/ 11 exp Coronavirus Infections/ 12 coronavirus*.tw. 13 exp Respiratory Syncytial Viruses/ 14 exp Respiratory Syncytial Virus Infections/ 15 respiratory syncytial virus*.tw. 16 respiratory syncythial virus.tw. 17 exp Parainfluenza Virus 1, Human/ 18 exp Parainfluenza Virus 2, Human/ 19 exp Parainfluenza Virus 3, Human/ 20 exp Parainfluenza Virus 4, Human/ 21 (parainfluenza or para‐influenza or para influenza).tw. 22 exp Severe Acute Respiratory Syndrome/ 23 (severe acute respiratory syndrome or SARS).tw. 24 acute respiratory infection*.tw. 25 acute respiratory tract infection*.tw. 26 or/1‐25 (59810) 27 exp Hand Washing/ 28 (handwashing or hand washing or hand‐washing).tw. 29 hand hygiene.tw. 30 (sanitizer* or sanitiser*).tw. 31 (cleanser* or disinfectant*).tw. 32 exp Gloves, Protective/ 33 exp Gloves, Surgical/ 34 glov*.tw. 35 exp Masks/ 36 mask*1.tw. 37 exp Patient Isolators/ 38 exp Patient Isolation/ 39 patient isolat*.tw. 40 (barrier* or curtain* or partition*).tw. 41 negative pressure room*.tw. 42 reverse barrier nursing.tw. 43 Cross Infection/pc [Prevention] 44 school closure*.tw. 45 (clos* adj3 school*).tw. 46 mass gathering*.tw. 47 public gathering*.tw. 48 (ban or bans or banned or banning).tw. 49 (outbreak* adj3 control*).tw. 50 distancing.tw. 51 exp Quarantine/ 52 quarantine*.tw. 53 or/27‐49 54 26 and 53 55 (animals not (humans and animals)).sh. 56 54 not 55

**CENTRAL search strategy** #1 MeSH descriptor Influenza, Human explode all trees #2 influenza:ti,ab,kw #3 flu:ti,ab,kw #4 MeSH descriptor Common Cold explode all trees #5 "common cold":ti,ab,kw #6 MeSH descriptor Rhinovirus explode all trees #7 rhinovirus*:ti,ab,kw #8 MeSH descriptor Adenoviridae explode all trees #9 adenovirus*:ti,ab,kw #10 MeSH descriptor Coronavirus explode all trees #11 MeSH descriptor Coronavirus Infections explode all trees #12 coronavirus*:ti,ab,kw #13 MeSH descriptor Respiratory Syncytial Viruses explode all trees #14 MeSH descriptor Respiratory Syncytial Virus Infections explode all trees #15 respiratory syncytial virus*:ti,ab,kw #16 respiratory syncythial virus*:ti,ab,kw #17 MeSH descriptor Parainfluenza Virus 1, Human explode all trees #18 MeSH descriptor Parainfluenza Virus 2, Human explode all trees #19 MeSH descriptor Parainfluenza Virus 3, Human explode all trees #20 MeSH descriptor Parainfluenza Virus 4, Human explode all trees #21 (parainfluenza or para‐influenza or para influenza):ti,ab,kw #22 MeSH descriptor Severe Acute Respiratory Syndrome explode all trees #23 (severe acute respiratory syndrome or SARS):ti,ab,kw #24 acute respiratory infection*:ti,ab,kw #25 acute respiratory tract infection*:ti,ab,kw #26 (#1 OR #2 OR #3 OR #4 OR #5 OR #6 OR #7 OR #8 OR #9 OR #10 OR #11 OR #12 OR #13 OR #14 OR #15 OR #16 OR #17 OR #18 OR #19 OR #20 OR #21 OR #22 OR #23 OR #24 OR #25) #27 MeSH descriptor Handwashing explode all trees #28 (handwashing or hand washing or hand‐washing):ti,ab,kw #29 hand hygiene:ti,ab,kw #30 (sanitizer* or sanitiser*):ti,ab,kw #31 (cleanser* or disinfectant*):ti,ab,kw #32 MeSH descriptor Gloves, Protective explode all trees #33 MeSH descriptor Gloves, Surgical explode all trees #34 glov*:ti,ab,kw #35 MeSH descriptor Masks explode all trees #36 mask*:ti,ab,kw #37 MeSH descriptor Patient Isolators explode all trees #38 MeSH descriptor Patient Isolation explode all trees #39 (barrier* or curtain* or partition*):ti,ab,kw #40 negative NEXT pressure NEXT room*:ti,ab,kw #41 "reverse barrier nursing":ti,ab,kw #42 MeSH descriptor Cross Infection explode all trees with qualifier: PC #43 school NEXT closure*:ti,ab,kw #44 (clos* NEAR/3 school*):ti,ab,kw #45 mass NEXT gathering*:ti,ab,kw #46 public NEXT gathering*:ti,ab,kw #47 ("ban" or "bans" or banned or banning):ti,ab,kw #48 (outbreak* NEAR/3 control*):ti,ab,kw #49 distancing:ti,ab,kw #50 MeSH descriptor Quarantine explode all trees #51 quarantine*:ti,ab,kw #52 (#27 OR #28 OR #29 OR #30 OR #31 OR #32 OR #33 OR #34 OR #35 OR #36 OR #37 OR #38 OR #39 OR #40 OR #41 OR #42 OR #43 OR #44 OR #45 OR #46 OR #47 OR #48 OR #49 OR #50 OR #51) #53 (#26 AND #52)

**Ovid Embase search strategy** 1 exp Influenza/ 2 influenza.tw. 3 flu.tw. 4 exp Common Cold/ 5 common cold.tw. 6 exp Human Rhinovirus/ 7 rhinovirus*.tw. 8 exp Adenovirus/ 9 adenovirus*.tw. 10 exp Coronavirus/ 11 coronavirus*.tw. 12 exp Respiratory Syncytial Pneumovirus/ 13 respiratory syncytial virus*.tw. 14 respiratory syncythial virus.tw. 15 (parainfluenza or para‐influenza or para influenza).tw. 16 exp Severe Acute Respiratory Syndrome/ 17 (severe acute respiratory syndrome or SARS).tw. 18 acute respiratory infection*.tw. 19 acute respiratory tract infection*.tw. 20 or/1‐19 21 exp Hand Washing/ 22 (handwashing or hand washing or hand‐washing).tw. 23 hand hygiene.tw. 24 (sanitizer$ or sanitiser$).tw. 25 (cleanser$ or disinfectant$).tw. 26 exp Glove/ 27 exp Surgical Glove/ 28 glov*.tw. 29 exp Mask/ 30 mask*1.tw. 31 patient isolat*.tw. 32 (barrier* or curtain* or partition*).tw. 33 negative pressure room*.tw. 34 reverse barrier nursing.tw. 35 Cross Infection/pc [Prevention] 36 school closure*.tw. 37 (clos* adj3 school*).tw. 38 mass gathering*.tw. 39 public gathering*.tw. (5) 40 (ban or bans or banned or banning).tw. 41 (outbreak* adj3 control*).tw. 42 distancing.tw. 43 quarantine*.tw. 44 or/21‐43 45 20 and 44

**EBSCO CINAHL search strategy** S26 S10 and S24 S25 S10 and S24 S24 S11 or S12 or S13 or S14 or S15 or S16 or S17 or S18 or S19 or S20 or S21 or S22 or 23 or S24 S23 TI outbreak* N3 control* or AB outbreak* N3 control* S22 TI ( school closure* or mass gathering* or public gathering* or ban or bans or banned or banning or distancing or quarantine* ) or AB ( school closure* or mass gathering* or public gathering* or ban or bans or banned or banning or distancing or quarantine* ) S21 TI ( patient isolat* or barrier* or curtain* or partition* or negative pressure room* or reverse barrier nursing) or AB ( patient isolat* or barrier* or curtain* or partition* or negative pressure room* or reverse barrier nursing) S20 TI ( glov* or mask* ) or AB ( glov* or mask* ) S19 TI ( handwashing or hand washing or hand‐washing or hand hygiene ) or AB (handwashing or hand washing or hand‐washing or hand hygiene ) S18 (MH "Quarantine") S17 (MM "Cross Infection") S16 (MH "Isolation, Reverse") S15 (MH "Patient Isolation+") S14 (MH "Respiratory Protective Devices") S13 (MH "Masks") S12 (MH "Gloves") S11 (MH "Handwashing+") S10 S1 or S2 or S3 or S4 or S5 or S6 or S7 or S8 or S9 S9 TI ( influenza or flu or rhinovirus* or adenovirus* or coronavirus* or respiratory syncytial virus* or respiratory syncythial virus* or parainfluenza or para‐influenza or para influenza or severe acute respiratory syndrome or SARS or respiratory viral infection* or viral respiratory infection* ) or AB ( influenza or flu or rhinovirus* or adenovirus* or coronavirus* or respiratory syncytial virus* or respiratory syncythial virus* or parainfluenza or para‐influenza or para influenza or severe acute respiratory syndrome or SARS or respiratory viral infection* or viral respiratory infection* )TI ( influenza or flu or rhinovirus* or adenovirus* or coronavirus* or respiratory syncytial virus* or respiratory syncythial virus* or parainfluenza or para‐influenza or para influenza or severe acute respiratory (syndrome or SARS or respiratory viral infection* or viral respiratory infection*) or AB (influenza or flu or rhinovirus* or adenovirus* or coronavirus* or respiratory syncytial virus* or respiratory syncythial virus* or parainfluenza or para‐influenza or para influenza or severe acute respiratory syndrome or SARS or respiratory viral infection* or viral respiratory infection* ) S8 (MH "SARS Virus") S7 (MH "Severe Acute Respiratory Syndrome") S6 (MH "Respiratory Syncytial Virus Infections") S5 (MH "Respiratory Syncytial Viruses") S4 (MH "Coronavirus+") S3 (MH "Coronavirus Infections+") S2 (MH "Common Cold") S1 (MH "Influenza+")

## Data and analyses

**Comparison 1:** Randomised trials: medical/surgical masks versus no masks

Outcome or subgroup title	No. of studies	No. of participants	Statistical method	Effect size
1.1 Viral illness	10		Risk Ratio (IV, Random, 95% CI)	Subtotals only
1.1.1 Influenza/COVID‐like illness	9	276917	Risk Ratio (IV, Random, 95% CI)	0.95 [0.84, 1.09]
1.1.2 Laboratory‐confirmed influenza or SARS‐cov‐2	6	13919	Risk Ratio (IV, Random, 95% CI)	1.01 [0.72, 1.42]
1.1.3 Laboratory‐confirmed other respiratory viruses	1	4862	Risk Ratio (IV, Random, 95% CI)	0.58 [0.25, 1.31]

**Comparison 2:** Randomised trials: N95 respirators compared to medical/surgical masks

Outcome or subgroup title	No. of studies	No. of participants	Statistical method	Effect size
2.1 Viral illness	5		Risk Ratio (IV, Random, 95% CI)	Subtotals only
2.1.1 Clinical respiratory illness	3	7799	Risk Ratio (IV, Random, 95% CI)	0.70 [0.45, 1.10]
2.1.2 Influenza‐like illness	5	8407	Risk Ratio (IV, Random, 95% CI)	0.82 [0.66, 1.03]
2.1.3 Laboratory‐confirmed influenza	5	8407	Risk Ratio (IV, Random, 95% CI)	1.10 [0.90, 1.34]
2.2 Viral illness in healthcare workers	4		Risk Ratio (IV, Random, 95% CI)	Subtotals only
2.2.1 Clinical respiratory illness	3	7799	Risk Ratio (IV, Random, 95% CI)	0.70 [0.45, 1.10]
2.2.2 Influenza‐like illness	4	8221	Risk Ratio (IV, Random, 95% CI)	0.81 [0.59, 1.11]
2.2.3 Laboratory‐confirmed influenza	4	8221	Risk Ratio (IV, Random, 95% CI)	1.05 [0.79, 1.40]

**Comparison 3:** Randomised trials: hand hygiene compared to control

Outcome or subgroup title	No. of studies	No. of participants	Statistical method	Effect size
3.1 Viral illness	19		Risk Ratio (IV, Random, 95% CI)	Subtotals only
3.1.1 Acute respiratory illness	9	52105	Risk Ratio (IV, Random, 95% CI)	0.86 [0.81, 0.90]
3.1.2 Influenza‐like illness	11	34503	Risk Ratio (IV, Random, 95% CI)	0.94 [0.81, 1.09]
3.1.3 Laboratory‐confirmed influenza	8	8332	Risk Ratio (IV, Random, 95% CI)	0.91 [0.63, 1.30]
3.2 ARI or ILI or influenza (including outcome with most events from each study)	19	71210	Risk Ratio (IV, Random, 95% CI)	0.89 [0.83, 0.94]
3.3 Influenza or ILI: sensitivity analysis including outcomes with the most precise and unequivocal definitions	12	28205	Risk Ratio (IV, Random, 95% CI)	0.88 [0.77, 1.02]
3.4 ARI or ILI or influenza: subgroup analysis	19	71210	Risk Ratio (IV, Random, 95% CI)	0.89 [0.83, 0.94]
3.4.1 Children	11	29259	Risk Ratio (IV, Random, 95% CI)	0.91 [0.84, 0.98]
3.4.2 Adults	8	41951	Risk Ratio (IV, Random, 95% CI)	0.84 [0.78, 0.91]
3.5 Absenteeism	3	3150	Risk Ratio (IV, Random, 95% CI)	0.64 [0.58, 0.71]

**Comparison 4:** Randomised trials: hand hygiene + medical/surgical masks compared to control

Outcome or subgroup title	No. of studies	No. of participants	Statistical method	Effect size
4.1 Viral illness	6		Risk Ratio (IV, Random, 95% CI)	Subtotals only
4.1.1 Influenza‐like illness	6	4504	Risk Ratio (IV, Random, 95% CI)	1.03 [0.77, 1.37]
4.1.2 Laboratory‐confirmed Influenza	4	3121	Risk Ratio (IV, Random, 95% CI)	0.97 [0.69, 1.36]

**Comparison 5:** Randomised trials: hand hygiene + medical/surgical masks compared to hand hygiene

Outcome or subgroup title	No. of studies	No. of participants	Statistical method	Effect size
5.1 Viral illness	3		Risk Ratio (IV, Random, 95% CI)	Subtotals only
5.1.1 Influenza‐like illness	3	2982	Risk Ratio (IV, Random, 95% CI)	1.03 [0.69, 1.53]
5.1.2 Laboratory‐confirmed influenza	3	2982	Risk Ratio (IV, Random, 95% CI)	0.99 [0.69, 1.44]

**Comparison 6:** Randomised trials: gargling compared to control

Outcome or subgroup title	No. of studies	No. of participants	Statistical method	Effect size
6.1 Viral illness	2	830	Risk Ratio (IV, Random, 95% CI)	0.91 [0.63, 1.31]
6.2 SARS‐CoV‐2	2	394	Risk Ratio (IV, Random, 95% CI)	0.07 [0.02, 0.23]

## Characteristics of studies

### Characteristics of included studies [ordered by study ID]

**Table:** Abaluck 2022

***Study characteristics***		
Methods	Cluster‐RCT Randomisation unit: villages (N = 600) Intervention duration: 8 weeks “Our intervention was designed to last 8 weeks in each village”	
Participants	Inclusion criteria: community level participants Intervention = 178,322 individuals, control = 163,861 individuals (Total N = 342,183 adults)	
Interventions	2 types of mask used: surgical and cloth masks PLUS a brief video of notable public figures discussing why, how, and when to wear a mask, PLUS a brochure based on WHO materials depicting proper mask‐wearing. Control villages: the control group did not receive any interventions See Table 4 for details.	
Outcomes	Effectiveness: primary outcome: symptomatic seroprevalence (symptomatic and seropositive) Laboratory: seropositivity was defined by having detectable IgG antibodies against SARS‐CoV‐2 Symptoms defined as per WHO‐defined COVID‐19 symptoms: (a) fever and cough; (b) 3 or more of the following symptoms (fever, cough, general weakness/fatigue, headache, myalgia, sore throat, coryza, dyspnoea, anorexia/nausea/vomiting, diarrhoea, altered mental status); or (c) loss of taste or smell. Secondary outcomes: prevalence of proper mask‐wearing as wearing either a project mask or an alternative face‐covering over the mouth and nose and improper mask‐wearing as wearing a mask in any way that did not fully cover the mouth and nose; prevalence of physical distancing per WHO guideline that defines physical distancing as one meter of separation; prevalence of symptoms consistent with COVID‐19: definition (see above) Safety not assessed. However, study mentioned that there was no adverse events reported during the study period	
Notes	The authors conclude that: a randomised trial of community‐level mask promotion in rural Bangladesh during the COVID‐19 pandemic shows that the intervention increased mask usage and reduced symptomatic SARS‐CoV‐2 infections, demonstrating that promoting community mask‐wearing can improve public health (a scalable and effective method to promote mask adoption and reduce symptomatic SARS‐CoV‐2 infections.) Funding: this trial was financially supported by a grant from GiveWell.org to Innovations for Poverty Action. The trial authors declare no competing interests.	
***Risk of bias***		
**Bias**	**Authors' judgement**	**Support for judgement**
Random sequence generation (selection bias)	Low risk	Random number generator used
Allocation concealment (selection bias)	High risk	Significant differences in the numbers of households included in each treatment group suggestive of a lack of allocation concealment
Blinding of participants and personnel (performance bias) All outcomes	High risk	Participants, mask promoters, and mask surveillance staff were not blinded as intervention materials were clearly visible
Blinding of outcome assessment (detection bias) All outcomes	High risk	Although the pre‐specified analyses and sample exclusions were made by analysts blinded to the treatment assignment, investigators dropped individuals who were missing symptom data or who did not consent to blood spot collection from the primary outcome. One of the outcomes is COVID‐19 symptoms reported by participants. Mask promoters, and mask surveillance staff were not blinded
Incomplete outcome data (attrition bias) All outcomes	High risk	Laboratory testing results were only available for around 40% of the symptomatic participants
Selective reporting (reporting bias)	High risk	Primary outcome of seroconversion was not reported

**Table:** Aelami 2015

***Study characteristics***		
Methods	A prospective cross‐sectional study conducted during the Hajj season 2012. Pilgrims were randomised into 2 groups. The intervention group received education on personal hygiene including a hygienic package containing alcohol‐based hand rub (gel or spray), surgical masks, soap, paper handkerchiefs, and user instructions; the control group did not receive any intervention. ILI was defined as the presence of at least 2 of the following during their stay: fever, cough, and sore throat. Questionnaires including demographic and clinical information were distributed amongst trained physicians before departure from Iran.	
Participants	Total enrolled: 664 Iranian pilgrims (306 in the intervention group and 358 in the control group) Inclusion criteria: not reported Exclusion criteria: not reported	
Interventions	Hygiene education and package. See Table 4 for details.	
Outcomes	ILI defined as the presence of at least 2 of the following during their stay: fever, cough, and sore throat. No safety outcomes were reported.	
Notes	This is an abstract, therefore few details were reported. Funding not mentioned. Disclosure of interest: none declared.	
***Risk of bias***		
**Bias**	**Authors' judgement**	**Support for judgement**
Random sequence generation (selection bias)	Unclear risk	Insufficient details provided
Allocation concealment (selection bias)	Unclear risk	Insufficient details provided
Blinding of participants and personnel (performance bias) All outcomes	High risk	Unblinded study
Blinding of outcome assessment (detection bias) All outcomes	Unclear risk	Insufficient details provided
Incomplete outcome data (attrition bias) All outcomes	Unclear risk	Insufficient details provided
Selective reporting (reporting bias)	Unclear risk	Insufficient details provided

**Table:** Aiello 2010

***Study characteristics***		
Methods	Cluster‐RCT assessing the effects of hand sanitiser and masks versus masks or no intervention on ILI symptoms. The trial was conducted in university halls of residence with more than 100 student residents in a US university during the 2006 to 2007 influenza “season”. The study lasted 6 weeks. The units of randomisation were 7 of the 15 halls. 1 hall was very large (1240 residents), and the 6 remaining ones, which had between 110 and 830 residents, were combined into 2 clusters roughly equivalent in size. The 3 clusters were then randomised by random extraction of the clustered halls’ names out of a container. The largest hall (single‐cluster) was randomised to the mask and hand sanitiser arm; the 4‐halls cluster received masks; and the remaining 2 halls were assigned as controls.	
Participants	A total of 1297 with completed baseline survey and at least 1 weekly survey result were analysed (face mask and hand hygiene group = 367; face mask–only group = 378; control group = 552). Inclusion criteria: aged 18 or more, willing to wear mask and use alcohol‐based hand sanitiser, have a throat swab specimen collected when ill, and complete the baseline and weekly surveys over the 6‐week study period Exclusion criteria: individuals reporting a skin allergy to alcohol were excluded Recruitment of students began in 26 November, but the trial did not go “live” with distribution of intervention materials until 22 January 2007 when the first case of influenza was confirmed on campus by laboratory tests. Enrolment continued until 16 February 2007, and the study was completed on 16 March 2007. During the study period there was a 1‐week break when the majority of residents left campus. There were 1327 eligible participants, 1297 of which had a complete baseline survey and at least 1‐weekly survey result. It is unclear what the ineligibility criteria were for the 30 missing (1327 minus 1297), but the explanation may be in the appendix.	
Interventions	Alcohol‐based hand sanitiser (62% ethyl alcohol in a gel base) in a squeeze bottle and TECNOL procedure masks with ear loops (KC Ltd) and educational material or masks and educational material or no intervention. Compliance was encouraged within halls and outside. Sleep wearing was optional. All participants received basic video‐linked instruction on cough etiquette and hand sanitation. At baseline and weekly during the study, participants were asked to fill in a web‐based survey collecting demographic and ILI symptom data. This was supplemented by direct observation of compliance by staff. Compliance with “optimal handwashing” (at least 20 seconds 5 or more times a day) was significantly higher in the sanitiser‐and‐mask arm. See Table 4 for details.	
Outcomes	Laboratory details are described in appendix. Effectiveness: ILI, defined as cough and at least 1 constitutional symptom (fever/feverishness, chills, headache, myalgia). ILI cases were given contact nurses' phone numbers to record the illness and paid USD 25 to provide a throat swab. 368 participants had ILI, and 94 of these had a throat swab analysed by PCR. 10 of these were positive for influenza (7 for A and 3 for B). Safety: N/A	
Notes	The authors conclude that “These findings suggest that face masks and hand hygiene may reduce respiratory illnesses in shared living settings and mitigate the impact of the influenza A (H1N1) pandemic”. This conclusion is based on a significantly lower level of ILI incidence in the mask and hand sanitiser arm compared to the other 2 arms after adjustment for covariates (30% to 50% less in arm 1 compared to controls in the last 2 weeks of the study). Comparison with the ILI rate of the control arm may not be a reflection of the underlying rate of ILI because the intervention arm received instruction on hand sanitation and hand etiquette. The play of adjustments is unclear. The intracluster correlation coefficient is reported in the footer of Table 4. Its very small size suggests lack of clustering within halls. The role of spring break is mentioned in the Discussion, as are the results of this study compared to other studies included in our review (Cowling 2008 and MacIntyre 2009). The authors report that 147 of 1297 participants (11.3%) had ILI symptoms “at baseline” and were excluded from analysis. During the 6 weeks of the study, 368 of 1150 participants (32%) had ILI. This averages out at about 5% per week. It is unclear what the term “at baseline” means; presumably this means during the 2 to 3 weeks of participant enrolment. If this is so, the reason for the triggering of the interventions (tied to influenza isolation) are obscure, as the trial is supposedly about ILI, and an ILI outbreak was already under way “at baseline”. This study has the same trial registration number as the Aiello 2012 study; the study was funded by government and pharmaceutical industry, i.e. this work was supported by funding from the Centers for Disease Control (CDC) and Prevention Grant U01 C1000441 (www.cdc.gov). Disclosure of interest: none declared.	
***Risk of bias***		
**Bias**	**Authors' judgement**	**Support for judgement**
Random sequence generation (selection bias)	Unclear risk	Described as randomised, but sequence generation not reported
Allocation concealment (selection bias)	High risk	The residence hall units were randomised by blindly selecting a uniform ticket with the name of each hall out of a container (A.S.M. and A.A.) for randomisation assignment to each study arm.
Blinding of participants and personnel (performance bias) All outcomes	High risk	Unblinded study
Blinding of outcome assessment (detection bias) All outcomes	Low risk	Outcome assessors blinded.
Incomplete outcome data (attrition bias) All outcomes	Low risk	Attrition is reported as follows: 9, 11, and 19 ineligible and 26, 52, and 21 lost to follow‐up (respectively by arm), for a total of 39 and 99 for each reason for attrition. In total, 1297 (97%) of 1331 participants completed a baseline and at least 1‐weekly survey. The text reports an ITT analysis with only 1 ILI episode included by participant. No reasons for the attrition of participants and swab volunteers are reported (were the swabs taken from a random sample or not?).
Selective reporting (reporting bias)	High risk	There is no information on the causes of ILI other than the reporting on the 10 influenza PCR‐positive swabs of 94 out of 368 students with ILI. This is a very low rate (and the Discussion confirms that the influenza season was mild), but investigation of the other known causes of ILI is not even mentioned in the text. This is especially important because stress, alcohol intake levels, and influenza vaccination were a significant predictor of ILI symptoms (Table 1). The reason for selective testing and/or reporting of influenza viruses tests over the other causes of ILI are unclear, especially as the study objective was focused on ILI. The text is also difficult to follow, weaving the reporting of ILI and influenza without a clear rationale.

**Table:** Aiello 2012

***Study characteristics***		
Methods	During the 2007 to 2008 influenza season, 1111 students residing in university residence halls were cluster‐randomised by residence house (N = 37) to either face mask and hand hygiene, face mask only, or control arms. Discrete time survival analysis using generalised models estimated rate ratios according to study arm, each week and cumulatively over the 6‐week intervention period, for clinically verified ILI and laboratory‐confirmed influenza A or B.	
Participants	A total of 1187 young adults living in 37 residence halls, randomly assigned to 1 of 3 groups for 6 weeks: face mask use (n = 392), face masks with hand hygiene (n = 349), control (n = 370) Inclusion criteria: aged 18 or more, willing to wear mask and use alcohol‐based hand sanitiser, have a throat swab specimen collected when ill, and complete the baseline and weekly surveys over the 6‐week study period Exclusion criteria: individuals reporting a skin allergy to alcohol were excluded	
Interventions	Participants were assigned to face mask and hand hygiene, face mask only, or control group during the study. See Table 4 for details.	
Outcomes	Clinically verified ILI: case definition (presence of cough and at least 1 or more of fever/feverishness, chills, or body aches) Laboratory‐confirmed influenza A or B. Throat swab specimens were tested for influenza A or B using RT‐PCR. No safety outcomes reported.	
Notes	This study has the same trial registration number as the Aiello 2010 study; the study was funded by government and pharmaceutical industry, i.e. this work was supported by funding from the Centers for Disease Control (CDC) and Prevention Grant U01 C1000441 (www.cdc.gov). Disclosure of interest: none declared.	
***Risk of bias***		
**Bias**	**Authors' judgement**	**Support for judgement**
Random sequence generation (selection bias)	Low risk	Computer generation of sequence described.
Allocation concealment (selection bias)	Low risk	All residence houses in each of the residence halls were randomised prior to the intervention implementation.
Blinding of participants and personnel (performance bias) All outcomes	High risk	No blinding for study participants and personnel
Blinding of outcome assessment (detection bias) All outcomes	Low risk	Outcome assessors blinded.
Incomplete outcome data (attrition bias) All outcomes	Low risk	Attrition low and similar in each group
Selective reporting (reporting bias)	Low risk	2 outcomes specified and reported.

**Table:** Alfelali 2020

***Study characteristics***		
Methods	Cluster open‐label RCT Location: Mina, Greater Makkah, Saudi Arabia Follow up for 4 days	
Participants	Arabic or English speaking Hajj pilgrims aged > 18 years from participating countries (Australia, Qatar and KSA) staying in allocated tents and able to provide signed informed consent were included.	
Interventions	Mask wearing. See Table 4 for details.	
Outcomes	Effectiveness: Laboratory: laboratory‐confirmed viral respiratory infections (nasal swab on 650 participants only) Secondary outcomes: clinical respiratory infections in participants Safety reported on side effects of mask wearing The most common side effects: difficulty in breathing (26.2%); discomfort (22%); a small minority (3%) reported feeling hot, sweating, a bad smell or blurred vision with eyeglasses	
Notes	The authors conclude that this trial was unable to provide conclusive evidence on facemask efficacy against viral respiratory infections most likely due to poor adherence to protocol. Funding: this report was made possible by a National Priorities Research Program grant (NPRP 6‐ 1505‐3‐358) from the Qatar National Research Fund, a member of Qatar Foundation. Disclosure of interests: the other authors have no competing interests to declare.	
***Risk of bias***		
**Bias**	**Authors' judgement**	**Support for judgement**
Random sequence generation (selection bias)	Low risk	Coin‐tossing by an individual who was not a member of the research team
Allocation concealment (selection bias)	High risk	Used coin tossing which can introduce imbalance
Blinding of participants and personnel (performance bias) All outcomes	High risk	No blinding
Blinding of outcome assessment (detection bias) All outcomes	Low risk	Laboratory staff were blinded to the assigned intervention group
Incomplete outcome data (attrition bias) All outcomes	Low risk	Reported both intention‐to‐treat and per‐protocol analysis and participant flow chart
Selective reporting (reporting bias)	Unclear risk	Insufficient information available.

**Table:** Almanza‐Reyes 2021

***Study characteristics***		
Methods	RCT randomised using a computer‐generated block scheme and stratified according to duty position, work shifts and the area/department of the service FU duration: 9 weeks	
Participants	Workers (doctors, nurses, administrators) in a hospital for the exclusive recruitment of patients diagnosed with COVID‐19 “General Tijuana Hospital”	
Interventions	Experimental group: mouthwash and nose rinse Silver mouth wash: 50 mL spray bottle containing AgNPs solution with 1 wt% concentration (0.6 mg/mLmetallic silver). Mix 4 to 6 spray shots (corresponding to volume ~ 0.5 mL) of this solution with 20 mL of water and to gargle with obtained solution for 15 to 30 seconds at least 3 times a day. Or use as nasal lavages on the inner part of the nasal alae and nasal passage with the same solution using a cotton swab twice a day. Mouth spray: cover evenly the oral cavity with the direct 1 to 2 spray shots of solution without its previous dilution in water. Control group: instructed to do mouth wash and nose rinse with a conventional mouthwash the way they normally did before the study See Table 4 for details.	
Outcomes	Effectiveness: Laboratory: Lab‐confirmed infection using RT‐PCR; symptoms of respiratory tract infection (RTI) no definition given; clinical Evacuation: CT (Toshiba Aquilion 16, Japan) chest scan (random selection) Safety: done using self‐reported by participants using a questionnaire. “The present study also showed that no harmful side effects were observed in the 114 participants who used AgNPs as a mouthwash and nose rinse solution for 9 weeks”	
Notes	Authors conclude that the mouth and nasal rinse with AgNPs helps in the prevention of SARS‐CoV‐2 infection in health personnel who are exposed to patients diagnosed with COVID‐19. Funding: Funded studies A. Pestryakov Development Program "Priority 2030" Tomsk Polytechnic University https://tpu.ru/en. Conflict of interest statement: the authors have declared that no competing interests exist.	
***Risk of bias***		
**Bias**	**Authors' judgement**	**Support for judgement**
Random sequence generation (selection bias)	Low risk	Computer‐generated stratified block scheme
Allocation concealment (selection bias)	High risk	Unbalanced baseline prognostic factors (vaccination and frequency of handwashing)
Blinding of participants and personnel (performance bias) All outcomes	High risk	Not blinded.
Blinding of outcome assessment (detection bias) All outcomes	Unclear risk	Insufficient information provided.
Incomplete outcome data (attrition bias) All outcomes	Unclear risk	No participant flow chart reported.
Selective reporting (reporting bias)	Unclear risk	No protocol available

**Table:** Alzaher 2018

***Study characteristics***		
Methods	A cluster‐RCT conducted amongst girls attending 4 primary schools between January and March 2018. The participants attended a hand‐hygiene workshop. The schoolgirls’ absences were followed up for 5 weeks. Incidence rate, percentage of absence days, and absence rate were calculated for total and upper respiratory infections absences.	
Participants	A total of 496 schoolgirls aged of 6 to 12 years, attending 4 public primary girls’ schools in the city of Riyadh, Saudi Arabia between January and March 2018. Students were randomised to education group (n = 234) or control group (n = 262). Exclusion criteria: not reported	
Interventions	Hand hygiene workshop. See Table 4 for details.	
Outcomes	Incidence rate, percentage of absence days, and absence rate were calculated for total and upper respiratory infections absences. The episode of URIs was defined as having 2 of the following symptoms for a day or 1 of the symptoms for 2 or more consecutive days: 1) a runny nose, 2) a stuffy or blocked nose or noisy breathing, 3) sneezing, 4) a cough, 5) a sore throat, and 6) feeling hot, having a fever or a chill. No safety outcomes reported.	
Notes	Source of funding is unclear. Disclosure of interest: none mentioned.	
***Risk of bias***		
**Bias**	**Authors' judgement**	**Support for judgement**
Random sequence generation (selection bias)	Unclear risk	Insufficient detail provided.
Allocation concealment (selection bias)	Low risk	Schools allocated prior to all schoolgirls attending selected schools were invited to participate.
Blinding of participants and personnel (performance bias) All outcomes	High risk	Unblinded study
Blinding of outcome assessment (detection bias) All outcomes	High risk	Unblinded study
Incomplete outcome data (attrition bias) All outcomes	Low risk	No loss to follow‐up
Selective reporting (reporting bias)	Unclear risk	No protocol available

**Table:** Arbogast 2016

***Study characteristics***		
Methods	A 13.5‐month prospective cluster‐RCT executed with alcohol‐based hand sanitiser in strategic workplace locations and personal use (intervention group) and brief hand hygiene education (both groups). Four years of retrospective data were collected for all participants.	
Participants	Data for a total of 1183 participants were analysed (intervention group = 525, control group = 607). Inclusion criteria: all employees at 3 facilities who were 18 years of age or older, were enrolled in the company health insurance coverage, did not transfer between sites, and worked onsite full time (≥ 32 hours) were eligible for the study Exclusion criteria: not reported	
Interventions	Alcohol‐based hand sanitiser in strategic workplace locations and personal use (intervention group) and brief hand hygiene education (both groups). See Table 4 for details.	
Outcomes	The number of healthcare insurance claims, for a defined set of preventable illnesses, per participant per year Absenteeism, defined as the number of sick episodes per participant per year Claims based on ICD‐9 codes No safety outcomes reported.	
Notes	Only 2 clusters (1 per group) included, hence study data not included in meta‐analysis. Industry funded. Disclosure of interest: none mentioned.	
***Risk of bias***		
**Bias**	**Authors' judgement**	**Support for judgement**
Random sequence generation (selection bias)	Unclear risk	No details provided.
Allocation concealment (selection bias)	Unclear risk	No details provided.
Blinding of participants and personnel (performance bias) All outcomes	High risk	Unblinded study
Blinding of outcome assessment (detection bias) All outcomes	High risk	Unblinded study
Incomplete outcome data (attrition bias) All outcomes	Low risk	Attrition minimal and similar in 2 groups
Selective reporting (reporting bias)	Unclear risk	No protocol available

**Table:** Ashraf 2020

***Study characteristics***		
Methods	Geographically pair‐matched community‐based cluster‐randomised trial Used a random number generator to block Open‐label Block randomised: unit of randomisation was a group of compounds visited by a single local promoter	
Participants	1. Infants (target child) will be eligible to participate in the study if: a. they are in utero at the baseline survey. b. their parents/guardians are planning to stay in the study village for the next 12 months (if a mother is planning to give birth at her natal home and then return, she will still be a candidate for enrolment) 2. Children < 36 months old at baseline that are living in the compound of a target child will be eligible to participate in diarrhoea measurement if: a. they are < 36 months old at the baseline survey; b. their parents/guardians are planning to stay in the study village for the next 12 months. 3. Children 18 to 27 months old at baseline that are living in the compound of a target child will be eligible to participate in intestinal parasite specimen collection if: a. they are 18 to 27 months old at the baseline survey; b. their parents/guardians are planning to stay in the study village for the next 12 months.	
Interventions	6 intervention arms: water quality, sanitation, hand washing, combined WSH, nutrition, nutrition + WSH Intervention was delivered at the household or the compound level See Table 4 for details.	
Outcomes	Effectiveness: Primary outcome: 7‐day prevalence of acute respiratory illness (ARI). Defined as: caregiver‐reported symptoms of persistent cough or panting, wheezing, or difficulty breathing (1 or 2) in the 7 days before the interview. No clinical data were collected Secondary analyses: alternate combinations of the measured symptoms: 7‐day prevalence of only panting, wheezing, or difficulty breathing (2) and ARI plus fever ([1 or 2] and 3) Outcomes were measured approximately 12 and 24 months following intervention roll out. Safety not assessed	
Notes	The authors conclude that: single targeted water, sanitation, and hygiene interventions reduced reported respiratory illness in young children. There was no apparent respiratory health benefit from combining WASH interventions. Financial support: this research was funded by Global Development grant OPPGD759 from the Bill & Melinda Gates Foundation to the University of California, Berkeley, CA. S. P. L., S. A., M. I., B. F. A., and J. M. C. report grants from the Bill & Melinda Gates Foundation during the conduct of the study. P. K. R. reports grants from Leland Stanford University during the conduct of the study for support to the WASH Benefits project. M. R. reports grants and non financial support from the Bill & Melinda Gates Foundation (through a subcontract from UC Berkeley) during the conduct of the study. Disclosure of interest: none mentioned.	
***Risk of bias***		
**Bias**	**Authors' judgement**	**Support for judgement**
Random sequence generation (selection bias)	Low risk	Random number generator
Allocation concealment (selection bias)	Low risk	Random allocation by an offsite investigator
Blinding of participants and personnel (performance bias) All outcomes	High risk	Not blinded
Blinding of outcome assessment (detection bias) All outcomes	Low risk	The research team who implemented the intervention was separate from the data collection team. The analysis was carried out masked to the allocated group.
Incomplete outcome data (attrition bias) All outcomes	Low risk	Provided participants flow diagram showing minimal attrition.
Selective reporting (reporting bias)	Low risk	Reported the pre‐specified outcomes.

**Table:** Azor‐Martinez 2016

***Study characteristics***		
Methods	Randomised, controlled, and open study with an 8‐month follow‐up. The experimental group washed their hands with soap and water, together with using hand sanitiser, and the control group followed their usual handwashing procedures. Absenteeism rates due to URIs were compared between the 2 groups through a multivariate Poisson regression analysis. The per cent of days missed in both groups were compared with a z test.	
Participants	A sample of 1341 (intervention group = 621, control group = 720) Inclusion criteria: children 4 to 12 years old, attending 5 state schools in Almerıa (Spain) whose parents/guardians had signed an informed consent document Exclusion criteria: children who had any of the following chronic illnesses that predisposed them to infection: neoplasia, primary and secondary immunodeficiencies, cystic fibrosis, chronic treatment with high doses of steroids or immunosuppressants	
Interventions	Hand‐washing workshops of 2‐hour duration. The experimental group washed their hands with soap and water together with using hand sanitiser, whilst the control group followed usual hand‐washing procedures. See Table 4 for details.	
Outcomes	Absenteeism rates due to URIs Per cent of days missed Respiratory illness was defined by 2 of the following symptoms during 1 day, or 1 of the symptoms for 2 consecutive days: (1) runny nose; (2) stuffy or blocked nose or noisy breathing; (3) cough; (4) feeling hot or feverish or having chills; (5) sore throat; or (6) sneezing. A school absenteeism case (episode) was defined as when a child failed to attend school due to an URI. Common infectious illnesses, such as conjunctivitis, and skin infections were not included. Other causes for absenteeism, such as doctors’ appointments, family vacations, and accident injuries, were also excluded. No safety outcomes reported.	
Notes	Government funded Disclosure of interest: none mentioned.	
***Risk of bias***		
**Bias**	**Authors' judgement**	**Support for judgement**
Random sequence generation (selection bias)	Low risk	A random number table was used.
Allocation concealment (selection bias)	Low risk	Schools/classes allocated prior to children recruited.
Blinding of participants and personnel (performance bias) All outcomes	High risk	Unblinded study
Blinding of outcome assessment (detection bias) All outcomes	High risk	Unblinded study
Incomplete outcome data (attrition bias) All outcomes	High risk	Attrition levels high and different in the 2 groups
Selective reporting (reporting bias)	Unclear risk	No protocol available

**Table:** Azor‐Martinez 2018

***Study characteristics***		
Methods	A cluster‐RCT, controlled, and open study of 911 children aged 0 to 3 years attending 24 DCCs in Almería, Spain, with an 8‐month follow‐up. 2 intervention groups of DCC families performed educational and hand hygiene measures, 1 with soap and water (n = 274), another with hand sanitiser (n = 339), and the control group followed usual hand‐washing procedures (n = 298). Respiratory infection (RI) episode rates were compared through multilevel Poisson regression models. The percentage of days missed were compared with Poisson exact tests.	
Participants	A total of 911 children attending 24 DCCs in Almería (Spain). Inclusion criteria: children between 0 and 3 years old enrolled in DCCs and attending for at least 15 hours per week whose parents or guardians had signed an informed consent Exclusion criteria: children with chronic illness or medication that could affect their likelihood of contracting an infection Data were analysed for 911 participants: hand sanitiser group (n = 339), soap and water group (n = 274), and control group (n = 298).	
Interventions	2 intervention groups. 1 group used soap and water, another used hand sanitiser, whilst the control group followed usual hand‐washing procedures. Groups received 1‐hour hand hygiene workshop. See Table 4 for details.	
Outcomes	Primary: RI incidence rate Secondary: (1) the presence or absence of at least 1 antibiotic prescription for each new RI episode during the study period (topical antibiotics were excluded), and (2) the percentage of RI absenteeism days in the 3 groups calculated as the ratio of RI absenteeism days to all possible days of attendance DCC absenteeism episode was defined as when a child failed to attend a DCC because of an RI. Respiratory illness was defined as the presence of 2 of the following symptoms during 1 day or the presence of 1 of the symptoms for 2 consecutive days‍: (1) runny nose, (2) stuffy or blocked nose or noisy breathing, (3) cough, (4) feeling hot or feverish or having chills, (5) sore throat, or (6) sneezing. No safety outcomes reported.	
Notes	Government funded. This work was supported by a grant from the Andalusia Department of Health. Competing interests: the authors have indicated they have no potential conflicts of interest to disclose.	
***Risk of bias***		
**Bias**	**Authors' judgement**	**Support for judgement**
Random sequence generation (selection bias)	Low risk	Computer randomisation using statistical software for the sequence
Allocation concealment (selection bias)	Low risk	Clusters assigned prior to recruitment.
Blinding of participants and personnel (performance bias) All outcomes	High risk	Unblinded study
Blinding of outcome assessment (detection bias) All outcomes	High risk	Unblinded study
Incomplete outcome data (attrition bias) All outcomes	Low risk	Attrition minimal and similar in 3 groups
Selective reporting (reporting bias)	Unclear risk	No protocol available

**Table:** Ban 2015

***Study characteristics***		
Methods	Quote: "Group randomised" trial. Only 2 clusters, which were 2 kindergartens in Xiantao City, Hubei Province, China.	
Participants	Data for a total of 393 participants were analysed (intervention group = 194, control group = 199). 5 classes (221 children) randomly selected from 1 kindergarten in the intervention group and 6 classes (244 children) randomly selected from another kindergarten in the control group. Children were aged 5 or under. There were 72 exclusions from the analysis.	
Interventions	Intervention group: hand hygiene and surface‐cleaning education and provision of products for kindergarten and home use. Control group: usual practice. See Table 4 for details.	
Outcomes	Respiratory illness, defined as: 2 or more of the following: fever, cough and expectoration, runny nose and nasal congestion, collected by parental questionnaire. Axillary temperature higher than 37.3 °C or the range of temperature fluctuation is more than 1 °C. 'Cough and expectoration' were defined as 3 or more coughs in a single hour and lasting for 4 or more hours in a single day, with or without expectoration. 'Runny nose and nasal congestion' were defined as a runny nose lasting for 4 or more hours in 1 day, with or without nasal congestion.	
Notes	Funding not mentioned. Disclosure of interest: none mentioned.	
***Risk of bias***		
**Bias**	**Authors' judgement**	**Support for judgement**
Random sequence generation (selection bias)	High risk	Method not described, and only 2 clusters.
Allocation concealment (selection bias)	Unclear risk	Method not described.
Blinding of participants and personnel (performance bias) All outcomes	High risk	Unblinded study
Blinding of outcome assessment (detection bias) All outcomes	High risk	Unblinded study
Incomplete outcome data (attrition bias) All outcomes	High risk	Parental report, and parents were aware of treatment allocation
Selective reporting (reporting bias)	High risk	Attrition reported and balanced between groups, but high rate of attrition in a trial with small numbers of participants.

**Table:** Barasheed 2014

***Study characteristics***		
Methods	Pilot, non‐blinded, parallel, cluster‐RCT	
Participants	22 tents were randomly selected from the Australian pilgrims camped in Mina, during Hajj in 2011; 12 tents were allocated to the mask group and 10 tents to the control group. A total of 164 Australian pilgrims were recruited: 75 in the mask group (39 ‘cases’ and 36 ‘contacts’) and 89 in the control group (36 ‘cases’ and 53 ‘contacts’). Inclusion criteria for index case: 1) Australian pilgrims of any gender aged > 15 years who attend the Hajj 2011, and 2) have symptoms of respiratory infection for 3 days. For close tent contact: 1) Australian pilgrims of any gender aged 15 years or more who attend the Hajj 2011, and 2) pilgrims who share the same tent and sleep "immediately close" to the index case. Exclusion criteria: for index case: 1) pilgrims who do not suffer from symptoms of respiratory infection, 2) pilgrims who present with symptoms of respiratory infection for > 3 days, and 3) children aged less than 15 years. For close tent contact: 1) pilgrims who are symptomatic at presentation, 2) pilgrims who are not close tent contacts of an index case, and 3) children aged less than 15 years. Only 10% to 15% of potential participants took part in the study.	
Interventions	"supervised mask use" versus "no supervised mask use". See Table 4 for details.	
Outcomes	Laboratory: 2 nasal swabs from all ILI cases and contacts, 1 for influenza POCT using the QuickVue Influenza (A+B) assay (Quidel Corporation, San Diego, USA) and 1 for later nucleic acid testing for influenza and other respiratory viruses. However, there was a problem with getting POCT on time during Hajj. Effectiveness: to assess the effectiveness of face masks in the prevention of transmission of ILI. ILI was defined as subjective (or proven) fever plus 1 respiratory symptom (e.g. dry or productive cough, runny nose, sore throat, shortness of breath). Safety: none planned or reported	
Notes	The study was conducted from 4 November 2011 to 10 November 2011. Compliance with face mask use by pilgrims was 56 of 75 (76%) in the mask group and 11 of 89 (12%) in the control group (P < 0.001). The proportion of face mask user in the ‘mask’ tents was 76% for both males (19/25) and females (38/50). The most often reported reason for not wearing face masks was discomfort (15%). Government funded: Qatar National Research Fund (QNRF). The other authors have declared no conflict of interest in relation to this work.	
***Risk of bias***		
**Bias**	**Authors' judgement**	**Support for judgement**
Random sequence generation (selection bias)	Unclear risk	Insufficient information provided.
Allocation concealment (selection bias)	Unclear risk	Quote: "tents were randomised to either intervention group (supervised mask tent) or control group (no supervised mask tent) by an independent study coordinator who was not an investigator", but did not mention how
Blinding of participants and personnel (performance bias) All outcomes	Low risk	Quote: "Because advice from the Saudi Ministry of Hajj to all pilgrims included recommending the wearing of masks, all pilgrims, both cases and controls, were asked about mask‐wearing"
Blinding of outcome assessment (detection bias) All outcomes	Unclear risk	Self‐reported outcomes (nasal swab was performed for those who reported ILI symptoms and was not intended as systematic detection). ILI was defined as subjective (or proven) fever plus 1 respiratory symptom.
Incomplete outcome data (attrition bias) All outcomes	Low risk	No loss to follow‐up, all numbers were reported from enrolment to analysis
Selective reporting (reporting bias)	Low risk	All planned outcomes were reported.

**Table:** Biswas 2019

***Study characteristics***		
Methods	Cluster‐RCT in 24 primary schools in Dhaka to assess the effectiveness of hand sanitiser and a respiratory hygiene education intervention in reducing ILI and laboratory‐confirmed influenza during June to September 2015. 12 schools were randomly selected to receive hand sanitiser and respiratory hygiene education, and 12 schools received no intervention. Field staff actively followed children daily to monitor for new ILI episodes (cough with fever) through school visits and by phone if a child was absent. When an illness episode was identified, medical technologists collected nasal swabs to test for influenza viruses.	
Participants	A total of 10,855 students were enrolled in the study (intervention schools = 5077 children; control schools = 5778 children). Children aged 5 to 10 years educated in 24 randomly selected primary schools in Dhaka, Bangladesh Exclusion: schools that offered education above grade 5 because of differences in student populations, as well as schools that had previously received a hand or respiratory hygiene intervention	
Interventions	Hand sanitiser and respiratory hygiene education versus no intervention. See Table 4 for details.	
Outcomes	Incidence of ILI Incidence of laboratory‐confirmed influenza (RT‐PCR) An ILI episode was defined as measured fever ≥ 38 °C or subjective fever and cough. If a child was absent, the field staff followed up by phone to identify the reason for absenteeism and to determine if the child met the ILI case definition. If a child in a participating school had an ILI episode, a trained medical technologist visited the child’s household to obtain consent from the child’s parent/guardian and collect a nasal swab from the child within 48 hours of symptom onset. If it was outside the 48‐hour window, the sample was not collected. No safety outcomes reported.	
Notes	Government funded. Disclosure of interest: none mentioned.	
***Risk of bias***		
**Bias**	**Authors' judgement**	**Support for judgement**
Random sequence generation (selection bias)	Low risk	Sequence generated using a computer‐based random number generator.
Allocation concealment (selection bias)	Low risk	Allocation completed prior to individuals being recruited.
Blinding of participants and personnel (performance bias) All outcomes	High risk	Unblinded study
Blinding of outcome assessment (detection bias) All outcomes	High risk	Unblinded study
Incomplete outcome data (attrition bias) All outcomes	High risk	Information missing for 30 children (28 children in the control schools and 2 children in the intervention schools)
Selective reporting (reporting bias)	Unclear risk	No protocol available

**Table:** Bundgaard 2021

***Study characteristics***		
Methods	Investigator‐initiated, nationwide, unblinded, randomised controlled trial stratified by the 5 regions of Denmark	
Participants	Inclusion criteria: community‐dwelling adults aged 18 years or older without current or prior symptoms or diagnosis of COVID‐19 reported being outside the home amongst others for at least 3 hours per day, and who did not wear masks during their daily work. Exclusion criteria: previously tested positive for SARS‐CoV‐2 and wear face masks at work	
Interventions	Exposure: mask (N = 2392) Control group: no mask (N = 2470) Both groups received materials and instructions for antibody testing on receipt and at 1 month; materials and instructions for collecting an oropharyngeal/nasal swab sample for polymerase chain reaction (PCR) testing at 1 month and whenever symptoms compatible with COVID‐19 occurred during follow‐up. They registered symptoms and results of the antibody test in the online REDCap system. Written instructions and instructional videos guided antibody testing, oropharyngeal/nasal swabbing, and proper use of masks, and a help line was available to participants. See Table 4 for details.	
Outcomes	Study duration: 1 month Effectiveness: primary outcome (composite) SARS‐CoV‐2 infection, defined as a positive result on an oropharyngeal/nasal swab test for SARS‐CoV‐2, development of a positive SARS‐CoV‐2 antibody test result (IgM or IgG) during the study period, or a hospital‐based diagnosis of SARS‐CoV‐2 infection or COVID‐19. Secondary outcome: PCR evidence of infection with other respiratory viruses Safety: adverse reaction: 14% in mask group (no further descriptions)	
Notes	The authors conclude that inconclusive results, missing data, variable adherence, patient‐reported findings on home tests, no blinding, and no assessment of whether masks could decrease disease transmission from mask wearers to others. Funding: the primary funding source was The Salling Foundations. Disclsure can be viewed at www.acponline.org /authors/icmje/ConflictOfInterestForms.do?msNum=M20‐6817.	
***Risk of bias***		
**Bias**	**Authors' judgement**	**Support for judgement**
Random sequence generation (selection bias)	Low risk	Computer algorithm stratified by the 5 regions of Denmark
Allocation concealment (selection bias)	Unclear risk	Insufficient information reported
Blinding of participants and personnel (performance bias) All outcomes	High risk	Not blinded
Blinding of outcome assessment (detection bias) All outcomes	High risk	Not blinded. Patient reported symptoms, POCT testing, patient‐reported findings on home tests.
Incomplete outcome data (attrition bias) All outcomes	Low risk	Participant flow chart showed acceptable attrition
Selective reporting (reporting bias)	Low risk	All pre‐specified outcomes reported.

**Table:** Canini 2010

***Study characteristics***		
Methods	A cluster‐RCT conducted in France during the 2008 to 2009 influenza season. Households were recruited during a medical visit of a household member with a positive rapid influenza A test and symptoms lasting less than 48 hours. Households were randomised either to the mask or control group for 7 days. In the intervention arm, the index case had to wear a surgical mask from the medical visit and for a period of 5 days. The trial was initially intended to include 372 households, but was prematurely interrupted after the inclusion of 105 households (306 contacts) following the advice of an independent steering committee. Generalised estimating equations were used to test the association between the intervention and the proportion of household contacts who developed an ILI during the 7 days following the inclusion.	
Participants	A total of 105 households were randomised, which represented 148 contacts in the intervention arm and 158 in the control arm. The study was conducted in 3 French regions (Ile de France, Aquitaine, and Franche‐Comté) and included households of size 3 to 8. Exclusion criteria: if index patient was treated for asthma or chronic obstructive pulmonary disease or was hospitalised	
Interventions	Surgical mask versus no mask. See Table 4 for details.	
Outcomes	The primary endpoint was the proportion of household contacts who developed an ILI during the 7 days following inclusion. Exploratory cluster‐level efficacy outcome, the proportion of households with 1 or more secondary illness in household contacts. A temperature over 37.8 °C with cough or sore throat was used as primary clinical case definition. Adverse reactions due to mask‐wearing	
Notes	Government funded. Competing interests: the authors have declared that no competing interests exist.	
***Risk of bias***		
**Bias**	**Authors' judgement**	**Support for judgement**
Random sequence generation (selection bias)	Low risk	Randomisation lists were generated by a computerised program.
Allocation concealment (selection bias)	Low risk	Randomisation was performed centrally by the GP after written consent on an interactive voice response system dedicated to the study.
Blinding of participants and personnel (performance bias) All outcomes	High risk	Unblinded study
Blinding of outcome assessment (detection bias) All outcomes	Low risk	Outcome assessors blinded.
Incomplete outcome data (attrition bias) All outcomes	Low risk	All households included in analysis.
Selective reporting (reporting bias)	Low risk	All specified outcomes reported.

**Table:** Carabin 1999

***Study characteristics***		
Methods	Cluster‐RCT carried out in DCCs in the Canadian province of Quebec between 1 September 1996 and 30 November 1997 (15 months). The aim was to test the effects of a hygiene programme on the incidence of diarrhoea and fecal contamination (data not extracted) and on colds and URTIs. The design included before and after periods analysed to assess the Hawthorne effect of study participation on control DCCs. The unit of randomisation was DCC, but analysis was also carried out at classroom and single‐child level. This is a common mistake in cluster‐RCT analysis. DCCs were stratified by URTI incidence preceding the trial and randomised by location. Cluster coefficients are not reported.	
Participants	A total of 1729 children aged 18 to 36 months in 47 DCCs (83 toddler classrooms) Inclusion criteria: presence of at least 1 sandbox and 1 play area and of at least 12 available toddler places For the autumn of 1997 intervention group (24 DCCs, 43 classrooms, and 414 children), control group (23 DCCs, 23 classrooms, and 374 children). It is not clear what is the distribution and data for the autumn of 1996.	
Interventions	Training session (1 day) with washing of hands, toy cleaning, window opening, sand pit cleaning, and repeated exhortations to hand wash. See Table 4 for details.	
Outcomes	Laboratory: N/A Effectiveness: diarrhoea and coliform contamination (data not extracted) Colds (nasal discharge with at least 1 of the following: fever, sneezing, cough, sore throat, earache, malaise, irritability) URTI (cold of at least 2 days' duration) Surveillance was carried out by educators, annotating absences or illness on calendars. Researchers also filled in a phone questionnaire with answers by DCC directors. Safety: N/A	
Notes	Risk of bias: high (no description of randomisation; partial reporting of outcomes, numerators, and denominators) Notes: the authors conclude that the intervention reduced the incidence of colds (IRR 0.80, 95% CI 0.68 to 0.93). This was a confusingly written study with unclear interweaving of 2 study designs. For unclear reasons analysis was only carried out for the first autumn. Unclear why colds are not reported in the results. Cluster‐coefficients and randomisation process were not described. Support for the study was provided by the Rhone‐Poulenc Rorer Canada Ltd. Disclosure of interest: none mentioned.	
***Risk of bias***		
**Bias**	**Authors' judgement**	**Support for judgement**
Random sequence generation (selection bias)	Unclear risk	Block randomisation of DCC according to region, but sequence generation not reported
Allocation concealment (selection bias)	Unclear risk	Not described
Blinding of participants and personnel (performance bias) All outcomes	High risk	Blinding not possible (hygiene session plus educational material versus none)
Blinding of outcome assessment (detection bias) All outcomes	High risk	Unblinded
Incomplete outcome data (attrition bias) All outcomes	High risk	Originally 52 eligible DCCs with 89 classrooms agreed to take part, but 5 dropped out (2 closed, 1 was sold, 2 either did not provide data or the data were "unreliable", and 6 classrooms had insufficient data). 43 children failing to attend DCC for at least 5 days in the autumn were also excluded. ITT analysis was carried out including an additional DCC whose director refused to let staff attend the training session. No correction made for clustering.
Selective reporting (reporting bias)	High risk	Denominators unclear and not explained

**Table:** Chard 2019

***Study characteristics***		
Methods	Cluster‐RCT conducted amongst 100 randomly selected primary schools lacking functional WASH facilities in Saravane Province, Lao People's Democratic Republic. Schools were randomly assigned to either the intervention (n = 50) or comparison (n = 50) arm. Intervention schools received a school water supply, sanitation facilities, hand‐washing facilities, drinking water filters, and behaviour change education and promotion. Comparison schools received the intervention after research activities had ended. At unannounced visits every 6 to 8 weeks, enumerators recorded pupils’ roll‐call absence, enrolment, attrition, progression to the next grade, and reported illness (diarrhoea, respiratory infection, conjunctivitis), and conducted structured observations to measure intervention fidelity and adherence. Stool samples were collected annually prior to de‐worming and analysed for soil‐transmitted helminth (STH) infection. In addition to our primary ITT analysis, we conducted secondary analyses to quantify the role of intervention fidelity and adherence on project impacts.	
Participants	100 primary schools (50 intervention, 50 comparison) with a total of 3993 pupils were enrolled throughout the study period (intervention schools = 2021 pupils, control schools = 1972 pupils). Up to 40 pupils selected from grades 3 to 5 in each school using systematic stratified sampling, with grade and sex as the stratification variables. Pupils selected at baseline were followed throughout the entire study period; pupils who left the school due to abandonment or transfer were replaced at the beginning of the following academic year, maintaining equal grade and sex ratios when possible. Pupils who progressed from fifth to the sixth grade were replaced with pupils from grade 3 the following academic year.	
Interventions	Water supply, sanitation facilities, hand‐washing facilities, drinking water filters, and behaviour change education and promotion versus control. See Table 4 for details.	
Outcomes	Primary impact of interest was pupil absence, measured by school‐wide roll‐call at each visit. Secondary health impacts included diarrhoea, symptoms of respiratory infection, and conjunctivitis/non‐vision‐related eye illness collected through pupil interviews. Pupils were considered to have symptoms of respiratory infection if they reported cough, runny nose, stuffy nose, or sore throat. No safety outcomes reported.	
Notes	Funded by government and pharmaceutical industry. Competing interests: all authors have completed the ICMJE uniform disclosure form at http://www.icmje.org/ coi_disclosure.pdf (available upon request from the corresponding author) and declare no conflicts of interest.	
***Risk of bias***		
**Bias**	**Authors' judgement**	**Support for judgement**
Random sequence generation (selection bias)	Unclear risk	Insufficient details provided.
Allocation concealment (selection bias)	Low risk	Schools allocated prior to recruitment of individuals.
Blinding of participants and personnel (performance bias) All outcomes	High risk	Unblinded study
Blinding of outcome assessment (detection bias) All outcomes	High risk	Unblinded
Incomplete outcome data (attrition bias) All outcomes	Low risk	Exclusions were due to participants leaving school, hence unlikely to cause bias.
Selective reporting (reporting bias)	Low risk	All specified outcomes reported.

**Table:** Correa 2012

***Study characteristics***		
Methods	Cluster‐RCT in childcare facilities in Colombia from 16 April to 18 December 2008 (3 school terms) testing the effects of hand hygiene using an alcohol‐based hand rub versus standard practice	
Participants	42 childcare facilities in 6 towns in Colombia. A total of 1727 were enrolled (intervention group = 794 from 21 centres, control group = 933 from 21 centres). Inclusion criteria: licensed to care for 12 or more children aged 1 to 5 years for 8 hours a day, 5 times per week, and where availability of tap water was limited	
Interventions	Intervention: alcohol‐based hand wash as an addition to hand‐washing Control: usual hand‐washing practice See Table 4 for details.	
Outcomes	ARI defined as: 2 or more of the following symptoms for at least 24 hours, lasting at least 2 days: runny, stuffy, or blocked nose or noisy breathing; cough; fever, hot sensation, or chills; and/or sore throat. Ear pain alone was considered an ARI.	
Notes	This work was supported by a grant from the Global Development Network (New Delhi, India), "Fifth Global Research Project: Promoting Innovative Programs from the Developing World: Towards Realizing the Health MDG's in Africa and Asia," and the Bill and Melinda Gates Foundation (Seattle, Washington, United States). Authors declare to have no conflicts of interest.	
***Risk of bias***		
**Bias**	**Authors' judgement**	**Support for judgement**
Random sequence generation (selection bias)	Low risk	"...using the random function in Microsoft Excel™ (Microsoft Corp., Redmond, Washington, United States), random numbers (1 or 2) were generated and allotted 1:1 within each group. Finally, a researcher flipped a coin to decide which number would correspond to either arm (heads = 1, intervention; tails = 2, control)."
Allocation concealment (selection bias)	Unclear risk	Method not described
Blinding of participants and personnel (performance bias) All outcomes	High risk	Unblinded study
Blinding of outcome assessment (detection bias) All outcomes	High risk	Unblinded study
Incomplete outcome data (attrition bias) All outcomes	Low risk	Lost to follow‐up similar in each group and not substantial
Selective reporting (reporting bias)	Unclear risk	No protocol available

**Table:** Cowling 2008

***Study characteristics***		
Methods	Cluster‐RCT carried out in Hong Kong SARS between February and September 2007. The study assessed the effects of non‐pharmaceutical interventions on the household transmission of influenza over a 9‐day period. ILI cases whose family contacts had been symptom‐free for at least 2 weeks rapid‐tested for influenza A and B were used and randomised to 3 interventions. Randomisation was carried out in 2 different schedules (2:1:1 for the first 100 households, and subsequently 8:1:1), but it is unclear why and how this was done.	
Participants	A total of 350 of 944 originally enrolled participants representing 122 households were analysed (control group = 71 households with 205 household contacts, face mask = 21 households with 61 household contacts, HH = 30 households with 84 household contacts). Inclusion criteria: residents of Hong Kong aged at least 2 years, reporting at least 2 symptoms of ILI ( (such as fever ≥ 38 degrees, cough, headache, coryza, sore throat, muscle aches and pains) and positive influenza A+B rapid test and living in a household with at least 2 other individuals, none of whom had ILI in the preceding 14 days Households were excluded because subsequent laboratory testing (culture) was negative. Attrition was not explained.	
Interventions	Households were randomised to either wearing face masks with education (as the control group plus education about face mask use) or hand‐washing with special medicated soap (with alcohol sanitiser) with education (as the control group plus education about hand‐washing) or education about general healthy lifestyle and diet (control group). The soap was distributed in special containers that were weighed at the start and end of the study. Interventions visits to the households were done on average 1 day after randomisation of index case household. See Table 4 for details.	
Outcomes	Laboratory: QuickVue RTI MDCK culture Samples were harvested using NTS, but the text refers to a second procedure from June 2007 onwards testing for non‐influenza viruses, with no data reported. Effectiveness: secondary attack ratios (SAR): SAR is the proportion of household contacts of an index case who were subsequently ill with influenza (symptomatic contact individuals with at least 1 NTS positive for influenza by viral culture or PCR) 3 clinical definitions were used for secondary analysis: Fever ≥ 38 degrees, or at least 2 of following symptoms: headache, coryza, sore throat, muscle aches and pains At least 2 of the following S/S: fever ≥ 37.8 degrees, cough, headache, sore throat, muscle aches and pains Fever ≥ 37.8 degrees plus cough or sore throat Safety: no harms were reported in any of the arms	
Notes	The trial authors conclude that “The secondary attack ratios were lower than anticipated, and lower than reported in other countries, perhaps due to differing patterns of susceptibility, lack of significant antigenic drift in circulating influenza virus strains recently, and/or issues related to the symptomatic recruitment design. Lessons learnt from this pilot have informed changes for the main study in 2008”. Although billed as a pilot study, the text is highly confusing and at times contradictory. The intervention was delivered at a home visit up to 36 hours after the index case was seen in the outpatients. This is a long time and perhaps the reason for failure of the intervention. Practically, the intervention will have to be organised before even seeking medical care, i.e. people know to do it when the child gets sick at home. This work has received financial support from the US Centers for Disease Control and Prevention (grant no. 1 U01 CI000439‐01), the Research Fund for the Control of Infectious Disease, Food and Health Bureau, Government of the Hong Kong SAR, and the Area of Excellence Scheme of the Hong Kong University Grants Committee (grant no. AoE/M‐12/06). Competing Interests: the authors have declared that no competing interests exist.	
***Risk of bias***		
**Bias**	**Authors' judgement**	**Support for judgement**
Random sequence generation (selection bias)	Low risk	Randomisation was computer generated by a biostatistician. Quote:"A pre‐specified table of random numbers will be used to assign one of the three interventions to the household of the index case."
Allocation concealment (selection bias)	Low risk	The households of eligible study index patients were allocated to 3 groups in a 1:1:1 ratio under a block randomisation structure with randomly permuted block sizes of 18, 24, and 30 using a random‐number generator. Allocation was concealed from treating physicians and clinics and implemented by study nurses at the time of the initial household visit.
Blinding of participants and personnel (performance bias) All outcomes	High risk	Participants and people who administered the interventions were not blinded to the interventions, but participants were not informed of the specific nature of the interventions applied to other participating households.
Blinding of outcome assessment (detection bias) All outcomes	High risk	Unblinded
Incomplete outcome data (attrition bias) All outcomes	High risk	Dropout was accounted for. Dropout from the randomised population was high: 32% in control group, 37.5% in hand hygiene group, and 39.4% in face mask and hand hygiene group. Reasons for dropout were distributed evenly across the 3 groups. Authors report follow‐up as proportion of patients remaining in the study after initial dropout.
Selective reporting (reporting bias)	High risk	The choice of season, change in randomisation schedules, and unexplained dropouts amongst contacts; the use of QuickVue, which proved unreliable, reporting bias on non‐influenza isolates resulted in a judgement of high risk of bias.

**Table:** Cowling 2009

***Study characteristics***		
Methods	Cluster‐RCT	
Participants	A total of 407 index cases and 794 household contacts were analysed. Of 407 enrolled households, 322 received the allocated interventions as follows: control group = 112 households with 346 contacts (only 91 households analysed with 279 contacts); hand hygiene = 106 households with 329 contacts (only 85 households analysed with 257 contacts); face mask + hand hygiene = 104 households with 340 contacts (only 83 households analysed with 258 contacts). Inclusion criteria: households in Hong Kong. Index cases from 45 outpatient clinics in both the private and public sectors across Hong Kong. They enrolled individuals who reported at least 2 symptoms of ARI (temperature 37.8 °C, cough, headache, sore throat, or myalgia); had symptom onset within 48 hours; and lived in a household with at least 2 other people, none of whom had reported ARI in the preceding 14 days. After giving informed consent, participants provided nasal and throat swab specimens. 2750 patients were eligible and tested between 2 January and 30 September 2008.	
Interventions	Participants with a positive rapid‐test result and their household contacts were randomly assigned to 1 of 3 study groups: control (lifestyle measures ‐ 134 households), control plus enhanced hand hygiene only (136 households), and control plus face masks and enhanced hand hygiene (137 households) for all household members. No detailed description of the instructions was given to participants. See Table 4 for details.	
Outcomes	Influenza virus infection in household contacts, as confirmed by RT‐PCR or diagnosed clinically after 7 days "The primary outcome measure was the secondary attack ratio at the individual level: that is, the proportion of household contacts infected with influenza virus. We evaluated the secondary attack ratio using a laboratory definition (a household contact with a nose and throat swab specimen positive for influenza by RT‐PCR) as the primary analysis and 2 secondary clinical definitions of influenza based on self‐reported data from the symptom diaries as secondary analyses." Statistical analysis: adjusted for clustering Results: no statistically significant difference in secondary attack ratio between groups in total population. Statistically significant reduction in RT‐PCR confirmed influenza virus infections in the household contacts in 154 households in which the intervention was applied within 36 hours of symptom onset in the index patient. Adherence to hand hygiene was between 44% and 62%. Adherence of index patient to wearing a face mask between 15% and 49%.	
Notes	"In an unintentional deviation from that protocol, 49 of the 407 randomly allocated persons had a household contact with influenza symptoms at recruitment (a potential co‐index patient). We also randomly assigned 6 of 407 persons who had symptoms for slightly more than 48 hours." The trial authors conclude that "Hand hygiene and face masks seemed to prevent household transmission of influenza virus when implemented within 36 hours of index patient symptom onset. These findings suggest that non‐pharmaceutical interventions are important for mitigation of pandemic and interpandemic influenza". Primary funding source: Centers for Disease Control and Prevention. Potential conflicts of interest: none disclosed.	
***Risk of bias***		
**Bias**	**Authors' judgement**	**Support for judgement**
Random sequence generation (selection bias)	Low risk	Randomisation was computer generated by a biostatistician. Quote:"A pre‐specified table of random numbers will be used to assign one of the three interventions to the household of the index case."
Allocation concealment (selection bias)	Low risk	The households of eligible study index patients were allocated to 3 groups in a 1:1:1 ratio under a block randomisation structure with randomly permuted block sizes of 18, 24, and 30 using a random‐number generator. Allocation was concealed from treating physicians and clinics and implemented by study nurses at the time of the initial household visit.
Blinding of participants and personnel (performance bias) All outcomes	High risk	Quote: "Participants and personnel administering the interventions were not blinded to group assignment."
Blinding of outcome assessment (detection bias) All outcomes	Unclear risk	It is not stated if the outcome assessor was blinded.
Incomplete outcome data (attrition bias) All outcomes	High risk	Dropout was accounted for. Dropout from the randomised population was high: 32% in control group, 37.5% in hand hygiene group, and 39.4% in face mask and hand hygiene group. Reasons for dropout were distributed evenly across the 3 groups. Trial authors report follow‐up as proportion of patients remaining in the study after initial dropout.
Selective reporting (reporting bias)	Unclear risk	In general good reporting

**Table:** DiVita 2011

***Study characteristics***		
Methods	The impact of hand‐washing promotion on the risk of household transmission of influenza, ILI, and fever was tested in rural Bangladesh. ILI was defined as fever in children < 5 years old and fever with cough or sore throat in individuals > 5 years old. Households were randomised to intervention or control. The intervention group received hand‐washing stations with soap and daily hand‐washing motivation at critical times for pathogen transmission, such as after coughing or sneezing. Daily surveillance was conducted, and household members with fever were tested for influenza viruses by PCR. Secondary attack ratios (SAR) were calculated for influenza, ILI, and fever in each arm. Logistic regression with generalised estimating equations was used to estimate the significance of the SAR comparison whilst controlling for clustering by household.	
Participants	The study included 233 patient index cases (intervention group = 100, control group 133) with 2540 household contacts (intervention group = 134, control group = 1226). Inclusion criteria: index case patients (individuals who developed ILI within the previous 2 days and were the only symptomatic person in their household) as well as their household contacts	
Interventions	Hand‐washing stations with soap and daily hand‐washing motivation versus control. See Table 4 for details.	
Outcomes	SAR were calculated for influenza, ILI, and fever. ILI was defined as fever in children < 5 years old and fever with cough or sore throat in individuals > 5 years old. No safety outcomes reported.	
Notes	Funding source unknown. Disclosure of interest: none declared.	
***Risk of bias***		
**Bias**	**Authors' judgement**	**Support for judgement**
Random sequence generation (selection bias)	Unclear risk	Insufficient details provided
Allocation concealment (selection bias)	Unclear risk	Insufficient details provided
Blinding of participants and personnel (performance bias) All outcomes	Unclear risk	Insufficient details provided
Blinding of outcome assessment (detection bias) All outcomes	Unclear risk	Insufficient details provided
Incomplete outcome data (attrition bias) All outcomes	Unclear risk	Insufficient details provided
Selective reporting (reporting bias)	Unclear risk	Insufficient details provided

**Table:** Farr 1988a

***Study characteristics***		
Methods	6‐month cluster‐RCT, controlled, double‐blind of the efficacy of virucidal nasal tissues in the prevention of natural cold, conducted in Charlottesville, Virginia, USA. Many of the families were enrolled because 1 or more family members worked at the State Farm Insurance Company; the remaining families were recruited from the Charlottesville community by advertisement in a local newspaper. Families were randomly assigned by the sponsoring company to receive boxes of treated tissues, placebo tissues, or no tissues. The randomisation was performed by computer. Study participants and investigators were unaware of the type of tissues each family was randomised to receive. Blinding efficacy was tested using a questionnaire: the mothers in each family were asked twice if she believed her family was using virucidal or placebo tissues. Participants in the treated and placebo groups were instructed to use only tissues received through the study, whilst families in the additional control group without tissues were allowed to continue their usual practice of personal hygiene. Each family member kept a daily listing of respiratory symptoms on a record card. A nurse epidemiologist visited each family monthly to encourage recording.	
Participants	186 families, 58 in the active group, 59 in the placebo group, and 69 in the no‐tissues group. A total of 302 families were originally recruited; 116 families who did not comply with the study protocol, lost their surveillance cards, could not complete the protocol were excluded from the analysis.	
Interventions	Use of virucidal tissues versus placebo tissues versus no tissues. The treated tissues were impregnated with malic and citric acids and sodium lauryl sulphate, whilst placebo tissues contained saccharin. See Table 4 for details.	
Outcomes	Laboratory: serological evidence: no Effectiveness: respiratory illness Safety: N/A	
Notes	The authors concluded that virucidal tissues have only a small impact on the overall rate of natural acute respiratory illnesses. The total illness rate was lower in families using virucidal tissues than in both of the other study groups, but only the difference between active and placebo groups was statistically significant (3.4 illness per person versus 3.9 for placebo group, P = 0.04, and 3.6 for the no‐tissue control group, P = 0.2, and overall 14% to 5% reduction). The questionnaire results suggest that some bias may have been present since a majority of mothers in the virucide group believed they were receiving the 'active' tissues. Another possible explanation of the low effectiveness of virucidal tissues is poor compliance by children in use of the virucidal tissues. A well‐designed and honestly reported study. Funding source not reported. Potential conflicts of interest: none disclosed.	
***Risk of bias***		
**Bias**	**Authors' judgement**	**Support for judgement**
Random sequence generation (selection bias)	Unclear risk	Quote:"The randomisation was performed by computer in each trial." However, method of sequence generation is not stated.
Allocation concealment (selection bias)	Unclear risk	Quote: "In trial I, families were randomly assigned by the sponsoring company to receive boxes of treated tissues, placebo tissues or no tissues." Quote: "Families with one or two children were randomised in one stratum, and families with three or more children were randomised in a second stratum in trial I." Concealment of allocation not described
Blinding of participants and personnel (performance bias) All outcomes	Low risk	Quote: "Study participants and investigators were unaware of the type of tissues which each family was randomised to receive in both trials. In trial I, the mother in each family was asked twice if she believed her family was using active or placebo tissues, first after three months and then at the end of the study."
Blinding of outcome assessment (detection bias) All outcomes	Low risk	Quote: "Study participants and investigators were unaware of the type of tissues which each family was randomised to receive in both trials. In trial I, the mother in each family was asked twice if she believed her family was using active or placebo tissues, first after three months and then at the end of the study."
Incomplete outcome data (attrition bias) All outcomes	High risk	Quote: "A total of 116 of the 302 families were excluded from the analysis. Families were excluded if they lost their surveillance cards or did not conscientiously record data, did not comply with the study protocol, or simply could not complete the protocol for family reasons. It was discovered that families with five or more members had so many colds that it was not possible to distinguish primary and secondary illnesses. These large families were therefore excluded from the analysis in trial I and were excluded from enrolment in trial II."
Selective reporting (reporting bias)	Low risk	All indicated outcomes are reported.

**Table:** Farr 1988b

***Study characteristics***		
Methods	Six‐month randomised, controlled, double‐blind trial of the efficacy of virucidal nasal tissues in the prevention of natural cold, conducted in Charlottesville, Virginia, USA. Families were recruited from the Charlottesville community by advertisement in a local newspaper. Families were randomly assigned by the sponsoring company to receive either virucidal tissues or placebo‐treated tissues. Stratified randomisation was performed by computer, and the strata were defined by total number in the family. Study participants and investigators were unaware of the type of tissues each family was randomised to receive. Each family member kept a daily listing of respiratory symptoms on a record card. A nurse epidemiologist visited each family monthly to encourage recording. In addition, a study monitor visited each family bimonthly to further encourage compliance and reporting of symptoms.	
Participants	98 families, 58 in the active group and 40 in the placebo group. 231 families were initially recruited, 222 completed the trial, data of 98 families were analysed. The other families were excluded from the analysis because they complained of side effects (sneezing, etc.) or reported not using the tissues regularly. See Table 4 for details.	
Interventions	Use of virucidal tissues versus placebo tissues. The treated tissues were impregnated with malic and citric acids and sodium lauryl sulphate, whilst the placebo tissues contained succinic acid. Participants in the treated and placebo groups were instructed to only use tissues received through the study.	
Outcomes	Laboratory: serological evidence: no Effectiveness: respiratory illness Safety: N/A	
Notes	The study suggests that virucidal tissues have only a small impact on the overall rate of natural acute respiratory illnesses. The total illness rate was lower in families using virucidal tissues than in the other study group, but the difference between active and placebo groups was not statistically significant. There was a small, non‐significant drop in illness rates across families (5%). The tissues appeared to be ineffective as the drop was confined to primary illness unaffected by tissue use. The placebo (succinic acid) was not inert, and was associated with cough and nasal burning. This impacted on allocation concealment. A well‐designed and honestly reported study marred by transparent allocation. Funding source not reported. Potential conflicts of interest: none disclosed.	
***Risk of bias***		
**Bias**	**Authors' judgement**	**Support for judgement**
Random sequence generation (selection bias)	Unclear risk	"The randomisation was performed by computer in each trial." However, method of sequence generation is not stated.
Allocation concealment (selection bias)	Unclear risk	Quote: "In trial II, families were randomly assigned by the sponsor to receive either virucidal tissues or placebo treated tissues." Quote:"In trial II, stratified randomisation was again used, but this time the strata were defined by total number in the family (i.e., one stratum for two‐member families, another stratum for three‐member families, and a final one for four‐member families)." Concealment of allocation not described
Blinding of participants and personnel (performance bias) All outcomes	Low risk	Quote:"Study participants and investigators were unaware of the type of tissues which each family was randomised to receive in both trials."
Blinding of outcome assessment (detection bias) All outcomes	Low risk	Quote:"Study participants and investigators were unaware of the type of tissues which each family was randomised to receive in both trials."
Incomplete outcome data (attrition bias) All outcomes	High risk	Quote:"A total of 222 (of 231) families completed trial II; 9 families were terminated early (table 1). In 124 families, one or more family members reported not using the tissues regularly and/or reported having significant side effects. The data from these families were not analysed, leaving 58 families (177 persons) and 40 families (114 persons) for analysis in the virucide and placebo groups, respectively."
Selective reporting (reporting bias)	Low risk	All indicated outcomes are reported.

**Table:** Feldman 2016

***Study characteristics***		
Methods	Prospective cluster‐RCT. Ships from a single, central naval base. Ships were stratified by vessel classes (corvette, fast missile boat, and patrol boat).	
Participants	All people participating in security operations, routine exercises, and patrol at a single, central naval base were eligible. The actual number of participants in the groups is not reported.	
Interventions	Chlorhexidine gluconate (CHG) dispensers in addition to soap‐and‐water hand‐washing versus soap‐and‐water hand‐washing. See Table 4 for details.	
Outcomes	Laboratory: bacterial palm cultures from 30 sailors from each group using a modified bag broth technique with sterile brain‐heart broth, at 0 and 4 months (sample participants) Effectiveness: Primary outcome: incidence of infectious diseases reported by the computerised patient records system using ICD‐9 diagnoses and grouped into diarrhoeal, respiratory, and skin infections; the number of sick call visits; and the number of sick leave and light‐duty days incurred by the sailors Secondary outcome: subclinical morbidity (i.e. symptoms of self‐reported infectious diseases) Safety: not reported	
Notes	No report on adherence Study was conducted between May and September 2014 (4 months follow‐up). CHG availability onboard the ships did not reduce the transmission of infectious diseases or colonisation. Government funded (Israeli Defense Force Medical Corps). Potential conflicts of interest: none disclosed.	
***Risk of bias***		
**Bias**	**Authors' judgement**	**Support for judgement**
Random sequence generation (selection bias)	Unclear risk	No description of randomisation
Allocation concealment (selection bias)	Unclear risk	No description of allocation
Blinding of participants and personnel (performance bias) All outcomes	High risk	Unblinded. Self‐reported outcomes
Blinding of outcome assessment (detection bias) All outcomes	Unclear risk	No information if personnel collecting data for ICD‐9 diagnosis were blinded
Incomplete outcome data (attrition bias) All outcomes	Unclear risk	No participants flow chart, no attrition data
Selective reporting (reporting bias)	Unclear risk	No protocol to compare

**Table:** Fretheim 2022a

***Study characteristics***		
Methods	Pragmatic RCT	
Participants	3717 participants in Norway (glasses n = 1852; no glasses n = 1865) Inclusion criteria: were at least 18 years of age; did not regularly wear glasses; owned or could borrow glasses that they could use (e.g. sun‐glasses); had not contracted COVID‐19 in the 6 weeks prior to participation; did not have COVID‐19 symptoms when providing consent; were willing to be randomised to wear, or not wear glasses outside their home when close to others for a 2‐week period; provided informed consent; and contact lenses were allowed in the control group for those dependent on this visual aid. Exclusion criteria: does regularly wear glasses (contact lenses are accepted); and contracted COVID‐19 after December 15th 2021.	
Interventions	Intervention group: wearing eyeglasses (any type) when close to other people outside their home (on public transport, in shopping malls etc.), over a 14‐day period. The control: encouraged not to wear glasses when close to others outside their home. See TIDieR Table (Table 4) for details.	
Outcomes	Primary outcome Any positive COVID‐19 test result reported to the Norwegian Surveillance System for Communicable Diseases (MSIS), from day 3 to day 17 of the study period. Secondary outcomes Any positive COVID‐19 test result based on self‐report, from day 1 to day 17 of the study period. Episode of respiratory infection based on self‐report of symptoms from day 1 to day 17 of the study period. Respiratory infection was defined as having 1 respiratory symptom (stuffed or runny nose, sore throat, cough, sneezing, heavy breathing) and fever, or 1 respiratory symptom and at least 2 more symptoms (body ache, muscular pain, fatigue, reduced appetite, stomach pain, headache, loss of smell). Healthcare use for respiratory symptoms, self‐reported, from day 1 to day 17 of the study period. Healthcare use for injuries, self‐reported, from day 1 to day 17 of the study period. Healthcare use (all causes), self‐reported, from day 1 to day 17 of the study period. Healthcare use for respiratory symptoms as registered in Norwegian Patient Registry (NPR), from day 3 to day 28 of the study period. Healthcare use for injuries (from day 1 to day 21 as registered in NPR and the Norwegian Registry for Primary Health Care (KPR), from day 3 to day 28 of the study period. Healthcare use (all causes) as registered in NPR and KPR from day 1 to day 21 of the study period.	
Notes	The study did not report on the latter 4 outcomes due to lack of access to this data at the time of publication. Negative experiences of using the eyeglasses were reported: fogging, feeling uncomfortable and tiring, reduced vision, fall, feeling silly when wearing glasses indoor, headache. Funding: the costs of running the trial were covered by the Centre for Epidemic Interventions Research (CEIR), Norwegian Institute of Public Health. Competing interests: all authors declare: no competing interests.	
***Risk of bias***		
**Bias**	**Authors' judgement**	**Support for judgement**
Random sequence generation (selection bias)	Low risk	Automatically randomised after signing the consent form in the online recruitment platform (Nettskjema).
Allocation concealment (selection bias)	High risk	A digital recruitment platform (Nettskjema) was used to generate allocation. However, more participants in the intervention group wore face masks.
Blinding of participants and personnel (performance bias) All outcomes	High risk	An open‐label study. Participants and investigators were not blinded.
Blinding of outcome assessment (detection bias) All outcomes	High risk	Outcome is self‐reported positive COVID‐19 test result reported to the Norwegian Surveillance System for Communicable Diseases (MSIS). However, the public policy requiring confirmatory PCR‐test had changed during the study conduct which may have affected case detection.
Incomplete outcome data (attrition bias) All outcomes	Low risk	Participants flow chart was provided.
Selective reporting (reporting bias)	Low risk	No deviation from the published protocol.

**Table:** Goodall 2014

***Study characteristics***		
Methods	A 2X2 factorial RCT with 4 treatment arms Vitamin D_3_ and gargling Placebo and gargling Vitamin D_3_ and no gargling Placebo and no gargling	
Participants	600 students from McMaster University, Hamilton, Ontario, Canada, randomised to the following. Vitamin D and gargling (N = 150, analysed 135) Vitamin D and no gargling (N = 150, 123 outcomes included in analysis) Placebo and gargling (N = 150, 121 known outcomes included in analysis) Placebo and no gargling (N = 150, 113 known outcomes included in analysis) Inclusion criteria: aged ≥ 17 years and lived with at least 1 student house mate. Exclusion criteria: students with contraindicated medical conditions (hypercalcaemia, parathyroid disorder, chronic kidney disease, use of anticonvulsants, malabsorption syndromes, sarcoidosis), who were currently or planning to become pregnant, who were taking ≥ 1000 international units (IU)/day vitamin D, or who were unable to swallow capsules	
Interventions	See Table 4 for details.	
Outcomes	Laboratory (influenza assessed via weekly self‐collected nasal swabs; only swabs for symptomatic participants were assessed). Lab‐confirmed influenza was determined by testing the Day 1 nasal swabs using an in‐house enterovirus/rhinovirus PCR and, if negative, a commercial multiplex PCR able to detect 16 respiratory viruses and viral subtypes (xTAG RVP FAST, Luminex, Austin TX). Clinical URTI assessed via weekly online surveys. Clinical URTI is defined as the participant’s perception of cold in conjunction with 1 or more symptoms (runny/stuffy nose, congestion, cough, sneezing, sore throat, muscle aches, or fever). When participants reported symptoms but were uncertain if they were ill, adjudication was applied by 2 clinicians. Safety: None assessed/reported by the investigators.	
Notes	Study was conducted during 2 periods: September to October in 2010 and 2011. Partial governmental funding. Competing interests: the authors declare that they have no competing interests.	
***Risk of bias***		
**Bias**	**Authors' judgement**	**Support for judgement**
Random sequence generation (selection bias)	Unclear risk	No description on how the randomisation sequence was generated
Allocation concealment (selection bias)	Low risk	Study used opaque, sealed, serially numbered envelopes. Envelopes were only accessed when both personnel were present.
Blinding of participants and personnel (performance bias) All outcomes	Low risk	Due to the nature of gargling with tap water, this intervention was not blinded. However, all other aspects of the study were blinded. Self‐reported symptoms were adjudicated by 2 clinicians.
Blinding of outcome assessment (detection bias) All outcomes	Low risk	Except for gargling, all other participants and study personnel were blinded.
Incomplete outcome data (attrition bias) All outcomes	Low risk	Study flow chart and reasons for lost to follow‐up are provided, imputation used for missing outcomes.
Selective reporting (reporting bias)	Low risk	All planned study outcomes were reported and match the published study protocol.

**Table:** Gutiérrez‐García 2022

***Study characteristics***		
Methods	Single‐blind (analyst) randomised controlled trial carried out in a single centre in Mexico City during September to November 2020. Randomisation was through tokens in opaque envelopes but the trial was open to all except the data analysts. There were some imbalances in age groups post‐randomisation at baseline in age and comorbidities	
Participants	85 front line healthcare workers, unvaccinated and with no history of COVID infection in each arm. 6 and 1 were excluded from the analysis as they tested positive to CUVID within 14 days of recruitment. Follow‐up was 2 weeks	
Interventions	Neutral electrolysed water (SES) (pH 6.5 to 7.5) nasal and oral rinses 3 times daily and PPE versus PPE only for the prevention of SARS‐CoV‐2 infection. See Table 4 for details.	
Outcomes	Laboratory RT‐PCR no further described “according to the WHO guidelines”, once only presumably with symptoms. Effectiveness COVID‑19 disease confirmed by RT‑PCR, between the 14th day since their recruitment and the 28th day of follow‐up. The following are listed as COVID‑19 signs and symptoms: dry cough, fever > 37.5 °C, headache, myalgia, arthralgia, rhinorrhoea, conjunctivitis, pharyngodynia, odynophagia. 1 and 10 participants were positive in the intervention and control arms respectively. All 11 were nurses. Safety Local harms from SES applications – none reported	
Notes	The authors conclude that quote: “the prophylactic protocol was demonstrated as a protective factor, in more than 90%, for developing the disease, and without adverse effects. Nasal and oral rinses with SES maybe an efficient alternative to reinforce the protective measures against COVID‑19 disease and should be further investigated.” Funding: no funding was received. Competing interests: the authors RGG, JCA and IDE declare that they have no competing interests. ACL, NMS and BPM state that they are employees at Esteripharma S.A. de C.V. company but did not participate in the decision to publish the results of the study, nor in the selection of the volunteers or in its development.	
***Risk of bias***		
**Bias**	**Authors' judgement**	**Support for judgement**
Random sequence generation (selection bias)	Unclear risk	Insufficient information provided.
Allocation concealment (selection bias)	High risk	Nurse or doctor chose one of two identical tokens that were placed inside an opaque plastic container. One token was labelled ‘with SES’ (treatment group) and the other ‘without SES’ (control group).
Blinding of participants and personnel (performance bias) All outcomes	High risk	Not blinded.
Blinding of outcome assessment (detection bias) All outcomes	Low risk	Primary endpoint was the number of healthcare professionals, nurses, or physicians, with COVID‑19 disease confirmed by RT‐PCR. Researchers that performed the statistical analyses were blinded.
Incomplete outcome data (attrition bias) All outcomes	Low risk	Minimal exclusions from the analysis.
Selective reporting (reporting bias)	Low risk	Pre‐specified outcomes reported.

**Table:** Gwaltney 1980

***Study characteristics***		
Methods	Study assessed the effectiveness of aqueous iodine applied to the fingers in blocking hand transmission of experimental infection with rhinovirus from 1 volunteer to another. Healthy, young adult volunteers were recruited from the general population at the University of Virginia, Charlottesville. Volunteers were not informed about the contents of the hand preparation until after the study. 2 experiments were conducted to evaluate the virucidal activity of aqueous iodine applied to the fingers immediately before viral contamination. Another 2 experiments were conducted to determine whether there was sufficient residual activity of aqueous iodine after 2 hours to interrupt viral spread by the hand route. Volunteers who were donors of virus for the hand exposures were challenged intranasally on 3 consecutive days with the rhinovirus strain HH. Recipients were randomly assigned to receive iodine or placebo. The donors contaminated their hands with nasal secretions by finger to nose contact before the exposure. Hand contact was made between a donor and a recipient by stroking of the fingers for 10 seconds. Donors and recipients wore masks during the exposure period.	
Participants	15 and 20 volunteers in 2 experiments	
Interventions	Treatment of fingers with iodine versus placebo. The virucidal preparation used was aqueous iodine (2% iodine and 4% potassium iodide). The placebo was an aqueous solution of food colours. See Table 4 for details.	
Outcomes	Experimental rhinovirus infection reduced (P = 0.06) Laboratory: serological evidence Effectiveness: rhinovirus infection (based on serology, isolation, and clinical symptoms) with high‐score clinical illness. Score was published elsewhere. Safety: N/A	
Notes	Risk of bias: high (poor description of randomisation process, concealment, or allocation) Notes: the study suggests that aqueous iodine applied to the fingers was effective in blocking transmission by hand contact of experimental infection with rhinovirus for up to 2 hours after application (1 out 10 volunteers were infected compared to 6 out of 10 in the placebo preparation arm, P = 0.06 with Fisher's exact test). The effectiveness of iodine treatment of the fingers in interrupting viral transmission in volunteers recommends its use for attempting to block transmission of rhinovirus under natural conditions. Although the cosmetic properties of 2% aqueous iodine make it impractical for routine use, it can be used as an epidemiologic tool to study the importance of the hand transmission route and to develop an effective cosmetically acceptable hand preparation. A summarily reported study. Funding source not reported. Disclosure of interest: none mentioned.	
***Risk of bias***		
**Bias**	**Authors' judgement**	**Support for judgement**
Random sequence generation (selection bias)	Unclear risk	Insufficient information
Allocation concealment (selection bias)	Unclear risk	insufficient information
Blinding of participants and personnel (performance bias) All outcomes	Low risk	Quote:Quote: "The viricidal preparation used was aqueous iodine... . The placebo was an aqueous solution of food colors... mixed to resemble the color of iodine. An odor of iodine was given to the placebo... . Volunteers were not informed about the contents of the hand preparation until after the study."
Blinding of outcome assessment (detection bias) All outcomes	Unclear risk	It is not stated whether the outcome assessor was blinded or not.
Incomplete outcome data (attrition bias) All outcomes	Unclear risk	Insufficient information
Selective reporting (reporting bias)	Unclear risk	Insufficient information

**Table:** Hartinger 2016

***Study characteristics***		
Methods	Communities were randomised to a comprehensive intervention was an improved solid‐fuel stove, installation of a kitchen sink with running water, solar drinking water disinfection, education on hand‐washing, and separating animals from the kitchen environment.	
Participants	534 children (267 in each group) in 51 communities (25 in intervention, 26 in control group). 250 children/households in the intervention group and 253 children/households in the control group were available for follow‐up. Conducted in a rural farming area	
Interventions	Environmental home‐based intervention package consisting of improved solid‐fuel stoves, kitchen sinks, solar disinfection of drinking water, and hygiene promotion. See Table 4 for details.	
Outcomes	Laboratory: *Escherichia coli* (not relevant to this review) Effectiveness: weekly collection of daily diary data on illness. ARI was defined as child presenting cough or difficulty breathing, or both. ALRI was defined as child presenting cough or difficulty breathing, with a raised respiratory rate (> 50 per min in children aged 6 to 11 months and > 40 per min in children aged 12 months) on 2 consecutive measurements. Safety: none described in methods and none reported	
Notes	The authors conclude that “combined home‐based environmental interventions slightly reduced childhood diarrhoea, but the confidence interval included unity. Effects on growth and respiratory outcomes were not observed, despite high user compliance of the interventions. The absent effect on respiratory health might be due to insufficient household air quality improvements of the improved stoves and additional time needed to achieve attitudinal and behaviour change when providing composite interventions”. Well‐reported trial. Age of children not reported. Funding: this work was supported by the UBS Optimus Foundation, Freiwillige Akademische Gesellschaft, Basel, Stiftung EmiliaGuggenheim‐Schnurr, Basel. Conflict of interest: the authors have no conflicts of interest to declare.	
***Risk of bias***		
**Bias**	**Authors' judgement**	**Support for judgement**
Random sequence generation (selection bias)	Unclear risk	Covariate‐constrained randomisation is mentioned, but method not described.
Allocation concealment (selection bias)	Unclear risk	Method not mentioned
Blinding of participants and personnel (performance bias) All outcomes	High risk	Unblinded
Blinding of outcome assessment (detection bias) All outcomes	High risk	Data collected by field worker and recorded by parent. All would be aware of allocation.
Incomplete outcome data (attrition bias) All outcomes	Low risk	Low attrition rate, reasons stated, balanced between groups.
Selective reporting (reporting bias)	Low risk	It is unlikely that other outcomes were measured but not reported.

**Table:** Helsingen 2021

***Study characteristics***		
Methods	Non‐inferiority open randomised trial carried out in May 25 to June 15 2020 during the first lockdown in Norway. Eligible individuals were randomised 1:1 stratified by fitness centre by a computerised random number generator to no access to fitness centre or access to fitness centre with “mitigation measures”	
Participants	3825 people aged 18 to 65 with no risk factors for Covid 19 (diabetes, cardiovascular disease including hypertension, age > 65). 61 randomised participants (18 and 43, respectively) withdrew consent before start of the intervention with 3764 remaining	
Interventions	The intervention consisted in gym access with: avoidance of body contact; 1 m distance between individuals at all times; 2 m distance for high intensity activities; disinfection of all work stations; cleaning of all equipment after use by participant; regular cleaning of facilities and access control by facility employees to ensure distance measures and avoid overcrowding; open changing rooms with showers and saunas remained closed; staff was present during all opening hours; lids on trash cans removed; individuals were instructed to stay home if they had any Covid‐19 related symptoms, participants were advised to avoid touching their eyes, nose and mouth. See Table 4 for details.	
Outcomes	Laboratory Self‐administered (at times facilitated by HCW) NP, saliva or OP swabs in transport medium taken at day 14 to 15 from beginning sent to central lab. RT‐RPC performed. Testing of antibodies (IGG) was carried out in late June with a mailed self‐administered spot slide which was then mailed and analysed centrally. Effectiveness Primary: PCR positivity in both arms Co‐primary: hospital admission in the two arms at 21 days (via data linkage) Secondary: proportion of participants with SARS‐CoV‐2 antibodies in the 2 study arms at 30 days. Testing also carried out for gym staff. Safety NR	
Notes	The authors conclude that “Provided good hygiene and physical distancing measures and low population prevalence of SARS‐CoV‐2infection, there was no increased infection risk of SARS‐CoV‐2 in fitness centres in Oslo, Norway for individuals without Covid‐19‐relevant comorbidities.” There was low and declining incidence on C19 in the Oslo area during the time of the trial as reported by the authors. The authors call the analysis set ITT but consent withdrawal individuals were not part of the analysis. There was marked difference in PCR uptake (88.7% in the training arm; 71.4% in the no‐training arm) and no cycle thresholds are reported. Funding: this study was funded by the Norwegian Research Council, grant no. 312757. The grant paid for necessary equipment, study personnel and researchers. Competing interests: Dr. Lise M. Helsingen reports grants from Norwegian Research Council (grant no. 312757), during the conduct of the study. All other authors declare no competing interests in relation to this work.	
***Risk of bias***		
**Bias**	**Authors' judgement**	**Support for judgement**
Random sequence generation (selection bias)	Low risk	Computer random‐number generator
Allocation concealment (selection bias)	High risk	Allocation performed by one of the study authors
Blinding of participants and personnel (performance bias) All outcomes	High risk	Not blinded.
Blinding of outcome assessment (detection bias) All outcomes	High risk	Not blinded.
Incomplete outcome data (attrition bias) All outcomes	High risk	More women were compliant with SARS‐CoV2 testing in the training arm as compared to the no‐training arm, and compliant individuals were somewhat younger in the training arm compared to the non‐training arm.
Selective reporting (reporting bias)	Low risk	All pre‐specified outcomes reported

**Table:** Hubner 2010

***Study characteristics***		
Methods	A prospective, controlled, intervention‐control group design to assess the epidemiological and economical impact of alcohol‐based hand disinfectants use at workplace. Volunteers in public administrations in the municipality of the city of Greifswald were randomised into 2 groups. Participants in the intervention group were provided with alcoholic hand disinfection, the control group was unchanged. In all, 1230 person‐months were evaluated.	
Participants	Employees (n = 134) from the administration of the Ernst‐Moritz‐Arndt University Greifswald, the municipality of Greifswald and the state of Mecklenburg‐Pomerania, were recruited for the study and randomised to intervention (N = 67) or control (N = 67). Final analysis was performed on 64 from the intervention and 65 from the control group. Inclusion criteria: all administrative officers, who did not already apply hand disinfection at work, were considered for participation and were invited by email or mail (n = 850). The 134 participants declared their written consent to participate and completed a pre‐study survey with demographic, social, health, and work‐related questions to provide data for randomisation. Exclusion criteria: employees that were already using hand disinfectants at work	
Interventions	Alcohol‐based hand disinfectants use at workplace versus usual hygiene. See Table 4 for details.	
Outcomes	Respiratory and gastrointestinal symptoms and days of work were recorded based on a monthly questionnaire over 1 year.	
Notes	Funding source not mentioned. Competing interests: the authors declare a financial competing interest: GK is employed by Bode Chemie GmbH, Hamburg, Germany. NOH and AK received financial support for research from Bode Chemie in the past. All other authors declare no conflict of interest.	
***Risk of bias***		
**Bias**	**Authors' judgement**	**Support for judgement**
Random sequence generation (selection bias)	Unclear risk	No details provided.
Allocation concealment (selection bias)	Unclear risk	No details provided.
Blinding of participants and personnel (performance bias) All outcomes	High risk	Unblinded study
Blinding of outcome assessment (detection bias) All outcomes	High risk	Self‐reported outcomes
Incomplete outcome data (attrition bias) All outcomes	Low risk	Lost to follow‐up minimal and similar in 2 groups
Selective reporting (reporting bias)	Unclear risk	No protocol available

**Table:** Huda 2012

***Study characteristics***		
Methods	Poorly described cluster‐RCT. Partial report of the SHEWA‐B trial focused on changing 11 targeted behaviours in villages to measure the impact on diarrhoea and respiratory illness amongst children. Unit of randomisation is not clear, but was probably a village. A group of 10 to 17 households within a village were the participants, based on the household having at least 1 child under the age of 5.	
Participants	A total of 1692 participants (intervention = 848, control = 844) at baseline and 1699 participants at 18 months (intervention = 849, control = 850) Households were eligible if they have a child < 5 years of age and a guardian agreed to participate.	
Interventions	SHEWA‐B programme targeting improved latrine coverage and usage, access to and use of arsenic‐free water, and improved hygiene practices using soaps. See Table 4 for details.	
Outcomes	Laboratory: none described in methods and none reported Effectiveness: ARI and diarrhoea. ARI defined as cough and fever or difficulty breathing and fever within 48 hours prior to interview. Safety: none described in methods and none reported	
Notes	The authors conclude that quote: “The prevalence of childhood diarrhea and respiratory illness was similar in the intervention and control communities”. Poorly reported trial. This research activity was funded by the United Kingdom's Department for International Development (DFID). Disclosure of interest: none mentioned.	
***Risk of bias***		
**Bias**	**Authors' judgement**	**Support for judgement**
Random sequence generation (selection bias)	Unclear risk	Mentions random‐number tables, but not clear if this was for random selection or randomisation
Allocation concealment (selection bias)	Unclear risk	Method not described
Blinding of participants and personnel (performance bias) All outcomes	High risk	Unblinded
Blinding of outcome assessment (detection bias) All outcomes	High risk	Data on illness were collected by a resident of the village, who was likely to know treatment allocation.
Incomplete outcome data (attrition bias) All outcomes	High risk	Not reported. No flow diagram
Selective reporting (reporting bias)	Unclear risk	Unlikely that other outcomes were measured and not reported

**Table:** Ibfelt 2015

***Study characteristics***		
Methods	Cluster‐RCT in 12 daycare nurseries in Denmark. Centres in the intervention group had their linen and children’s toys commercially cleaned and disinfected every 2 weeks. Control group centres had usual practice. Swabbing for bacteria and respiratory viruses was conducted at baseline and the end of the intervention period.	
Participants	12 nurseries in Copenhagen (intervention = 6, control = 6) with a total of 587 children aged 6 months to 3 years Not clear how many children were in each group. Data on illness collected at the individual level, and on presence of bacteria and viruses at the cluster level.	
Interventions	Washing and disinfection of toys and linen every 2 weeks for 3 months. See Table 4 for details.	
Outcomes	Laboratory: counts of bacteria (not relevant to this review) and 11 respiratory viruses at baseline and end of intervention period, taken from swabs of 10 predefined locations in playroom (7 locations) and toilet area (3 locations). Viruses were influenza A and B; coronavirus NL63229E, OC43, and HKU1; parainfluenza virus 1, 2, 3, and 4; rhinovirus; RSV A/B; adenovirus; enterovirus; parechovirus; metapneumovirus; and bocavirus. Testing by PCR Effectiveness: illness counts in the children. Absence due to sickness recorded daily with reason categorised, but no definitions of illness provided. Safety: none mentioned in methods and none reported	
Notes	The authors conclude that “Although cleaning and disinfection of toys every two weeks can decrease the microbial load in nurseries, it does not appear to reduce sickness absence among nursery children”. The results of the disinfection are reported as follows: “The most prevalent virus was coronavirus (97% positive samples), followed by bocavirus (96%), adenovirus (73%) and rhinovirus (46%). The intervention reduced the presence of adenovirus, rhinovirus and RSV approximately two‐ to five‐fold [odds ratio (OR) 2.4, 95% confidence interval (CI) 1.1‐5.0 for adenovirus; OR 5.3, 95% CI 2.3‐12.4 for rhinovirus; OR 4.1, 95% CI 1.5‐11.2 for RSV] compared with the control group. On the other hand, metapneumovirus was found significantly less often in the control group than in the intervention group. The intervention had no effect on the detection of other viruses. The fomites with the highest presence of respiratory virus were pillows and sofas, followed by toys and playroom tables. When looking at the samples from the toys alone, there was a significant decrease following the intervention in the intervention group compared with the control group for rhinovirus (OR 3.8, 95% CI 1.3‐10.5; P = 0.01) and RSV (OR 5.2, 95% CI 1.1‐23.8; P = 0.04), but not adenovirus”. This a poorly reported cluster‐RCT. Its importance lies in the surface viral prevalence data (which could have been overestimated by PCR) and the finding that even in the presence of high viral prevalence, sickness was lower in the control (no surface disinfection) arm. This suggests the absence of other factors that could activate surface respiratory viruses. Funding: this work was supported by the Danish Council for Technology and Innovation under the Ministry of Science, Innovation and Higher Education as part of the Sundhed i Børneinstitutioner innovation consortium. Conflict of interest statement: Ecolab Denmark, Berendsen Denmark and 3M Denmark supplied materials and cleaning free of charge, but had no influence on the analysis of the data or the writing of the manuscript.	
***Risk of bias***		
**Bias**	**Authors' judgement**	**Support for judgement**
Random sequence generation (selection bias)	Unclear risk	Method not mentioned
Allocation concealment (selection bias)	Unclear risk	Method not mentioned
Blinding of participants and personnel (performance bias) All outcomes	High risk	Unblinded
Blinding of outcome assessment (detection bias) All outcomes	Low risk	Objective measure of bacterial and viral counts. However, illness reporting is unclear.
Incomplete outcome data (attrition bias) All outcomes	Unclear risk	No attrition or denominators given for results.
Selective reporting (reporting bias)	Low risk	Unlikely that other outcomes were measured but not reported

**Table:** Ide 2014

***Study characteristics***		
Methods	Randomised, open‐label, 2‐group parallel study of 757 high school students (15 to 17 years of age) conducted for 90 days during the influenza epidemic season from 1 December 2011 to 28 February 2012, in 6 high schools in Shizuoka Prefecture, Japan. The green tea gargling group gargled 3 times a day with bottled green tea, and the water gargling group did the same with tap water. The water group was restricted from gargling with green tea.	
Participants	A total of 747 students were enrolled (green tea gargling group = 384, water gargling group = 363) High school students (15 to 17 years of age) who attended 6 high schools in the Kakegawa and Ogasa districts of Shizuoka Prefecture, Japan	
Interventions	See Table 4 for details.	
Outcomes	Incidence of laboratory‐confirmed influenza Incidence of clinically defined influenza infection Time for which the participant was free from clinically‐defined influenza infection Clinically‐defined influenza infection, specified as fever (≥ 37.8 °C) plus any 2 of the following additional symptoms: cough, sore throat, headache, or myalgia. Influenza infection with viral antigen was detected by immunochromatographic assay. No safety data reported.	
Notes	Funding: this work was supported by Grants‐in‐Aid for Scientific Research (KAKENHI) Grant Number 23590887. Competing Interests: the authors have declared that no competing interests exist.	
***Risk of bias***		
**Bias**	**Authors' judgement**	**Support for judgement**
Random sequence generation (selection bias)	Low risk	Computer‐generated permuted block randomised schema
Allocation concealment (selection bias)	Low risk	Randomised at the Data Management Center of Shizuoka General Hospital in Japan
Blinding of participants and personnel (performance bias) All outcomes	High risk	Unblinded study
Blinding of outcome assessment (detection bias) All outcomes	High risk	Unblinded study
Incomplete outcome data (attrition bias) All outcomes	Low risk	Minimal attrition
Selective reporting (reporting bias)	Unclear risk	Protocol not available

**Table:** Ide 2016

***Study characteristics***		
Methods	Randomised controlled study in Japan. Participants were randomly allocated into the catechin‐treated (epigallocatechin gallate‐treated) or non‐treated face mask groups for 60 days from January to March 2016. Incidence of laboratory‐confirmed influenza infection was measured and compared between groups using Fisher's exact test. Multivariate analysis was performed to calculate adjusted ORs and associated 95% CIs.	
Participants	Participants included workers in a nursing home, a rehabilitation facility, and a hospital. A total of 234 participants were eligible for the study (catechin group, n = 118; control group, n = 116).	
Interventions	Catechin‐treated mask versus non‐treated face mask. See Table 4 for details.	
Outcomes	Incidence of laboratory‐confirmed influenza infection Laboratory‐confirmed influenza infection with viral antigen detected by immunochromatographic assay performed when participants reported ILI. No safety outcomes reported.	
Notes	Funding: this work was supported in part by a grant from the Japan Society for the Promotion of Science (JSPS), through the Grant‐in‐Aid for JSPS Fellows (No. 15J10190 to KI) and Grants‐in‐Aid for Scientific Research (C) (15K08924 to HY). Conflict of Interest: the authors declare that they have no conflicts of interest.	
***Risk of bias***		
**Bias**	**Authors' judgement**	**Support for judgement**
Random sequence generation (selection bias)	Unclear risk	Computer‐generated randomisation, but method not stated
Allocation concealment (selection bias)	Low risk	Central randomisation service at Data Management Centre of Shizouka General Hospital
Blinding of participants and personnel (performance bias) All outcomes	Low risk	Double‐blinded
Blinding of outcome assessment (detection bias) All outcomes	Low risk	Attrition minimal
Incomplete outcome data (attrition bias) All outcomes	Low risk	Attrition minimal
Selective reporting (reporting bias)	Low risk	Specified outcomes reported.

**Table:** Jacobs 2009

***Study characteristics***		
Methods	Open‐RCT lasting 77 days from January 2008 to test “superiority” of face masks in preventing "URTI". This term appears as an acronym in the introduction and is not explained. It is assumed that it stands for 'upper respiratory infections', but it is preceded in the text by the term 'common cold', which is also lacking a definition. Randomisation was carried out in blocks within each of 3 professional figures (physicians, nurses, and “co‐medical” personnel).	
Participants	33 HCWs mainly females aged around 34 to 37 in a tertiary healthcare hospital in Tokyo, Japan. HCW with quote: “predisposing conditions” (undefined) to “URTI” and those taking antibiotics were excluded. A baseline descriptive survey was carried out including “quality of life”. 1 participant dropped out at end of week 1, but no reason is reported nor the allocation arm. Analysis was performed on 32 participants (mask = 17, no mask = 15).	
Interventions	Surgical mask MA‐3 (Osu Sangyo, Japan) during all phases of hospital work (n = 17) or no mask (n = 15) (except when specifically required by hospital SOPs). See Table 4 for details.	
Outcomes	Laboratory: N/A Effectiveness: URTI is defined on the basis of a symptoms score, with a score > 14 being a URTI according to Jackson’s 1958 criteria (“Jackson score”). These are not explained in text, although the symptoms are listed in Table 3 (any, sore throat, runny nose, stuffy nose, sneeze, cough, headache, ear ache, feel bad) together with their mean and scores SD by intervention arm. Safety: the text does not mention or report harms. These appear to be indistinguishable from URTI symptoms (e.g. headache which is reported as of significantly longer duration in the intervention arm). Compliance is self‐reported as high (84.3% of participants).	
Notes	The authors conclude that quote: “Face mask use in healthcare workers has not been demonstrated to provide benefit in terms of cold symptoms or getting colds. A larger study is needed to definitively establish non‐inferiority of no mask use”. This is a small, badly reported trial. The purpose of trials is to test hypotheses not to prove or disprove 'superiority' of interventions. There is no power calculation, and CIs are not reported (although there is a mention in Discussion). No accurate definitions of a series of important variables (e.g. URTI, runny nose, etc.) are reported, and the Jackson scores are not explained, nor their use in Japanese personnel or language validated. Intervention arm data not extracted due to the uncertainty of its meaning. Funding source not mentioned. Conflicts of interest: none to report	
***Risk of bias***		
**Bias**	**Authors' judgement**	**Support for judgement**
Random sequence generation (selection bias)	Unclear risk	Open RCT, but sequence generation not reported
Allocation concealment (selection bias)	Unclear risk	"Mask and no mask groups were formed using block randomisation of participants within their respective job categories: nurses, doctors, and co‐medical personnel." Concealment of allocation not described
Blinding of participants and personnel (performance bias) All outcomes	High risk	Unblinded study. Blinding not possible, as 1 group wore face masks
Blinding of outcome assessment (detection bias) All outcomes	High risk	Unblinded study
Incomplete outcome data (attrition bias) All outcomes	Low risk	1 dropout in each group accounted for.Quote: "Analyses were performed following the principles of intention‐to‐treat."
Selective reporting (reporting bias)	High risk	NB: influenza vaccine coverage was 100% in mask group and only 81% in the non‐mask‐wearing group.

**Table:** Kotch 1994

***Study characteristics***		
Methods	Pair‐matched, cluster‐RCT conducted from 19 October 1988 to 23 May 1989 in 24 childcare centres in North Carolina, USA The trial tested the effects of a hand‐washing and environment sterilising programme on diarrhoea (data not extracted) and ARIs. Child daycare centres had to care for 30 children or less, at least 5 of whom had to be in nappies, and intending to stay open for at least another 2 years. Randomisation is not described, nor are cluster coefficients reported.	
Participants	389 children aged 3 years or less in daycare for at least 20 hours a week. There were some withdrawals, but attrition of participants is not stated, only that in the end data for 31 intervention classrooms and 36 control classrooms were available. 291 children aged up to 24 months and 80 over 24 months took part. The text is very confusing, as 371 seems to be the total of the number of families that took part. No denominator breakdown by arm is reported, and numerators are only reported as new episodes per child‐year.	
Interventions	Structured hand‐washing and environment (including surfaces, sinks, toilets, and toys) disinfecting programme with waterless disinfectant scrub. See Table 4 for details.	
Outcomes	Laboratory: N/A Effectiveness: ARI (coughing, runny nose, wheezing, sore throat, or earache) Safety: N/A	
Notes	Risk of bias: high (poor reporting of randomisation, outcomes, numerators and denominators) Note: the authors conclude that the fully adjusted RR for prevention of ARIs was 0.94 (−2.43 to 0.66). A poorly reported study. This study was supported in part by grant MCJ‐373111 from the Maternal and Child Health Program (Title V. Social Security Act), Health Resources and Services Administration, Department of Health and Human Services. Cal Stat^TM^ was contributed by Cal‐ gon Vestal Laboratories, a subsidiary of Merck and Co, Inc, St Louis, MO. Conflicts of interest: none to report.	
***Risk of bias***		
**Bias**	**Authors' judgement**	**Support for judgement**
Random sequence generation (selection bias)	Unclear risk	Quote: "Pair‐matched cluster‐randomised, controlled trial", but sequence generation not reported
Allocation concealment (selection bias)	Unclear risk	Centres were matched in pairs and then randomly allocated to either intervention or control programmes. Allocation concealment was not reported.
Blinding of participants and personnel (performance bias) All outcomes	High risk	Not possible (intervention was training session)
Blinding of outcome assessment (detection bias) All outcomes	High risk	"The same staff who conducted the training unobtrusively recorded observations at 5‐week intervals"
Incomplete outcome data (attrition bias) All outcomes	High risk	18 families were dropped, denominator not clear.
Selective reporting (reporting bias)	High risk	Denominators not clearly reported

**Table:** Ladegaard 1999

***Study characteristics***		
Methods	RCT with cluster‐randomisation to intervention or control. Of 10 institutions, 2 were excluded because they wanted institutions to be comparable in uptake area (i.e. housing and income). Interventions were administered to children, parents, and teachers at the institutions.	
Participants	Children 0 to 6 years old	
Interventions	Multifaceted: information, t‐shirts to the children with: "Clean hands ‐ yes, thank you", performance of a fairytale "The princess who did not want to wash her hands", exercise in hand‐washing, importance of clean and fresh air. The aims of the intervention were to: increase the hygiene education of the daycare teachers; motivate the children by practical learning to have better hand hygiene; and inform the parents about better hand hygiene. See Table 4 for details.	
Outcomes	34% decrease in "sickness" (probably mostly gastroenteritis)	
Notes	Risk of bias: only limited data available Note: the authors conclude that there was a 34% decrease in sickness in the intervention arm; this is probably overall sickness, as gastroenteritis is part of the outcomes (data not extracted). Only limited data available from translation by Jørgen Lous. Funding was received from a local part of the Danish Health Authority (Forebyggelserådet för Fyns Amt).	
***Risk of bias***		
**Bias**	**Authors' judgement**	**Support for judgement**
Random sequence generation (selection bias)	Unclear risk	Not described
Allocation concealment (selection bias)	Unclear risk	Randomisation by "lottery", the same as "flip the coin". Concealment not reported
Blinding of participants and personnel (performance bias) All outcomes	High risk	Not possible
Blinding of outcome assessment (detection bias) All outcomes	High risk	Not possible
Incomplete outcome data (attrition bias) All outcomes	High risk	Total numbers of children included in each arm Not reported.
Selective reporting (reporting bias)	High risk	Limited data reported, in particular denominators missing.

**Table:** Larson 2010

***Study characteristics***		
Methods	Cluster block‐randomised, controlled trial carried out between 20 November 2006 and 20 June 2008 in an upper Manhattan immigrant Latino neighbourhood (“19 month data collection period”). The study aimed at assessing the effects of education versus education and hand sanitiser use versus education and hand sanitiser use and common mask use against upper respiratory infections over a period of under 2 years. Follow‐up was through an automated telephone system with a small financial incentive (USD 20) for those with 75% or more compliance. Those reporting an ILI received a visit within 48 hours for swabbing. An index case was someone who at the “onset day of illness nobody else in the household had been symptomatic within the previous five days”. A secondary case for each episode quote: “was any member of the household who developed symptoms within five days following the index case”; “The secondary attack rate was defined as the number of secondary cases recorded within 5 days of the onset of symptoms in the index case divided by the number of household members minus one”. The text implies that the unit of observation was the episode (“study subjects contributed more than one episode in which they were considered to be the index case”).	
Participants	617 households were randomised to the education group (n = 211), the hand‐sanitiser group (n = 205), and the hand‐sanitiser and mask group (n = 201). There were 2708 participants, mostly adult Latino immigrants to the USA. Recruitment and allocation were carried out by household. There had to be at least 3 people living in the household, with at least 1 being a preschool or elementary school child, speaking English or Spanish, having a telephone, willingness to complete symptom assessments and have bimonthly home visits, and not using alcohol‐based hand sanitiser routinely. Intracluster correlation coefficients are reported on page 179 of the manuscript.	
Interventions	Written Spanish or English language educational materials regarding the prevention and treatment of URTIs and influenza or the same educational materials and hand sanitiser (Purell, J&J), in large (8‐ and 4‐ounce) and small (1‐ounce) containers to be carried by individual household members to work or school, or the same interventions as well as regular surgical face masks (Procedure Face Masks for adults and children, Kimberly‐Clark) with instructions for both the caretaker and the ill person to wear them when an ILI occurred in any household member. Replenishment of intervention stocks was done at the bimonthly home visit. Caretakers had to wear a mask for 7 days when within 3 feet of a symptomatic case. They were also encouraged to wear masks within 3 feet of any household member. Reinforcing phone calls were made 3 times in 6 days. The text clearly reports active influenza vaccine promotion during the bimonthly visits. (“The home visit to each household was made every 2 months to minimise study dropout, reinforce adherence to the assigned intervention, replenish product supplies and record use of supplies, answer questions, and correct ongoing misconceptions. At each visit, new educational materials regarding URTI prevention and treatment and influenza vaccination were distributed.” (PDF page 3). Also just before the Discussion as follows: “Influenza vaccination rates: There was an increase between the baseline and exit interview in all three groups that reported 50% of more of members receiving influenza vaccine (pre‐ versus post‐intervention for each group: 21.1% and 40.8% in the Education group, 19.0% and 57.1% in the hand sanitiser group, and 22.4% and 43.5% in the hand sanitiser and face mask group (P = 0.001). Additionally, those in the hand sanitiser group reported a significantly greater increase than the other 2 groups, controlling for baseline rates (P = 0.002)”) Coverage was unequal across groups, no information on the progressive impact of the vaccine, or indeed the nature of the vaccine(s) is reported. Apparently the first season was mild and the vaccine mismatched, compliance with the trial interventions was low in Arm 3, and a local epidemic of *Staphylococcus aureus* meant that the control group started washing hands. The trial authors report no effect on reporting rates of vaccine coverage by arms, but with so many confounders who knows? See Table 4 for details.	
Outcomes	Laboratory: PCR carried out on samples from deep nasal swabs for influenza and the most common other pathogens (RSV, rhinovirus, enterovirus, parainfluenza viruses, etc.). The text describing the results of the swabbing is confusing, but in general appears to be non‐random “Households reported 669 episodes of ILI (0 to 5 per individual)". Of the 234 deep nasal swabs obtained, 33.3% (n = 78) tested positive for influenza: 43.6% (n = 34) were influenza A and 56.4% (n = 44) were influenza B. Amongst the 66.7% who tested negative for influenza, 30.8% (48/156) tested positive for other viruses: 7 for respiratory syncytial virus, 9 for parainfluenza, 11 for enterovirus, 10 for rhinovirus, 6 for adenovirus, and 5 for metapneumovirus. Swabs were not obtained from the remaining 435 reported ILI episodes for the following reasons: 72.0% (n = 313) did not meet the CDC definition of an ILI and were therefore included in the URTI symptom count; 21.4% of episodes (n = 93) were reported after 48 hours of ILI onset or the participant refused to be swabbed; and the research staff were unable to reach the participant in 6.7% of episodes (n = 29). As no definition of URTI is given, it is unclear what kind of biases were introduced by the non‐swabbing of the 313/435 “not meeting CDC definition”. Effectiveness: ILI (CDC definition): “temperature of 37.8°C or more and cough and/or sore throat in the absence of a known cause other than influenza” URTI only referred to as “Viral upper respiratory infections (URTIs)”. Safety: N/A	
Notes	The authors conclude that quote: “the Hand Sanitizer group was significantly more likely to report that no household member had symptoms (P,0.01), but there were no significant differences in rates of infection by intervention group in multivariate analyses. Knowledge improved significantly more in the Hand Sanitizer group (P,0.0001). The proportion of households that reported >50% of members receiving influenza vaccine increased during the study (P.0.001). Despite the fact that compliance with mask wearing was poor, mask wearing as well as increased crowding, lower education levels of caretakers, and index cases 0–5 years of age (compared with adults) were associated with significantly lower secondary transmission rates (all P,0.02). In this population, there was no detectable additional benefit of hand sanitiser or face masks over targeted education on overall rates of URTIs, but mask wearing was associated with reduced secondary transmission and should be encouraged during outbreak situations. During the study period, community concern about methicillin‐resistant *Staphylococcus aureus* was occurring, perhaps contributing to the use of hand sanitiser in the Education control group, and diluting the intervention’s measurable impact”. The study is at high risk of bias. Randomisation and reasons for dropout are not described. Differentials in cluster characteristics across arms point to randomisation not having worked, and the confounding effects of a post randomisation staphylococcal scare are difficult to judge. Symptom‐driven follow‐up gives no idea of the effects on asymptomatic ILI/influenza. Poor definitions (URTI?). There are unexplained dropouts, and the analysis plan is unclear. Finally, the very small number of cases of influenza and an unclear swabbing attrition may introduce further elements of confounding. Funding: this study was funded by grant #1 U01 CI000442‐01, “Stopping URIs and Flu in the Family: The Stuffy Trial.” Conflicts of interest: none reported	
***Risk of bias***		
**Bias**	**Authors' judgement**	**Support for judgement**
Random sequence generation (selection bias)	Unclear risk	Quote: "Cluster block randomised, controlled trial", but sequence generation not reported
Allocation concealment (selection bias)	Unclear risk	Quote:"Households were block randomised into one of three groups" Allocation concealment not reported
Blinding of participants and personnel (performance bias) All outcomes	High risk	Blinding of participants and personnel was not possible.
Blinding of outcome assessment (detection bias) All outcomes	Unclear risk	Blinding of outcome assessment is not stated.
Incomplete outcome data (attrition bias) All outcomes	High risk	In control group households (n = 211), 26 dropped out and 37 did not consent. In hand‐sanitiser group households (n = 205), 21 dropped out and 36 did not consent. In hand‐sanitiser and face mask group households (n = 201), 19 dropped out and 35 did not consent. Reasons for dropout were not described.
Selective reporting (reporting bias)	Unclear risk	617 of 772 eligible households were randomised.

**Table:** Little 2015

***Study characteristics***		
Methods	Individuals sharing a household by mailed invitation through general practices in England were recruited. After consent, participants were randomised online by an automated computer‐generated random‐number program to receive either no access or access to a bespoke automated web‐based intervention that maximised hand‐washing intention, monitored hand‐washing behaviour, provided tailored feedback, reinforced helpful attitudes and norms, and addressed negative beliefs. Participants were enrolled into an additional cohort (randomised to receive intervention or no intervention) to assess whether the baseline questionnaire on hand‐washing would affect hand‐washing behaviour. Participants were not masked to intervention allocation, but statistical analysis commands were constructed masked to group. The primary outcome was number of episodes of RTIs in index participants in a modified intention‐to‐treat population of randomly assigned participants who completed follow‐up at 16 weeks.	
Participants	344 physician offices were recruited over a wide area of England, and 20,066 participants were enrolled and randomised to intervention (N = 16,086) and control (N = 10,026). Modified ITT was performed on 16,908 participants who completed the follow‐up questionnaire at 16 weeks (intervention = 8241 and control = 8667). Inclusion criteria: adult patients (aged 18 years or older) identified from computerised lists in general practitioner (GP) practices in England, for whom there was at least 1 other individual living in the household who was willing to report illness to the index person Exclusion criteria: patients with severe mental problems (e.g. major uncontrolled depression or schizophrenia, dementia, or severe mental impairment) or who were terminally ill, and those reporting a skin complaint that would restrict hand‐washing	
Interventions	Automated web‐based intervention that maximised hand‐washing intention, monitored hand‐washing behaviour, provided tailored feedback, reinforced helpful attitudes and norms, and addressed negative beliefs. Control no access to intervention web pages. See Table 4 for details.	
Outcomes	The primary outcome was the number of index individuals that reported 1 or more RTIs (including ILI) at 16 weeks. Secondary: duration of symptoms, transmission of respiratory infections, gastrointestinal infections, attendance at the practice, and use of health service resources Infections self‐reported by participants. RTI defined as 2 symptoms of an RTI for at least 1 day or 1 symptom for 2 consecutive days. Definition of ILI was a high temperature (feeling very hot or very cold; or measured temperature > 37.5 °C), a respiratory symptom (sore throat, cough, or runny nose), and a systemic symptom (headache, severe fatigue, severe muscle aches, or severe malaise). No safety outcomes reported.	
Notes	Government funded. The study was funded by the Medical Research Council (study number 09/800/22). Declaration of interests: the authors declare no competing interests.	
***Risk of bias***		
**Bias**	**Authors' judgement**	**Support for judgement**
Random sequence generation (selection bias)	Unclear risk	Participants were automatically randomly assigned by the intervention software, but sequence generation not described.
Allocation concealment (selection bias)	Low risk	Participants were automatically randomly assigned by the intervention software.
Blinding of participants and personnel (performance bias) All outcomes	High risk	Unblinded study
Blinding of outcome assessment (detection bias) All outcomes	High risk	Unblinded study
Incomplete outcome data (attrition bias) All outcomes	High risk	High attrition that was different in the 2 groups
Selective reporting (reporting bias)	Low risk	Specified outcomes reported.

**Table:** Loeb 2009

***Study characteristics***		
Methods	Open non‐inferiority RCT carried out to compare the surgical mask with the N95 respirator in protecting healthcare workers against influenza. The trial was carried out between 2008 (enrolment started in September and follow‐up on 12 January 2009) and 23 April 2009 (when all HCWs caring for febrile patients were told to wear an N95 respirator) because of the appearance of novel A/H1N1). The trial trigger was the beginning of the influenza season, defined as isolation of 2 or more viruses in a district in the same week. Following the 2003 SARS outbreak, all Ontario nurses caring for febrile patients (38 °C or more and new onset cough or SOB) had to wear surgical masks. The randomisation (carried out in blocks of 4 by centre) then consisted of either confirmation to same‐maker surgical mask wear or N95 respirator wear. Investigators and laboratory staff were blind to allocation status, but for obvious reasons (the visible difference in interventions), participants were unblinded. “The criterion for non‐inferiority was met if the lower limit of the 95% confidence interval (CI) for the reduction in incidence (N95 respirator minus surgical group) was greater than ‐9%”. So this is the non‐inferiority margin. It is assumed that the “minus surgical group” means minus surgical mask group.	
Participants	Consenting nurses (n = 446 randomised) aged a mean of 36.2 years working full time (≥ 37 hours/week) in 23 acute units (a mix of paediatric, A&E, and acute medical units) in 8 hospitals in Ontario, Canada. 225 were randomised to the surgical mask and 221 to the N95 respirator. There were 13 and 11 dropouts, respectively from each arm (all accounted for), plus 21 and 19 lost to follow‐up; 11 in each arm gave no reason, the others are accounted for. There were no deaths. The final total of 212 and 210 was included in the analysis. Table 1 reports the demographic data of participants by arm, which appear comparable.	
Interventions	Surgical masks (as standard wear by the standard distributor) or fit–tested N95 respirator. All nurses wore gloves or gowns in the presence of a febrile patient. See Table 4 for details.	
Outcomes	Laboratory RT‐PCR paired sera with 4‐fold antibody rise from baseline (only for unvaccinated) nurses Effectiveness: follow‐up (lasting a mean of around 97 days for both arms) was carried out twice‐weekly on a web‐based instrument. Nurses with new symptoms were asked to swab a nostril if any of the following signs or symptoms had developed: fever (temperature ≥ 38 °C), cough, nasal congestion, sore throat, headache, sinus problems, muscle aches, fatigue, earache, ear infection, or chills. The text defines influenza with laboratory confirmation, and separately reports criteria for swab triggering and a definition of ILI (“Influenza‐like illness was defined as the presence of cough and fever: a temperature ≥ 38°C"). But this is not formally linked to influenza in the text, as it appears that primary focus was the detection of laboratory‐confirmed influenza (either by RT‐PCR or serology). Additional outcome data sought were work‐related absenteeism and physician visits for respiratory illness. Secondary outcomes included detection of the following non‐influenza viruses by PCR: parainfluenza virus types 1, 2, 3, and 4; respiratory syncytial virus types A and B; adenovirus; metapneumovirus; rhinovirus‐enterovirus; and coronaviruses OC43, 229E, SARS, NL63, and HKU1. Audits to assess nurse compliance with the interventions were carried out in the room of each patient cared for. The text reports that 50 and 48 nurses in the surgical mask and N95 groups, respectively, had laboratory confirmation of influenza infection, indicating non‐inferiority. Interestingly, non‐inferiority seemed to be applicable both to seasonal viruses and nH1N1 viruses (as 8% and 11.9% were serologically positive to nH1N1). This finding is explained either by seeding or cross reaction with seasonal H1N1. Equivalent conclusions could be drawn for nurses with complete follow‐up. Non‐inferiority was applicable also to other ILI agents identified. None of the 52 individuals with positive isolates met the criteria for ILI. All cases of ILI were confirmed as having influenza (9 and 2 respectively). This means that all the 11 cases of ILI had influenza, but that most of those with a laboratory diagnosis of influenza did not have cough and fever. For example, the text reports that “Of the 44 nurses in each group who had influenza diagnosed by serology, 29 (65.9%) in the surgical mask group and 31 (70.5%) in the N95 respirator group had no symptoms”. By implication, of the 88 nurses with antibody rises, 28 had symptoms of some kind, i.e. two‐thirds were asymptomatic. Absenteeism was 1 versus 39 episodes in the mask versus respirator arms. No episodes of LRTI were recorded. The number of family contacts with ILI were the same for each arm (45 versus 47). Physician visits were similar in both groups. Safety: no AEs are reported	
Notes	The authors conclude that “Among nurses in Ontario tertiary care hospitals, use of a surgical mask compared with a N95 respirator resulted in non‐inferior rates of laboratory‐confirmed influenza”. This a well‐designed and conducted trial with credible conclusions. The only comment is that the focus in the analysis on influenza (symptomatic and asymptomatic) is not well‐described, although the rationale is clear (interruption of transmission). Funding/Support: this study was supported by the Public Health Agency of Canada. Financial disclosures: none reported.	
***Risk of bias***		
**Bias**	**Authors' judgement**	**Support for judgement**
Random sequence generation (selection bias)	Unclear risk	"Randomisation was performed centrally ....", but method of sequence generation not described.
Allocation concealment (selection bias)	Low risk	"...by an independent clinical trials coordinating group such that investigators were blind to the randomisation procedure and group assignment and was stratified by centre in permuted blocks of 4 participants."
Blinding of participants and personnel (performance bias) All outcomes	High risk	"It was not possible to conceal the identity of the N95 respirator or the surgical mask since manipulating these devices would interfere with their function"
Blinding of outcome assessment (detection bias) All outcomes	Low risk	Outcome assessment blinded: "Laboratory personnel conducting hemagglutinin inhibition assays, polymerase chain reaction (PCR), and viral culture for influenza were blinded to allocation."
Incomplete outcome data (attrition bias) All outcomes	Low risk	21 of 225 randomised to mask group and 19 of 221 randomised to N95 group were lost to follow‐up, reasons reported. Study stopped early: Quote: We had planned to stop the study at the end of influenza season. However, because of the 2009 influenza A(H1N1) pandemic, the study was stopped on April 23, 2009, when the Ontario Ministry of Health and Long‐Term Care recommended N95 respirators for all healthcare workers taking care of patients with febrile respiratory illness."
Selective reporting (reporting bias)	Low risk	All outcomes reported.

**Table:** Longini 1988

***Study characteristics***		
Methods	Cluster‐controlled, double‐blind, randomised trial to assess the efficacy of virucidal tissues in interrupting family transmission of rhinovirus and influenza virus. The study was carried out in the community of Tecumseh, Michigan, USA during the period of 25 November 1984 to 28 April 1985. However, the authors only report results for the period of 13 January to 23 March 1985, when a high circulation of influenza A H3N2 and rhinovirus was detected.	
Participants	296 households were enrolled, but 5 households were eliminated from the analysis for "technical reasons". The analysis was carried out in households with 3 to 5 members. The authors report data on 143 households randomised to virucidal tissues and 148 to placebo tissue. The average age in households was around 22, and the difference between arms was not significant. Randomisation was carried out by the sponsor, and tissues were pre‐packed in coded boxes with no other identifying features and delivered to households at the beginning of the study period.	
Interventions	Disposable 3‐layered virucidal tissues (citric and malic acids with sodium lauryl sulphate in the middle layer) or placebo (succinic acid in the middle layer) tissues. They were used to blow the nose and for coughing or sneezing into. Households were also stratified by level of tissue use. Tissue use was significantly higher in the intervention arm (82% versus 71%). See Table 4 for details.	
Outcomes	Laboratory: yes ‐ viral culture from nasal and throat swabs from symptomatic participants Effectiveness: ARI (with a proportion of laboratory‐confirmed diagnosis in non‐randomly chosen participants with symptoms lasting 2 days or more) Follow‐up and surveillance was carried out using a telephone questionnaire. Safety: N/A	
Notes	Risk of bias: high (inappropriate choice of placebo) Note: the authors conclude that virucidal tissues were up to 36.9% effective in preventing transmission of ARIs as measured by secondary attack rates (18.7% versus 11.8%). This finding was not statistically significant, but may well have been affected by the lack of do‐nothing community controls. This a well‐designed, well‐written study despite the unexplained attrition of 5 families, the lack of reporting of cluster coefficients, and the differential in tissue use between the 2 arms, which raises questions about the robustness of double‐blinding. Particularly notable is the discussion on the low generalisability of results from the study from the placebo arm given that even the inert barrier of the tissues is likely to have limited spread. Also, the lengths to which the authors went to obtain allocation concealment and maintenance of double‐blind conditions. Funding: The Kimberly‐Clark Corporation sponsored this research. This research was also partially supported by National Institutes of Health Grant 1‐RO1‐AI22877‐01 and General Clinical Research Center Public Health Research Grant 5‐MO1‐RR000039. Declaration of interests: none declared.	
***Risk of bias***		
**Bias**	**Authors' judgement**	**Support for judgement**
Random sequence generation (selection bias)	Unclear risk	Quote: "Treated and placebo tissues were randomly assigned ..." Sequence generation not reported
Allocation concealment (selection bias)	Low risk	Quote:"Treated and placebo tissues were randomly assigned by the sponsor to 296 participating households stratified by household size, such that roughly half the households would receive treated tissues. Thus, the investigators were unaware of the assignment of treated tissues."
Blinding of participants and personnel (performance bias) All outcomes	Low risk	"Treated and placebo tissues were randomly assigned by the sponsor to the randomly assigned 296 households stratified by household size... The type of tissue was identified by code, and the boxes in which tissues were contained were not marked with any specific identifiers. Therefore, the study was double‐blinded."
Blinding of outcome assessment (detection bias) All outcomes	Low risk	Quote:"The investigators were unaware of the assignment of the treated tissues"
Incomplete outcome data (attrition bias) All outcomes	Unclear risk	296 households eligible. "The final sample used for analysis consisted of 143 households in the treatment group and 148 households in the placebo group."
Selective reporting (reporting bias)	High risk	Quote:"The analysis of secondary spread was restricted to households of three to five members for technical reasons, which eliminated five households." "The two groups were almost identical in composition."

**Table:** Luby 2005

***Study characteristics***		
Methods	Partly double‐blind, cluster‐RCT carried out during 15 April 2002 to 5 April 2003 in Karachi, Pakistan. The trial assessed the effects of mother and child hand‐washing on the incidence of respiratory infections, impetigo (data not extracted), and diarrhoea (data not extracted). Randomisation took place by computer‐generated random numbers in 3 phases. 25 neighbourhoods were assigned to hand‐washing and 11 to standard practice. 300 households were assigned to using antiseptic soap. 300 households were assigned to using plain soap. 306 households were assigned to standard practice. 1523 children younger than 15 years were assigned to using antiseptic soap. 1640 children younger than 15 years were assigned to using plain soap. 1528 children younger than 15 years were assigned to standard practice. Soaps were of identical weight, colour, and smell and were packed centrally with a coded packing case matched to households containing 96 bars. Neither field workers nor participants were aware of the content. Control arm households were visited with the same frequency as intervention household but were given books and pens. Codes were held centrally by the manufacturer and broken after the end of the trial to allow analysis.	
Participants	Householders of slums in Karachi. Of the 1523 children younger then 15 years assigned to using antiseptic soap, 117 dropped out (1 died, 51 were born in, and 65 aged out) = 1406; 504 were aged less than 5. Of 1640 children younger then 15 years assigned to using plain soap, 117 dropped out (3 died, 44 were born in, and 70 aged out) = 1523; 517 were aged less than 5. Of 1528 children younger then 15 years assigned to standard practice, 125 dropped out (3 died, 40 were born in, and 82 aged out) = 1403; 489 were aged less than 5.	
Interventions	Instruction programme and antibacterial soap containing 1.2% triclocarban, or ordinary soap to be used throughout the day by householders, or standard procedure. See Table 4 for details.	
Outcomes	Laboratory: N/A Effectiveness: Number of new respiratory illness per person per week Pneumonia (cough or difficulty in breathing with a respiratory rate of > 60 min in children less than 60 days old, > 50 min in those less than 1 year old, and > 40 min for those aged 1 to 5 years) Follow‐up was weekly with household interview and direct observation. Children aged less than 5 were weighed, and the report presents stratification of results by child weight. Safety: N/A	
Notes	Risk of bias: low (cluster coefficients and analysis by unit of randomisation provided) Note: the authors conclude that "handwashing" neighbourhoods has significantly fewer episodes of respiratory disease than controls (e.g. 50% less cough). "Handwashing" children aged less than 5 had 50% fewer episodes of pneumonia than controls (−65% to −35%). However, there was no difference in respiratory illness between types of soap. The report is confusing, with a shifting focus between children age groups. The impression reading is of an often rewritten manuscript. There is some loss of data (e.g. in the results by weight, i.e. risk group) because of lack of clarity on denominators. Despite this, the trial is a landmark. Funding: most of the funding for this study was provided by Procter and Gamble, manufacturer of Safeguard Bar Soap. The balance of the funding was provided by the Centers for Disease Control and Prevention. Conflict of interest statement: S Luby was supported by the grant from the Procter & Gamble company that funded this study. W Billhimer is an employee of the Procter & Gamble company. The other authors declare that they have no conflict of interest.	
***Risk of bias***		
**Bias**	**Authors' judgement**	**Support for judgement**
Random sequence generation (selection bias)	Low risk	Randomisation took place by computer‐generated random numbers in 3 phases.
Allocation concealment (selection bias)	Low risk	Quote:"One of the investigators (SL) who did not participate in recruiting neighbourhoods or households programmed a spreadsheet to randomly generate the integers of a 1 or a 2. He applied the random numbers sequentially to the list of neighbourhoods. Neighbourhoods with a 1 were assigned to control, and those with a 2 were assigned to handwashing promotion. Random assignment continued until neighbourhoods consisted of at least 600 handwashing promotion households and 300 control households were assigned."
Blinding of participants and personnel (performance bias) All outcomes	Low risk	Quote:"The antibacterial soap ... contained 1‐2% triclocarban as an antibacterial substance. The plain soap was identical to the antibacterial soap except that it did not contain triclocarban... . Neither the fieldworkers nor the families knew whether soaps were antibacterial or plain."
Blinding of outcome assessment (detection bias) All outcomes	Low risk	Quote:"Neither the fieldworkers nor the families knew whether soaps were antibacterial or plain."
Incomplete outcome data (attrition bias) All outcomes	Unclear risk	89% of the study population followed up, but no data on the clusters.
Selective reporting (reporting bias)	Low risk	Quote:"At baseline, households in the three intervention groups were similar."

**Table:** MacIntyre 2009

***Study characteristics***		
Methods	Prospective cluster‐RCT carried out in Sydney, Australia, to assess the use of surgical masks, P2 masks, and no masks in preventing ILI in households. The study was carried out during the 2 winter seasons of 2006 and 2007 (August to the end of October 2006 and June to the end of October 2007). “Gaussian random effects were incorporated in the model to account for the natural clustering of persons in households"	
Participants	290 adults from 145 families. 47 households (94 enrolled adults and 180 children) were randomised to the surgical mask group, 46 (92 enrolled adults and 172 children) to the P2 mask group, and 52 (104 enrolled adults and 192 children) to the no‐mask (control) group.	
Interventions	Use of surgical masks and P2 mask versus no mask. The P2 mask is described as very cumbersome. See Table 4 for details.	
Outcomes	Laboratory: serological evidence Effectiveness: ILI (described as fever, history of fever or feeling feverish in the past week, myalgia, arthralgia, sore throat, cough, sneezing, runny nose, nasal congestion, headache) However, a positive laboratory finding for influenza converts the ILI definition into one of influenza. Safety: N/A	
Notes	The study authors conclude that adherence to mask use significantly reduced the risk for ILI‐associated infection, but < 50% of participants wore masks most of the time. They concluded that household use of face masks is associated with low adherence and is ineffective for controlling seasonal respiratory disease. Compliance was by self‐report, therefore likely to be an underestimate. The primary outcome was ILI or lab‐positive illness. This showed no effect. Sensitivity analysis by adherence showed that under the assumption that the incubation period is equal to 1 day (the most probable value for the 2 most common viruses isolated, influenza (21) and rhinovirus (26)), adherent use of P2 or surgical masks significantly reduces the risk for ILI infection, with a hazard ratio = 0.26 (95% CI 0.09 to 0.77; P = 0.015). No other covariate was significant. Under the less likely assumption that the incubation period is equal to 2 days, the quantified effect of complying with P2 or surgical mask use remains strong, although borderline significant; hazard ratio was 0.32 (95% CI 0.11 to 0.98; P = 0.046). The study was underpowered to determine if there was a difference in efficacy between P2 and surgical masks (Table 5). The study conclusion appears to be a post hoc data exploration. Regardless of this, the study message is that respirator use in a family setting is unlikely to be effective as compliance is difficult unless there is a situation of real impending risk. Funding: the Office of Health Protection, Department of Health and Ageing, Australia, 3M Australia, and Medical Research Council (UK). Disclosure: Simon Cauchemez, PhD; Dominic E. Dwyer, BSc(Med), MBBS, FRACP, FRCPA, MD; Holly Seale, BSc, PhD; Pamela Cheung, RN; Gary Browne, MBBS; James Wood, BSc, PhD; and Zhanhai Gao, BSc, MSc, PhD, have disclosed no relevant financial relationships. C. Raina MacIntyre, MBBS, FRACP, FAFPHM, M App Epid, PhD, has disclosed that she has received grants for clinical research from 3M. Michael Fasher, MBBS, PhD, has disclosed that he has received grants for educational activities from and has served as an advisor or consultant to GlaxoSmithKline. Robert Booy, MBBS, FRACP, FRCPCH, MSc, MD, has disclosed that he has received grants for clinical research and educational activities from, and has served as an advisor or consultant to, CSL, Roche, Sanofi, GlaxoSmithKline, and Wyeth. All funding received is directed to a research account at The Children’s Hospital at Westmead, Sydney, Australia, and is not personally accepted by Dr. Booy. Neil Ferguson, FmedSci, DPhi, has disclosed that he has served as an advisor or consultant to Crucell Inc.	
***Risk of bias***		
**Bias**	**Authors' judgement**	**Support for judgement**
Random sequence generation (selection bias)	Unclear risk	"Participating households were randomised to 1 of 3 arms by a secure computerised randomisation process", but sequence generation not described.
Allocation concealment (selection bias)	Unclear risk	Not reported
Blinding of participants and personnel (performance bias) All outcomes	High risk	"Study participants and trial staff were not blinded, as it is not technically possible to blind the mask type to which participants were randomised."
Blinding of outcome assessment (detection bias) All outcomes	Low risk	"However, laboratory staff were blinded to the arm of randomisation."
Incomplete outcome data (attrition bias) All outcomes	Low risk	143 of 145 randomised families were analysed; 2 families in the control group were lost to follow‐up during the study, for which no reasons were given.
Selective reporting (reporting bias)	Low risk	No differences between groups at baseline

**Table:** MacIntyre 2011

***Study characteristics***		
Methods	A cluster‐RCT of 1441 HCWs in 15 Beijing hospitals was performed during the 2008 to 2009 winter. Participants wore masks or respirators during the entire work shift for 4 weeks. Outcomes included CRI, ILI, laboratory‐confirmed respiratory virus infection, and influenza. A convenience no‐mask ⁄ respirator group of 481 health workers from 9 hospitals was compared.	
Participants	Participants (N = 1441) were hospital HCWs aged > 18 years from the emergency departments and respiratory wards of 15 hospitals. These wards were selected as high‐risk settings in which repeated and multiple exposures to respiratory infections are expected. Participants were randomised to medical mask (N = 492 staff from 5 hospitals), N95 fit‐tested masks (N = 461 staff from 5 hospitals), and N95 non‐fit‐tested mask (N = 488 staff from 5 hospitals).	
Interventions	Fit‐tested N95 respirators versus non‐fit‐tested N95 respirators versus medical masks. See Table 4 for details.	
Outcomes	Clinical respiratory illness, defined as 2 or more respiratory symptoms or 1 respiratory symptom and a systemic symptom Influenza‐like illness, defined as fever ≥ 38 °C plus 1 respiratory symptom (i.e. cough, runny nose, etc.) Laboratory‐confirmed viral respiratory infection (detection of adenoviruses, human metapneumovirus, coronavirus 229E ⁄ NL63, parainfluenza viruses 1, 2, and 3, influenza viruses A and B, respiratory syncytial virus A and B, rhinovirus A or B, and coronavirus OC43/HKU1 by multiplex PCR) Laboratory‐confirmed influenza A or B Adherence with mask or respirator use. Reported problems associated with using the masks or respirators	
Notes	Control arm not randomised so has been ignored. Funding source unknown. Conflict of interests: Raina MacIntyre receives funding from influenza vaccine manufacturers GSK and CSL Biotherapies for investigator‐driven research. She has also been on advisory boards for Wyeth, GSK and Merck. Dr Simon Cauchemez received consulting fees from MacIntyre et al. 178 ª 2011 Blackwell Publishing Ltd, Influenza and Other Respiratory Viruses, 5, 170–179 Sanofi‐Pasteur MSD on the modelling of varicella zoster virus. The remaining authors declare that they have no competing interests. The corresponding author had full access to all the data in the study and had final responsibility for the decision to submit for publication. Prior to the start of this study, NMF acted as a consultant for Roche, Novartis and GSK Biologicals (ceasing in 2007).	
***Risk of bias***		
**Bias**	**Authors' judgement**	**Support for judgement**
Random sequence generation (selection bias)	Unclear risk	Randomisation process (using a secure computerised randomisation program), but sequence generation not described
Allocation concealment (selection bias)	Low risk	Hospitals randomised prior to inclusion of participants.
Blinding of participants and personnel (performance bias) All outcomes	High risk	Unblinded study
Blinding of outcome assessment (detection bias) All outcomes	High risk	Unblinded study
Incomplete outcome data (attrition bias) All outcomes	Low risk	No loss to follow‐up
Selective reporting (reporting bias)	Low risk	Specified outcomes reported.

**Table:** MacIntyre 2013

***Study characteristics***		
Methods	A cluster‐RCT	
Participants	A total of 1669 nurses and doctors from 68 emergency departments and respiratory wards of 19 Beijing hospitals were included. Inclusion criteria: any nurse or doctor aged 18 years or older who worked full time in the emergency or respiratory wards was eligible. Exclusion: HCWs if they (1) were unable or refused to consent; (2) had beards, long moustaches, or long facial hair stubble; (3) had a current respiratory illness, rhinitis, and/or allergy; or (4) worked part time or did not work in the aforementioned wards or departments Final analysis was performed on 572 staff and 24 wards in medical mask group, 516 staff and 20 wards in the targeted N95 mask group, and 581 staff and 24 wards in the N95 mask group.	
Interventions	Quote: "Masks used in the study were the 3M Standard Tie‐On Surgical Mask (catalog number mask 1817; 3M, St. Paul, MN) and the 3M Health Care N95 Particulate Respirator (catalog number 1860; 3M)... . Participants wore the mask or respirator on every shift after being shown how to fit and wear it. Participants were supplied daily with either three masks for the medical mask arm or two N95 respirators. Participants using N95 respirators underwent a fit testing procedure using a 3M FT‐30 Bitrex Fit Test Kit according to the manufacturer’s instructions (3M)." See Table 4 for details.	
Outcomes	Laboratory: Laboratory‐confirmed viral respiratory infection in symptomatic participants, defined as detection of adenoviruses; human metapneumovirus; coronaviruses 229E/NL63 and OC43/HKU1; parainfluenza viruses 1, 2, and 3; influenza viruses A and B; respiratory syncytial viruses A and B; or rhinoviruses A/B by nucleic acid testing (NAT) using a commercial multiplex polymerase chain reaction (Seegen, Inc., Seoul, Korea). Laboratory‐confirmed influenza A or B in symptomatic participants. Laboratory‐confirmed bacterial colonisation in symptomatic participants, defined as detection of *Streptococcus pneumoniae*, *Legionella*, *Bordetella pertussis*, chlamydia, *Mycoplasma pneumoniae*, or *Haemophilus influenzae* type B by multiplex polymerase chain reaction (Seegen, Inc.). Effectiveness: CRI, defined as 2 or more respiratory symptoms or 1 respiratory symptom and a systemic symptom. ILI, defined as fever (38 °C) plus 1 respiratory symptom Safety: adverse effects measured using a semi‐structured questionnaire. Investigators stated that there was higher reported adverse effects and discomfort of N95 respirators compared with the other 2 arms. In terms of comfort, 52% (297 of 571) of the medical mask arm reported no problems, compared with 62% (317 of 512) of the targeted arm and 38% (217 of 574) of the N95 arm (P < 0.001).	
Notes	Compliance with the product was highest in the targeted N95 arm (82%; 422 of 516), then the medical mask arm (66%; 380 of 572), and the N95 arm (57%; 333 of 581); these differences were statistically significant (P < 0.001). The period study conducted: 28 December 2009 to 7 February 2010 Funding: unclear Declaration of interests: none declared.	
***Risk of bias***		
**Bias**	**Authors' judgement**	**Support for judgement**
Random sequence generation (selection bias)	Unclear risk	"using a secure computerized randomization program", but sequence generation not described
Allocation concealment (selection bias)	Unclear risk	No information provided.
Blinding of participants and personnel (performance bias) All outcomes	Low risk	Outcome was objectively assessed with lab confirmation in addition to clinical illness.
Blinding of outcome assessment (detection bias) All outcomes	Low risk	"Laboratory outcomes are reported for all subjects (with at least one respiratory symptom or fever) tested, and then for the subset meeting the CRI definition"
Incomplete outcome data (attrition bias) All outcomes	Low risk	No loss to follow‐up. Flow chart and text match, investigators conducted ITT and PP analysis. All the outcomes were accounted for amongst all participants.
Selective reporting (reporting bias)	Low risk	All outcomes were reported as planned.

**Table:** MacIntyre 2015

***Study characteristics***		
Methods	A cluster‐RCT of cloth masks compared with medical masks in healthcare workers in 14 secondary‐/tertiary‐level hospitals in Hanoi, Vietnam. Hospital wards were randomised to: medical masks, cloth masks, or a control group (usual practice, which included mask wearing). Participants used the mask on every shift for 4 consecutive weeks.	
Participants	1607 hospital HCWs aged ≥ 18 years working full time in selected high‐risk wards. Medical mask group (n = 580 HCWs), cloth mask group (n = 569 HCWs), control group (n = 458 HCWs)	
Interventions	Medical masks, cloth masks, or a control group. See Table 4 for details.	
Outcomes	Clinical respiratory illness, influenza‐like illness, and laboratory‐confirmed respiratory virus infection Clinical respiratory illness, defined as 2 or more respiratory symptoms or 1 respiratory symptom and a systemic symptom Influenza‐like illness, defined as fever ≥ 38 °C plus 1 respiratory symptom Laboratory‐confirmed viral respiratory infection. Laboratory confirmation was by nucleic acid detection using multiplex reverse transcriptase PCR (RT‐PCR) for 17 respiratory viruses. Adverse events associated with mask use	
Notes	Government funded. Competing interests: CRM has held an Australian Research Council Linkage Grant with 3M as the industry partner, for investigator‐driven research. 3M has also contributed masks and respirators for investigator‐driven clinical trials. CRM has received research grants and laboratory testing as in‐kind support from Pfizer, GSK and Bio‐CSL for investigator‐driven research. HS had a NHMRC Australian‐based Public Health Training Fellowship at the time of the study (1012631). She has also received funding from vaccine manufacturers GSK, bio‐CSL and Sanofi Pasteur for investigator‐driven research and presentations. AAC used filtration testing of masks for his PhD thesis conducted by 3M Australia.	
***Risk of bias***		
**Bias**	**Authors' judgement**	**Support for judgement**
Random sequence generation (selection bias)	Low risk	Epi info V.6 was used to generate a randomisation allocation.
Allocation concealment (selection bias)	Low risk	74 wards randomised prior to recruitment of individuals.
Blinding of participants and personnel (performance bias) All outcomes	High risk	Unblinded study
Blinding of outcome assessment (detection bias) All outcomes	High risk	Unblinded study
Incomplete outcome data (attrition bias) All outcomes	Low risk	No loss to follow‐up
Selective reporting (reporting bias)	Low risk	Specified endpoints reported.

**Table:** MacIntyre 2016

***Study characteristics***		
Methods	Cluster‐RCT to examine medical mask use as source control for people with respiratory illness in 6 major hospitals in 2 districts of Beijing, China. Index cases with ILI were randomly allocated to medical mask (n = 123) and control arms (n = 122). Since 43 index cases in the control arm also used a mask during the study period, an as‐treated post hoc analysis was performed by comparing outcomes amongst household members of index cases who used a mask (mask group) with household members of index cases who did not use a mask (no mask group).	
Participants	245 index cases with ILI (medical mask = 123, control group = 122) and 597 household contacts (medical mask = 302, control group = 295)	
Interventions	Medical mask versus no mask (control). See Table 4 for details.	
Outcomes	Clinical respiratory illness, ILI, and laboratory‐confirmed viral respiratory infection Clinical respiratory illness, defined as 2 or more respiratory symptoms (cough, nasal congestion, runny nose, sore throat, or sneezes) or 1 respiratory symptom and a systemic symptom (chill, lethargy, loss of appetite, abdominal pain, muscle or joint aches). ILI, defined as fever ≥ 38 °C plus 1 respiratory symptom. Laboratory‐confirmed viral respiratory infection, defined as detection of adenoviruses, human metapneumovirus, coronaviruses 229E/NL63 and OC43/HKU1, parainfluenza viruses 1, 2, and 3, influenza viruses A and B, respiratory syncytial virus A and B, or rhinovirus A/B by nucleic acid testing using a commercial multiplex PCR. No safety outcomes reported.	
Notes	Government funded. Competing interests: all authors have completed the Unified Competing Interests form (available on request from the corresponding author) and declare that: CRM has held an Australian Research Council Linkage Grant with 3M as the industry partner, for investigator driven research. 3M have also contributed supplies of masks and respirators for investigator‐driven clinical trials. She has received research grants and laboratory testing as in‐kind support from Pfizer, GSK and Bio‐CSL for investigator‐driven research. HS had an NHMRC Australian based Public Health Training Fellowship at the time of the study (1012631). She has also received funding from vaccine manufacturers GSK, bio‐CSL and Saniofi Pasteur for investigator‐driven research and presentations. AAC had testing of filtration of masks by 3M for PhD.	
***Risk of bias***		
**Bias**	**Authors' judgement**	**Support for judgement**
Random sequence generation (selection bias)	Low risk	Random allocation sequence using Microsoft Excel
Allocation concealment (selection bias)	High risk	Doctors enrolled the participants randomly to intervention and control arms.
Blinding of participants and personnel (performance bias) All outcomes	High risk	Unblinded study
Blinding of outcome assessment (detection bias) All outcomes	High risk	Clinical endpoints assessed unblinded.
Incomplete outcome data (attrition bias) All outcomes	Low risk	No loss to follow‐up
Selective reporting (reporting bias)	Low risk	Specified outcomes reported.

**Table:** McConeghy 2017

***Study characteristics***		
Methods	Pilot study of comprehensive intervention (education, cleaning of surfaces, audit and feedback) to staff of nursing homes versus usual care. Pair‐matched cluster‐randomised design with only 5 clusters (nursing homes) in each group	
Participants	10 nursing homes in Colorado, USA Intervention group = 481 long‐stay residents and control group = 380 'Long‐stay' defined as resident at least 90 days prior to baseline, or recently readmitted after previous long stay.	
Interventions	A multifaceted hand‐washing/surface‐cleaning intervention comprised of 1) 1‐hour online educational module focused on how to prevent infections; 2) provided with an “essential bundle” of 7 products, ranging from hand sanitiser gel and foam to antiviral facial tissues, disinfecting spray, and hand and face wipe and recommendation to use 4 skin cream and wipe products; 3) audit and feedback system. See Table 4 for details.	
Outcomes	Laboratory: surface cultures mentioned in Methods, but no results given Effectiveness: LRTI, all infections, hospitalisation, use of antibiotics (not relevant to this review) Safety: none mentioned in Methods and no results given	
Notes	The authors conclude that Quote: “This multifaceted hand‐washing and surface cleaning intervention was designed to reduce infection rates among nursing homes residents. In our 10‐facility randomized, matched pair pilot study, we observed program compliance and satisfaction along with reductions in surface bacterial counts, but did not observe a statistically significant reduction in infection rates, antimicrobial use, or hospitalizations”. Very poorly reported study with results not explained, summarised in Table 3 as RDs. Denominators and attrition are unclear. This work was supported by Kimberly‐Clark Corporation (Contract # 14792008). Declaration of interests: none declared.	
***Risk of bias***		
**Bias**	**Authors' judgement**	**Support for judgement**
Random sequence generation (selection bias)	Unclear risk	Method not described
Allocation concealment (selection bias)	Unclear risk	Method not described
Blinding of participants and personnel (performance bias) All outcomes	High risk	Unblinded
Blinding of outcome assessment (detection bias) All outcomes	High risk	Illness and absenteeism reported by treating staff.
Incomplete outcome data (attrition bias) All outcomes	Unclear risk	No attrition given. Data were collected from e‐medical record at baseline, but not clear whether illness data during the study were collected by the same method.
Selective reporting (reporting bias)	High risk	Upper respiratory tract infection was mentioned in the Methods (intervention presumably would target these), but only LRTI and overall infection reported.

**Table:** Millar 2016

***Study characteristics***		
Methods	Cluster‐RCT, open‐label study, factorial design	
Participants	Around 30,000 healthy, male army trainees aged 18 to 42 years at Fort Benning, Georgia were included. Inclusion criteria: trainees assigned to 1 of the 6 selected training battalions, trainees who present with an SSTI at the clinic or the hospital, provide informed consent. Exclusion criteria: fails to meet inclusion criteria. No denominator breakdown by arm is reported.	
Interventions	Promotion of hand‐washing in addition to a once‐weekly application of chlorhexidine‐based body wash. See Table 4 for details.	
Outcomes	This study was nested in a large field‐based RCT and utilised clinic‐based medical records. Laboratory: none Effectiveness: incidence of ARI at 20 months. The case definition was any occurrence of the following ICD‐9 symptom or disease‐specific codes: 460 to 466, 480 to 488, and specifically 465.9, 482.9, 486, and 487.1. Safety: adverse effects neither planned nor reported by the investigators	
Notes	The period study conducted: May 2010 to January 2012 Government funded. Declaration of interests: none declared.	
***Risk of bias***		
**Bias**	**Authors' judgement**	**Support for judgement**
Random sequence generation (selection bias)	Low risk	quote: "computer‐generated random numbers to 1 of the 3 study groups"
Allocation concealment (selection bias)	Unclear risk	No information provided.
Blinding of participants and personnel (performance bias) All outcomes	High risk	The study was open‐label and self‐reporting of ARI. It is planned as secondary objective of an original trial. Data abstractors were blinded to group assignment.
Blinding of outcome assessment (detection bias) All outcomes	Low risk	Data abstractors were blinded to group assignment.
Incomplete outcome data (attrition bias) All outcomes	High risk	There is a statistically significant difference between attrition rates in the 3 groups. The reasons for attrition are briefly reported in Table 1 of the original study (Ellis and colleagues 2014), but are unlikely to be related to the outcomes of this study. ARI cases were captured utilising clinic‐based medical records, but this outcome is not prespecified in the protocol.
Selective reporting (reporting bias)	High risk	The study was conducted for another purpose. According to the study protocol, the outcomes of interest in the current report were not mentioned as outcomes when the study was planned. ARI is not prespecified as an outcome in the protocol published on ClinicalTrials.gov.

**Table:** Miyaki 2011

***Study characteristics***		
Methods	A quasi‐cluster‐RCT	
Participants	A total of 15,134 assigned to intervention (N = 6634 workers) and control (N = 8500 workers) Inclusion criteria: all general employees (aged 19 to 72 years in 2009) of 2 sibling companies of a major car industry in Kanagawa Prefecture, Japan*.* All workers who regularly reported to the workplace were included, regardless of treatment for chronic diseases*.* All employees have the same health insurance plan and were followed up in the same way.	
Interventions	Quote: "The intervention involved asking workers whose family members developed an influenza‐like illness (ILI) to stay at home. If any co‐habiting family members showed signs of influenza‐like illness (ILI), employees ... were asked to stay at home voluntarily until 5 days has passed since the resolution of the ILS symptoms or 2 days after alleviation of fever." See Table 4 for details.	
Outcomes	Workroom: influenza A test kit (rapid test) Effectiveness: assess the effectiveness of household quarantine in reducing the incidence of influenza A H1N1. ILI was defined as a body temperature greater than 38 °C or more than 1 °C above the normal temperature accompanied with more than 2 of these symptoms: nasal mucus, pharyngeal pain, cough, chills or heat sensation Safety: the incidence of influenza A H1N1 amongst workers who were told to stay home if a family member developed ILI was higher (relative risk of 2.17; P < 0.001) compared to control group. No other safety measures/harms reported. Compliance: quote: "our intervention was not compulsory; we only asked the employees to leave the workplace for a while on full pay, and we succeeded in getting all workers’ agreement. In our case, explaining that the home waiting policy might be beneficial to the whole workers and help to avoid stopping the manufacturing lines (explaining it is for the benefit of the public) and guaranteeing payment during the leave (financial support) helped them to obey our request."	
Notes	Period study conducted: 1 July 2009 to 19 February 2010 Unfunded There are no conflicts of interest to declare.	
***Risk of bias***		
**Bias**	**Authors' judgement**	**Support for judgement**
Random sequence generation (selection bias)	Unclear risk	No information given.
Allocation concealment (selection bias)	Unclear risk	No information given.
Blinding of participants and personnel (performance bias) All outcomes	Low risk	The nature of the intervention (stay at home) was confirmed in the intervention group, where all workers agree as they were financially supported during absences due to ILI.
Blinding of outcome assessment (detection bias) All outcomes	Low risk	"Company doctors diagnosed the disease through a positive result of an influenza A test or clinical symptoms", but not clear if they were blinded to assignment; however, the diagnostic process is meticulous and objectively confirmed.
Incomplete outcome data (attrition bias) All outcomes	Low risk	All cases are included in the analysis, and none were lost to follow‐up.
Selective reporting (reporting bias)	Unclear risk	Although all outcomes of interest are clearly specified, described, and followed up, and text and numbers checked out well and based on the outcome stated for the study, there is no published protocol to match the planned vs the reported outcomes.

**Table:** Morton 2004

***Study characteristics***		
Methods	Cross‐over study to evaluate the effectiveness of an alcohol gel as an adjunct to regular hand‐washing for decreasing absenteeism amongst elementary children by reducing specific communicable diseases such cold, flu, and conjunctivitis. The study was conducted in an elementary school in New England, USA. In the cross‐over design, classrooms in each grade level were randomised to begin as the experimental group (alcohol gel) or the control group (regular hand‐washing). A study protocol for hand hygiene was introduced following the germ unit education. The hand‐washing product was a soap‐and‐water alternative that is approximately 60% ethyl alcohol. In phase 1 (46 days) children in 9 classrooms were in the experimental group, and children in 8 classrooms were in the control group. After a 1‐week washout period when no children had access to the alcohol gel, phase 2 (47 days) started, and the classroom that had participated before as experimental group passed into the control group and vice versa. Data were collected by the parents, who informed the secretary or the school nurse of the reasons for a child's absence, including symptoms of any illness. Respiratory illnesses were defined by symptoms of URTI.	
Participants	253 children, 120 girls and 133 boys, from kindergarten to 3rd grade. Of the eligible 285 students, 32 children dropped out (10 due to skin irritation and 22 because of lack of parental consent). No denominator breakdown by arm is reported because the study used a cross‐over design.	
Interventions	Use of an alcohol gel as an adjunct to regular hand‐washing and educational programme versus regular hand‐washing and educational programme. See Table 4 for details.	
Outcomes	Laboratory: no Effectiveness: days of absences from school for respiratory illness Safety: N/A	
Notes	Risk of bias: high (no description of randomisation; partial reporting of outcomes, numerators and denominators) Note: the authors conclude that significantly fewer children became ill whilst using the alcohol gel as an adjunct to regular hand‐washing than when using regular hand‐washing only (decreased school absenteeism of 43% with the use of alcohol gel on top of hand‐washing). The authors also described, as a limitation of the study, the fact that the school nurse served as the data collector, which could be perceived as bias in measurement of the outcome variable. Randomisation and allocation are not described; no cluster coefficients were reported; and attrition was not taken into consideration during the analysis. Unit of randomisation and analysis are different. No reporting by arm. No ORs, no CIs reported. Funding: Maine Administrative School District #35 in Eliot, Maine, and South Berwick, Maine. Conlicts of interest: none declared.	
***Risk of bias***		
**Bias**	**Authors' judgement**	**Support for judgement**
Random sequence generation (selection bias)	Unclear risk	Insufficient information
Allocation concealment (selection bias)	Unclear risk	Insufficient information
Blinding of participants and personnel (performance bias) All outcomes	Unclear risk	Quote: "A cross‐over design was used. In the crossover design, classrooms in each grade level were randomized to begin as the experimental group (regular hand washing)."
Blinding of outcome assessment (detection bias) All outcomes	Unclear risk	Quote:"The school nurse served as the data collector for the duration of the study. This could be perceived as bias in the measurement of the outcome variable, absenteeism related to infectious illness."
Incomplete outcome data (attrition bias) All outcomes	Unclear risk	Insufficient information
Selective reporting (reporting bias)	Unclear risk	Insufficient information

**Table:** Najnin 2019

***Study characteristics***		
Methods	Cluster‐RCT, parallel assignment	
Participants	Residents of the high‐risk, cholera‐prone study areas. Low‐income communities in Mirpur area of urban Dhaka defined by low per capita income, poor sanitation, unsafe water use, sharing of water source, and poor living conditions. 90 geographic clusters were included, with 30‐metre buffer zones. A total of 7842 households, with 52,237 individuals analysed Vaccine‐only area: data were analysed for 1965 households consisting of 13,148 individuals Vaccine‐plus‐behaviour‐change area: data were analysed for 3886 households consisting of 25,566 individuals Control area: data were analysed for 1991 households consisting of 13,523 individuals Study criteria from published protocol: Inclusion criteria: apparently healthy residents of selected vaccination sites, aged 1 year and above, non‐pregnant women, written informed consent Exclusion criteria: age less than 1 year and pregnant women	
Interventions	Hand‐washing and water treatment promotion. See Table 4 for details.	
Outcomes	Laboratory: none used Effectiveness: prevalence of respiratory illness. People were classified as having respiratory illness if they reported having fever plus either cough or nasal congestion or fever plus breathing difficulty in the past 2 days of unannounced home visits: in each intervention group and amongst those who had soap/soapy water with water present in the hand‐washing station (35% of all groups combined) versus those without this (regardless of the intervention group). Planned secondary outcome: prevalence of reported respiratory illness during 2‐year intervention period Safety: no adverse effects planned or reported	
Notes	The period study conducted: 2011 to 2013 Funding: government and private Bill & Melinda Gates Foundation Conlicts of interest: none declared.	
***Risk of bias***		
**Bias**	**Authors' judgement**	**Support for judgement**
Random sequence generation (selection bias)	Low risk	Computer‐generated randomisation sequence was used to allocate 90 geographical clusters to 1 of 3 groups. Before randomisation, clusters were stratified blocked into 2 categories according to the distance to the hospital. (parent article: Lancet. 2015 Oct 3;386(10001):1362‐1371)
Allocation concealment (selection bias)	Unclear risk	No information provided.
Blinding of participants and personnel (performance bias) All outcomes	High risk	All trial participants and investigators were aware of group assignment. Several in and out migrations across all groups before, after, and during outcome monitoring, and large number of changes in intervention areas
Blinding of outcome assessment (detection bias) All outcomes	High risk	Several in and out migrations across all groups before, after, and during outcome monitoring, and large number of changes in intervention areas
Incomplete outcome data (attrition bias) All outcomes	High risk	High migration movement. This could have distorted the baseline characteristics even more. Very hard to assess because the numbers in the index paper are different from the parent paper (Qadri 2015). In addition to that, for each intervention, data were analysed for 15% to 30% of those allocated on start date. Each group started with approximately 80,000 people; the number analysed is much lower (237,216 people were in the study area on start date of outcome monitoring, the total number analysed across all groups was 52,237). No info about data on migrated individuals or on those who changed intervention areas was dealt with? Also data for prevalence of ARI adjusted for age and wealth were not shown. The outcome is addressed in the 2 days preceding an unannounced visit. This means that if there was a respiratory illness in the past week it would not have been reported. Moreover, these monthly unannounced visits were done to a different set of participants in each group!
Selective reporting (reporting bias)	High risk	Published protocol does not include respiratory illness as an outcome.

**Table:** Nicholson 2014

***Study characteristics***		
Methods	Cluster‐RCT	
Participants	70 low‐income communities in Mumbai, India (35 communities per arm) were randomised to intervention arm (N = 1025) and control arm (N = 1026). Households located in low‐income urban communities in west and south Mumbai, India. Each household contains 1 target child in the first year of a municipal school (typically aged 5 years).	
Interventions	Combination of hand‐washing promotion with provision of free soap aimed at 5‐year‐olds with provision of free soap. See Table 4 for details.	
Outcomes	Laboratory: none reported Effectiveness: Primary outcomes: episodes of diarrhoea, ARIs, and school absences amongst target children, and episodes of diarrhoea and ARIs among their families Secondary outcomes: episodes of eye infections, vomiting, abscesses or boils, headaches, and earache Operational deﬁinitions for all the illnesses were taken from *Black’s Medical Dictionary* (MacPherson 1999). ARIs as "pneumonia, cough, fever, chest pain and shortness of breath, cold, inﬂammation of any or all of the airways, that is, nose, sinuses, throat, larynx, trachea and bronchi" Safety: no safety measures planned or reported by the investigators	
Notes	The period study conducted: 22 October 2007 to 2 August 2008 Funding: multinational corporate company (Unilever plc.) Conlicts of interest: none declared.	
***Risk of bias***		
**Bias**	**Authors' judgement**	**Support for judgement**
Random sequence generation (selection bias)	High risk	Coin tossing used, which could have led to a large imbalance.
Allocation concealment (selection bias)	Low risk	"a coin toss was used to assign one community in each pair to intervention and one to control"
Blinding of participants and personnel (performance bias) All outcomes	High risk	Participants knew to which arm they had been recruited. Households were removed from the study if they provided no data for 5 consecutive weeks.
Blinding of outcome assessment (detection bias) All outcomes	High risk	Data collectors were independent of the behaviour change intervention. Each was assigned exclusively to either households in the intervention group or to control households. However, communities, where very low literacy levels exist, were replaced after randomisation.
Incomplete outcome data (attrition bias) All outcomes	High risk	Data for non‐completers were available and similar across groups. ITT and PP were performed. However, households were removed from the study if they provided no data for 5 consecutive weeks.
Selective reporting (reporting bias)	Unclear risk	No information to judge

**Table:** Pandejpong 2012

***Study characteristics***		
Methods	Cluster‐RCT, single study centre	
Participants	Children (total number = 1437) were randomised to alcohol hand gel every 60 minutes (N = 452 children), every 120 minutes (N = 447 children), and once before lunch (N = 540 children). Inclusion criteria: all children in a large private school in suburban Bangkok, Thailand, all ages, both genders with parental consent to participate. Exclusion criteria: an allergy to alcohol hand gel	
Interventions	3 disinfection interventions: Alcohol hand gel applied every 60 minutes vs every 120 minutes vs once before lunch (3 groups). The current school standard for hand hygiene (q lunch group). See Table 4 for details.	
Outcomes	Laboratory: none Effectiveness: Primary: rates of absenteeism from physician‐confirmed ILI Secondary: rate of absenteeism caused by total reported ILI (with and without a doctor’s confirmation) In case the child was sick but did not see a doctor, the parents were asked to report any of the following symptoms: runny nose or cough, fever or chills, sore throat, headache, diarrhoea, and presence of hand, foot, or mouth ulcers. If 2 or more of these symptoms were reported, then the child’s illness was documented as an ILI. Safety: investigators reported that no adverse reaction to the alcohol hand gel was reported in any participants	
Notes	The period study conducted: December 2009 to February 2010 Funding: Royal College of Physicians of Thailand Conflict of interest: none to report	
***Risk of bias***		
**Bias**	**Authors' judgement**	**Support for judgement**
Random sequence generation (selection bias)	Unclear risk	No information provided.
Allocation concealment (selection bias)	Unclear risk	No information provided.
Blinding of participants and personnel (performance bias) All outcomes	Unclear risk	Parents and teachers are aware of the assignment. Teachers were responsible for recording the absenteeism case record forms. Parents would report child sickness. No diagnostic tests, even in the case of physician‐confirmed ILI
Blinding of outcome assessment (detection bias) All outcomes	Low risk	Outcome is physician‐confirmed ILI.
Incomplete outcome data (attrition bias) All outcomes	Low risk	Quote: "No students were lost to follow‐up or discontinued the intervention during the study period."
Selective reporting (reporting bias)	Low risk	All outcomes were reported.

**Table:** Priest 2014

***Study characteristics***		
Methods	A cluster‐RCT	
Participants	Study included children aged 5 to 11 years at 68 primary schools in New Zealand. Schools were randomised to hand sanitiser + education session arm (34 schools and 8859 children) and education session arm (34 schools and 7386 children). Inclusion criteria: School‐level inclusion: at least 100 children of primary school age (school years 1 to 6; children will generally range in age from 5 years to 11 years) at November 2008. Schools that are not currently using hand‐sanitiser products or are willing to not use them for the period of the trial. Schools are within the City boundaries of Christchurch, Dunedin, or Invercargill in New Zealand. The principal of the school consents to the school being included in the trial. Not ‘‘special schools’’ (e.g. schools for children with deafness or disability) and either not currently using hand‐sanitiser products or willing to not use them for the period of the trial if they were randomised to the control group were eligible to participate in the trial. Student‐level inclusion (follow‐up children): children were eligible to participate in the follow‐up group, for whom more detailed information on absences was collected, if they attended a school year 1 to 6 class in 1 of the included schools at the beginning of the second school term in 2009 (the end of April), and their caregivers completed the consent form indicating that they were willing to be telephoned following their child’s absences and that they were able to take part in telephone interviews in English Exclusion criteria: School‐level exclusion: special needs schools Student‐level exclusion (follow‐up children): children of the principal investigators and study personnel of the trial. Or, children of families that the principal of the primary school directs us not to approach	
Interventions	Hand sanitiser provision (in addition to hand hygiene education session also provided to control group) in schoolchildren. See Table 4 for details.	
Outcomes	Laboratory: none Effectiveness: Primary outcome: the incidence rate of absence episodes from school (reported by the parents during telephone calls) due to any illness during the study period (winter term) Secondary outcomes: assessing whether hand sanitiser was effective in reducing the: incidence rate of respiratory illness absence episodes, incidence rate of gastrointestinal illness absence episodes, incidence rate of absence for any reason, length of illness episode, length of illness absence episode, and incidence rate of subsequent illness amongst other children or adults in the household. Definition of respiratory illness: at least 2 of the following caregiver‐reported symptoms for 1 day, or 1 of the following symptoms for 2 days (but not fever alone): runny nose, stuffy or blocked nose or noisy breathing, cough, fever, sore throat, or sneezing Safety: examined whether the use of hand sanitiser was associated with an increased risk of any skin reactions during the intervention period. Skin reactions: dryness, redness, flakiness, itchiness, eczema, and any other skin reactions	
Notes	The period study conducted: 27 April to 25 September 2009 Government funded: Health Research Council of New Zealand Competing Interests: the authors have declared that no competing interests exist. All authors affirm that they are not involved in any other trials on the same or a related intervention.	
***Risk of bias***		
**Bias**	**Authors' judgement**	**Support for judgement**
Random sequence generation (selection bias)	Low risk	Quote: "Stata/MP 10.1 for Windows was used to generate the random numbers"
Allocation concealment (selection bias)	Low risk	Done by trial statistician provided with school codes and district and randomised the schools to either "A" or "B"
Blinding of participants and personnel (performance bias) All outcomes	Low risk	Outcome assessors were blinded to the group allocation until the analysis was completed.
Blinding of outcome assessment (detection bias) All outcomes	Low risk	Outcome assessors were blinded to the group allocation until the analysis was completed.
Incomplete outcome data (attrition bias) All outcomes	Unclear risk	The study flow diagram gives a clear account on follow‐up, with numbers of those lost to follow‐up and those who discontinued the intervention along with the reasons for doing so. No child was excluded from the analysis. Only PP analysis was reported.
Selective reporting (reporting bias)	Low risk	All outcomes stated in the published protocol were reported in the study. The exception was quote: "1 planned secondary outcome (that is irrelevant to our study) that was not collected and 2 collected secondary outcomes that were not planned in the original protocol".

**Table:** Radonovich 2019

***Study characteristics***		
Methods	Cluster‐RCT, multicentre, pragmatic effectiveness trial	
Participants	Study included 280 clusters randomly assigned to N95 respirators (189 clusters and 1993 HCPs) and medical masks (191 clusters and 2058 HCPs). All participants in a cluster worked in the same outpatient clinic or outpatient setting. All participants were permitted to participate for 1 or more years and gave written consent for each year of participation. Inclusion criteria: healthcare workers in outpatient settings serving adult and paediatric patients with a high prevalence of acute respiratory illness. Participants were aged at least 18 years and employed at 1 of the 7 participating health systems, and self‐identified as routinely positioned within 6 feet (1.83 m) of patients. Participants were full‐time employees (defined as direct patient care for approximately ≥ 24 hours weekly) and worked primarily at the study site (defined as ≥ 75% of working hours). Exclusion criteria: medical conditions precluding safe participation or anatomic features that could interfere with respirator fit, such as facial hair or third‐trimester pregnancy. Participants self‐identified race and sex using fixed categories; these variables were collected because facial anthropometrics related to race and sex may influence N95 respirator fit.	
Interventions	Fit‐tested N95 respirators versus medical masks when near patients with respiratory illness. See Table 4 for details.	
Outcomes	Laboratory. Primary outcome: the incidence of laboratory‐confirmed influenza, defined as: detection of influenza A or B virus by RT‐PCR in an upper respiratory specimen collected within 7 days of symptom onset; detection of influenza from a randomly obtained swab from an asymptomatic participant; and influenza seroconversion (symptomatic or asymptomatic), defined as at least a 4‐fold rise in haemagglutination inhibition antibody titres to influenza A or B virus between pre‐season and postseason serological samples deemed not attributable to vaccination. Effectiveness. Secondary outcomes: the incidence of 4 measures of viral respiratory illness or infection as follows: acute respiratory illness with or without laboratory confirmation; laboratory‐detected respiratory infection, defined as detection of a respiratory pathogen by PCR or serological evidence of infection with a respiratory pathogen during the study surveillance period(s), which was added to the protocol prior to data analysis; laboratory‐confirmed respiratory illness, identified as previously described (defined as self‐reported acute respiratory illness plus the presence of at least PCR–confirmed viral pathogen in a specimen collected from the upper respiratory tract within 7 days of the reported symptoms and/or at least a 4‐fold rise from pre‐intervention to postintervention serum antibody titres to influenza A or B virus; and influenza‐like illness, defined as temperature of at least 100 °F (37.8 °C) plus cough and/or a sore throat, with or without laboratory confirmation. Safety: no serious study‐related adverse events were reported. 19 participants reported skin irritation or worsening acne during years 3 and 4 at 1 site in the N95 respirator group.	
Notes	The study was conducted from September 2011 to May 2015, with final follow‐up on 28 June 2016. Compliance: adherence was reported on daily surveys 22,330 times in the N95 respirator group and 23,315 times in the medical mask group. Quote: “Always” was reported 14,566 (65.2%) times in the N95 respirator group and 15,186 (65.1%) times in the medical mask group; “sometimes” 5407 (24.2%) times in the N95 respirator group and 5853 (25.1%) times in the medical mask group; “never” 2272 (10.2%) times in the N95 respirator group and 2207 (9.5%) times in the medical mask group; and “did not recall” 85 (0.4%) times in the N95 respirator group and 69 (0.3%) times in the medical mask group. Participant‐reported adherence could not be assessed in 784 participants (31.2%) in the N95 respirator group and 822 (30.8%) in the medical mask group (P = 0.84) because of lack of response to surveys or lack of adherence opportunities (i.e. participants did not encounter an individual with respiratory signs or symptoms). Analysed post hoc, participant adherence was reported as always or sometimes 89.4% of the time in the N95 respirator group and 90.2% of the time in the medical mask group. Government funded. Conflict of interest disclosures: Dr Bessesen reported receiving grants from the Department of Veterans Affairs during the conduct of the study. Dr Brown reported receiving grants from the US Department of Veterans Affairs during the conduct of the study. Dr Cummings reported receiving grants from the Centers for Disease Control and Prevention, the National Institutes of Health, and MedImmune outside the submitted work and the Biomedical Advanced Research and Development Authority during the conduct of the study. Ms Los reported receiving grants from Centers for Disease Control and Prevention, the Veterans Health Administration, and the Biodefense Advanced Research and Development Agency during the conduct of the study. Dr Gibert reported receiving financial support for the conduct of the study, including research personnel, from the Veterans Health Administration during the conduct of the study. Dr Gorse reported receiving grants from the US Department of Veterans Affairs during the conduct of the study. Dr Nyquist reported receiving grants from the Centers for Disease Control and Prevention/Division of Healthcare Quality Promotion, the National Institute for Occupational Safety and Health, and the Veterans Health Administration during the conduct of the study; personal fees and nonfinancial support from Sequirus outside the submitted work; and serving on a policy making committee regarding infectious disease for the American Academy of Pediatrics Committee on Infectious Diseases. Dr Reich reported receiving grants from Veterans Health Administration during the conduct of the study. Dr Rodriguez‐Barradas reported receiving grants from Veterans Affairs Central Office during the conduct of the study. Dr Perl reported receiving grants from the Centers for Disease Control and Prevention and Biomedical Advanced Research and Development Authority during the conduct of the study and grants from Medimmune outside the submitted work. No other disclosures were reported.	
***Risk of bias***		
**Bias**	**Authors' judgement**	**Support for judgement**
Random sequence generation (selection bias)	Low risk	Computer‐generated random sequences by an individual not involved in the study implementation and data analyses. Used stratified randomisation
Allocation concealment (selection bias)	Low risk	Used constrained randomisation
Blinding of participants and personnel (performance bias) All outcomes	Low risk	The participants cannot be blinded, but it seems that all the measures otherwise were the same with meticulous follow‐up. Besides, the primary outcome was lab based (an objective outcome), which is unlikely to be affected by of lack of blinding. Investigators were blinded to the randomisation until completion of the study and analysis.
Blinding of outcome assessment (detection bias) All outcomes	Low risk	Primary outcome is laboratory‐confirmed diagnosis.
Incomplete outcome data (attrition bias) All outcomes	Low risk	Quote: "Missing outcomes were imputed using standard multiple imputation techniques, creating multiple imputed data sets with no missing values for each analysis"
Selective reporting (reporting bias)	Low risk	Reported study outcomes matched the published protocol. Every outcome was accounted for.

**Table:** Ram 2015

***Study characteristics***		
Methods	RCT	
Participants	377 household compounds (index cases) completed the study. Control arm has 184 compounds with 1607 contacts, and intervention group has 193 compounds with 1814 contacts. Final analysis was performed on 193 index cases and 1661 contacts in the intervention group and 184 index cases and 1498 contacts in the control group. In 2009, index case‐patients with symptom onset within 7 days preceding enrolment were eligible. Eligibility criteria changed in 2010 to include index case‐patient with symptom onset within 48 hours preceding enrolment. Inclusion criteria: Individuals ≥ 5 years old: ILI, defined as history of fever and either cough or sore throat with fever onset within the previous 24 hours. Individuals < 5 years old: any child with acute fever with onset within the previous 24 hours. Return to home within 24 hours of presentation to Upazilla Health Complex, Jahurul Islam Medical College Hospital or the local pharmacies, i.e. the index case cannot be admitted for treatment. If admitted, the patient would not be eligible. No fever in any bari resident during the 7 days preceding the patient's presentation to hospital (see definition below). At least 2 individuals (in addition to the index case‐patient) who intend to reside in the bari during the subsequent 20 days. Residence within 30 minutes travel time (1‐way) from the Upazilla Health Complex or Jahurul Islam Medical College Hospital or the local pharmacy. Exclusion criteria: compounds were excluded if any compound member(s) was reported to have fever within 3 days before index case‐patient enrolment. At another time point, compounds were excluded if any primary household member was reported to have fever (fever occurring within 48 hours prior to enrolment recorded).	
Interventions	Promoting intensive hand‐washing in households to prevent transmission of ILI. See Table 4 for details.	
Outcomes	Laboratory: PCR for influenza A and B, with further subtyping of influenza A isolates for all ILI amongst contacts Effectiveness: incidence of ILI. An age‐based definition of ILI was used as follows. For individuals > 5 years old, ILI was defined as history of fever with cough or sore throat. For children < 5 years old, ILI was defined as fever (the authors used this relatively liberal case definition in order to include influenza cases with atypical presentations in children). Safety: no safety data planned or reported by investigators	
Notes	Inclusion/exclusion criteria changed 3 times during the study conduct. The period study conducted: June 2009 to December 2010 Government funded Competing interests: the authors have declared that no competing interests exist.	
***Risk of bias***		
**Bias**	**Authors' judgement**	**Support for judgement**
Random sequence generation (selection bias)	Low risk	Block randomisation, with a block size of 4, in order to promote random and even allocation of household compounds to the 2 treatment arms. The list of random assignments was generated by an investigator with no contact with the participants.
Allocation concealment (selection bias)	Low risk	Once baseline data collection was complete, the data collector notified the field research officer, who consulted the block randomisation list to make the assignment of the household compound to intervention or control.
Blinding of participants and personnel (performance bias) All outcomes	High risk	Relied on symptom reporting from the head of family. Inclusion/exclusion criteria changed 3 times during the study conduct. Given the provision of a hand‐washing station as part of the intervention, it was not possible to ensure blinding of participants, intervention staff, or data collectors.
Blinding of outcome assessment (detection bias) All outcomes	High risk	Relied on symptom reporting from the head of family. Inclusion/exclusion criteria changed 3 times during the conduct of the study. Given the provision of a hand‐washing station as part of the intervention, it was not possible to ensure blinding of participants, intervention staff, or data collectors.
Incomplete outcome data (attrition bias) All outcomes	Low risk	Flow chart followed all households an individuals from recruitment to analysis.
Selective reporting (reporting bias)	Low risk	The specified outcomes are clearly accounted for Investigators report all outcomes for each modified enrolment.

**Table:** Roberts 2000

***Study characteristics***		
Methods	Open cluster‐RCT carried out between March and November 1996 (the Southern Hemisphere winter season) in 23 childcare centres caring for a minimum of 50 children 10 hours a day, 5 days a week in Australia. The study assessed the effects of an Australian national hand‐washing programme compared to standard procedure. Randomisation was according to a random‐number table, and cluster coefficients are reported.	
Participants	Children (299 in the intervention arm and 259 in the control arm) aged 3 or younger attending the centres at least 3 days a week. Attrition was 51 children in the intervention arm and 72 children in the control arm due mainly to staff leaving the centres.	
Interventions	Hand‐washing programme with training for staff and children. It is unclear whether any extra hand‐cleansing agents were used, as GloGerm (?) is mentioned when it was used in a preliminary study. See Table 4 for details.	
Outcomes	Laboratory: N/A Effectiveness: ARI (runny nose, cough, and blocked nose) Follow‐up was via a parental phone interview every 2 weeks. Safety: N/A	
Notes	Risk of bias: low (cluster coefficients and analysis by unit of randomisation) Note: the authors conclude that although there was no overall decrease in respiratory illness (RR 0.95, 95% CI 0.89 to 1.01), in children up to 24 months the decrease was statistically significant (RR 0.90, 95% CI 0.83 to 0.97). The authors speculated that this was because maximum benefits are likely from this age group due to their limited ability to wipe their nose and hands without a structured programme. Analyses by 3 compliance levels are also reported. A so‐so reported and well‐conducted trial. This work was supported by a grant from the Commonwealth Department of Family Services and Health, Research and Development Scheme. Conflict of interest: none to report.	
***Risk of bias***		
**Bias**	**Authors' judgement**	**Support for judgement**
Random sequence generation (selection bias)	Low risk	Randomisation was according to a random‐number table.
Allocation concealment (selection bias)	Unclear risk	Not reported
Blinding of participants and personnel (performance bias) All outcomes	High risk	It was not possible to blind the intervention.
Blinding of outcome assessment (detection bias) All outcomes	Low risk	Quote: "The observer was not informed of the content of the training sessions or the intervention status of the centres."
Incomplete outcome data (attrition bias) All outcomes	Unclear risk	Recruitment rate 88% (23 of 26 CCCs); loss to follow‐up not clear, as no denominator given
Selective reporting (reporting bias)	Low risk	Centres were comparable at baseline.

**Table:** Sandora 2005

***Study characteristics***		
Methods	Single‐blind, cluster‐RCT carried around the Boston area, USA, in the period of November 2002 to April 2003. The trial tested the effects of using a hand sanitiser and a programme of instruction on the transmissions of GI infections (data not extracted) and ARIs in families. Units of randomisation were childcare centres and were carried out on enrolment by an investigator using random block size generated by computer. Assignment was single‐blind (i.e. investigator blinded to the status of the centre). Cluster correlation was 0.01.	
Participants	292 families with 1 or more children aged 6 months to 5 years who were in child care for 10 or more hours a week 155 children in 14 centres were allocated to the intervention arm and 137 children in 12 centres to the control arm**.** The mean age was 3 to 2.7 years. Attrition was respectively 15 (3 lost to follow‐up and 12 who discontinued the intervention) and 19 (8 lost to follow‐up and 11 who discontinued the intervention). ITT analysis was carried out.	
Interventions	Alcohol‐based hand sanitiser with biweekly hand hygiene educational materials over 5 months versus biweekly educational material on healthy diet. See Table 4 for details.	
Outcomes	Effectiveness: ARI (2 of the following symptoms for 1 day or 1 of the following symptoms for 2 days: runny nose, cough, sneezing, stuffy or blocked nose, fever, sore throat). An illness episode had to be separated by 2 symptom‐free days from a previous episode. A secondary illness was when it followed a similar illness in another family member by 2 to 7 days. Follow‐up was by means of biweekly phone calls to caregivers. Safety: dry skin (71 reports), stinging (11 reports), bad smell (7 reports), dislike (2 reports), allergic reaction (2 reports), slippery feel (1 report), and irritation (20 reports).	
Notes	Risk of bias: low Note: the authors conclude that although the rate of GI illnesses was significantly lower in the intervention group, the IRR was not significantly different for ARIs (0.97, 95% CI 0.72 to 1.30). Compliance and droplet route spread may account for this apparent lack of effect. A well‐reported trial. Study funds and hand sanitiser were provided by GOJO Industries, Inc (Akron, OH). No conflict of interest declared.	
***Risk of bias***		
**Bias**	**Authors' judgement**	**Support for judgement**
Random sequence generation (selection bias)	Low risk	Quote: "Random assignments were generated by computer using a permuted‐blocks design with random block sizes."
Allocation concealment (selection bias)	Low risk	Low riskUnclear riskHigh risk
Blinding of participants and personnel (performance bias) All outcomes	High risk	Quote: "Teachers in the intervention classrooms were responsible for encouraging the use of the disinfecting wipes and hand sanitizer according to the study protocol ... Given that no placebo was provided and sanitizer use was recorded, neither families nor data collectors could be blinded as to the group assignment of the family."
Blinding of outcome assessment (detection bias) All outcomes	High risk	Quote: "Given that no placebo was provided and sanitizer use was recorded, neither families nor data collectors could be blinded as to the group assignment of the family."
Incomplete outcome data (attrition bias) All outcomes	Low risk	Attrition was 15 in intervention arm (3 lost to follow‐up and 12 who discontinued the intervention) and 19 in the control arm (8 lost to follow‐up and 11 who discontinued the intervention). ITT analysis was carried out.
Selective reporting (reporting bias)	Unclear risk	Well‐reported

**Table:** Sandora 2008

***Study characteristics***		
Methods	Cluster‐RCT carried out in a single elementary school system located in Avon, Ohio, USA to assess the effectiveness of a multifactorial infection‐control intervention, including alcohol‐based hand sanitiser and surface disinfection, in reducing absenteeism caused by gastrointestinal and respiratory illnesses amongst elementary school students. The study also aimed to describe the viral and bacterial contamination of common surfaces in the school classroom and to assess the impact of an environmental disinfectant on the presence of selected viruses and bacteria on these surfaces. Clustering was described as "teams of 3‐4 classes depending on the class year”.	
Participants	A total of 363 students in 15 different classrooms were eligible to participate and received letters about the study. A sample of 285 of these students provided written informed consent and were randomly assigned to the intervention group (146) or to the control group (139) and contributed to final analysis. No students were lost to follow‐up or discontinued the intervention during the study period. Baseline demographic characteristics were similar in the intervention and control groups. Most families were white and non‐Hispanic and in excellent or very good health at baseline.	
Interventions	Alcohol‐based hand sanitiser to use at school and quaternary ammonium wipes to disinfect classroom surfaces daily for 8 weeks versus usual hand‐washing and cleaning practices. See Table 4 for details.	
Outcomes	Laboratory: Serological evidence: no Swabs for bacteria and viruses from 3 types of classroom surfaces were taken. Effectiveness: Respiratory illness defined as days absent as measured by a (blinded) school worker who routinely recorded reason for absenteeism either for gastrointestinal or respiratory causes. Safety: N/A	
Notes	The authors conclude that the multifaceted intervention that included alcohol‐based hand sanitiser use and disinfection of common classroom surfaces reduced absenteeism from gastrointestinal illness amongst elementary school students. The intervention did not impact on absenteeism from respiratory illness. In addition, norovirus was detected less frequently on classroom surfaces in the group receiving the intervention. The study is of good quality with low risk of bias. The authors checked compliance by counting discarded wipes. Reasons given for the apparent lack of effect against ARIs but good effect on GI illness are that disinfecting the classroom surfaces (daily at lunchtime with alkali) was important, as were the alcohol wipes. The authors measured the norovirus concentration on surfaces and found this to be reduced. Other reasons may be that droplets are not affected by this method, or that contamination of hands by respiratory infections is likely to be continuous (in orofaecal transmission is mostly at the time of defecation). Study funds, hand‐sanitiser, and disinfecting wipes were provided by The Clorox Company (Oakland, CA). Financial disclosures: Drs Sandora and Goldmann received a consulting fee from The Clorox Company for their efforts in designing and conducting this study; Dr Shihh as indicated she has no financial relationships relevant to this article to disclose.	
***Risk of bias***		
**Bias**	**Authors' judgement**	**Support for judgement**
Random sequence generation (selection bias)	Low risk	Quote: "The allocation sequence was generated by computer ..."
Allocation concealment (selection bias)	Unclear risk	Quote: "...and teams were assigned to study groups by a study investigator (Dr Shih)." Blinding of allocation cannot be guaranteed.
Blinding of participants and personnel (performance bias) All outcomes	High risk	Not possible
Blinding of outcome assessment (detection bias) All outcomes	Low risk	Quote: " All of the students absences were recorded in the usual fashion by the school employee who normally answers this dedicated telephone line. This employee was blinded to the group assignment of the child."
Incomplete outcome data (attrition bias) All outcomes	Low risk	No students were lost to follow‐up or discontinued the intervention during the study period.
Selective reporting (reporting bias)	Unclear risk	Well‐reported

**Table:** Satomura 2005

***Study characteristics***		
Methods	RCT. Randomisation was achieved by simple computer‐generated random digit. Allocation was concealed using sealed, opaque envelopes. Not clear if there was a central randomisation centre. Post hoc exchange of envelopes was prevented by writing both the name of each participant and the number on the envelope he/she drew before breaking the seal. Participants were not blinded to the intervention; however, disease incidence was determined by 1 study physician who was not informed of the results of assignment. Analysis was done based on the intention‐to‐treat principle. The study targeted community healthcare all over Japan and was conducted between December 2002 and March 2003 for a follow‐up period of 60 days.	
Participants	387 participants at 18 sites were recruited, 384 were included in the analysis: water gargling (N = 122), povidone‐iodine gargling (N = 132), and control (N = 130). Follow‐up was completed on 338 participants. Attrition was fully explained for URTI analysis; however, 2 participants were not accounted for in the ILI analysis. 46 participants did not complete the follow‐up due to either discontinuation of diary use (n = 9) or contracting ILI (n = 37). Of the 37 participants with ILI, 11 were in the povidone‐iodine group, 12 in the water group, and 14 in the control group. Analysis was performed on 35 participants (Kitamura 2007 [Kitamura 2007]).	
Interventions	Participants were randomised to 1 of the following: water gargling, n = 122 (20 mL of water for about 15 seconds 3 times consecutively, at least 3 times a day); povidone‐iodine gargling, n = 133 (20 mL of 15 to 30 times diluted 7% povidone‐iodine (as indicated by the manufacturer) in the same way as water gargling); and control, n = 132 (retain their previous gargling habits). All groups were asked to fill a daily gargling diary (standardised form to record: gargling habits, hand‐washing, and influenza complaints). The frequency of gargling in the water group was higher (3.6); the frequency of hand‐washing was similar amongst the 3 groups. URTI symptom was classified according to Jackson methods. Diary recording was continued throughout the follow‐up period and for 1 week after the onset of URTI. ILI was reported separately. See Table 4 for details.	
Outcomes	Laboratory: none Effectiveness: Primary outcome: incidence of first URTI. Index cases were defined as all of the following conditions: both nasal and pharyngeal symptoms, severity of at least 1 symptom increased by 2 grades or more, and worsening of a symptom of 1 increment or more for > 3 days. Secondary outcome: severity of URTI of the incident cases was assessed by grading each symptom during the initial 7 days after the onset of URTI in numeric scores: none = 0, mild = 1, moderate = 2, and severe = 3 ILI was defined as both developing a fever of 38 °C or higher and worsening arthralgia in addition to some respiratory symptoms (Kitamura 2007). Safety: no harm was reported. However, 2 participants in the povidone‐iodine group switched to water gargling (analysed in their assignment group).	
Notes	The authors concluded that simple water gargling is effective in preventing URTIs amongst healthy people. However, no statistically significant difference was observed against ILIs. The study was well‐conducted; blinding would have added to the validity of the results. In addition, the study was not powered enough to detect a statistically significant preventative effect against ILI. The study demonstrates that in addition to hand‐washing, simple gargling even with water can reduce URTI, but not ILI. However, during periods of endemic influenza, multiple inexpensive and simple modalities (hand‐washing, masks, gargling) can be utilised together to reduce infection and transmission. Overall, the reporting of the 2 combined studies together is highly confusing. In the first study (Satomura 2005), the main outcome is URTI defined as fever and arthralgia. The second study (which is a presentation of further data from the 2005 publication in the guise of a short report) introduces the outcome ILI with a definition similar to that of URTI in the first study but referring to the earlier outcome as common cold. Also of note is reporting of significance without confidence intervals. Overall, this potentially important study should be repeated with a larger denominator. Unclear risk of bias because of confused reporting and absence of double‐blinding. Partial financial support was provided by the Suzuken Memorial Foundation (2002) and Uehara Memorial Foundation (2003) (trial registry, ISRCTN67680497). No financial conflict of interest was reported by the authors of this paper.	
***Risk of bias***		
**Bias**	**Authors' judgement**	**Support for judgement**
Random sequence generation (selection bias)	Low risk	Quote: "Group assignment was based on simple computer‐generated random digits..."
Allocation concealment (selection bias)	Low risk	Quote: "By an individual drawing of sealed opaque envelopes, subjects were randomly assigned to the following three groups" Quote: "allocation was completely concealed from study administrators"
Blinding of participants and personnel (performance bias) All outcomes	High risk	Not blinded
Blinding of outcome assessment (detection bias) All outcomes	Low risk	Quote: "To prevent post hoc exchange of the envelopes, local administrators wrote down both the name of each subject and the number on the envelope he/she drew before breaking the seal."
Incomplete outcome data (attrition bias) All outcomes	Low risk	338 of 385 randomised followed up; reasons reported.
Selective reporting (reporting bias)	Unclear risk	Confusing reporting

**Table:** Savolainen‐Kopra 2012

***Study characteristics***		
Methods	Open cluster‐RCT, 3‐arm intervention trial	
Participants	A total of 21 clusters (683 individuals) were randomised to implement hand hygiene with soap and water (257 individuals), alcohol‐based hand rub (202 individuals), or control (224 individuals). The study was conducted in distinct office work units in 6 corporations in the Helsinki Region that together employed some 10,000 staff. All employees (age ≥ 18 years, both genders) were contacted by email survey. Inclusion criteria: quote: "Volunteers working in defined units" Exclusion criteria: quote:"Persons with open wounds or chronic eczema in hands" The designated 21 study clusters were identified as operationally distinct working units, each containing at least 50 people.	
Interventions	Hand hygiene with soap and water and standardised instructions on how to limit the transmission of infections. Usual hand hygiene (control). See Table 4 for details.	
Outcomes	Laboratory: Quote: "Between November 2008 and May 2010, the seven occupational health clinics serving the six participating corporations were advised to collect, using standard techniques, two to three respiratory samples per week from typical RTI patients and also faecal samples from a few representative patients with gastrointestinal symptoms when a GIT outbreak was suspected. The samples could originate from the study participants and also from work units not included in the study. In the laboratory, viral nucleic acids were extracted with well‐characterized commercial kits and tested by validated real‐time PCR methods to detect influenza A and B viruses, respiratory syncytial virus, parainfluenza virus types 1, 2, and 3, adenoviruses, human rhinoviruses and human enteroviruses from respiratory specimens, and norovirus from faecal specimens (detailed descriptions of the test procedures are available from the authors)." Effectiveness: Predefined primary endpoints: Number of reported infection episodes in a cluster per total reported weeks. Number of reported sick leave episodes in a cluster per total reported weeks. Secondary endpoints and outcome measures: Number of days with reported symptoms of RTI and/or GTI in a cluster within a time frame of 100 reporting weeks. Number of days‐off due to own RTI or GTI in a cluster within a time frame of 100 reporting weeks. Safety: reported 0 adverse events	
Notes	The period study conducted: January 2009 to May 2010 Government funded. Competing interests: the authors declare that they have no competing interests.	
***Risk of bias***		
**Bias**	**Authors' judgement**	**Support for judgement**
Random sequence generation (selection bias)	Unclear risk	Insufficient information
Allocation concealment (selection bias)	Low risk	Quote:"clusters were matched and randomized prior to onset of the interventions"
Blinding of participants and personnel (performance bias) All outcomes	High risk	The interventions were not blinded to any party involved (i.e. the study group, participants, or the occupational health services). Subjective reporting of disease episodes
Blinding of outcome assessment (detection bias) All outcomes	High risk	Subjective reporting of disease episodes
Incomplete outcome data (attrition bias) All outcomes	High risk	24% loss to follow‐up. However, new recruiting in most clusters; the total number of reporting participants at the end of the trial was 91.7% compared to that at the beginning. Attrition was reported, and 76% of volunteers who started reporting continued to do so until the end of the study. Because of new recruiting in most clusters, the total number of reporting participants at the end of the trial was 626, or 91.7%, compared to that at the beginning. This means that 15.7% of the participants were replaced during the study!!! Raw data on the effects of the interventions on the occurrence of respiratory infections and vomiting/diarrhoea diseases were not reported. Zero adverse effects were reported.
Selective reporting (reporting bias)	Low risk	All planned outcomes were reported.

**Table:** Simmerman 2011

***Study characteristics***		
Methods	Randomised controlled study	
Participants	Study recruited 348 households and 885 members and randomised them as follows: Control (index household = 119, with 302 family members) Hand‐washing (index household = 119, with 292 family members) Hand‐washing and face mask (index household = 110, with 291 family members) The household members of children (index cases) presenting with ILI at the outpatient department of the Queen Sirikit National Institute of Child Health (QSNICH) in Bangkok, the largest public paediatric hospital in Thailand Inclusion criteria: For index cases: children aged 1 month through 15 years, residents of the Bangkok metropolitan area, and had an onset of illness < 48 hours before respiratory specimens tested positive for influenza by an RIDT that was later confirmed by qualitative real‐time RT‐PCR (rRT‐PCR) Eligible index cases’ households must have had at least 2 other members aged ≥ 1 month who planned to sleep inside the house for a period of at least 21 days from the time of enrolment. Exclusion criteria: For index cases: children at high risk for severe influenza complications (e.g. chronic lung disease, renal disease, and long‐term aspirin therapy) and those treated with influenza antiviral medications Excluded households: those with any member reporting an ILI that preceded the index case by 7 days or less and households where any member had received influenza vaccination during the preceding 12 months	
Interventions	Hand‐washing, or hand‐washing plus paper surgical face mask, or control. See Table 4 for details.	
Outcomes	Laboratory: To identify index cases: QuickVue Influenza A+B rapid diagnostic kit (Quidel Co., San Diego, CA, USA), followed by rRT‐PCR for influenza viral RNA Index cases and contacts tested with nasal swab and throat swab both processed for rRT‐PCR. 2 blood samples for antibody seroconversion collected on Days 1 and 21 (seroconversion defined as a fourfold rise in HI titre between paired sera for any of the antigens assayed). Effectiveness: Laboratory‐confirmed secondary influenza virus infections amongst household members described as the secondary attack rate (SAR). A secondary influenza virus infection was defined as a positive rRT‐PCR result on Days 3 or 7 or a fourfold rise in influenza HI antibody titres with the virus type and subtype matching the index case. SAR for ILI defined by the WHO as fever plus cough or sore throat, based on self‐reported symptoms. Safety: no safety measures planned or reported by the investigators Adherence: participants in the control arm reported an average of 3.9 hand‐washing episodes/day (on Day 7), whilst participants in the hand‐washing arm reported an average of 4.7 hand‐washing episodes/day (95% CI 4.3 to 5.0; P = 0.002 compared to controls), and participants in the hand‐washing plus face mask arm reported 4.9 episodes/day (95% CI 4.5 to 5.3; P < 0.001 compared to controls). In the intervention arms, parents had the highest reported daily hand‐washing frequency (5.7, 95% CI 5.3 to 6.0) followed by others (4.8, 95% CI 4.3 to 5.3), siblings (4.3, 95% CI 3.7 to 4.8), and the index cases (4.1, 95% CI 3.8 to 4.4). There was no difference in the average amount of soap used in a week in the hand‐washing arm (54 mL per person) and the hand‐washing plus face mask arm (58.1 mL per person) (P = 0.15). 289 participants in the hand‐washing plus face mask arm used an average of 12 masks per person per week (median 11, IQR 7 to 16) and reported wearing a face mask a mean of 211 minutes/day (IQR 17 to 317 minutes/day). Parents wore their masks for a median of 153 (IQR 40 to 411) minutes per day, far more than other relations (median 59; IQR 9 to 266), the index patients themselves (median 35; IQR 4 to 197), or their siblings (median 17; IQR 6 to 107). The study authors note that differences in average usage may be an attenuated measure of appropriate use in relation to the actual unmeasured exposure risk such as proximity to the index case.	
Notes	The period study conducted: April 2008 and August 2009 Government funded. BJC has received research funding from MedImmune Inc. No other declarations are reported.	
***Risk of bias***		
**Bias**	**Authors' judgement**	**Support for judgement**
Random sequence generation (selection bias)	Low risk	"Randomization was achieved using a block randomization method using a list of blocks each with 12 household IDs, four of which were assigned to each of the three study arms."
Allocation concealment (selection bias)	Unclear risk	Quote: "A study coordinator assigned each household to one study arm after consent was obtained"
Blinding of participants and personnel (performance bias) All outcomes	Low risk	Recruiting clinicians were blinded to the allocation of the specific intervention. The participants were not blinded, but it is unlikely that the outcome would have been affected by lack of blinding.
Blinding of outcome assessment (detection bias) All outcomes	Low risk	The primary outcome is a laboratory‐confirmed influenza.
Incomplete outcome data (attrition bias) All outcomes	Low risk	Household flow chart provided with reasons for exclusions, all numbers provided. Analysis was done by ITT and PP.
Selective reporting (reporting bias)	Low risk	All outcomes are accounted for in the ITT analysis of the results.

**Table:** Stebbins 2011

***Study characteristics***		
Methods	Cluster‐RCT, open‐label	
Participants	Study included 3360 students from 10 Pittsburgh elementary schools. Intervention arm (5 schools, 1695 people) and control arm (5 schools, 1665 people) No inclusion or exclusion criteria were provided.	
Interventions	Training in hand and respiratory (cough) hygiene. Hand‐sanitiser was provided and encouraged to be used regularly. See Table 4 for details.	
Outcomes	Laboratory: Primary outcome: laboratory‐confirmed influenza (RT‐PCR) amongst children presenting with ILIs leading to their absence from school 2 nasal swabs were obtained using test manufacturer‐approved sterile Dacron swabs. 1 swab was employed for influenza testing using the QuickVue Influenza A+B test (Quidel Corp, San Diego, CA). The second nasal swab was delivered on cold pack to the University of Pittsburgh Medical Center Clinical Virology Laboratory, Pittsburgh, PA for RT‐PCR testing (performed within 48 hours). The RT‐PCR used viral nucleic acid extract (EasyMag; bioMerieux, Durham, NC) and primer/probe sequences for influenza A, influenza B, and influenza A H1 and H3 subtypes (CDC, Atlanta GA). Effectiveness: Secondary outcome: absence episodes and cumulative days of absence due to ILI, any illness, and all causes Safety: none mentioned	
Notes	The period study conducted: 1 November 2007 through 24 April 2008 Funding: this research was supported by Cooperative Agreement number 5UCI000435‐02 from the Centers for Disease Control and Prevention (CDC). DC and DB received support from the NIH MIDAS program (1U01‐GM070708). DC holds a Career Award at the Scientific Interface from the Burroughs Welcome Fund. No other conflicts declared.	
***Risk of bias***		
**Bias**	**Authors' judgement**	**Support for judgement**
Random sequence generation (selection bias)	Low risk	Quote: "constrained randomization algorithm"
Allocation concealment (selection bias)	Low risk	Quote: "Random allocation of schools to two arms was created by Dr. Cummings and concealed until intervention assignment". "At the beginning of the school year parents and guardians were given the opportunity to decline participation"
Blinding of participants and personnel (performance bias) All outcomes	High risk	Unblinded
Blinding of outcome assessment (detection bias) All outcomes	Low risk	In 76% and 78% of illness in intervention and control group were laboratory confirmed. ILI is objectively defined.
Incomplete outcome data (attrition bias) All outcomes	High risk	Only episodes of identified causes were analysed. Causes of absence episodes in 66% of the study participants were not identified (2092 in the intervention group and 2232 in the control group). The parents could be contacted in only 34% cases of absence. About half of them had an illness, and in one‐third of these cases the illness met the criteria of ILI (361 cases (33%)). Of these, 279 (77%) were tested for influenza.
Selective reporting (reporting bias)	Unclear risk	Insufficient information to judge

**Table:** Suess 2012

***Study characteristics***		
Methods	Cluster‐RCT, open‐label, parallel design	
Participants	Study sample included 84 households randomised as follows: 30 control (index cases = 30, household contact = 82) 26 mask group (index cases = 26, household contact = 69) 28 mask and hand hygiene group (index cases = 28, household contact = 67) Inclusion criteria: patients presenting to general practitioners or family physicians at the study sites within 2 days of symptom onset; had a positive rapid antigen test for influenza (later to be confirmed by quantitative RT‐PCR (qRT‐PCR); and was at least 2 years old. Index cases also had to be the only household member suffering from respiratory disease within 14 days prior to symptom onset. Exclusion criteria were pregnancy, severely reduced health status, and HIV infection. 1‐person households were also not eligible or inclusion.	
Interventions	Quote: "facemask and practising intensified hand hygiene (MH group), wearing facemask only (M group) and none of the 2 (control group)". See Table 4 for details.	
Outcomes	Primary outcomes: SAR of laboratory‐confirmed (qRT‐PCR) influenza infection amongst household members (secondary infection cases) presenting with ILI within the observation period (8 days from the date of onset). ILI was defined as fever (> 38.0 °C) + cough or sore throat. Nasal wash specimens (or if these were not possible, nasal swabs) from all participating household members Effectiveness: Secondary outcomes: laboratory‐confirmed influenza infection in a household contact (secondary infection cases). The study authors defined a symptomatic secondary influenza virus infection as a laboratory‐confirmed influenza infection in a household member who developed fever (> 38.0 °C), cough, or sore throat during the observation period. They termed all other secondary cases as subclinical. A secondary outcome measure was the occurrence of ILI as defined by WHO as fever plus cough or sore throat. Safety: study reported that the majority of participants (107/172, 62%) did not report any problems with mask‐wearing. This proportion was significantly higher in the group of adults (71/100, 71%) compared to the group of children (36/72, 50%) (P = 0.005). The main problem reported by participants (adults as well as children) was "heat/humidity" (18/34, 53% of children; 10/29, 35% of adults) (P = 0.1), followed by "pain" and "shortness of breath" when wearing a face mask.	
Notes	Period study conducted: November 2009 to April 2011 Adherence: in general, daily adherence was good, reaching a plateau of over 50% in nearly all groups (M and MH groups; 2009/10 and 2010/11) from the third day on (by then the intervention had been implemented in all households). A gradual decline towards lower adherence began around the sixth day of the index patient's illness. Government funded. The authors declare that they have no competing interests.	
***Risk of bias***		
**Bias**	**Authors' judgement**	**Support for judgement**
Random sequence generation (selection bias)	Low risk	Quote: "prepared lists of random numbers with Microsoft Excel 2003 (Mircosoft™ Cooperation, Seattle, USA) which were divided between the three intervention groups. Each participating physician received a list of random numbers with the interventions represented in a 1:1:1 ratio"
Allocation concealment (selection bias)	Low risk	Quote: "the participating physician received a list of random numbers with the interventions represented in a 1:1:1 ratio. Eligible index patients were randomly assigned a number, which was then communicated to the study center. The resulting intervention was only communicated to the households with the physicians. Intervention material was given to the study sites in closed boxes marked only with the randomisation number. Recruiting physicians were not aware of the allocation of the numbers to the interventions and the boxes for the three intervention arms looked identical. After randomisation, participants were given their box by the physician's assistants"
Blinding of participants and personnel (performance bias) All outcomes	Unclear risk	Outcomes are very objective and therefore unlikely to be influenced by lack of blinding. In addition, Quote: “physicians (as well as laboratory personnel) blinded from the randomisation results”.
Blinding of outcome assessment (detection bias) All outcomes	Low risk	Quote: physicians (as well as laboratory personnel) blinded from the randomisation results”. Outcomes are very objective and therefore unlikely to be influenced by lack of blinding.
Incomplete outcome data (attrition bias) All outcomes	Low risk	No loss to follow‐up. Daily follow‐up home visits over the short period of data collection (8 days)
Selective reporting (reporting bias)	Low risk	The follow‐up period is very short (8 days) with very good coverage, and the criteria for defining the outcome are highly objective. All planned outcomes were reported.

**Table:** Swarthout 2020

***Study characteristics***		
Methods	Cluster randomised open‐label controlled trial carried out over 18 months in Kenyan geographically near villages to test the effect of a package of measures on pregnant mothers and then on prevalence of ARIs in their young children	
Participants	7246 pregnant women in 702 clusters were enrolled, with 6960 children in year 1 and 7088 in year 2 children with available ARI data. The mean ages of index children and siblings younger than 3 years were 14.2 months (SD: 6.77 months) and 22.9 months (SD: 5.70 months) for years 1 and 2, respectively. The cluster‐level intra‐cluster correlation coefficient for ARIs was 0.026 for both years. There were 2212 households with 2279 children lost to follow‐up by year 2 for unspecified reasons	
Interventions	There were 6 intervention groups: chlorinated drinking water (W), improved sanitation (S), handwashing with soap (H), combined WSH, improved nutrition (N) through counselling lipid based nutrient supplementation (LNS) combined WSHN There were 2 control groups passive control (no promotional visits), a double‐sized active control (monthly visits to measure mid–upper arm circumference) All were done through health promoters with follow up 1 or 2 years after intervention. See Table 4 for details.	
Outcomes	Laboratory NR Effectiveness Prevalence of ARIs in children (defined as cough or difficulty breathing, including panting or wheezing, within 7 days before the interview ‐ in children younger than 3 years). Secondary outcomes included difficulty breathing, including panting or wheezing, in the past 7 days (a more specific indicator of respiratory infection than a cough alone); ARI symptoms presenting with fever in the past 7 days (a potentially more severe infection); and facilitator observed runny nose. As this was a rare outcome, caregiver‐reported runny nose was analysed post hoc Safety NR	
Notes	Quote: “The authors conclude that Water, sanitation, and handwashing interventions with behaviour change messaging did not reduce ARIs. Nutrition counselling and LNS modestly reduced ARI symptoms compared with controls in year 1 [prevalence ratio (PR): 0.87, 95% confidence interval (CI): 0.77–0.99], but no effect in the combined WSHN group weakens this finding” Financial support: this work was supported by the Bill & Melinda Gates Foundation (OPPGD759). The authors declare no further competing interests.	
***Risk of bias***		
**Bias**	**Authors' judgement**	**Support for judgement**
Random sequence generation (selection bias)	Low risk	Computer random‐number generator
Allocation concealment (selection bias)	Unclear risk	Insufficient information provided.
Blinding of participants and personnel (performance bias) All outcomes	High risk	Not blinded
Blinding of outcome assessment (detection bias) All outcomes	High risk	Not blinded
Incomplete outcome data (attrition bias) All outcomes	Low risk	Attrition balanced across groups and < 20%
Selective reporting (reporting bias)	High risk	None of the outcomes reported were prespecified in the trial registry

**Table:** Talaat 2011

***Study characteristics***		
Methods	Cluster‐RCT	
Participants	Children (N = 44,451) in the first 3 primary grades from 60 governmental elementary schools in Cairo, Egypt were included and randomised to 30 schools in the intervention arm (N = 20,882 students) and 30 control schools (N = 23,569 students). No exclusion criteria provided.	
Interventions	Students were required to wash their hands at least twice during the school days for about 45 seconds, followed by proper rinsing and drying on a clean towel. Campaign material was developed, and posters were placed near sinks in the classroom and playground to encourage hand‐washing with soap and water upon arriving at school, before and after meals, using the bathroom, and after coughing and sneezing. See Table 4 for details.	
Outcomes	Laboratory: point‐of‐care influenza A and B viruses using QuickVue (QuickVue; Quidel Corp., San Diego, CA, USA). School nurses collected nasal swabs from children who visited the school clinic with ILI, and only for students who had prior written approval of a parent. Effectiveness: rates of absenteeism caused by ILI and laboratory‐confirmed influenza. ILI defined as fever > 38 °C and either cough or sore throat. Safety: none planned or reported by the investigators	
Notes	The period study conducted: 16 February to 12 May 2008 Funding: this work was supported by the Centers of Diseases Prevention and Control, Work Unit no. 6000.000.000.E0016. No interests declared.	
***Risk of bias***		
**Bias**	**Authors' judgement**	**Support for judgement**
Random sequence generation (selection bias)	Low risk	Quote: "computer‐generated random number table"
Allocation concealment (selection bias)	Unclear risk	No information given.
Blinding of participants and personnel (performance bias) All outcomes	Unclear risk	The participants and study personnel were not blinded, although lack of blinding is unlikely to have influenced the outcome. Laboratory‐confirmed influenza was only conducted only for students who had prior written approval of a parent.
Blinding of outcome assessment (detection bias) All outcomes	Unclear risk	Quote: “Differential interest of study teams may have contributed to the low rate of testing in students who were absent because of ILI in the control schools compared to the intervention schools (12% vs 22%)”
Incomplete outcome data (attrition bias) All outcomes	High risk	No flow chart of clusters flow during the study period. No information on withdrawal. Differential interest of study teams may have contributed to the low rate of testing in students who were absent because of ILI in the control schools compared to the intervention schools (12% vs 22%) incomplete or loss of data. The total number ILI episodes could be an underestimate, as there is no proactive method to look for symptoms of ILI amongst the students; it depends on the student being absent or in class with symptoms that are picked up by the teachers at school.
Selective reporting (reporting bias)	Unclear risk	Insufficient information to judge

**Table:** Teesing 2021

***Study characteristics***		
Methods	Cluster ‐ trial taking place in 66 nursing homes units (33 nursing homes) in the Netherlands during October to December 2016 with 2 follow‐up periods (January to April 2017, May to October 2017). Randomisation was carried out by computer and there were some post‐randomisation imbalances: the intervention arm had more small and medium‐sized nursing homes (< 88 beds, 88 to 118 beds) and the control arm had more large nursing homes (> 118 beds).	
Participants	Nursing home staff whose compliance was measured with direct observation according to the WHO‐defined HH moments and recorded in a novel app. “The nurses were blinded by giving distinct names to the lessons (The New Way of Working) and the observations (HANDSOME), so that they appeared to be different projects. Nurses were told that the observers were registering the frequency of health care activities (in general)”. Staff worked in 66 nursing home units, 36 (976 beds, median 25 per unit) in the intervention arm, and 30 (886 beds, median 28 per unit) in the control arm. During the trial 8 (12%) units left the study during the follow‐up for various reasons: 6 intervention units (four during Follow‐up 1 and 2 during Follow‐up 2) and 2 control units (both during Follow‐up 2)	
Interventions	Hand hygiene (HH) enhancement activities versus no activities. Activities for staff were: an e‐learning session, 3 live lessons, posters, and a photo competition. See Table 4 for details.	
Outcomes	Laboratory NR Effectiveness Incidence of gastroenteritis*, influenza‐like illness (ILI), assumed pneumonia*, urinary tract infections (UTIs)*, and infections caused MRSA* in residents *Data not extracted Safety NR	
Notes	The authors conclude that quote: “This study, similarly to comparable studies, could not conclusively demonstrate the effectiveness of an HH intervention in reducing HAIs among residents of nursing homes, despite the use of clearly defined outcome measures, a standardized illness incident reporting instrument, and directly observed HH in a multicenter cluster‐RCT. This could be due to an insufficient increase in HH compliance and/or other factors in the nursing home environment that need to be addressed concurrently in order to decrease illness rates” The trend of ILI incidence reflects that of the outside community at a higher level. This is probably due to ascertainment bias in the nursing homes in the trial. The trend is seasonal and could be accounted for by visitor transmission. Funding: this study was funded by the Netherlands Organization for Health Research and Development (ZonMw). Non‐financial support was received from Essity during the conduct of the study. Competing interests: the authors declare that they have no competing interests.	
***Risk of bias***		
**Bias**	**Authors' judgement**	**Support for judgement**
Random sequence generation (selection bias)	Low risk	Computer random‐number generator
Allocation concealment (selection bias)	Unclear risk	Insufficient information provided.
Blinding of participants and personnel (performance bias) All outcomes	High risk	Nurses blinded but participants and other staff members not blinded.
Blinding of outcome assessment (detection bias) All outcomes	High risk	Staff members of nursing homes in the intervention arm were potentially extra alert to infections and more motivated to register them.
Incomplete outcome data (attrition bias) All outcomes	Unclear risk	Participant flow diagram not reported.
Selective reporting (reporting bias)	Unclear risk	Insufficient information available

**Table:** Temime 2018

***Study characteristics***		
Methods	2‐arm cluster‐RCT	
Participants	All residents and staff of 27 privately held chains of nursing homes owned by Korian. 26 nursing homes (13 per arm), with an average of 80 residents per nursing home, were included in the study.	
Interventions	Quote: "The intervention was based on a bundle of HH‐related measures aimed at NH staff, residents, visitors, and outside care providers. These measures included facilitated access to handrub solution using pocket‐sized containers and new dispensers, a campaign to promote HH with posters and event organization, the formation of local work groups in each NH to work on HH guidelines, and staff education using e‐learning on infection control and HH training performed by the same nurse for all NHs." See Table 4 for details.	
Outcomes	Laboratory: none used Effectiveness: Primary outcomes: incidence rate of ARIs and AGE reported in the context of episodes of clustered cases, deﬁned as at least 5 cases within 4 days amongst nursing home residents or staff. ARIs were deﬁned as the combination of at least 1 respiratory symptom with 1 symptom of systemic infection. AGE was deﬁned as the sudden onset of diarrhoea or vomiting in the absence of a non‐infectious aetiology. Secondary endpoints were mortality rate, hospitalisation rate, and antibiotic prescription rate (measured in deﬁned daily doses (DDDs) per 100 resident days). Safety: no adverse event surveillance planned or reported by the investigators	
Notes	The period study conducted: 1 April 2014 to 1 April 2015 Funding: private (Institute of Ageing Well Korian (Institut du bien vieillir Korian), which runs the nursing homes included in the study) Conflicts of interest: none to report.	
***Risk of bias***		
**Bias**	**Authors' judgement**	**Support for judgement**
Random sequence generation (selection bias)	High risk	"simple” randomisation is used
Allocation concealment (selection bias)	Unclear risk	No information provided.
Blinding of participants and personnel (performance bias) All outcomes	High risk	Quote: “we suspected that underreporting occurred. The data were verified qualitatively after the end of the intervention through individual phone interviews with each participating NH. Based on these interviews, ARI clustered cases episodes had actually occurred in 12 out of 13 control NHs; however, only 1 had been notified to health authorities. No unreported clustered cases episodes were identified in the intervention NHs”
Blinding of outcome assessment (detection bias) All outcomes	High risk	Data were collected at NH level and reported to centralised by the NH group headquarters in Paris through computerised databases. There was underreporting of ARI and AGE in the control groups. The trial authors suspected that underreporting occurred. Primary outcome: high risk. Secondary outcomes: low risk
Incomplete outcome data (attrition bias) All outcomes	High risk	For the primary outcome, there was underreporting of ARI and AGE in the control groups; no study flow chart was provided; and no reporting on any exclusions. Surveillance is based on voluntary and standardised notifications to health authorities of any AGE or ARI clustered case episode.
Selective reporting (reporting bias)	Low risk	Reported outcomes match planned outcomes published in the protocol.

**Table:** Turner 2004a

***Study characteristics***		
Methods	Double‐blind RCT conducted by Hill Top Research, Inc., Winnipeg, Canada, to assess the efficacy of acids with virucidal activity for the inactivation of virus and prevention of experimental rhinovirus colds. Participants in good health, aged 18 to 60, were recruited from Winnipeg and surrounding communities for participation. Qualified participants were randomised to treatment with vehicle (62% ethanol, 1% ammonium lauryl sulphate, and 1% Klucel), vehicle containing 3.5% salicylic acid, or vehicle containing 1% salicylic acid and 3.5% pyroglutamic acid. The volunteers' hands were disinfected, and then test product was applied to both hands of participant. 15 minutes after application, the fingerprints of each hand were contaminated with rhinovirus type 39. The volunteers touched conjunctiva and the nasal mucosa only with the right hand. Viral contamination of the fingers was assessed in the left hands of the volunteers, and viral infection was assessed by culture of nasal lavage specimens and blood samples.	
Participants	85 volunteers; 31 control group, 27 used vehicle with 3.5% salicylic acid, 27 used vehicle with 1% salicylic acid and 3.5% pyroglutamic acid	
Interventions	Use of salicylic acid versus salicylic acid and pyroglutamic acid versus "placebo" substance. See Table 4 for details.	
Outcomes	Laboratory: yes Effectiveness: rhinovirus type 39 infection Safety: N/A	
Notes	Risk of bias: unclear (no description of randomisation process, concealment or allocation) Note: the authors concluded that organic acids commonly used in over‐the‐counter skin care and cosmetic products have substantial virucidal activity against rhinovirus. These preparations provided effective residual antiviral activity on the hands. The virucidal effect of these hand treatments resulted in a reduction in the incidence of rhinovirus infection in the treated volunteers (P = 0.025). The utility of this observation in the natural setting remains to be determined. The volunteers were not allowed to use their hands in the interval between the hand treatment and the virus challenge, so the effect of normal use of the hands on the virucidal activity of these organic acids is not known. Similarly, the virus challenge method used in these experiments may not simulate the natural setting in all aspects. The effect of nasal secretions that would be transferred with the virus in the natural setting on the activity of the acids or on the transmission of virus was not tested in the model. We are unsure as to the practical significance of this study and the generalisability of its results to the real world. Poorly reported study Funding for this study was provided by the Procter & Gamble Co., Cincinnati, Ohio. No interests declared.	
***Risk of bias***		
**Bias**	**Authors' judgement**	**Support for judgement**
Random sequence generation (selection bias)	Unclear risk	Quote: "randomised" Sequence generation not described.
Allocation concealment (selection bias)	Unclear risk	Not reported
Blinding of participants and personnel (performance bias) All outcomes	Unclear risk	Quote: "double blind", but no description
Blinding of outcome assessment (detection bias) All outcomes	Unclear risk	Quote: "double blind", but no description
Incomplete outcome data (attrition bias) All outcomes	Low risk	All accounted for (short study).
Selective reporting (reporting bias)	High risk	Poorly reported

**Table:** Turner 2004b

***Study characteristics***		
Methods	Double‐blind RCT conducted by Hill Top Research, Inc., Winnipeg, Canada, to assess the residual virucidal activity of a skin cleanser wipe and its effectiveness in preventing experimental rhinovirus colds. Participants in good health, aged 18 to 60 years, were recruited from Winnipeg and surrounding communities for participation. The residual activity of a skin cleanser wipe containing 4% pyroglutamic acid formulated with 0.1% benzalkonium chloride was tested. The negative control treatment was 62% ethanol. Benzalkonium chloride had been previously tested and was found to have no virucidal activity. Volunteers were randomly assigned to use the control preparation or the active preparation. The study material was applied to hands with a towelette. 15 minutes later, when the fingers were completely dry, the fingertips of each hand of the control participants and the volunteers in the active treatment group were contaminated with rhinovirus type 39. An additional volunteer in the active group was challenged with virus 1 hour after application, and the final group of volunteers was challenged 3 hours after application. Viral infection was assessed by culture of nasal lavage specimens and blood samples.	
Participants	122 volunteers; 30 in control group, 92 in active group (30 tested after 15 minutes, 30 after 1 hour, 32 after 2 hours)	
Interventions	Use of a skin cleanser wipe containing 4% pyroglutamic acid formulated with 0.1% benzalkonium chloride versus skin cleanser wipe containing ethanol. See Table 4 for details.	
Outcomes	Laboratory: yes Effectiveness: rhinovirus type 39 infection Safety: N/A	
Notes	Risk of bias: unclear (no description of randomisation process, concealment or allocation) Funding for this study was provided by the Procter & Gamble Co., Cincinnati, Ohio. No interests declared.	
***Risk of bias***		
**Bias**	**Authors' judgement**	**Support for judgement**
Random sequence generation (selection bias)	Unclear risk	Quote: "randomised" Sequence generation not described.
Allocation concealment (selection bias)	Unclear risk	Not reported
Blinding of participants and personnel (performance bias) All outcomes	Unclear risk	Quote: "double blind", but no description given
Blinding of outcome assessment (detection bias) All outcomes	Unclear risk	Quote: "double blind", but no description given
Incomplete outcome data (attrition bias) All outcomes	Low risk	All accounted for (short study).
Selective reporting (reporting bias)	High risk	Poorly reported

**Table:** Turner 2012

***Study characteristics***		
Methods	Randomised controlled clinical trial	
Participants	A total of 212 participants were enrolled (116 in the treatment group, 96 in the control group). Healthy adult volunteers aged > 18 years from the University of Virginia community Written informed consent was obtained, and volunteers were compensated for participation. Exclusion: individuals with skin conditions that would interfere with safety evaluations or medical conditions that could impact the person's well‐being or affect study results, and those whose occupations required frequent hand‐washing	
Interventions	Antiviral hand treatment containing 2% citric acid, 2% malic acid, and 62% ethanol (n = 116) or to a no‐treatment control group (n = 96). The hand treatment was applied every 3 hours and after hand‐washing whilst the participants were awake. See Table 4 for details.	
Outcomes	Laboratory: PCR using AmpliTaq Gold DNA Polymerase from Applied Biosystems Effectiveness: reduction of rhinovirus‐induced common colds; comparison of the number of RV‐associated illnesses per 100 participants in the control group with that in the treatment group over 9 weeks. Definitions: a common cold illness was defined as the presence of any of the symptoms of nasal obstruction, rhinorrhoea, sore throat, or cough on at least 3 consecutive days. Illnesses separated by at least 3 symptom‐free days were considered to be separate illnesses. Rhinovirus infection was defined as the detection of RV in nasal lavage. All volunteers were seen weekly for nasal lavage, and specimens were assayed by PCR for the presence of RV. PCR–positive specimens separated by at least 8 days and at least 1 negative PCR specimen were considered to be separate infections. RV‐associated illnesses were based on detection of RV either at the time of the illness or at the first weekly visit after the illness. Safety: hand irritation occurred in 11 of the 116 volunteers (9%) in the treatment group, which met protocol criteria for removal from the study. An additional 8 participants who did not meet these protocol criteria voluntarily withdrew due to hand irritation. There was no hand irritation in the control group. No other adverse effects of the study treatment were noted.	
Notes	The period study conducted: August 2009 to November 2009 Funding: The Dial Corporation ‐ a Henkel Company, Scottsdale, Arizona, USA Potential conflicts of interest: R. B. T. is a consultant to Henkel and received grant funding to conduct these studies. All other authors are current or former employees of Henkel. All authors have submitted the ICMJE Form for Disclosure of Potential Conflicts of Interest.	
***Risk of bias***		
**Bias**	**Authors' judgement**	**Support for judgement**
Random sequence generation (selection bias)	Low risk	Quote: "A randomization code generated using commercially available software was provided by the sponsor"
Allocation concealment (selection bias)	Low risk	Quote: "staff at the study site assigned sequential subject numbers as they enrolled volunteers into the study, and treatment assignment was determined by the subject number."
Blinding of participants and personnel (performance bias) All outcomes	Low risk	The outcomes are unlikely to be influenced by lack of blinding.
Blinding of outcome assessment (detection bias) All outcomes	Low risk	Quote: "Personnel who conducted the laboratory assays were blinded to study groups and to whether the specimen was from a routine or illness related visit"
Incomplete outcome data (attrition bias) All outcomes	Low risk	Attrition (and reasons for it) was reported. Study outcomes reported as ITT and PP.
Selective reporting (reporting bias)	Low risk	All planned outcomes in study protocol were reported on.

**Table:** White 2001

***Study characteristics***		
Methods	Double‐blind, placebo‐controlled, cluster‐RCT that took place in 3 schools in California during March to April 1999. The study assessed the incremental value of using an alcohol hand rub together with water‐and‐soap hand‐washing. Both arms were administered an educational programme beginning 2 weeks prior to start of the trial. Randomisation was by classroom, and the placebo hand rub was indistinguishable from the active ingredient. Details of randomisation are not given.	
Participants	Of the 72 classes originally recruited, lack of compliance (use of supplementary product at least 3 times a day) reduced the classes to 32 (16 in both arms) and a total of 769 participants aged 5 to 12 (381 students who received the sanitiser, and 388 who received the placebo).	
Interventions	Pump‐activated antiseptic hand rub with benzalkonium chloride (SAB) (Woodward Laboratories) or inert placebo that "virtually" looked the same in batches of 4 colour‐coded bottles. School staff, parents, and participants were blinded. See Table 4 for details.	
Outcomes	Laboratory: testing of virucidal and bactericidal activity of the active compound Effectiveness: ARI (cough, sneezing, sinus trouble, bronchitis, fever, red eye, headache, mononucleosis, acute exacerbations of asthma) Gastrointestinal and other illnesses (data not extracted) Follow‐up and observation was carried out by classroom staff, and illnesses were described by parents. Safety: 7 students dropped out because of mild sensitivity to the rub	
Notes	Risk of bias: high (no description of randomisation; partial reporting of outcomes, numerators and denominators) Note: the authors conclude that addition of the rub led to a 30% to 38% decrease of illness and absenteeism (RR for illness absence incidence 0.69, RR for absence duration 0.71). Very high attrition, unclear randomisation procedure, educational programme and use of placebo hand rub make generalisability of the results debatable. No confidence intervals reported. This study was supported by an Orange County School Nurses Organization Health Promotion Grant. No interests declared.	
***Risk of bias***		
**Bias**	**Authors' judgement**	**Support for judgement**
Random sequence generation (selection bias)	Unclear risk	Quote: "randomised trial", but sequence generation not described
Allocation concealment (selection bias)	Unclear risk	Not described
Blinding of participants and personnel (performance bias) All outcomes	Low risk	Quote: "To distinguish content, both the active and placebo formulations were distributed in four color‐coded groups of 1oz spritz bottles. The content were and distribution patters were only know to the researchers and were indecipherable by the school staff or students."
Blinding of outcome assessment (detection bias) All outcomes	Low risk	Quote: "Teachers were responsible for recording attendance for each day during the study"
Incomplete outcome data (attrition bias) All outcomes	High risk	Partial reporting of outcomes, numerators and denominators
Selective reporting (reporting bias)	High risk	Poor reporting

**Table:** Yeung 2011

***Study characteristics***		
Methods	Clustered‐RCT of a hand hygiene intervention involving pocket‐sized containers of alcohol‐based hand rub for the control of infections in long‐term care facilities. Staff hand hygiene adherence was directly observed, and residents' infections necessitating hospitalisation were recorded. After a 3‐month pre‐intervention period, long‐term care facilities (LTCFs) were randomised to receive pocket‐sized containers of alcohol‐based gel, reminder materials, and education for all HCWs (treatment group) or to receive basic life support education and workshops for all HCWs (control group). A 2‐week intervention period (1 to 15 April 2007) was followed by 7 months of postintervention observations.	
Participants	6 out of 7 community‐based, private or semiprivate, residential LTCFs in Hong Kong agreed to participate and were randomised to: hand hygiene group (3 LTCFs, 73 nursing staff and 244 residents analysed); or control group (3 LTCFs, 115 nursing staff and 379 residents analysed). All were nursing homes serving an elderly population. All LTCFs were situated in different regions of Hong Kong, including urban and rural areas. The targets of the intervention were all full‐ and part‐time HCWs at these LTCFs. The LTCFs employed 3 types of HCWs: nurses, nursing assistants, and physiotherapists.	
Interventions	Pocket‐sized containers of alcohol‐based gel, reminder materials, and education (intervention group) or basic life‐support education and workshop (control group). See Table 4 for details.	
Outcomes	Rates of infection (requiring hospitalisation) Outbreaks Death due to infection Diagnoses of infection coded into 6 categories, all of which were common endemic infections in LTCFs: pneumonia, urinary tract infection, septicaemia, skin or soft‐tissue infection (including cellulitis or pressure sores), gastroenteritis, and fever. Infections recorded in death certificates were also included, regardless of whether the resident had been hospitalised. The causes of death were categorised as due to infection, not due to infection, or unknown. If the primary or the secondary diagnosis on the death certificate belonged to 1 of the 6 endemic infection categories, the death was coded as due to infection. No safety outcomes reported.	
Notes	University and industry funded.	
***Risk of bias***		
**Bias**	**Authors' judgement**	**Support for judgement**
Random sequence generation (selection bias)	Unclear risk	No details provided.
Allocation concealment (selection bias)	Unclear risk	No details provided.
Blinding of participants and personnel (performance bias) All outcomes	High risk	Unblinded study
Blinding of outcome assessment (detection bias) All outcomes	High risk	Unblinded study
Incomplete outcome data (attrition bias) All outcomes	Low risk	No loss to follow‐up
Selective reporting (reporting bias)	Unclear risk	No protocol available

**Table:** Young 2021

***Study characteristics***		
Methods	Cluster‐randomised, controlled trial of daily contact testing in students and staff at secondary schools and colleges in England to show whether daily contact testing increases school attendance and to assess the impact of daily contact testing on SARS‐CoV‐2 transmission within schools.	
Participants	201 schools, of which 99 were randomly assigned to self‐isolation of school‐based COVID‐19 contacts for 10 days (control) and 102 to voluntary daily lateral flow device (LFD) testing for 7 days with LFD‐negative contacts remaining at school (intervention)	
Interventions	All schools in the intervention and control groups followed the national policy of offering twice weekly asymptomatic testing with LFDs. Individuals with positive LFD results were required to self‐isolate immediately and requested to obtain a confirmatory PCR test within 2 days. Those with indicator symptoms of possible COVID‐19 (new cough, fever, loss or change in taste or smell) were required to self‐isolate along with their household and obtain an urgent PCR test. If a student or staff member tested positive by LFD or PCR, close contacts (hereafter referred to as contacts) were identified by schools using national guidelines. Those in close contact with a case less than 48 hours before symptom onset (or a positive test if asymptomatic) were required to self‐isolate for 10 days. At schools in the intervention group, contacts were offered daily contact testing as an alternative to self‐isolation, provided the contact was school‐based (i.e. with a staff member or student), the contact did not have indicator symptoms of COVID‐19, and contacts were able to attend for on‐site testing at school. See Table 4 for details.	
Outcomes	Laboratory PCR confirmed infections Effectiveness COVID‐19‐related school absence and symptomatic PCR‐confirmed COVID‐19. Safety NR	
Notes	The authors conclude that quote: “Daily contact testing of school‐based contacts was non‐inferior to self‐isolation for control of COVID‐19 transmission, with similar rates of symptomatic infections among students and staff with both approaches." Funding: UK Government Department of Health and Social Care. Declaration of interests: DWE reports lecture fees from Gilead outside the submitted work. VB, RO, and DC are consultants employed by Department of Health and Social Care as part of Deloitte’s broader project work supporting the delivery of NHS Test and Trace. TF reports honoraria from Qatar National Research Fund outside the submitted work. All other authors declare no competing interests. Potential conflicts of interest: all authors report no conflicts of interest relevant to this article.	
***Risk of bias***		
**Bias**	**Authors' judgement**	**Support for judgement**
Random sequence generation (selection bias)	Low risk	Computer random‐number generator
Allocation concealment (selection bias)	Unclear risk	Insufficient information reported
Blinding of participants and personnel (performance bias) All outcomes	High risk	Not blinded.
Blinding of outcome assessment (detection bias) All outcomes	High risk	Not blinded.
Incomplete outcome data (attrition bias) All outcomes	High risk	Participant flow diagram reported showing high attrition at different rates in the 2 groups
Selective reporting (reporting bias)	Low risk	Prespecified outcomes reported

**Table:** Zomer 2015

***Study characteristics***		
Methods	Cluster‐RCT	
Participants	71 daycare centres (36 intervention DCCs, and 35 control) in Rotterdam‐Rijnmond, Gouda and Leiden in the Netherlands Study enrolled 545 children (intervention = 278, control = 267). Inclusion/exclusion criteria: children who attended the DCC at least 2 days a week; were aged between 6 months and 3.5 years at start of the trial; intended to attend the DCC throughout the study period; and if their parents consented, were Dutch‐speaking, and had access to email or regular post. Children were excluded if they had a chronic illness or medication that predisposed them to infection, a sibling taking part in the trial (i.e. 1 child per family could be included), or if they started attending CCC after the beginning of the trial).	
Interventions	4 components: HH products, paper towel dispensers, soap, alcohol‐based hand sanitiser, and hand cream were provided for 6 months. Training and a booklet outlining the training. 2 team training sessions aimed at specific HH improvement activities. Posters and stickers for caregivers and children as reminders. See Table 4 for details.	
Outcomes	Laboratory: none Effectiveness: incidence of respiratory infections in children monitored by parents. The common cold was defined as a blocked or runny nose with at least 1 of the following symptoms: coughing, sneezing, fever, sore throat, or earache. Safety: none planned or reported by the investigators	
Notes	The period study conducted: September 2011 to April 2012 Funding: mixed. The Netherlands Organisation for Health Research and Development (ZonMw). Dispensers and refills were sponsored by SCA Hygiene Products, Sweden. Declaration of interest: none.	
***Risk of bias***		
**Bias**	**Authors' judgement**	**Support for judgement**
Random sequence generation (selection bias)	Low risk	Quote: "Stratified randomization is performed by assigning each DCC to one of six strata based on size (i.e. small < 46 children per day versus large ≥ 46 children per day) and geographic location (i.e. highly urban versus urban versus slightly/non‐urban). DCCs are assigned to either intervention or control group by means of computer generation with a 1:1 ratio in each of the strata"
Allocation concealment (selection bias)	Unclear risk	No information provided.
Blinding of participants and personnel (performance bias) All outcomes	High risk	Outcome is subjective.
Blinding of outcome assessment (detection bias) All outcomes	High risk	Symptoms were reported by parents, no validation.
Incomplete outcome data (attrition bias) All outcomes	Low risk	Very few children were excluded or lost to follow‐up (reasons for exclusions provided).
Selective reporting (reporting bias)	Low risk	All planned outcomes are reported. However, between published protocol and the paper, secondary outcomes became the primary outcome in the published paper!

AEs: adverse events AFH: Armed Forces Hospital AGE: acute gastroenteritis AgNPs: ARGOVIT silver nanoparticles ALRI: acute lower respiratory infection ARI: acute respiratory infection ASR: adverse skin reactions A&E: accident and emergency BIPAP: bilevel positive airway pressure CCC: childcare centre CDC: Centers for Disease Control and Prevention CG: control group CHG: chlorhexidine gluconate CI: confidence interval CMF: citric acid: malic acid: sodium lauryl sulphate (a virucidal mixture added to tissue paper) CoV: coronavirus cluster‐RCT: cluster‐randomised controlled trial CRI: clinical respiratory illness CXR: chest X‐ray DCC: daycare centre EG: experimental group FRI: febrile respiratory illness FU: follow up GI: gastrointestinal GTI: gastrointestinal infection GP: general practitioner HCW: healthcare worker HFH: Hanoi French Hospital HH: hand hygiene HR: high risk HSG: hand sanitiser group ICD‐9: International Classification of Disease, 9th Revision, Clinical Modification IgG: immunoglobulin G ICU: intensive care unit ILI: influenza‐like illness IQR: interquartile range IRR: incident rate ratio ITT: intention‐to‐treat KSA: Kingdom of Saudi Arabia LFD: lateral flow device LNS: lipid based nutrient supplementation LRTI: lower respiratory tract infection LTCF: long‐term care facility m: metre MCU: medical convalescent unit MDCK: Madin Darby canine kidney cell line M group: face mask group MH group: face mask and hand hygiene group MS: monkey‐derived cell line N/A: not applicable NAT: nucleic acid testing NH: nursing home NICU: neonatal intensive care unit NOS: Newcastle‐Ottawa Scales NP: non‐pharmaceutical NR: not reported NTS: nasal and throat swab OR: odds ratio PCR: polymerase chain reaction PCU: physical conditioning unit POCT: point‐of‐care testing PP: per protocol PPE: personal protective equipment QNAF: Qatar National Research Fund RCT: randomised controlled trial RDS: respiratory distress syndrome RI: respiratory infection RIDT: rapid influenza diagnostic test RNA: ribonucleic acid RR: risk ratio rRT‐PCR: real‐time reverse transcription‐polymerase chain reaction RTI: respiratory tract infection RT‐PCR: reverse‐transcriptase polymerase chain reaction RSV: respiratory syncytial virus RV: rhinovirus SAB: surfactant, allantoin, and benzalkonium chloride SAR: secondary attack rate SARS: severe acute respiratory syndrome SCBU: special care baby unit SD: standard deviation SES: electrolysed water SHEWA‐B: Sanitation, Hygiene Education and Water Supply in Bangladesh SOB: shortness of breath SOPs: standard operating procedures S/S: signs/symptoms SSTI: skin and soft‐tissue infection STH: soil‐transmitted helminth SWG: soap and water group TIDieR: Template for Intervention Description and Replication UHR‐I: ultra high‐risk infection UHR‐S: ultra high‐risk SARS URI: upper respiratory infection URTI: upper respiratory tract infection WBC: white blood cell WHO: World Health Organization WSH: water, sanitation, and handwashing (combined)

### Characteristics of excluded studies [ordered by study ID]

**Table:**

Study	Reason for exclusion
Abou El Hassan 2004	Topic completely extraneous
Ahmadian 2022	Excluded as study is an experiment that did not measure any of our outcomes of interest.
Amirav 2005	Randomised controlled trial of aerosol treatment
Anderson 2004	Mathematical model with interesting discussion of interaction between public health measures
Anonymous 2002	News item
Anonymous 2004	News item
Anonymous 2005a	News item
Anonymous 2005b	News item
Anonymous 2005c	News item
Apisarnthanarak 2009	Intervention bundle not broken down.
Apisarnthanarak 2010	Participants took antivirals.
Aragon 2005	Descriptive paper (non‐comparative). Has no viral outcomes
Azor‐Martinez 2014	Results reported as respiratory and gastrointestinal infections. No extractable respiratory data
Barros 1999	Correlational study between incidence of URTI and factors such as overcrowding
Bauer 2009	Historical comparison with RSV gammaglobulin amongst interventions
Bell 2004	Has unpublished entry exit screening data and extensive references but no comparative data
Bellissimo‐Rodrigues 2009	Intervention is chlorhexidine.
Ben‐Abraham 2002	Exclude ‐ bacterial illness only
Black 1981	Diarrhoea only outcome
Borkow 2010	No human beings involved.
Bouadma 2010	Hospital‐based ventilator routine
Bowen 2007	Outcomes of composite infections. Respiratory infections are not reported separately.
Breugelmans 2004	Description of risk factors in aircraft
Cai 2009	Compliance study
Cantagalli 2010	Outcome outside inclusion criteria
Carbonell‐Estrany 2008	Immunoglobulin intervention and descriptive review
Carter 2002	News item
Castillo‐Chavez 2003	Editorial
Cava 2005a	Survey of quarantinees' views
Cava 2005b	Personal experiences of quarantine
CDC 2003a	Case reports
CDC 2003b	No data presented.
Chai 2005	Letter ‐ about MRSA
Chami 2012	Outcomes of composite infections. Respiratory infections are not reported separately.
Chaovavanich 2004	Case report
Chau 2003	No original retrievable data. Mathematical model fitting expected to observed cases with quarantine in the SARS of Hong Kong
Chau 2008	Audit of infection control procedures and compliance with guidelines
Chen 2007	An assessment of the impact of different hand‐washing teaching methods. No clinical outcomes
Chen 2022	Not a RCT.
Cheng 2010	Confounded by antiviral use for postexposure prophylaxis
Chia 2005	Knowledge survey
Clynes 2010	Letters
Costa 2021	No clinical outcome assessed
Cowling 2007	Epidemiology, non‐comparative, non‐interventions study
Cyril Vitug 2021	Is a treatment for COVID‐19 infection
Dalakoti 2022	Excluded as study is an experiment that did not measure any of our outcomes of interest.
Daniels 2010	Commentary
Daugherty 2008	No free data presented.
Davies 1994	Antibody titres as outcomes with so many biases that interpretation of study is problematic
Day 1993	No acute respiratory infection outcome data
Day 2006	Mathematical model; no new data
Dell'Omodarme 2005	Probabilistic and Bayesian mathematical model of screening at entry
Denbak 2018	Outcomes of composite infections. Respiratory infections are not reported separately.
Desenclos 2004	Description of transmission
DiGiovanni 2004	Qualitative study of compliance factors in quarantine
Doebbeling 1992	RCT respiratory data not present. Only 3 viruses isolated in total with no viral typing available.
Dwosh 2003	Case series
Edmonds 2010	Lab study
Egger 2022	Excluded as study is an experiment that did not measure any of our outcomes of interest.
Fendler 2002	Cohort study badly biased with differential health profiles and healthcare workers dependency in intervention and control semi‐cohorts. No attempt to adjust for confounders was made. No denominators available.
Ferrer 2021	Is a treatment (not something to interrupt transmission)
Flint 2003	Description of spread in aircraft and non‐comparative data
Fung 2004	Non‐comparative
Garcia 2010	Commentary
Gaydos 2001	Editorial linked to Ryan 2001. (Ryan 2001 was an included trial in a previous version of this review (2011). Non‐RCTs were removed in this 2020 update).
Gensini 2004	Interesting historical review
Gharebaghi 2020	Study on the prevention of ventilator associated pneumonia in mechanical ventilatory patients
Girou 2002	Non‐clinical outcomes
Giuliano 2021	Outcome is hospital aquired pneumonia which is a syndrome with multiple aetiologies, mainly bacterial and mycotic
Glass 2006	Mathematical model ‐ no original data presented
Goel 2007	Non‐comparative study
Gomersall 2006	Non‐comparative study
Gore 2001	Summary of Dyer 2000. (Dyer 2000 was a prospective, cluster open‐label cross‐over cohort study included in the previous version of this review (2011). Non‐RCTs were removed in this 2020 update).
Gostin 2003	Not an analytical study
Gralton 2010	Review
Guinan 2002	It would appear that 9 classes took part and "acted as their own controls", but it is not clear if there was cross‐over of classes or not. In addition, the outcome is combined gastrointestinal/respiratory. The clue lies in the presence of a nested economic analysis which shows considerable savings in time for staff and pupils if the soap is used: in other words this is a (covert) publicity study.
Gupta 2005	Economic model ‐ no new data
Gwaltney 1982	No breakdown of cases given by arm.
Han 2003	Non‐comparative
Hayden 1985	This is an RCT with laboratory‐induced colds, small numbers, and uncertain numerators, but almost certainly because of the unique laboratory conditions (placebo tissues not being a placebo at all) of impossible generalisation. It was a pilot to the far bigger trial by Farr 1988a; Farr 1988b.
Hendley 1988	Inappropriate intervention
Hens 2009	Model
Heymann 2009	Already included in review as Heymann 2004. (Heymann 2004 was a controlled before and after study included in the previous version of this review (2011). Non‐RCTs were removed in this 2020 update).
Hilburn 2003	No ARI/viral outcomes (e.g. URTIs)
Hilmarsson 2007	Animal study
Hirsch 2006	Study tested pharmacological interventions.
Ho 2003	Descriptive review
Hsieh 2007	Mathematical model
Hugonnet 2007	Letter without any data
Jiang 2003	Two papers that are probably different versions of the same paper: Jiang SP, Huang LW, Wang JF, Wu W, Yin SM, Chen WX, et al. A study of the architectural factors and the infection rates of healthcare workers in isolation units for severe acute respiratory syndrome. Chung‐Hua Chieh Ho Ho Hu Hsi Tsa Chih [Chinese Journal of Tuberculosis & Respiratory Diseases]. 26(10):594‐7, 2003 Oct
Johnson 2009	Outcomes are non‐clinical.
Jones 2005	Historical account
Karakaya 2021	Outcome is ventilator associated pneumonia which is a syndrome with multiple aetiologies, mainly bacterial and mycotic
Kawyannejad 2020	Trial on mouthwash for VAP patients with no viral infection outcomes
Kaydos‐Daniels 2004	Not an analytical study
Kelso 2009	Model
Khaw 2008	Assessing the efficacy of O_2_ delivery
Kilabuko 2007	Aetiological study
Kosugi 2004	Non‐comparative study
Lam 2004	Outcomes were generic (infection rates). No laboratory data available for viral diagnosis.
Lange 2004	No data presented.
Larson 2004a	Inappropriate outcomes
Larson 2004b	Inappropriate outcomes
Larson 2005	Cluster‐RCT comparing the effects of 2 hand hygiene regimens on infection rates and skin condition and microbial counts of nurses' hands in neonatal intensive care units. Outcomes were generic (e.g. pneumonia and microbial counts of participants' skin). No laboratory data available for viral diagnosis.
Lau 2004	Attitude survey
Lau 2005	Herbal remedy effectiveness assessment
Lee 2005	Descriptive study of risk and protective factors of transmission in households. No assignment took place.
Lee 2010	Cohort study; unclear numbers were vaccinated against influenza
Lennell 2008	Measured absenteeism due to non‐specific infection
Lim 2022	Not a RCT.
Lipsitch 2003	Mathematical model fit to evidence
Luckingham 1984	Historical report on Tucson experience during Spanish flu pandemic
Ma 2004	Case‐control study of risk factors for SARS
MacIntyre 2010	Commentary on Cowling 2009
Malaczek 2022	Excluded as study is an experiment that did not measure any of our outcomes of interest.
Malone 2009	Model
Marin 1991	Viral resistance study
McSweeny 2007	Historical description
Meister 2022	Excluded as this is a treatment trial (all participants had COVID).
Mielke 2009	Review
Mikolajczyk 2008	No intervention
Mo 2022	Not a RCT.
Monsma 1992	Non‐comparative study
Montero‐Vilchez 2022	Excluded as study is an experiment that did not measure any of our outcomes of interest.
Munoz‐Basagoiti 2022	Excluded as this is a report of another study.
Nandrup‐Bus 2009	The trial had only 2 clusters.
Nishiura 2009	Model
O'Callaghan 1993	Letter linked to Isaacs 1991. (Isaacs 1991 was a retrospective and prospective cohort study included in a previous version of this review (2011). Non‐RCTs were removed in the 2020 update).
Olsen 2003	Description of transmission
Ooi 2005	Descriptive study, but with interesting organisational chart
Orellano 2010	Confounded by antiviral use
Panchabhai 2009	Pharma intervention
Pang 2004	Descriptive study of Beijing outbreak. Some duplicate data in common with Pang 2003. (Pang 2003 was an eclogical study included in a previous version of this review (2011). Non‐RCTs were removed in the 2020 update).
Patel 2012	Although within each district the participating schools and households were randomly selected, the allocation of districts to the intervention and comparison arms was not randomly assigned.
Pittet 2000	Analysis of relationship between hand‐washing compliance campaign and nosocomial bacterial infections (e.g. MRSA)
Prasad 2004	Letter about retrospective cohort ‐ behavioural
Rabenau 2005	In vitro test of several disinfectants
Reynolds 2008	Describes the psychological effects of quarantine
Richardson 2010	Non‐clinical study
Riley 2003	Mathematical model fit to evidence
Rodriguez 2009	A “reasonable attempt at minimizing bias” (see inclusion criteria) does not include absenteeism
Rosen 2006	Non‐specific outcome. Measured absenteeism
Rosenthal 2005	Outcomes were generic (e.g. pneumonia, URTIs). No laboratory data available for viral diagnosis.
Safiulin 1972	Non‐comparative set of studies with no clinical outcomes
Sanchez Barrueco 2022	Excluded as this is a treatment trial (all participants had COVID)
Sandrock 2008	Review
Sattar 2000	Experiment assessing virucidal activity of fingertip surface ‐ no clinical outcome data
Schull 2007	Describes the impact of SARS in a Toronto study
Seal 2010	Lab study
Seale 2009	Study looking at whether using respirators in A&E department is feasible
Seneviratne 2021	Not an intervention to reduce transmission and they did not look at ARIs or other clinically relevant outcomes
Sevinc Gul 2022	Excluded as this is a treatment trial (all participants had COVID)
Sizun 1996	This is a review; no original data presented.
Slayton 2016	Compares hand‐washing plus (antibacterial) towel versus hand‐washing without towel
Stebbins 2009	Attitude survey
Stedman‐Smith 2015	Composite outcome. No data on separate respiratory illnesses reported.
Stoner 2007	No study data available.
Stukel 2008	Impact of the SARS disruption on care/mortality for other pathologies (e.g. acute myocardial infarction). There are no interventions, and outcomes are unrelated to acute respiratory infections.
Svoboda 2004	Descriptive study with before‐and‐after data but shifting denominators
Tracht 2010	Model
Ueno 1990	Experimental study. No clinical intervention
Uhari 1999	No respiratory illness data to be extracted
van der Sande 2008	Laboratory study without any clinical outcomes
Vessey 2007	Composite outcome. No data on separate respiratory illnesses reported.
Viscusi 2009a	Lab study
Viscusi 2009b	Lab study
Wang 2003	Descriptive study
Wang 2005	Case‐control study of susceptibility factors
Weber 2004	Editorial linked to Larson 2004a
Wen 2010	Lab study
White 2005	Redundant publication of White 2003. (White 2003 was a prospective, open, cohort study included in a previous version of this review (2011). Non‐RCTs were removed in the 2020 update).
Wilczynski 1997	Clinical trial of the effects of breastfeeding
Wilder‐Smith 2003	Description of risk factors in aircraft
Wilder‐Smith 2005	Descriptive review
Wong 2005	Attitude survey
Yen 2010	Model
Yu 2004	Description of transmission
Zamora 2006	Head‐to‐head comparison of 2 sets of PPEs with no controls and no clinical outcomes
Zhai 2007	Non‐comparative study
Zhao 2003	CCT of SARS treatment

A&E: accident and emergency ARI: acute respiratory infection CCT: controlled clinical trial MRSA: methicillin‐resistant *Staphylococcus aureus* RCT: randomised controlled trial RSV: respiratory syncytial virus PPE: personal protective equipment SARS: severe acute respiratory syndrome URTI: upper respiratory tract infection VAP: ventilator associated pneumonia

### Characteristics of studies awaiting classification [ordered by study ID]

**Table:** Contreras 2022

Methods	Follow‐up of the WASH Benefits Bangladesh cluster‐randomised controlled trial. Access to and reported use of latrines was high in both arms, and latrine quality was significantly improved by the intervention, while use of child faeces management tools was low. A random subset of households from the sanitation and control arms was enrolled into a longitudinal substudy, which measured child health with quarterly visits between 1 to 3.5 years after implementation.
Participants	9800 observations on children < 5 years through intention‐to‐treat analysis using generalised linear models with robust standard errors. 720 households (360 per arm) from the parent trial were enrolled and made 9800 child observations between June 2014 and December 2016.
Interventions	Multicomponent sanitation intervention including periods with differing intensity of behavioural promotion: water, sanitation, hygiene, and nutrition interventions. The sanitation intervention included provision of or upgrades to improved latrines, sani‐scoops for faeces removal, children's potties, and in‐person behavioural promotion. Promotion was intensive up to 2 years after intervention initiation, decreased in intensity between years 2 to 3, and stopped after 3 years. The study period included approximately 1 year of high‐intensity promotion, 1 year of low‐intensity promotion, and 6 months with no promotion.
Outcomes	Diarrhoea and ARI, at 1 to 2 years after intervention implementation to 3.5 years (follow‐up). Outcomes were caregiver‐reported and there were limited data collected after promotion ceased.
Notes	Trial registration: ClinicalTrials.gov; NCT01590095; https://clinicaltrials.gov/ct2/show/NCT01590095

**Table:** Croke 2022

Methods	Cluster‐randomised trial assessing the effect of a national water, sanitation, and hygiene program on adherence with COVID‐19 policies in Congo. The trial is a follow‐up of the Villages et Ecoles Assainis programme which was running prior to the COVID‐19 pandemic.
Participants	332 communities were randomly assigned to the Villages et Ecoles Assainis program or control. (590/1312; 45%) individuals who owned phones were surveyed by phone 3 times between May 2020 to August 2021.
Interventions	Large‐scale water and sanitation programme not described in detail.
Outcomes	Primary outcomes were COVID symptoms, non‐ COVID illness symptoms, child health, psychological well‐being, and vaccine acceptance. Secondary outcomes included COVID‐19 preventive behaviour and knowledge, and perceptions of governmental performance, including COVID response. All outcomes were self‐reported. COVID symptoms were defined as the number of household members in the past week with fever, dry cough, difficulty breathing/shortness of breath, or fatigue, while non‐COVID illness variable was defined as the number of sick household members in the last 7 days (excluding those with COVID symptoms). The child health index was created using the proportion of children under 5 with fever/cough/diarrhoea in the last 2 weeks. The mental health index is a summary index of scores from answers to questions.
Notes	Cannot find NCT and unclear funders although acknowledgments list a potential load of funders. Probably public.

**Table:** Delaguerre 2022

Methods	Prospective, open‐label, non‐inferiority randomised (2:1), controlled trial
Participants	Study included healthy individuals aged 18 to 45 years, with negative RADT test 3 days prior to concert event, with no risk factors and not living with someone with risk factors, and residing in Paris. Study excluded people with positive RADT test within 3 days before the gathering. People with clinical signs suggestive of an infectious respiratory disease, or with risk factor for severe COVID‐19, or living with someone with risk factors for severe COVID‐19. Persons not covered by French National Health Insurance or who cannot stand for the duration of the experiment (about 5 hours from entry line to exit) were excluded. Person under legal guardianship, pregnant woman or woman orally declaring non‐use of effective contraception and breastfeeding woman were also excluded.
Interventions	Participants were randomly assigned to: medical face mask wearing during an indoor concert event, or not attending. Both groups had RADT test 3 days before the event Saliva samples for RT‐PCR were collected from both groups on D0 and D7 using self‐saliva‐collection kits
Outcomes	Primary outcome: the number of SARS‐CoV‐2‐positive RT‐PCR tests on self‐collected saliva at day 7. Secondary outcomes: the conversion rate of salivary carriage between the day 0 and day 7 visits; the percentages of adequately masked (nose and mouth covered) faces over the total 4‐hour period gathering.
Notes	French Ministry of Health. ITT and PP analysis were used. Several imputation for missing data. It is not clear if participants had COVID‐19 in the past (in the table with baseline characteristics it is reported quote: “”declared Covid‐19 history”: what does it mean? Surgical masks were worn also by all attendees, regardless of study participation? What is the intervention? Combined screening test + surgical mask?

**Table:** Loeb 2022

Methods	Multicentre, randomised, non‐inferiority trial
Participants	1009 healthcare workers who provided direct care to patients with suspected or confirmed COVID‐19. Conducted in 29 healthcare facilities in Canada, Israel, Pakistan, and Egypt from 4 May 2020 to 29 March 2022.
Interventions	Use of medical masks versus fit‐tested N95 respirators for 10 weeks, plus universal masking, which was the policy implemented at each site.
Outcomes	The primary outcome was confirmed COVID‐19 on reverse transcriptase polymerase chain reaction (RT‐PCR) test.
Notes	Financial support was given by the Canadian Institutes of Health Research, World Health Organization, and Juravinski Research Institute. Disclosures can be viewed at www.acponline.org/authors/icmje/ConflictOfInterestForms.do?msNum=M22-1966

**Table:** Varela 2022

Methods	Open‐label non‐inferiority randomised controlled trial
Participants	Study was conducted in Colombia Inclusion criteria: people aged > 18 years of both genders and who: (a) lived in a geographic area with active COVID‐19 transmission and in areas with medium, medium‐high, and high vulnerability index; and (b) worked outside their homes for at least 2 days during the last week. Exclusion criteria: retirement, unemployment, home‐based working, history of laboratory‐confirmed COVID‐19, working in health care, and daily N95 mask or face shield use. In addition, during follow‐up if participants reported an occupation change from work outside the home to home‐based work, or became unemployed
Interventions	Intervention group (IG): instructed to wear closed face shields with surgical face masks Active control group (ACG): instructed to wear only surgical face mask PPE was sent to their home address for each day of participation All participants received a follow‐up twice a week by phone All participants received recorded educational intervention via email or phone that provided recommendations about COVID‐19 prevention measures, guidance to ensure adherence, and appropriate handling of the assigned PPE. Weekly short questionnaire was performed on days 7, 14, and 21 to evaluate health status SARS‐CoV‐2 symptoms, PPE use, and adherence.
Outcomes	Primary outcome was the composite result of positive RT‐PCR or seroconversion during follow‐up Secondary outcomes including PPE use and adherence
Notes	Study was nested within an observational study (CoVIDA project). Funding was provided by donors administered by the philanthropy department at the Universidad de Los Andes, external financing from the United Nations Development Programme (UNDP), and donations of diagnostic material from the Engineering Services Laboratory S.A.S. (LABSERVING S.A.S. Colombia). Funders had no input on the study at any stage. Provided analysis as ITT and PP. Missing data were imputed with negative results.

ARI: acute respiratory infection h: hours ITT: intention‐to‐treat NCT: trial register number PPE: personal protective equipment PP: per protocol RADT: rapid antigen detection test RT‐PCR: reverse‐transcriptase polymerase chain reaction

### Characteristics of ongoing studies [ordered by study ID]

**Table:** Brass 2021

Study name	Prevention of SARS‐CoV‐2 (COVID‐19) transmission in residential aged care using ultraviolet light (PETRA)
Methods	A multicentre, 2‐arm double‐cross‐over, randomised controlled trial will be conducted to determine the efficacy of GUV devices to reduce respiratory viral transmission in RACF, as an adjunct to existing infection control measures. The study will be conducted in partnership with 3 aged care providers in metropolitan and regional South Australia. RACF will be separated into paired within‐site zones, then randomised to intervention order (GUV or control). The initial 6‐week period will be followed by a 2‐week washout before cross‐over to the second 6‐week period. After accounting for estimated within‐zone and within‐facility correlations of infection, and baseline infection rates (10 per 100 person‐days), a sample size of n = 8 zones (n = 40 residents/zone) will provide 89% power to detect a 50% reduction in symptomatic infection rate.
Participants	RACF within metropolitan and regional South Australia will be considered for recruitment if they possess the ability to sub‐divide communal living areas into discrete areas that enable a concurrent comparison of interventions, with the facility cohorts otherwise subject to the same facility practices (e.g. environmental cleaning, staffing, and social distancing).
Interventions	The intervention will involve the commercially available Laftech GUV appliances: UV‐FLOW‐C wall‐ and ceiling‐mounted system, UV‐FAN‐XS wall‐mounted air purifier, and UV‐FAN M2/95HP air purification device (LAF Technologies, Melbourne, Australia).
Outcomes	The primary outcome will be the incidence rate ratio of combined symptomatic respiratory infections for intervention versus control. Secondary outcomes include incidence rates of hospitalisation for complications associated with respiratory infection; respiratory virus detection in facility air and fomite samples; rates of laboratory‐confirmed respiratory illnesses and genomic characteristics.
Starting date	
Contact information	Andrew P. Shoubridge The South Australian Health and Medical Research Institute (SAHMRI), Adelaide, SA, Australia The Microbiome and Host Health Programme, College of Medicine and Public Health, Flinders University, Bedford Park, SA, Australia
Notes

**Table:** NCT03454009

Study name	Appropriate time‐interval application of alcohol hand gel on reducing influenza‐like illness amongst preschool children: a randomised, controlled trial
Methods	This is a comprehensive randomised cluster hand‐hygiene improvement intervention to reduce self‐reported ARI/ILI and GI illness, absenteeism, presenteeism and related behavioural and attitudinal change over a 90‐day trial. The intervention group will receive hand hygiene supplies and a variety of educational materials, including environmental posters in common areas. The control group will perform their usual hygiene activities and will not receive an intervention. Identical weekly surveys will be administered to the intervention and control groups to measure self‐reported illness, absenteeism, presenteeism, along with behaviour and attitudes measured at specified intervals during the study. The intervention and control groups were randomised by work floors before the onset of the enrolment period. It is hypothesised that employees in the intervention group will experience reduced self‐reported illness, absenteeism, and presenteeism along with improved protective hygiene behaviours and related attitudes, relative to those in the control group over the 90‐day trial.
Participants	Inclusion criteria At least 18 years of age or older No known allergies to alcohol or surface disinfecting wipes Works at least 30% of office hours at the study host site Consent to receiving emails from Kent State University Exclusion criteria Under 18 years of age Known allergies to alcohol or surface disinfecting wipes Works less than 30% of office hours at the study host site Does not consent to receiving emails from Kent State University
Interventions	The intervention group will receive hand hygiene supplies and a variety of educational materials, including environmental posters in common areas. The control group will perform their usual hygiene activities and will not receive an intervention.
Outcomes	Self‐reported ARI/ILI and GI illness, absenteeism, presenteeism and related behavioural and attitudinal change over a 90‐day trial
Starting date	5 February 2018
Contact information	Maggie Stedman‐Smith, PhD, Kent State University College of Public Health
Notes	Recruitment completed. Last update in ClinicalTrials.gov was 1 May 2019. NCT03454009

**Table:** NCT04267952

Study name	Hand hygiene intervention program on primary school students' health outcomes and absenteeism in school
Methods	Study Type: interventional (clinical trial) Estimated enrolment: 200 participants Allocation: randomised Intervention model: parallel assignment Masking: single (participant) Masking description: participation will not know whether they are in the experimental or control group
Participants	Inclusion criteria: primary school student (especially third‐ and fourth‐class student) Exclusion criteria: people with chronic disease
Interventions	Experimental: first group Hand hygiene intervention programme prepared by using planned behaviour theory will be applied to the students in this group. Active comparator: second group Students in this group will be given classic hand hygiene training.
Outcomes	Primary outcome measure: children with symptoms of infection will be referred to the family physician to have a rapid antigen test and to report the result to the researcher. 10 identified upper respiratory tract symptoms (fever, sore throat, runny nose, etc.) will be recorded weekly by family of children. The researcher will receive symptom information from the family via weekly SMS. The number of days the child does not attend school due to illness and the percentage of absenteeism Group A streptococcal infections in rapid antigen test (time frame: total 20 weeks) Incidence of symptoms of acute upper respiratory tract infection (time frame: total 20 weeks) School absenteeism (time frame: total 20 weeks) Secondary outcome measures: Glogerm gel applied hands will shine areas containing micro‐organisms. Contamination rate will be calculated by taking a photo of the hands and performing brightness analysis in Adobe Photoshop program. Pollution rate of hands (time frame: from date of randomisation until the date of first documented progression assessed up to 7 months)
Starting date	9 September 2019
Contact information	Contact: Uyanık +905068949969; gulcinyelten@hotmail.com
Notes	Recruitment is ongoing. Last update in ClinicalTrials.gov was 13 February 2020. NCT04267952

**Table:** NCT04471766

Study name	Evaluation of locally produced cloth face mask on COVID‐19 and respiratory illnesses prevention at the community level ‐ a cluster‐RCT
Methods	Study type: interventional (clinical trial) Estimated enrolment: 66,000 participants Allocation: randomised Intervention model: parallel assignment Masking: single (outcomes assessor) Primary purpose: prevention
Participants	Ages eligible for study: 10 years and older (child, adult, older adult) Sexes eligible for study: all Accepts healthy volunteers: no Criteria Inclusion criteria: Household resident Age 10 years and older Exclusion criteria: Refusal to participate
Interventions	Experimental: certified cloth face mask plus preventive information Active comparator: information on COVID‐19 prevention
Outcomes	Self‐reported main symptoms of COVID‐19 (3 or more of fever, cough, fatigue, shortness of breath, loss of smell/taste) Consultation for COVID‐19 like illness or reported positive test, or both Self reported COVID‐19 like illness plus hospitalisation or death Any death during the follow‐up period: Reported COVID‐19 like illness (time frame: 4 months' follow‐up) Consultation (time frame: 4 months' follow‐up) Severe illness (time frame: 4 months' follow‐up) Mortality (time frame: 4 months' follow‐up)
Starting date	Estimated study start date: July 2020
Contact information	Amabelia Rodrigues, PhD, 00245966078659; a.rodrigues@bandim.org
Notes	The number of cases of COVID‐19 is still increasing, and transmission of SARS‐CoV‐2 seems to occur mainly through person‐to‐person transmission through respiratory droplets, indirect contact with infected people and surfaces. The use of face masks is recommended as a public health measure, but in many settings only domestic cloth made masks are available to the majority of the people. However, masks can be of different quality, and very little is known about the utility of cloth face masks at the community level. In Bandim Health Project's Health and Demographic Surveillance System we evaluated the effect of providing locally produced cloth face masks on the severity of COVID‐19 like illness and mortality in an urban population. The locally produced cloth mask is made according to a laboratory‐certified model and was provided to the intervention group alongside information of how the risk of transmission can be reduced. The control group received information alone. Follow‐up will be implemented through telephone calls and post epidemic home visits.

ARI: acute respiratory tract infections GUV: germicidal ultraviolet ILI: influenza‐like illness GI: gastrointestinal n: number RACF: residential aged care facilities RCT: randomised controlled trial SARS: severe acute respiratory syndrome

## Differences between protocol and review

We changed the title of the review in 2010 (see Published notes below). For the 2020 update, we added one additional outcome: adverse events related to the intervention, and we split the outcomes into primary and secondary outcomes. We also focused only on randomised controlled trials (RCTs) and cluster‐RCTs and removed observational studies.
